# A single amino acid change led to structural and functional differentiation of *PvHd1* to control flowering in switchgrass

**DOI:** 10.1093/jxb/erad255

**Published:** 2023-07-04

**Authors:** Soyeon Choi, Pradeep K Prabhakar, Ratul Chowdhury, Thomas H Pendergast, Breeanna R Urbanowicz, Costas Maranas, Katrien M Devos

**Affiliations:** Department of Genetics, University of Georgia, Athens, GA 30602, USA; Complex Carbohydrate Research Center, University of Georgia, Athens, GA 30602, USA; Department of Biochemistry and Molecular Biology, University of Georgia, Athens, GA 30602, USA; Chemical Engineering, Penn State University, State College, PA 16801, USA; Department of Plant Biology, University of Georgia, Athens, GA 30602, USA; Department of Crop and Soil Sciences, University of Georgia, Athens, GA 30602, USA; Institute of Plant Breeding, Genetics and Genomics, University of Georgia, Athens, GA 30602, USA; Complex Carbohydrate Research Center, University of Georgia, Athens, GA 30602, USA; Department of Biochemistry and Molecular Biology, University of Georgia, Athens, GA 30602, USA; Chemical Engineering, Penn State University, State College, PA 16801, USA; Department of Plant Biology, University of Georgia, Athens, GA 30602, USA; Department of Crop and Soil Sciences, University of Georgia, Athens, GA 30602, USA; Institute of Plant Breeding, Genetics and Genomics, University of Georgia, Athens, GA 30602, USA; University College Dublin, Ireland

**Keywords:** Bioenergy, CONSTANS (CO), flowering time, Heading date 1 (Hd1), latitudinal adaptation, *Panicum virgatum*, protein structure and function, quantitative trait locus (QTL), single nucleotide polymorphism (SNP), switchgrass

## Abstract

Switchgrass, a forage and bioenergy crop, occurs as two main ecotypes with different but overlapping ranges of adaptation. The two ecotypes differ in a range of characteristics, including flowering time. Flowering time determines the duration of vegetative development and therefore biomass accumulation, a key trait in bioenergy crops. No causal variants for flowering time differences between switchgrass ecotypes have, as yet, been identified. In this study, we mapped a robust flowering time quantitative trait locus (QTL) on chromosome 4K in a biparental F_2_ population and characterized the flowering-associated transcription factor gene *PvHd1*, an ortholog of *CONSTANS* in Arabidopsis and *Heading date 1* in rice, as the underlying causal gene. Protein modeling predicted that a serine to glycine substitution at position 35 (p.S35G) in B-Box domain 1 greatly altered the global structure of the PvHd1 protein. The predicted variation in protein compactness was supported *in vitro* by a 4 °C shift in denaturation temperature. Overexpressing the *PvHd1-p.35S* allele in a late-flowering *CONSTANS*-null Arabidopsis mutant rescued earlier flowering, whereas *PvHd1-p.35G* had a reduced ability to promote flowering, demonstrating that the structural variation led to functional divergence. Our findings provide us with a tool to manipulate the timing of floral transition in switchgrass cultivars and, potentially, expand their cultivation range.

## Teaser

We uncovered a key gene that can be used to manipulate flowering time and biomass accumulation in switchgrass, a North America-native bioenergy crop, and demonstrate how a single variant can substantially change protein functionality.

## Introduction

Switchgrass (*Panicum virgatum* L.) is an outcrossing perennial grass native to North America and regarded as a promising high-yielding biofuel crop. Switchgrass can be classified into, mainly, later-flowering lowland and earlier-flowering upland ecotypes based on their latitudinal origins (Casler *et al.*, 2004, 2007). Since delayed floral transition in lowland ecotypes contributes to higher biomass production (van Esbroeck *et al.*, 1998), there is considerable interest in identifying the genetic variants governing the differential photoperiodic flowering time of the two ecotypes.

Flowering is initiated by integrating environmental signals such as photoperiod with internal cues such as circadian rhythms and hormones (Mouradov *et al.*, 2002). Photoperiod signals trigger expression of flowering pathway genes, such as the photoperiodic flowering pathway involving the three genes *GIGANTEA* (*GI*)–*CONSTANS* (*CO*)–*FLOWERING LOCUS T* (*FT*) first identified in the long-day model plant *Arabidopsis thaliana*. In Arabidopsis, *GI*, a photoperiodic oscillator gene, is activated when the daylength is longer than the critical daylength and induces expression of *FT* directly or in a CO-dependent manner (Koornneef *et al.*, 1991; Kobayashi *et al.*, 1999; Samach *et al.*, 2000; Suárez-López *et al.*, 2001; Mouradov *et al.*, 2002; Sawa and Kay, 2011; Golembeski *et al.*, 2014). Under long inductive daylengths, transcription of *CO*, which is regulated by the circadian clock, peaks at the end of the day, and expression of *FT* coincides with the expression pattern and stability of the CO protein throughout the day (Suárez-López *et al.*, 2001; Valverde *et al*., 2004; Golembeski *et al.*, 2014). FT moves to the shoot apex to initiate the transition of the shoot apical meristem (SAM) into a floral meristem by activating floral identity genes such as *APETALA1* (*AP1*) (Mouradov *et al.*, 2002; Corbesier *et al.*, 2007). This photoperiodic flowering pathway involving the three genes *GI*–*CO*–*FT* is highly conserved across species, as exemplified in rice (*Oryza sativa*, *Os*) and maize (*Zea mays*, *Zm*), by *OsGI*–*Heading date 1*–*Heading date 3a* (*Hd3a*) and *ZmGI*–*CONSTANS of Zea mays1* (*CONZ1*)–*ZEA CENTRORADIALIS 8* (*ZCN8*), respectively (Yano *et al.*, 2000; Kojima *et al.*, 2002; Hayama *et al.*, 2003; Miller *et al.*, 2008; Coles *et al.*, 2010; Meng *et al.*, 2011; Brambilla *et al.*, 2017; Guo *et al.*, 2018).

In switchgrass, overexpression of an *FT* ortholog, *P. virgatum FLOWERING LOCUS T1* (*PvFT1*), in the lowland switchgrass accession Alamo (2*n*=4*x*=36) led to earlier flowering, implying that transcript level differences, caused either by differential expression or copy number variation, of *FT* orthologs between the ecotypes could be a driver of differential flowering time (Niu *et al.*, 2016). This was supported by the finding of Taylor and colleagues that *FT* was expressed more highly in an early-flowering F_2_ progeny carrying an upland *FT* allele than in a later-flowering F_2_ progeny carrying a lowland allele (Taylor *et al.*, 2020). Another flowering-inducing gene, an ortholog of the Myb transcription factor gene *LUX ARRHYTHMO* (*LUX*) in Arabidopsis, has also been shown to be expressed more highly in uplands throughout development in both leaf and SAM tissues. Furthermore, in the SAM, *LUX* expression was activated earlier and reached the maximum level faster in uplands than in lowlands (Tornqvist *et al.*, 2017).

In addition to expression analyses of select genes involved in flowering, a number of studies have been conducted to identify chromosomal regions and loci associated with flowering time in switchgrass. A genome-wide association study (GWAS) across upland and lowland genotypes identified single nucleotide polymorphisms (SNPs) significantly associated with differential flowering time within and across the gene pools (Grabowski *et al.*, 2016). However, the flowering time-associated SNPs from the GWAS did not locate close to flowering time quantitative trait loci (QTLs) identified in biparental and four-way crosses scored for flowering time variation across 12 field sites located at different latitudes (Milano *et al.*, 2016; Tornqvist *et al.*, 2018; Lowry *et al.*, 2019). QTL-associated genes involved in flowering identified in the mapping studies included orthologs of *PSEUDO-RESPONSE REGULATOR 5* (*PRR5*), *FRIGIDA ESSENTIAL 1* (*FES1*), *FT*, and *FT-LIKE9/10* (*FTL9/10*) (Grabowski *et al.*, 2016; Tornqvist *et al.*, 2018).

Despite efforts to unravel the molecular mechanisms regulating the differential timing of floral transition in upland and lowland switchgrass ecotypes, the genetic variants that are key to this process are still unknown. None of the candidate flowering time genes identified in switchgrass (Milano *et al.*, 2016; Tornqvist *et al.*, 2017, 2018; Taylor *et al.*, 2018) has been functionally validated. Here, we report on the identification and functional validation of an ecotype-specific variant in the coding region of a gene involved in flowering, *PvHd1*, that contributes to an ~1 week difference in flowering time between the switchgrass lowland genotype AP13 and upland genotype VS16. The upland-specific amino acid (p.35S) is conserved across grasses, while the lowland allele (p.35G) is predicted to lead to a significant structural alteration of the protein. Differential functionality of the two alleles, driven by structural diversification, was validated in transgenic studies in Arabidopsis. Our study paves the way for directed manipulation of flowering time in switchgrass.

## Materials and methods

### QTL mapping for flowering time in a biparental F_2_ mapping population

Three replicated blocks (Reps 1, 2, and 3) of 330 F_2_ progeny from a biparental mapping population, generated by intercrossing two F_1_ progenies (lines 304 and 346) from a cross between the lowland genotype AP13 (a selection from the cultivar Alamo) and the upland genotype VS16 (a selection from the cultivar Summer), were established in 2015 (Rep1) and 2016 (Reps 2 and 3) at the Iron Horse Farm in Watkinsville, GA, USA (Qi *et al.*, 2021). The emergence of the first shoot after winter dormancy (spring emergence) and of the first panicle (ordinal heading date) was recorded every 2–3 d from January to April and from May to August, respectively, for three consecutive years from 2017 to 2019. The date of first protruding anthers in the first emerged panicle (ordinal anthesis date) was scored only in Rep2 in 2018 and 2019. Ordinal heading date, days to heading (number of days from spring emergence to heading), ordinal anthesis date, and days to anthesis (number of days from heading to anthesis) were used as phenotypes for QTL mapping using Windows QTL Cartographer V2.5_011 (Wang *et al.*, 2012) with a 10 cM window size, 1 cM walk speed, and default heritability of 0.8. The genetic map used in the QTL analyses was reported in Qi *et al*. (2021) and comprised 5154 genotyping-by-sequencing- (GBS) derived SNP markers distributed across the 18 switchgrass chromosomes, 3132 of which mapped to unique positions.

### Identification of gene candidates

The QTL region on Chr04K was narrowed down to the interval shared between overlapping QTLs that were consistently identified across the years and the blocks. The GBS markers flanking the shared QTL region were projected onto the switchgrass v5.1 genome (AP13) assembly (Lovell *et al.*, 2021), and functionally annotated genes in the target region that had Gene Ontology (GO) terms in the Uniprot database (www.uniprot.org) associated with ‘flowering time’ were identified. The corresponding VS16 genome sequences were extracted from the assembly provided pre-publication by the Department of Energy Joint Genome Institute and Thomas Juenger [University of Texas (UT)-Austin]. Genes with SNPs between AP13 and VS16 with predicted moderate (missense mutations) or high (nonsense and frameshift mutations) functional effects were retained as likely gene candidates. The transcript level of the gene candidates was compared between the lowland accessions Alamo (population from which AP13 was selected) and Kanlow, and the upland accessions Summer (population from which VS16 was selected) and Dacotah using RNA-seq data generated from the most recently emerged leaf from 13-week-old plants grown under long days and harvested 1 h after programmed daybreak (Qi *et al.*, 2021) (RNA samples under NCBI-SRA BioProject PRJNA713271), with normalization of the raw reads to averaged trimmed mean of M (TMM) values using edgeR (Robinson *et al.*, 2009; Chen *et al.*, 2016). The 13 week time point was selected based on growth chamber observations of differences in flowering time between upland and lowland accessions. It is possible, however, that this time point is not optimal for capturing transcriptional differences between the ecotypes for flowering-associated genes. The sequencing reads were aligned as described in Qi *et al.* (2021) and only the uniquely and concordantly mapped paired-end reads [tagged as ‘NH:i:1’ for uniquely mapped reads in the HISAT2 alignment (Kim *et al.*, 2019) and ‘0x2’ for concordant paired-end reads in SAMTools (Li *et al.*, 2009)] were used for the TMM normalization using command lines combined with parameters (for f in *.bam; do samtools view -h -f 0x2 $f | grep -P ‘^@ | NH:i:1\b’ | samtools view -h -b > $f.filt.bam; done) in SAMTools (Li *et al.*, 2009). Transcripts with raw read counts equal to 0 based on CPM (count per million reads) across the samples were removed and the TMM values were obtained using command lines of ‘calcNormFactors(method=”TMM”)’ and ‘cpm(normalized.lib.sizes=TRUE)’ in edgeR (Robinson *et al.*, 2009; Chen *et al.*, 2016) in R (R Core Team, 2022).

### Identification of subpopulation-wide allelic variants in PvHd1 and their correlation with flowering time

A switchgrass GWAS mapping panel consisting of 382 genotypes and the associated genome-wide SNPs were provided by Thomas Juenger (UT-Austin) (Lovell *et al.*, 2021). One replicate of the panel (Rep1) was planted at the Iron Horse Farm, Watkinsville, GA, USA in 2019 and a second replicate (Rep2) was planted in 2020. Spring emergence and flowering time were recorded in Rep1 in 2020, and in both Rep1 and Rep2 in 2021, as described for the biparental mapping population. Statistically significant differences in the average flowering time in 2021 of accessions belonging to different genetic subpopulations and subpopulations by ecotypes (subpopulation:ecotype) (Lovell *et al.*, 2021) were identified by pairwise post-hoc Tukey testing using the ‘emmeans’ package (Lenth, 2022) following a one-way and a two-way ANOVA, respectively, considering each Rep as a random effect variable in R (R Core Team, 2022) (version 4.2.0). The allelic status of the candidate heading date gene *PvHd1* in the GWAS panel was extracted from the provided genome-wide filtered SNPs.

### Protein structure modeling

Homology models were built by uploading the amino acid sequences into the SwissModel (Waterhouse *et al.*, 2018) server. Only the B-Box domain was resolved by the homology models while the rest of the model was built as a linear polypeptide. The models were equilibrated using an all-atom molecular dynamics (MD) simulation first using NAMD (Phillips *et al.*, 2005; Huang and MacKerell, 2013) and CHARMM36 (Huang and MacKerell, 2013) forcefield employing a 2 fs time step for 300 ns. The resulting structures at 300 ns were fed to the Fragment Guided (FG)-MD (Zhang *et al.*, 2011) online server of the I-TASSER (Yang *et al.*, 2014) suite of programs. The FG-MD simulations were run for 1000 ns for each protein. The resulting models at the end of a 1000 ns simulation were used as the final models.

### Plasmid construction and transformation of Agrobacterium tumefaciens strain GV3101

The T-binary vector, pCN-S-OX, a derivate of pCambia3300 (Cambia, https://cambia.org/), was used for overexpressing the lowland and upland *PvHd1* alleles in Arabidopsis under control of the 35S cauliflower mosaic virus (CaMV) promoter. The coding sequences (CDS) encoding the two ancestral and variant alleles of PvHd1 were acquired from AP13 and Summer genotypes (Supplementary Fig. S1). We will refer to the two proteins as ‘PvHd1-p.35G’ (with G being glycine) for the AP13 allele and ‘PvHd1-p.35S’ (with S being serine) for the Summer allele. The CDS with an added *Sac*I site immediately upstream of the start codon and a *Bam*HI site immediately downstream of the stop codon were synthesized (Twist Bioscience) and cloned into the *Sac*I/*Bam*HI-digested pCN-S-OX vector. Constructs were used to transform *Escherichia coli* 10β cells (NEB) and the presence of inserts in bacterial transformants was confirmed by PCR using ‘4KG163000_SacI’ (forward) and ‘4KG163000_BamHI’ (reverse) primers (Supplementary Table S1) with annealing at 50 °C for 25 s in GoTaq^®^ Flexi polymerase reactions (Promega). The cloned constructs were isolated from insert-positive colonies and verified for integrity by direct sequencing (Azenta Life Sciences).

*Agrobacterium tumefaciens* strain GV3101 was transformed with 300 ng to 1 µg of the sequence-verified overexpression constructs. The presence of the constructs was confirmed by PCR using ‘pS-OX_promoter_F’ (forward) and ‘pS-OX_RB_R’ (reverse) primers (Supplementary Table S1) with annealing at 57.9 °C for 15 s in Q5^®^ High-Fidelity DNA polymerase (NEB) reactions, and amplicons were sequenced (Azenta Life Sciences).

### Agrobacterium-mediated transformation of Arabidopsis

GV3101 transformants containing sequence-verified constructs were grown on LB plates supplemented with kanamycin (50 µg µl^–1^), gentamicin (30 µg µl^–1^), and rifampicin (50 µg µl^–1^) at 28 °C for 3 d. A single colony was inoculated in 5 ml of liquid LB with antibiotics and grown overnight with shaking at 220 rpm at 28 °C. The overnight culture was transferred to 100 ml of LB without antibiotics and grown until the OD_600 nm_ reached 0.3. Cells were spun down and resuspended in freshly made infiltration solution (5% sucrose, 10.5 mM MgCl_2_, 0.03% Silwet-77) to an OD_600 nm_ of ~1. Transformation of Arabidopsis lines Landsberg *erecta* (L*er*) and CS175 [*co-2* (*CO*-null) mutant in L*er* background (Koornneef *et al.*, 1991)] was performed by floral dip using a modification of the protocol established by Clough and Bent (1998). T_0_ plants were left in the dark overnight at 23 °C and then grown to maturity under 16 h daylengths at 23 °C with 70% humidity and 77 µmol of light in a growth chamber.

Matured T_1_ seeds were sterilized with isopropanol and 50% commercial bleach (Clorox, 3.7% sodium hypochlorite) containing 0.05% Triton X-100 (Bio-Rad), imbibed in 0.001× Basta (200 mg l^–1^ glufosinate-ammonium) (Finale), stratified in the dark at 4 °C for 3 d, and spread on humid soil (Sun Gro) in the growth chamber with no watering until germination. Green seedlings (transgene-positive) were sprayed with 0.001× Basta (Finale) three times a week to eliminate false positives. Presence of the *PvHd1* alleles was confirmed by PCR using the forward (‘pS-OX_promoter_F’) and reverse (‘pS-OX_RB_R’) primers (Supplementary Table S1) in Q5^®^ High-Fidelity DNA polymerase (NEB) reactions, and the nucleotides present at the 35th codon were verified by direct sequencing of the amplicons (Azenta Life Sciences). Sequence-verified T_1_ plants were grown to maturity in the growth chamber for harvesting of T_2_ seeds. Independent transformants carrying the switchgrass *PvHd1-p.35G* allele are referred to as ‘AP13_T1_X’ (X being the line number) and those carrying the *PvHd1-p.35S* allele as ‘Su_T1_X’. Presence of the *PvHd1* alleles in the T_2_ plants was confirmed by PCR using forward (‘PvHd1_inside_F’) and reverse (‘PvHd1_CDS_R’) primers (Supplementary Table S1) in GoTaq® Flexi polymerase reactions (Promega) with annealing at 55 °C for 15 s.

### Flowering time and expression level analyses in T_2_ transformants

Surface-sterilized and stratified seeds of L*er*, CS175 [*co-2*; *CO*-null in L*er* background (Koornneef *et al.*, 1991)], and transgenic lines (T_2_ generation) were grown in a growth chamber (16 h daylength/23 °C/70% humidity/77 µmol light).

To assess the effect of the presence and expression level of the *PvHd1-p.35G* and *PvHd1-p.35S* alleles on flowering time when transformed into the late-flowering *CO*-null Arabidopsis line CS175, flowering dates were recorded and the most recently fully expanded leaf after bolting was harvested from the three earliest-flowering and the three latest-flowering T_2_ plants from two independent harvest batches within each transgenic line 4 h after programmed daybreak. Statistically significant differences in flowering time between early- and late-flowering transformants relative to the *CO*-null and L*er* wild-type lines were determined by a post-hoc Tukey test using the ‘emmeans’ (Lenth, 2022) package in R (R Core Team, 2022) (version 4.2.0) following a one-way ANOVA which considered ‘Batch’ as a random variable.

For expression analysis, total RNA was extracted from the collected leaf samples using Trizol extraction followed by clean-up with the Zymo RNA Clean & Concentrator-5 kit (Zymo Research). The first strand of cDNA was synthesized from 400 ng to 1 µg of total RNA using the SuperScript™ IV First-Strand Synthesis kit (Invitrogen) according to the manufacturer’s instructions. A 60 ng aliquot of cDNA was used as template for real-time quantitative PCRs (qRT-PCRs) in 1× SsoAdvanced Universal SYBR Green Supermix (Bio-Rad) with 300 nM of primers. Primers used in this study were ‘ACN_F’/‘ACN_R’ (*ACT2* reference gene) and ‘PvHd1_CDS_F’/‘PvHd1_qPCR_R’ (Supplementary Table S1). The qPCR conditions were according to the manufacturer’s instructions with repeats of 38 cycles of annealing and extension at 50.4 °C for 45 s in a CFX96 Touch Real-Time PCR Detection System (Bio-Rad). The relative fold expression level of the genes across samples was estimated by the ddCq (ΔΔCq) method using *ACT2* (AT3G18780) as a reference gene (Rao *et al.*, 2013). Only a single reference gene was used to normalize the relative transcript abundance of *PvHd1* across the transgenic plants because all transgenic plants were in the same genetic background (homozygous *CO*-null in L*er*) and were grown in the same growth chamber under the same temperature/daylength/humidity conditions. If differences between the dCq (Cq_PvHd1_–Cq_Actin_) value of one of the technical replicates and those of the other two were >1, the dCq value of that technical replicate was removed for calculating averaged ddCq.

Because Arabidopsis T_2_ transformants, with the exception of lines ‘AP13_T1_3’, ‘AP13_T1_6’. and ‘Su_T1_3’, were twice independently harvested for the expression analysis, a one-way ANOVA was done within each line in R (R Core Team, 2022) (version 4.2.0) using ‘Batch’ as a fixed variable to test the significant effect of the variables on expression levels, which are averaged dCq values across three technical replicates. ‘Batch’ had no statistically significant effect on dCq except for ‘Su_T1_1’. Pearson’s correlation coefficients between days to flowering in the selected samples from each Arabidopsis line and the averaged ddCq (dCq relative to ‘AP13_T1_8_15’) values across technical replicates were calculated across batches in R (R Core Team, 2022) (version 4.2.0) except for ‘Su_T1_1’ where correlations were calculated within batches.

### Plasmid construction for expression of the PvHd1 protein variants in *E. coli*

For protein expression in *E. coli*, 50 ng of the synthesized *PvHd1* fragments were amplified using 240 nM of primers ‘attB1-pvHd1_F’ and ‘attB2-pvHd1_R’ (Supplementary Table S1) using the CloneAmp HiFi PCR Premix master mix (Takara). PCR conditions were initial denaturation at 98 °C for 30 s, 30 cycles of denaturation at 98 °C for 10 s, annealing at 51 °C for 10 s, and extension at 72 °C for 30 s, followed by a final extension at 72 °C for 2 min. The amplicons were purified using the Zymoclean Gel DNA Recovery Kit (Zymo Research). Purified amplicons were cloned into the pDONR221 vector (Invitrogen) using BP clonase II (Invitrogen) according to the manufacturer’s instructions. A 2 μl aliquot of the ligated products was used for transformation in *E. coli* 10β, and single transformed colonies were tested by PCR for the presence of the insert using the primer set ‘attB1_PvHd1_F’/‘PvHd1_stdiff_R3’ and Q5^®^ High-Fidelity DNA polymerase (NEB) (Supplementary Table S1). The constructs were miniprepped from the insert-positive colonies and sequenced (Azenta Life Sciences). The verified constructs were then transferred into the pDEST-HisMBP destination vector [Addgene ID 11085 (Nallamsetty *et al.*, 2005)] using Gateway® recombination cloning with LR clonase II (Invitrogen). The resulting expression construct encodes a fusion protein that consists of an N-terminal hexahistidine tag (His-tag), followed by maltose-binding protein (MBP), and then the full-length PvHd1 protein. His_6_MBP–PvHd1expression plasmids were extracted from ampicillin-resistant colonies using the E.Z.N.A.® Plasmid DNA Mini Kit I, (Q-spin) (Omega BIO-TEK).

### Protein purification and thermal shift assay

Extracted His_6_MBP–PvHd1expression plasmids were transformed in Tuner (DE3) *E. coli* competent cells. Transformed colonies were selected on LB plates with ampicillin (100 µg ml^–1^), used to generate 5 ml overnight cultures which, in turn, were used to inoculate 500 ml of LB supplemented with ampicillin (100 µg ml^–1^) and ZnCl_2_ (10 µM). Cultures were grown at 37 °C with shaking at 180 rpm until the OD_600 nm_ reached 0.8. Expression of *PvHd1* was then induced with 1 mM isopropyl-β-d-thiogalactopyranoside (IPTG) at 20 °C for 16 h. The induced cells were centrifuged at 6000 *g* for 10 min and resuspended in 30 ml of Buffer A (50 mM HEPES, 400 mM NaCl, 20 mM imidazole, pH 8). The cells were lysed using a French press and centrifuged at 15 000 *g* for 30 min. The supernatant was filtered using 5 µm filters and purified using a 1 ml HisTrap HP column (GE Healthcare) followed by size exclusion chromatography using a HiLoad 16/600 Superdex 200 pg (Cytiva) column according to previously published methods (Prabhakar *et al.*, 2020). Fractions containing the fusion protein were pooled and dialyzed into 50 mM HEPES, 100 mM NaCl at pH 8. The proteins were then concentrated using Pierce™ Protein Concentrators PES, 10K MWCO (Thermo Scientific) for further analysis.

For the thermal shift assay, 45 µl of purified fusion proteins (2.8 µM) were incubated in 1× SYPRO Orange Protein Gel Stain (ThermoFisher). The fluorescence intensity of the protein unfolding was measured in a CFX96 Touch Real-Time PCR Detection System (Bio-Rad) as the temperature increased from 25 °C to 100 °C in 1 °C increments. Each temperature was held for 30 s and increased by 5 °C s^–1^. Raw data were processed and analyzed to calculate the melting temperatures of the two proteins using the JavaScript Thermal Shift Analysis (JTSA) script (https://github.com/paulsbond/jtsa).

## Results

### A robust flowering time QTL was identified on Chr04K

**Fig. 1.**
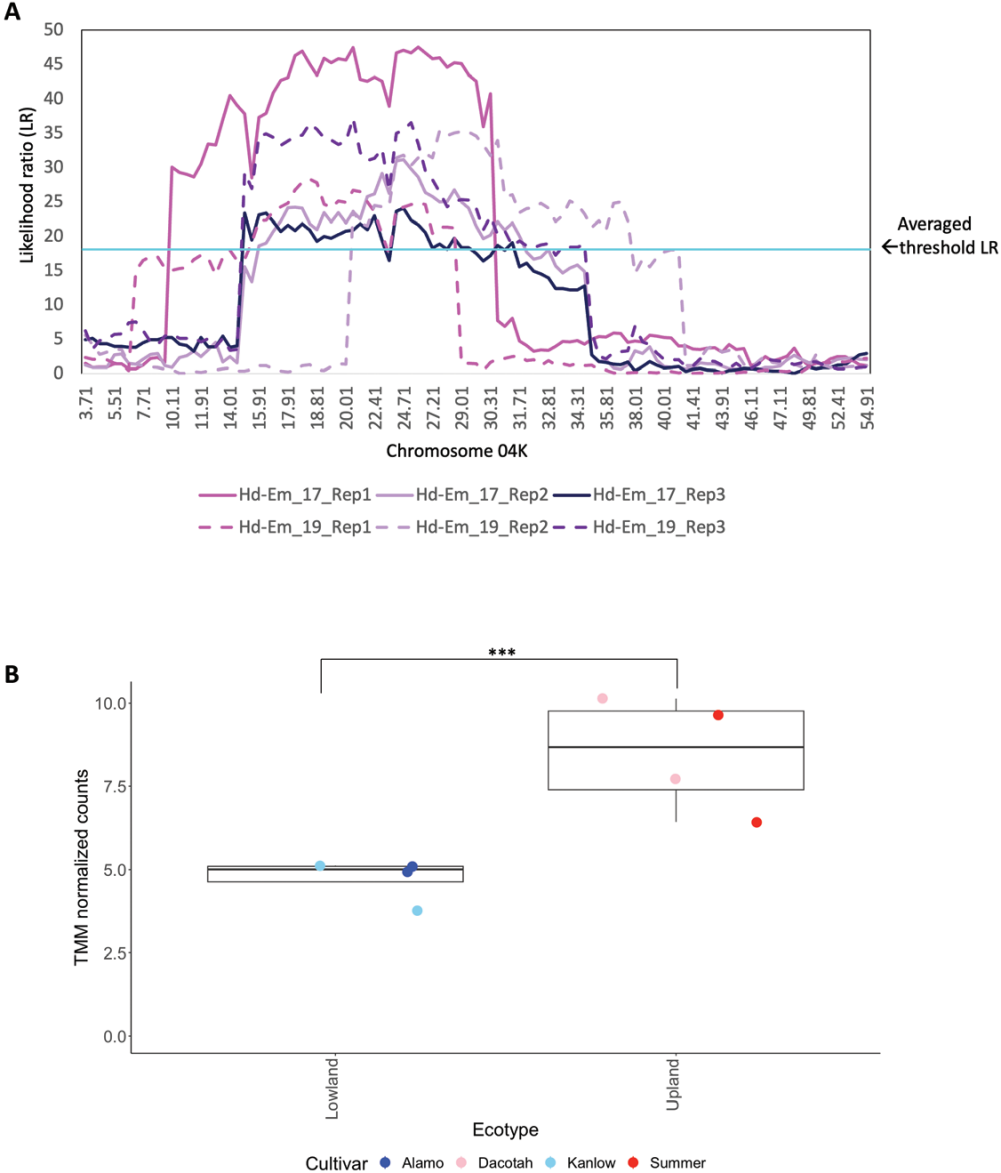
Days to heading QTL identified on chromosome 04K (Chr04K) and ecotype-specific expression levels of a gene candidate, *PvHd1* (Pavir.4KG163000), underlying the QTL. (A) Days to heading QTL on Chr04K. The *x*-axis indicates the genetic position of the markers on Chr04K and the *y*-axis indicates the likelihood ratio (LR) of association between the loci and ‘Days to heading’ in 2017 and 2019. The cyan horizontal line indicates the averaged threshold LR (range 17.7–19.2; average=18.5) at *P*=0.05 after 500 permutations across the QTL for the three replicates and the 2 years. The QTLs for all measured traits are presented in Supplementary Fig. S2. (B) TMM-normalized counts of *PvHd1* (Pavir.4KG163000) in four tetraploid switchgrass cultivars, Alamo (blue circles; lowland), Kanlow (light blue circles; lowland), Dacotah (pink circles; upland), and Summer (red circles; upland). The *PvHd1* transcripts in upland cultivars were significantly more abundant than in lowland cultivars (*P*=0.003).

A total of 16 QTLs were identified in a sib (F_1_304×F_1_346)-mated biparental lowland (AP13)×upland (VS16) F_2_ population across 3 years and three replicates for ordinal heading date (date of emergence of first panicle), 12 QTLs for days to heading (number of days from emergence of first leaf after winter dormancy to emergence of first panicle), six for ordinal anthesis date (date of first protruding anthers in the first emerged panicle), and four for days to anthesis (number of days from heading to anthesis) (Table 1). Of these, the QTL on chromosome 4K (Chr04K) was identified across all traits except days to anthesis, across multiple replicates and across multiple years, and explained between 7.4% and 19.8% of the phenotypic variation (Table 1; Fig. 1A; Supplementary Fig. S2). The phenotypic scores are provided in Supplementary Table S2. The lowland (AP13) allele at the Chr04K locus contributed to a delay of 2.1–8.6 d in panicle emergence and anthesis (Supplementary Table S3). The additive effects of the QTL were 1.4–4.2 d (Table 1). Nineteen other QTLs, on Chr05K, 05N, 06N, 07N, 08N, 09K, and 09N, were identified for at least two traits, replicates, or years, while the remaining QTLs were unique (Table 1).

**Table 1.** Identified flowering time QTL at *P*<0.05 for 2017–2019

Trait	Rep	Chr	*R* ^2^ at the highest LR (%)	Highest LR	Position (cM)	Left marker position (cM)	Right marker position (cM)	Additive effect^a^	Direction of allele effect
Ordinal heading date 2017	1	2K	5.8	19.8	8.6	7.6	11.7	–1.2	H>L≠U
	2	2N	0.7	23.1	85.8	85.2	87.2	2.3	L>U
	1; 2; 3	**4K**	16.6; 8.0; 10.9	47.3; 28.3; 34.4	25.8; 24.7; 24.7	16.0; 21.8; 14.3	36.8; 29.2; 32.8	4.2; 3.5; 4.2	L>U
	3	7N	10.6	28.8	17.3	7.1	30.0	3.1	L>U
	1; 2; 3	9K	8.4; 1.8; 5.4	27.8; 22.9; 20.4	16.6; 22.6; 20.5	11.8; 21.9; 18.9	29.2; 23.0; 20.6	3.2; 2.7; 3.1	L>U
Ordinal heading date 2018	2	2K	6.8	21.4	29.2	29.2	31.8	2.03	L>U
	3	3N	2.8	24.5	10.3	2.5	14.6	–1.8	L<U
	1; 3	5K	2.9;0.3	30.4; 21.2	48.2; 52.6	38.8; 52.1	54.5; 52.6	–2.0; –1.4	L<U
	1	5N	1.5	28.6;	6.3	4.4	15.7	1.8	L>U
	2	8N	3.6	19.4	42.3	42.3	43.8	1.8	L>U
Ordinal heading date 2019	1; 2; 3	**4K**	12.3; 17.7; 14.3	28.8; 43.1; 48.3	26.8; 30.6; 24.8	14.0; 13.1; 13.3	31.2; 33.3; 32.8	1.8; 2.4; 2.8	L>U
	2	5K	1.2	20.6	51.7	48.4	53.7	-1.5	L<U
	1; 2; 3	5N	0.7; 2.8; 4.5	24.7; 21.5; 27.3	93.8; 91.9; 84.0	84.0; 82.1; 80.9	99.5; 94.6; 91.9	1.6; 1.7;2.0	L>U
	1	6N	1.2	19.8	23.7	21.7	24.9	–1.5	L<U
	3	7N	6.1	20.5	40.9	18.3/37.9^b^	18.3/41.7^b^	0.8	H<U<L
	3	9N	6.2	23.2	68.7	67.9	72.5	–1.9	L<U
Days to heading 2017	1; 2; 3	**4K**	14.3; 8.2;7.4	47.5; 31.1; 24.0	25.8; 24.7; 24.7	10.1; 17.0; 14.3	30.3; 31.7; 31.2	4.2; 3.7; 3.7	L>U
	1; 2; 3	5K	10.6; 9.5; 5.3	54.5; 38.3; 24.8	46.3; 50.2; 46.3	35.9; 43.3; 42.3	56.1; 61.9; 53.7	–4.7; –4.1; –3.7	L<U
	1	5N	5.0	24.5	45.5	35.1	44.1	3.0	L>U
	3	7N	7.0	22.1	41.7	17.3/35.9^b^	18.3/44.0^b^	1.6	H<U<L
	2	8N	4.4	29.8	47.8	38.5	50.5	3.4	L>U
	1;2;3	9K	5.2; 7.7; 5.0	36.4; 34.6; 30.2	22.6; 19.4; 19.2	9.0; 11.5; 14.2	28.8; 30.3; 28.6	3.5; 3.7; 3.9	L>U
Days to heading 2018	3	2K	7.3	25.3	61.2	52.0	62.2	4.5	L>U
	1	5N	2.2	22.6	6.3	6.2	6.3	3.4	L>U
Days to heading 2019	1;2;3	**4K**	14.6; 13.4; 12.0	28.2; 35.2; 37.2	18.6; 29.0; 20.5	14.9; 20.5; 14.3	28.4; 47.1; 34.5	4.0; 3.4; 4.0	L>U
	2;3	5K	7.3; 10.9	20.3; 25.6	64.4; 45.8	54.5; 38.6	65.5; 55.6	–2.8; –2.9	L<U
	2;3	5N	8.2; 6.4	30.1; 31.3	26.7; 29.8	16.3; 14.7	35.1; 35.1	3.3; 3.6	L>U
	1	6N	4.6	27.1	16.3	14.3	25.8	–3.3	L<U
Ordinal anthesis date 2018	2	**4K**	10.4	22.3	23.3	14.9	26.8	1.4	L>U
Ordinal anthesis date 2019	2	1K	6.7	19.6	78.2	77.2	78.2	–1.3	L>U
	2	**4K**	19.8	59.6	28.4	18.2	38.2	3.7	L>U
	2	5N	4.0	28.4	82.1	75.4	93.1	2.4	L>U
	2	7N	3.8	22.7	10.5	10.0	18.3	2.4	L>U
	2	9N	3.9	21.8	102.8	101.7	104.1	2.3	L>U
Days to anthesis 2018	2	3K	10.2	24.0	39.4	38.3	47.7	–1.5	L<U
	2	9N	6.7	21.9	69.1	64.8	69.1	1.6	L>U
Days to anthesis 2019	2	**4K**	2.2	23.3	43.3	38.4	46.1	1.2	L>U
	2	5N	3.6	20.1	12.6	10.4	14.2	–0.03	H>L≠U

Rep, replicate (Block); Chr, chromosome; LR, likelihood ratio [LR=logarithm of the odds (LOD)×4.605] (Wang *et al.*, 2012).

^a^ Positive and negative additive effects indicate the amount of the contribution of AP13 (lowland, L) and VS16 (upland, U) alleles, respectively, to delayed flowering or anthesis.

^b^ Two QTL intervals above the threshold at distal locations on the same chromosome.

### *PvHd1* is a candidate gene for the Chr04K heading/anthesis QTL

Within the overlapping interval of the ‘heading’/‘days to heading’ and ‘anthesis’ QTL that mapped to Chr04K (Supplementary Fig. S2; 21.8–28.4 cM, 12 387 330–23 577 337 bp), 583 genes were annotated in the switchgrass genome annotation v5.1 (Lovell *et al.*, 2021) (Supplementary Table S4). Of these, five genes were identified that were potentially involved in the regulation of flowering based on their GO terms (Table 2; Supplementary Table S4). One of the five flowering-associated candidates, Pavir.4KG163000, homologous to Arabidopsis *CO* and rice *Hd1*, showed moderate but significantly different (two-tailed Student’s *t*-test *P*-value=0.003) expression levels in leaves of 13-week-old plants of two lowland accessions (Alamo and Kanlow; delayed flowering) compared with two upland accessions (Summer and Dacotah; earlier flowering) that were grown from seed under long days, and harvested 1 h after programmed daybreak (Table 2). Considering that *CO* and *Hd1* are light regulated and activate the mobile florigen *FT* responsible for floral induction (Samach *et al*., 2000; Suárez-López *et al.*, 2001; Valverde *et al*., 2004; Corbesier *et al.*, 2007), more abundant transcripts of Pavir.4KG163000 in uplands compared with lowlands may result in the functional threshold expression level needed to activate the florigen genes being met earlier, thereby contributing to or driving the earlier flowering of uplands compared with lowlands.

**Table 2.** Flowering-associated genes underlying the flowering time QTL on Chr04K

Gene	Start (bp)	End (bp)	Homologous protein name^a^ (organism)	AvgTMM_lowland (±SD) ^b^	AvgTMM_upland (±SD)^b^	SNP_AP13vsVS16^c^
Pavir.4KG120500	14 144 616	14 145 981	AT-hook motif nuclear-localized protein 29 (Arabidopsis)	0.31 ± 0.22	0.19 ± 0.31	Start codon substitution (c.1ATG>CTG)
Pavir.4KG129700	18 808 649	18 817 294	Protein ANTHESIS PROMOTING FACTOR 1 (Arabidopsis)	8.87 ± 1.60	8.36 ± 1.06	No substitution
Pavir.4KG134100	17 455 359	17 460 959	Zinc finger protein CONSTANS-LIKE 9 (Arabidopsis)	0.67 ± 0.33	0.94 ± 0.37	p.N65S (B-Box domain, conservative); p.V152A (conservative)
Pavir.4KG163000	19 029 204	19 031 483	Zinc finger protein Heading date 1 (rice)	4.73 ± 0.64^d^	8.48 ± 1.72^d^	p.E11K^H1^; p.S35G (B-Box domain 1, non-conservative); p.R59S^H1^ (B-Box domain 1, non-conservative); p.G134R^H1^ (non-conservative); p.ins167I^H1^ p.ins167N^H2^ p.del207Q p.A246D^H2^ (non-conservative)
Pavir.4KG209000	23 146 947	23 149 933	Phosphatidylinositol 4-kinase gamma 4 (rice)	31.57 ± 3.61	27.75 ± 3.27	p.Q171E^H1^ (ubiquitin domain, non-conservative) p.R188H^H1^ (ubiquitin domain, conservative) p.G345A^H2^ (PI3-PI4 kinase catalytic domain, conservative); p.C356S (PI3-PI4 kinase catalytic domain; non-conservative); p.N466D^H1^ (PI3-PI4 kinase catalytic domain, non-conservative); p.L488H^H1^ (PI3-PI4 kinase catalytic domain, non-conservative); p.V498A^H1^ (PI3-PI4 kinase catalytic domain, conservative); p.V504M (PI3-PI4 kinase catalytic domain, conservative)

^a^ The top hit of a BLASTP search against NCBI’s Uniprot (www.uniprot.org) database (threshold e-value=1e-5).

^b^ AvgTMM=averaged trimmed mean of M-values of the raw RNA-seq reads across the two lowland cultivars, Alamo and Kanlow, and across the two upland cultivars, Summer and Dacotah. RNA-seq data were generated from the most recently emerged leaf in 13-week-old plants grown in a growth chamber under long days. Leaves were harvested 1 h after programmed daybreak (Qi *et al*., 2021).

^c^ Amino acid variation within candidate gene-encoded proteins in AP13 (lowland, Alamo selection) compared with VS16 (upland, Summer selection) alleles; ^H1^ and ^H2^ indicate that the variant was identified only in haplotype 1 or haplotype 2, respectively, of the VS genome assembly.

^d^ Transcript levels were statistically different in the lowland ecotypes Alamo and Kanlow compared with the upland ecotypes Summer and Dacotah (two-tailed Student *t*-test in R followed by *F*-test in R, *P*-value 0.003).

To further assess Pavir.4KG163000 (*PvHd1*) as a causal candidate for switchgrass flowering time, variant analysis within the coding region was conducted in the AP13 and VS16 genome assemblies. AP13 and VS16 are clonally maintained selections from Alamo and Summer, respectively. The sequence comparison indicated the presence of eight amino acid substitutions differentiating the AP13 and V16 PvHd1 proteins (Table 2). Only the variants at positions 35 and 207 were homozygous in VS16. Both variants as well as the heterozygous (in VS16) variant p.ins167N were validated in a randomly selected Summer genotype grown from seed (Supplementary Fig. S1A, B). Position 35 in B-Box domain 1 was highly conserved across grasses, which carried serine at this position, similarly to VS16. VS16 therefore contains the ancestral *PvHd1* allele (Supplementary Fig. S1C). Similar analyses in the other candidate genes also revealed non-synonymous SNPs in functional domains, but all led to conservative amino acid changes or occurred in one haplotype only (Table 2). While this does not preclude these variants having functional effects, we focused our attention on Pavir.4KG163000 which displayed both expression variation (Fig. 1B) and a homozygous non-conservative amino acid substitution in a functional domain.

### The non-conservative amino acid substitution in B-Box domain 1 of PvHd1 is predicted to alter protein folding

**Fig. 2.**
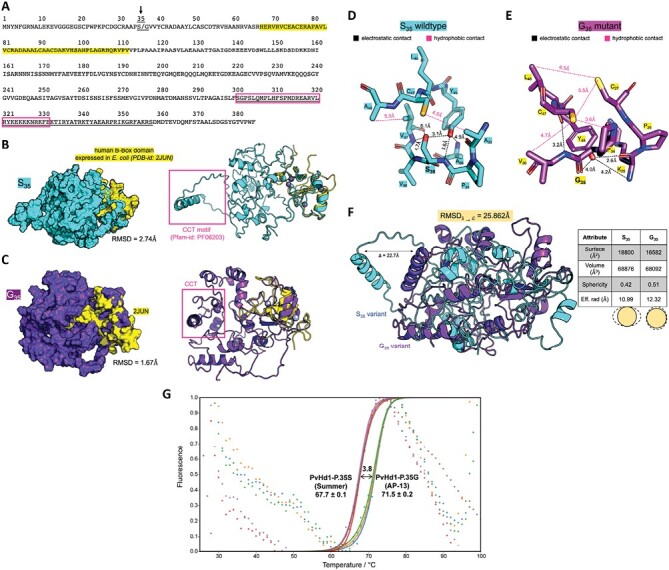
Modeling of the variation caused by the p.S35G amino acid substitution in PvHd1 and melting curves of PvHd1-p.35S and PvHd1-p.35G. (A) The amino acid sequences of the modeled PvHd1 protein highlighting the S/G mutation at the 35th position (shown with an arrow), B-Box domain 1 (yellow highlight), and CCT domian (underlined) as identified by IUPred3. The magenta box indicates the α-helix region shown in the magenta box in (B) and (C). (B, C) The 3D structural models of the PvHd1 proteins superposed with the human B-Box domain structure 2JUN. The S (B) and G variants (C) of PvHd1 show RMSDs 2.74 Å and 1.67 Å, respectively, when superposed on 2JUN. (D, E) The differential local structural packing of the amino acids around the 35th position in the S (D) and G variants (E) of PvHd1 highlighting the non-covalent electrostatic and hydrophobic contacts. (F) The 3D structural models of the S and G variants of the PvHd1 proteins show an RMSD of 25.862 Å when superposed globally upon one another. The helix of the CCT motif in the PvHd1 variants is structurally most different, with 22.7 Å distance between them. The table on the right shows the global structural characteristics of the PvHd1 protein variants. (G) Melting curves of the PvHd1-p.35G (three replicates; in blue, orange, and green) and PvHd1-p.35S (three replicates; in purple, red, and maroon) protein variants were generated using a thermal shift assay. Background dots are raw fluorescence intensities, and the fitted curves were drawn with solid lines. The three replicates of each protein variant clustered tightly.

To predict the effect of the non-conservative p.S35G substitution in B-Box domain 1 on the overall protein structure, protein modeling was conducted using the IPRO suite of programs (Pantazes *et al.*, 2014). IUPred3 (Erdős *et al.*, 2021) analysis of structural domains of the S variant and G variant revealed a zinc finger B-Box globular domain (Pfam-id: PF00643) and a CCT motif (Pfam-id: PF06203) in both proteins (Fig. 2A). Homology modeling followed by micro-second all-atom MD simulations (Supplementary Fig. S3; Fig. 2) showed that the B-Box domains of both proteins were structurally well defined and identical to an NMR structure of a human B-Box protein (PDB: - 2JUN) (Tao *et al.*, 2008) (Fig. 2B, C). The structures were all-helical except for the four small strands seen in the B-Box domains (Fig. 2B, C). In both proteins, an α-helix was seen in the CCT motif which was structurally distant from the B-Box domain (Fig. 2B, C; box inset colored magenta). To further shed light on the effect of the S/G mutation on the structural domains of the protein, both global and local parameters were traced. Locally, the variants had significantly different amino acid packing around the 35th residue, as indicated by the inter-residue non-covalent interactions (Fig. 2D, E). The global characteristics of the proteins indicate slightly different overall shapes for both (Fig. 2F). The G variant was ~20% more spherical and hence had an ~12% smaller surface area when compared with the S variant. Interestingly, the helix of the CCT motif was the most dissimilar (22.7 Å apart) between the two proteins (Fig. 2F). This structural difference may underpin functional differences between the protein variants.

The predicted difference in protein compactness was supported by the results of a thermal shift assay (Fig. 2G). The average melting temperature of the p.35S PvHd1 protein variant (PvHd1-p.35S; Supplementary Fig. S1B) expressed in *E. coli* was 67.7 °C (±0.1 °C), while that of the p.35G PvHd1 protein variant (PvHd1-p.35G; Supplementary Fig. S1B) was 71.5 °C (±0.2 °C) (Fig. 2G). The higher thermal energy needed to denature PvHd1-p.35G compared with PvHd1-p.35S suggests that the structure of PvHd1-p.35G is more condensed, as predicted by the IPRO model.

Although our predicted protein models of the PvHd1 alleles were supported by the *in vitro* thermal shift assay, it is important to stress that the accuracy of modeling predictions on protein structure rearrangements upon amino acid changes are contingent upon the accuracy of the employed force fields. Generally, as more amino acid changes are considered in the simulations, the confidence in the predicted structures diminishes. In addition, pH and/or temperature changes can have a significant effect on amino acid protonation states, electrostatic interactions, and the formation and strength of hydrogen bonds. Nevertheless, protein modeling is a useful tool that can inform the mechanistic reasons for the effect of amino acid changes in protein structures when used in combination with experimental studies. Moving forward, data-driven protein structure prediction using, for example, AlphaFold2 (Jumper *et al.*, 2021), RoseTTAFold (Baek *et al.*, 2021; Humphreys *et al.*, 2021), RGN2 (Chowdhury *et al.*, 2022), and ESMFold (Lin *et al.*, 2023), along with temperature- and pH-aware MD simulations could be used to resolve the effect of amino acid changes on structure and function, albeit at a much higher computational cost.

### The two *PvHd1* alleles differ in their ability to induce floral transition in Arabidopsis

**Fig. 3.**
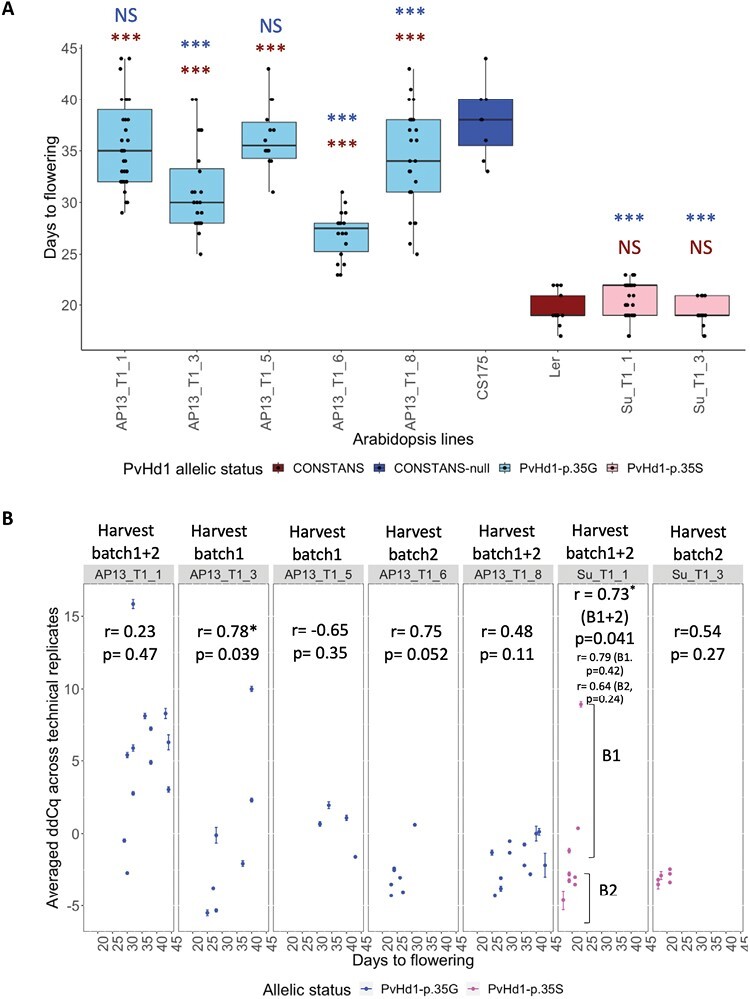
Flowering time and expression level of AP13_T1 and Su_T1 transgenic lines overexpressing *PvHd1-p.35G* and *PvHd1-p.35S*, respectively, in a *CO*-null background. (A) Box plot showing the average number of days between germination and bolting (‘Days to flowering’) in Arabidopsis wild-type L*er* (red bar), *CO*-null mutant CS175 (dark blue bar), and T_2_ transgenic progeny derived from lines Su_T1_1 and Su_T1_3 (overexpressing the switchgrass *PvHd1-p.35S* allele) (pink bars) and from lines AP13_T1_1, AP13_T1_3, AP13_T1_5, AP13_T1_6, and AP13_T1_8 (overexpressing the *PvHd1-p.35G* allele) (light blue bars). Black dots in the box plot represent individual data points. Significantly different flowering times of transgenic lines relative to L*er* are indicated in red and relative to CS175 in blue with ***P-value <0.001 and NS=not significant, acquired from Tukey post-hoc testing comparisons following a one-way ANOVA [‘Days to flowering’~‘Arabidopsis lines’+Error (‘Batch’)] (Supplementary Table S6). (B) Scatter plot showing the correlation between days to flowering and the average ddCq value across technical replicates for the 2–6 earliest flowering and the 2–6 latest flowering T_2_ progeny in each independent T_1_ line across two harvest batches (shown above the labels of each line) (Supplementary Table S7). For Su_T1_1, there was a significant ‘Batch’ effect on expression levels as determined by a one-way ANOVA, and data points were grouped by ‘Batch’. ddCq=difference in dCq value between each line and AP13_T1_8-derived line T_2__15 (AP13_T1_8_15, Batch1) with dCq=Cq_PvHd1_–Cq_Actin_; Cq indicates the number of PCR cycles at which fluorescence above the threshold was detected; therefore, more negative dCq values indicate a higher expression level of *PvHd1* relative to actin. Error bars on the dots show SDs of dCq across two or three technical replicates within each Arabidopsis line. *r*=Pearson’s correlation coefficient between the averaged Cq values and days to flowering. p=*P*-values for Pearson’s correlation test. *P-value of the correlation test <0.05.

The *PvHd1-p.35S* and *PvHd1-p.35G* alleles (Supplementary Fig. S1A) were overexpressed in the late-flowering Arabidopsis *CO*-null line CS175 (*co-2*). Five independent *PvHd1-p.35G*- and two independent *PvHd1-p.35S*-overexpressing (OE) T_1_ transformants were acquired and confirmed by sequencing. The *PvHd1-*containing T_2_ progeny from the two independent *PvHd1-p.35S* OE lines bolted, on average (±SD), 20.8 (±1.8) days after germination (dag) (Su_T1_1) and 19.2 (±1.4) dag (Su_T1_3). These values were similar to the average number of days to bolting in L*er* wild types (19.5 ± 1.8), and significantly earlier than bolting in the *CO*-null line, CS175 (37.9 ± 3.6) (Fig. 3A). This demonstrates that the *PvHd1-p.35S* allele can rescue the *CO* null mutation under flowering-promoting conditions (long daylength). On the other hand, *PvHd1*-positive T_2_ plants from all five independent *PvHd1-p.35G* OE lines bolted, on average, 35.7 ± 4.2 (line AP13_T1_1), 31.3 ± 4.1 (AP13_T1_3), 36.4 ± 3.1 (AP13_T1_5), 26.9 ± 2.3 (AP13_T1_6), and 34.1 ± 4.8 dag (AP13_T1_8), which is significantly later than the flowering of both L*er* wild types and *PvHd1-p.35S* T_2_ lines but similar (AP13_T1_1 and AP13_T1_5) or earlier (AP13_T1_3, AP13_T1_6 and AP13_T1_8) than CS175 (Fig. 3A; Supplementary Figs S4–S6; Supplementary Tables S5, S6), suggesting a reduced ability of the *PvHd1-p.35G* allele to induce flowering.

To confirm that the variation in flowering time between the AP13_T1 and Su_T1 transgenic lines was driven by the variant alleles, and not by differences in expression level, flowering time in the 2–6 earliest-flowering and the 2–6 most delayed-flowering T_2_ progeny in each independent transgenic line was correlated with transcript levels of the *PvHd1* allele. While there was a trend for higher expressing lines to flower earlier in about half of the transformants, the correlation was significant only in AP13_T1_3 and Su_T1_1 (Fig. 3B). When comparing flowering time across simultaneously grown lines, *PvHd1-p.35S* OE lines consistently flowered earlier than *PvHd1-p.35G* OE lines despite several *p.35G* lines having similarly high or higher expression levels (negative ddCq values) than *p.35S* lines (Fig. 3B; Supplementary Table S7). This indicates that *PvHd1-p.35S* has higher functionality than *PvHd1-p.35G* and that the structure of the protein rather than the expression level is the prime determinant of the observed flowering time differences.

### Distribution of *PvHd1* alleles on Chr04K across switchgrass germplasm

**Fig. 4.**
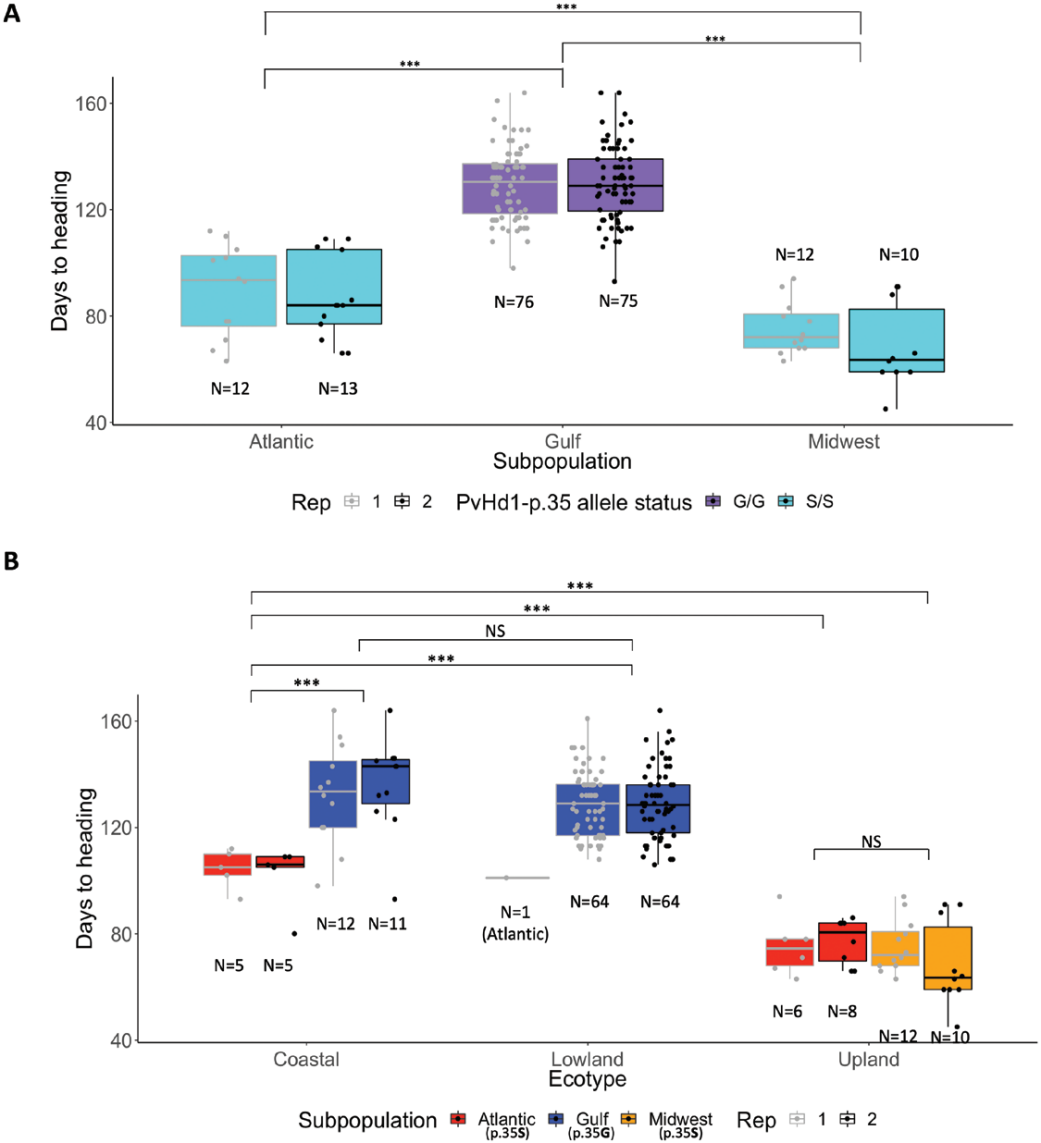
*PvHd1-p35* allele status and flowering time in switchgrass by genetic subpopulation and ecotype. (A) Box plot showing days to heading recorded in 2021 (Rep1 and Rep2) for accessions belonging to three genetic subpopulations in a GWAS panel established at the Iron Horse Farm in Watkinsville, GA. Gray and black dots in the box plot represent individual data points for Rep1 and Rep2, respectively. Subpopulation membership was obtained from Lovell *et al.* (2021). (B) Same data as in (A), shown for the three ecotypes (Lovell *et al.*, 2021) within each subpopulation. Allelic status of *PvHd1* in each subpopulation is present in parentheses under the color codes for the subpopulations. Statistically significant differences between the comparisons are shown as *P*-values based on the results from pairwise post-hoc Tukey tests across the samples combining both Reps. *P*-values <0.001 are indicated with ***. NS=not significant.

Lovell *et al*. (2021) previously showed that switchgrass accessions can be classified morphologically into three ecotypes, upland, lowland, and coastal, and that the ecotypic classification does not correspond to the three genetic subpopulations, Atlantic, Midwest, and Gulf. Analysis of the *PvHd1* alleles across a subset of the switchgrass GWAS panel sequenced by Lovell *et al*. (2021) and grown at the Iron Horse Farm in Watkinsville, GA, showed that the glycine observed in lowland accession AP13 at position 35 (*PvHd1-p.35G* allele) was fixed across accessions within the Gulf subpopulation, while the ancestral serine (*PvHd1-p.35S* allele) was fixed across accessions belonging to the Atlantic and Midwest subpopulations (Fig. 4A; Supplementary Fig. S7). Accessions in the Gulf subpopulation flowered significantly later than accessions in the Atlantic and Midwest subpopulations (Fig. 4A; Supplementary Fig. S7; Supplementary Tables S8, S9). When considering the different ecotypes within each subpopulation, coastal and lowland ecotypes that carried the *PvHd1-p.35G* allele (Gulf subpopulation) flowered significantly later than the coastal and lowland ecotypes that carried the *PvHd1-p.35S* allele (Atlantic subpopulation) (Fig. 4B; Supplementary Fig. S8; Supplementary Tables S8, S10). Within a subpopulation, the number of days to heading was similar for coastal and lowland ecotypes and, as can be observed within the Atlantic subpopulation, significantly higher than for upland ecotypes, despite these accessions all carrying the same *PvHd1* allele. These results indicate that, while flowering time in switchgrass is significantly associated with the allele status of *PvHd1*, other genes also play a role in the differential flowering of switchgrass ecotypes.

## Discussion

### The QTL on Chr04K can be exploited to manipulate flowering time in switchgrass

One strategy to meet the industrial demand for short hydrocarbons is to increase the biomass production of lignocellulosic feedstocks, including switchgrass. Because enhancing biomass accumulation in switchgrass can be achieved by delaying flowering time (van Esbroeck *et al.*, 1998), identifying the genetic loci contributing to the differential floral transition between the early-flowering upland accessions and the later-flowering lowland accessions has been the focus of multiple studies (Grabowski *et al.*, 2016; Milano *et al.*, 2016; Tornqvist *et al.*, 2017, 2018; Taylor *et al.*, 2018, 2020; Lowry *et al.*, 2019). Flowering time QTLs on Chr04K have been mapped in several upland–lowland segregating mapping populations, including in our study (Tornqvist *et al.*, 2018; Lowry *et al.*, 2019). As in our study, the lowland alleles were associated with delayed flowering (Tornqvist *et al.*, 2018; Lowry *et al.*, 2019). Although we could not determine whether the flowering time QTLs in these studies overlapped with the Chr04K QTL identified in our study, they may well represent the same locus. The causal gene and variants for the Chr04K QTL, however, have not previously been identified.

### The heading date gene *PvHd1* underlies the Chr04K flowering time QTL

We identified Pavir.4KG163000 (*PvHd1*) as the causal gene to the Chr04K flowering QTL identified in our study. While Pavir.4KG163000 transcript levels were higher in upland than in lowland accessions, our transformation data in Arabidopsis indicate that a p.S35G substitution is key to reducing the ability of Pavir.4KG163000 to induce flowering. The transcript level variation may act in synergy with the effects of the structural variation, but it is also possible that the lower transcript levels in lowlands are a consequence of faster decay through the nonsense-mediated RNA decay pathway that acts on the less functional mutant *p.35G* allele (Baker and Parker, 2004; Palacios, 2012; Nickless *et al.*, 2017; Kurosaki *et al.*, 2019). Overexpression of *PvHd1-p.35S* in an Arabidopsis *CO*-null line resulted in, on average, a 12 d acceleration in flowering time compared with overexpression of the *PvHd1-p.35G* allele. The allelic effect of the Chr04K QTL in switchgrass was 2.1–8.6 d in our study and 5 d in Tornqvist *et al*. (2018). Both Tornqvist *et al*. (2018) and Lowry *et al.* (2019) noted that the effect size of the QTL differed across latitudes, suggesting that functionality of the *PvHd1* alleles is affected by G×E interactions which can be mostly explained by latitudinal difference-reflecting environmental factors such as photoperiod. The effect size may also vary depending on the genetic background of the switchgrass accessions. The variant in B-Box domain 1 in *PvHd1* was fixed within subpopulations and significantly associated with differential flowering time. All accessions analyzed that belonged to the Midwest and Atlantic subpopulations were homozygous for serine at position 35 in PvHd1 on switchgrass Chr04K while those in the Gulf subpopulation had glycine (Fig. 4). Plants that have PvHd1-p.35S flowered earlier than plants with PvHd1-p.35G; however, flowering time was also significantly different between subpopulations with the same allelic status (e.g. Atlantic and Midwest; Fig. 4A) and between ecotypes within a subpopulation (e.g. upland and coastal within the Atlantic subpopulation; Fig. 4B). This suggests that differential interactions between PvHd1 and its partner proteins or other genes involved in flowering can synergistically contribute to the effect of *PvHd1* alleles on flowering time.

### Potential effects of the two alleles of *PvHd1* on expression of florigen genes

Our data collectively show that PvHd1-p.35S is a fully functional protein, while PvHd1-p.35G has a strongly reduced ability to induce flowering, probably because the changes in the 3D structure caused by the non-conservative amino acid substitution in the B-Box domain 1 impede physical interactions with its partner proteins. CO in Arabidopsis, Hd1 in rice, and their orthologs in other flowering species have been shown to bind to CO-responsive element (CORE) motifs in the promoter of florigen genes such as *FT* in Arabidopsis and *Hd3a* in rice. This binding is enhanced by the formation of heterotrimers with a nuclear factor Y (NF-Y) subunit beta (NF-YB) and subunit gamma (NF-YC) complex that binds to a distal *CCAAT* enhancer motif in *FT* (Wenkel *et al.*, 2006; Tiwari *et al.*, 2010; Cao *et al.*, 2014; Liu *et al.*, 2017; Sang *et al.*, 2019; Shen *et al.*, 2020; Lv *et al.*, 2021). The interaction between CO/Hd1 and the NF-Y complexes is mediated via the α-helix 1 and loop 1 secondary structures of the CCT domain in CO/Hd1 (Shen *et al.*, 2020; Lv *et al.*, 2021). Based on the predicted 3D model of the two alleles of PvHd1 (Fig. 2), the p.S35G variant in B-Box domain 1 greatly affects the relative position of the neighboring CCT domain, specifically α-helix 1 and loop 1 (Fig. 2B–F). The α-helix 1 and loop 1 of the ancestral PvHd1-p.35S allele are predicted to face outwards, while they are buried inside the PvHd1-p.35G protein (difference of 22.7 Å between the p.35S and p.35G proteins; Fig. 2F), leading to the hypothesis that the internalization of the CCT region in PvHd1-p.35G represses its interaction with the partner NF-Y complexes. The weak and unstable docking of the heterotrimer transcription complexes is then assumed to activate *FT* less efficiently, so that it takes longer for FT to reach the minimum threshold to induce floral transition.

### Conclusion

Our study identified *PvHd1* as the causal gene for a flowering time QTL on switchgrass chromosome 4K. While *PvHd1* is an ortholog of the known flowering time genes *CO* in Arabidopsis and *Hd1* in rice, our study demonstrates that a single missense mutation leading to a non-conservative amino acid substitution (p.S35G) in the B-Box domain 1 is sufficient to change the 3D structure and functionality of the PvHd1 protein. We now have a tool in hand to manipulate switchgrass flowering time. We envisage that knock out of the *PvHd1* gene copy in upland switchgrass will generate plants with an extended vegetative period of a few days that will increase biomass production at northern latitudes but still allow them to senesce before the onset of winter. It is possible that flowering time can be further modulated by replacing the *PvHd1-p.35S* allele in upland switchgrass with the *PvHd1-p.35G* allele, which encodes a protein with reduced functionality. Conversely, introgression of the *PvHd1-p.35S* allele in a lowland background can lead to earlier flowering, allowing the plants to senescence earlier to prepare for the upcoming winter, which may help with cold tolerance when grown in northern regions. Controlling floral transition will ultimately broaden switchgrass cultivation areas, leading to increases in biomass production.

## Supplementary data

The following supplementary data are available at *JXB* online.

Table S1. Primers used in the study.

Table S2. Flowering time traits measured for three consecutive years (2017–2019) in a biparental (AP13×VS16) F_2_ mapping population planted at the Iron Horse Farm in Watkinsville, GA.

Table S3. Average trait values for plants homozygous for the AP13 allele (delayed-flowering), homozygous for the VS16 allele (earlier-flowering), and heterozygous at the flowering time QTL on Chr04K.

Table S4. Annotated genes underlying the flowering time QTL on chromosome 4K.

Table S5. Flowering time of *PvHd1* allele-overexpressing T_2_ plants in *CO*-null L*er*, L*er*, and CS175 (*CO*-null).

Table S6. Post-hoc Tukey testing results showing the significance of differences in flowering time of *PvHd1*-OE transgenic lines relative to L*er* and CS175 (data shown in Fig. 3A).

Table S7. Cq values measured in *PvHd1*-overexpressing T_2_ transgenic plants in *CO*-null L*er* plants.

Table S8. Days to heading in a switchgrass diversity panel and the p.35 variant present in PvHd1 which locates at position 19 031 195 on Chr04K in the AP13 genome assembly v5.1.

Table S9. Post-hoc Tukey testing results showing the significance of differences in days to heading recorded in 2021 (Rep1 and Rep2) for accessions belonging to three genetic subpopulations in a GWAS panel established at the Iron Horse Farm in Watkinsville, GA (data shown in Fig. 4A).

Table S10. Post-hoc Tukey testing results showing the significance of differences in days to heading recorded in 2021 (Rep1 and Rep2) for accessions belonging to three ecotypes within genetic subpopulations in a GWAS panel established at the Iron Horse Farm in Watkinsville, GA (data shown in Fig. 4B).

Fig. S1. Amino acid and nucleic acid variation in PvHd1.

Fig. S2. Flowering time QTL identified on Chr04K.

Fig. S3. The amino acid sequence of the proteins used to build homology models.

Fig. S4. Box plot showing bolting in Arabidopsis wild type (L*er*), *CO*-null mutant (CS175), and T_2_ transgenic progenies.

Fig. S5. Representative phenotypes at bolting of different Arabidopsis lines and T_2_ transformants.

Fig. S6. Leaf numbers at bolting in the Arabidopsis lines and their correlation with ‘days to flowering’.

Fig. S7. Box plot showing days to heading recorded in 2020 (Rep1) and 2021 (Rep1 and Rep2) for accessions belonging to three genetic subpopulations in a GWAS panel established at the Iron Horse Farm in Watkinsville, GA.

Fig. S8. Box plot showing days to heading recorded in 2020 (Rep1) and 2021 (Rep1 and Rep2) for accessions belonging to three ecotypes within genetic subpopulations in a GWAS panel established at the Iron Horse Farm in Watkinsville, GA.

erad255_suppl_Supplementary_Figures_S1-S8

erad255_suppl_Supplementary_Tables_S1_S3_S6_S9-10

erad255_suppl_Supplementary_Tables_S2

erad255_suppl_Supplementary_Tables_S4

erad255_suppl_Supplementary_Tables_S5

erad255_suppl_Supplementary_Tables_S7

erad255_suppl_Supplementary_Tables_S8

## Data Availability

All raw data used in this study are included in the supplementary data or have previously been reported (genetic maps) and/or uploaded to NCBI’s SRA (RNA-seq data; BioProject PRJNA713271).
