# Supplementary material for: A single amino acid change led to structural and functional differentiation of PvHd1 to control flowering in switchgrass
Source: J Exp Bot. 2023 Jul 4;74(18):5532–46. doi: 10.1093/jxb/erad255 (PMC10540729; doi:10.1093/jxb/erad255)
Supplement: erad255_suppl_Supplementary_Figures_S1-S8 [file erad255_suppl_supplementary_figures_s1-s8.pdf]

Supplementary Table S1. Primers used in the study

**Primer_name**	**Sequence (5'- -3')**
4KG163000_SacI	gcagagctcATGAATTATA
4KG163000_BamHI	tgcggatccTCAGAACCAT
pS-OX_promoter_F	CAGTGGTCCCAAAGATGGAC
pS-OX_RB_R	CCGCCAATATATCCTGTCA
PvHd1_inside_F	TCAGTGCTTACACCGATTCC
PvHd1_CDS_R	TCAGAACCATGGGACAGT
ACN_F	ACCTTTAACTCTCCCGCTA
ACN_R	CAAGGTCAAGACGGAGGAT
PvHd1_CDS_F	ATGAATTATAATTTTGGCAG
PvHd1_qPCR_R	CAGAGGTACGCAGCGTCAG
attB1-pvHd1_F	GGGGACAAGTTTGTACAAAAAAGCAGGCTCCATGAATTATAATTTTGGCAG
attB2-pvHd1_R	GGGGACCACTTTGTACAAGAAAGCTGGGTCTCAGAACCATGGGACAGT
PvHd1_stdiff_R3	CAGAGTGCACCTTGGCGTCA

Supplementary Table S2. Flowering time traits measured for three consecutive years (2017-2019) in a biparental (AP13 x VS16) F_2 mapping population (Qi *et al.* , 2021) planted at the Iron Horse Farm in Watkinsville, GA

Genotype	Em 2017 Re	Em 2017 Re	Em 2017 Re	Hd 2017 Re	Hd 2017 Re	Hd 2017 Re	Hd Em 2017	Hd Em 2017	Hd Em 2017	Em 2018 Re	Em 2018 Re	Em 2018 Re	Em 2019 Re	Em 2019 Re	Em 2019 Re	Hd 2018 Re	Hd 2018 Re	Hd 2018 Re	Hd 2019 Re	Hd 2019 Re	Hd 2019 Re	Hd Em 2018	Hd Em 2018	Hd Em 2018	Hd Em 2019	Hd Em 2019	Hd Em 2019	An 2018 Re	An 2019 Re	An Hd 2018	An Hd 2019 Rep2
B10B	80	76	83	142	142	151	62	66	68	66	89	79	73	NA	71	166	162	166	158	154	156	100	73	87	85	NA	85	185	170	27	16
B10C	90	90	NA	160	160	NA	70	70	NA	NA	79	NA	NA	84	NA	NA	162	NA	NA	158	NA	NA	83	NA	NA	74	NA	185	175	25	17
B10D	87	94	90	156	160	160	69	66	70	72	79	79	80	84	87	163	162	165	161	156	156	91	83	86	81	72	69	182	175	22	19
B10E	83	73	80	156	160	165	73	87	85	66	72	66	71	73	73	169	169	166	165	163	168	103	97	100	94	90	95	182	177	16	14
B10F	83	80	80	160	172	153	77	92	73	66	99	79	87	65	77	169	180	173	168	168	165	103	81	94	81	103	88	NA	184	NA	16
B10G	87	76	87	167	165	167	80	89	80	79	61	66	73	65	73	173	173	171	168	168	168	94	112	105	95	103	95	188	184	16	16
B10I	87	80	83	147	147	142	60	67	59	79	72	72	80	NA	NA	165	166	169	161	156	161	86	94	97	81	NA	NA	182	172	17	16
B10J	90	90	94	147	151	153	57	61	59	66	72	79	91	84	87	166	164	178	179	165	158	100	92	99	88	81	71	185	184	22	19
B10K	87	83	83	165	165	165	78	82	82	79	61	66	NA	80	65	165	164	169	161	163	168	86	103	103	NA	83	103	185	182	23	19
B11A	90	87	97	172	172	NA	82	85	NA	79	72	NA	87	84	NA	171	173	NA	170	170	NA	92	101	NA	83	86	NA	190	186	18	16
B11B	73	80	80	165	172	167	92	92	87	66	66	66	71	73	73	171	178	171	172	168	170	105	112	105	101	95	97	191	184	16	16
B11C	83	80	87	160	165	165	77	85	78	66	66	66	84	77	77	163	162	164	163	163	158	97	96	98	79	86	81	188	184	30	21
B11D	76	76	73	147	142	142	71	66	69	61	66	61	71	NA	NA	165	163	164	158	156	156	104	97	103	87	NA	NA	185	172	26	16
B11E	83	76	80	156	156	160	73	80	80	66	61	66	73	77	73	NA	169	169	168	168	168	NA	108	103	95	91	95	188	179	23	11
B11F	76	80	80	156	156	160	80	76	80	72	61	72	71	71	71	171	166	169	161	163	170	99	105	97	90	92	99	186	182	21	19
B11G	87	73	80	172	165	165	85	92	85	72	72	66	80	84	77	171	169	165	168	172	168	99	97	99	88	88	91	190	184	24	12
B11H	83	76	101	147	147	NA	64	71	NA	89	79	NA	77	NA	NA	176	171	NA	168	168	NA	87	92	NA	91	NA	NA	184	175	17	7
B11I	83	NA	80	147	NA	142	64	NA	62	66	NA	72	77	NA	NA	166	NA	169	154	NA	156	100	NA	97	77	NA	NA	NA	NA	NA	NA
B11J	80	87	80	167	174	167	87	87	87	79	79	82	NA	84	77	173	176	173	170	172	170	94	97	91	NA	88	93	204	189	29	17
B11K	87	83	87	153	156	153	66	73	66	66	66	66	NA	73	73	160	160	164	158	156	156	94	94	98	NA	83	83	185	172	26	16
B12B	73	73	80	138	142	142	65	69	62	66	61	61	67	NA	NA	169	169	169	161	158	161	103	108	108	94	NA	NA	190	177	24	19
B12C	76	76	83	142	142	151	66	66	68	82	72	79	84	NA	84	169	166	162	163	158	165	87	94	83	79	NA	81	188	172	23	14
B12D	73	80	73	165	165	165	92	85	92	66	66	61	71	71	71	165	165	166	161	161	156	99	99	105	90	90	85	188	179	24	18
B12E	83	73	76	138	147	138	55	74	62	61	61	61	71	NA	NA	165	162	164	161	161	156	104	101	103	90	NA	NA	182	177	21	16
B12F	80	80	80	165	174	165	85	94	85	79	79	79	80	71	98	176	194	178	172	NA	NA	97	115	99	92	NA	NA	NA	NA	NA	NA
B12G	73	73	73	167	165	172	94	92	99	61	61	61	71	73	71	173	169	176	168	165	165	112	108	115	97	92	94	186	182	21	17
B12H	83	NA	NA	160	NA	NA	77	NA	NA	66	NA	NA	NA	73	NA	160	NA	NA	161	NA	NA	94	NA	NA	NA	NA	NA	NA	NA	NA	NA
B12I	80	83	80	172	167	167	92	84	87	54	61	61	NA	71	71	176	171	171	172	168	168	122	110	110	NA	97	97	NA	182	NA	14
B12J	73	76	80	160	160	160	87	84	80	72	61	66	NA	73	71	169	166	169	168	163	163	97	105	103	NA	90	92	186	182	21	19
B12K	80	80	80	167	167	165	87	87	85	66	79	61	NA	73	84	173	173	166	172	165	168	107	94	105	NA	92	84	190	182	19	17
B13A	80	80	83	165	165	167	85	85	84	61	66	66	73	77	67	171	171	169	170	168	172	110	105	103	97	91	105	185	189	16	21
B13B	80	76	94	151	160	NA	71	84	NA	66	72	NA	71	65	NA	166	165	NA	161	161	NA	100	93	NA	90	96	NA	182	177	20	16
B13C	83	87	80	167	NA	172	84	NA	92	79	72	79	71	73	NA	169	169	NA	170	165	NA	90	97	NA	99	92	NA	190	179	24	14
B13D	83	80	83	153	151	156	70	71	73	61	61	66	73	73	71	162	160	159	161	161	NA	101	99	93	88	88	NA	185	175	27	14
B13E	73	73	73	160	156	156	87	83	83	66	66	66	71	65	67	166	162	164	172	158	170	100	96	98	101	93	103	185	179	27	21
B13F	97	NA	97	NA	NA	NA	NA	NA	NA	NA	NA	NA	NA	NA	NA	NA	NA	NA	NA	NA	NA	NA	NA	NA	NA	NA	NA	NA	NA	NA	NA
B13G	76	83	80	153	165	151	77	82	71	61	61	61	71	71	71	176	176	173	170	165	170	115	115	112	99	94	99	190	182	16	17
B13H	90	97	NA	156	165	NA	66	68	NA	110	103	NA	NA	91	NA	NA	180	NA	NA	NA	NA	NA	77	NA	NA	NA	NA	196	NA	18	NA
B13I	80	87	NA	165	165	NA	85	78	NA	61	54	72	NA	73	77	169	173	165	163	163	168	108	119	93	NA	90	91	188	179	16	16
B13J	87	94	80	165	165	165	78	71	85	66	61	66	NA	71	71	164	165	165	163	163	161	98	104	99	NA	92	90	188	179	25	16
B13K	87	83	83	160	165	165	73	82	82	66	61	72	73	73	71	169	169	169	168	172	170	103	108	97	95	99	99	197	199	31	27
B14A	73	73	80	147	142	147	74	69	67	61	61	79	71	NA	NA	163	163	162	156	158	NA	102	102	83	85	NA	NA	186	177	25	19
B14B	76	76	80	172	165	172	96	89	92	61	61	61	65	65	71	173	176	171	170	172	172	112	115	110	105	107	101	188	193	15	21
B14C	83	73	83	165	151	165	82	78	82	61	72	61	73	77	73	171	169	173	165	161	163	110	97	112	92	84	90	185	177	19	16
B14D	76	83	80	156	165	165	80	82	85	61	61	61	71	65	65	176	169	173	168	165	163	115	108	112	97	100	98	184	179	19	14
B14E	87	83	83	165	160	160	78	77	77	66	72	66	67	77	67	164	162	164	163	165	163	98	90	98	96	88	96	190	184	32	19
B14F	87	87	97	156	165	165	69	78	68	72	72	82	73	84	80	162	164	164	163	165	163	90	92	82	90	81	83	184	184	22	19
B14G	118	83	101	NA	156	160	NA	73	59	NA	82	86	NA	98	84	NA	163	171	NA	NA	168	NA	81	85	NA	NA	84	184	NA	23	NA
B14H	76	76	NA	156	156	NA	80	80	NA	79	66	79	NA	73	71	162	162	158	156	158	156	83	96	79	NA	85	85	185	172	27	14
B14J	80	97	80	156	160	165	76	63	85	72	61	66	NA	80	71	162	162	163	158	161	161	90	101	97	NA	81	90	185	182	24	21
B14K	87	87	83	156	156	147	69	69	64	79	72	61	91	77	71	162	165	164	161	161	161	83	93	103	70	84	90	186	177	24	16
B15A	80	80	97	165	156	NA	85	76	NA	61	72	110	71	77	98	169	176	178	168	170	172	108	104	68	97	93	74	NA	193	NA	23
B15C	90	90	90	174	174	165	84	84	75	NA	106	110	108	NA	NA	NA	NA	176	NA	NA	172	NA	NA	66	NA	NA	NA	NA	NA	NA	NA
B15D	80	87	80	156	165	147	76	78	67	72	66	72	77	77	NA	173	171	169	163	163	163	101	105	97	86	86	NA	182	179	14	16
B15F	97	83	83	172	147	142	75	64	59	79	61	72	77	NA	NA	166	162	166	163	NA	161	87	101	94	86	NA	NA	185	177	24	16
B15G	87	76	80	156	156	160	69	80	80	66	61	79	71	71	71	173	165	169	161	161	161	107	104	90	90	90	90	184	177	22	16
B15H	80	76	73	138	138	142	58	62	69	61	66	61	67	NA	NA	171	165	164	161	163	161	110	99	103	94	NA	NA	185	177	22	14
B15I	80	80	83	156	156	153	76	76	70	66	61	86	NA	73	71	176	169	171	163	161	158	110	108	85	NA	88	87	185	177	19	16
B15J	87	83	83	160	167	160	73	84	77	72	72	72	NA	77	71	173	166	169	172	168	170	101	94	97	NA	91	99	188	184	24	16
B15K	80	76	80	147	151	138	67	75	58	72	66	66	73	NA	73	164	164	169	161	158	161	92	98	103	88	NA	88	185	175	25	17
B16A	76	76	80	167	165	165	91	89	85	66	72	66	73	67	71	169	166	169	165	158	165	103	94	103	92	91	94	182	172	17	14
B16B	83	83	80	151	165	151	68	82	71	72	61	66	77	77	71	178	163	171	168	158	161	106	102	105	91	81	90	185	177	24	19
B16C	73	76	80	156	151	160	83	75	80	66	61	82	71	67	71	176	173	169	163	177	161	110	112	87	92	110	90	186	196	15	19
B16D	83	83	87	151	151	160	68	68	73	NA	72	110	NA	73	NA	NA	158	169	NA	156	NA	NA	86	59	NA	83	NA	192	177	36	21
B16E	87	80	87	160	156	156	73	76	69	66	72	66	73	77	80	163	164	169	165	163	165	97	92	103	92	86	85	184	182	22	19
B16F	73	80	73	165	165	160	92	85	87	61	66	61	67	65	67	165	165	165	168	165	161	104	99	104	101	100	94	190	189	27	24
B16G	80	73	87	174	172	160	94	99	73	79	79	NA	77	71	NA	178	173	NA	175	168	NA	99	94	NA	98	97	NA	199	186	27	18
B16H	80	83	80	153	153	142	73	70	62	86	72	61	73	73	NA	163	162	165	170	163	161	77	90	104	97	90	NA	188	177	27	14
B16I	90	NA	NA	165	NA	NA	75	NA	NA	82	NA	NA	NA	73	NA	169	NA	NA	161	NA	NA	87	NA	NA	NA	NA	NA	NA	NA	NA	NA
B16J	73	73	80	160	160	153	87	87	73	61	79	66	NA	71	71	173	171	164	NA	168	172	112	92	98	NA	97	101	186	184	17	16
B17A	73	76	73	156	151	147	83	75	74	61	61	61	71	73	NA	169	164	169	161	163	161	108	103	108	90	90	NA	185	177	24	14
B17B	76	83	80	174	NA	167	98	NA	87	61	66	89	NA	NA	71	NA	NA	183	NA	NA	175	NA	NA	94	NA	NA	104	NA	NA	NA	NA
B17C	97	83	76	156	151	156	59	68	80	66	61	61	80	77	77	176	171	169	170	168	165	110	110	108	90	91	88	182	189	13	21
B17G	87	80	80	165	160	165	78	80	85	66	86	66	73	80	77	165	164	164	168	163	163	99	78	98	95	83	86	182	177	20	14
B17H	76	83	83	151	147	151	75	64	68	61	61	72	73	73	71	163	162	163	161	154	156	102	101	91	88	81	85	185	168	25	14
B17J	73	73	73	160	172	156	87	99	83	66	66	61	NA	71	65	178	166	169	172	170	168	112	100	108	NA	99	103	192	186	27	16
B17K	76	76	80	165	160	167	89	84	87	61	61	66	NA	71	73	176	176	183	172	163	168	115	115	117	NA	92	95	190	179	16	16
B18A	73	76	73	156	165	156	83	89	83	66	61	61	67	67	65	173	176	169	172	168	168	107	115	108	105	101	103	190	182	17	14
B18B	80	80	80	165	156	172	85	76	92	79	61	66	73	73	80	178	169	NA	168	168	172	99	108	NA	95	95	92	182	179	16	11
B18C	83	83	73	160	165	NA	77	82	NA	66	72	66	80	73	73	178	173	173	175	168	172	112	101	107	95	95	99	188	184	16	16
B18D	87	87	NA	165	165	NA	78	78	NA	72	79	NA	84	87	NA	164	163	NA	165	163	NA	92	84	NA	81	76	NA	185	182	23	19
B18E	80	76	83	156	153	160	76	77	77	66	61	61	67	71	71	171	176	173	168	170	175	105	115	112	101	99	104	188	184	15	14
B18F	90	76	94	167	160	165	77	84	71	66	72	103	73	73	80	169	166	176	170	163	170	103	94	73	97	90	90	188	182	24	19
B18G	73	73	80	160	165	160	87	92	80	61	61	66	71	71	73	162	160	163	165	156	165	101	99	97	94	85	92	186	177	27	21
B18H	80	73	73	156	165	160	76	92	87	61	61	66	NA	67	65	169	164	166	163	161	161	108	103	100	NA	94	96	185	184	23	23
B18I	73	80	80	160	165	165	87	85	85	72	61	61	NA	87	77	163	165	166	163	163	163	91	104	105	NA	76	86	185	182	22	19
B18J	97	90	87	160	167	167	63	77	80	79	89	79	NA	80	87	169	169	173	170	170	168	90	80	94	NA	90	81	188	184	22	14
B19A	73	76	73	156	160	151	83	84	78	66	79	61	67	71	77	176	171	176	175	NA	172	110	92	115	108	NA	95	190	NA	22	NA
B19B	87	NA	NA	172	NA	NA	85	NA	NA	79	NA	NA	NA	NA	NA	NA	NA	NA	NA	NA	NA	NA	NA	NA	NA	NA	NA	NA	NA	NA	NA
B19C	80	80	76	156	156	147	76	76	71	66	82	79	73	NA	NA	171	176	NA	168	NA	NA	105	94	NA	95	NA	NA	190	NA	15	NA
B19D	73	76	80	160	160	165	87	84	85	61	66	66	67	71	73	176	164	165	175	163	163	115	98	99	108	92	90	185	184	25	21
B19E	83	73	80	165	165	167	82	92	87	66	61	61	67	71	73	173	176	173	175	NA	170	107	115	112	108	NA	97	190	196	18	28
B19G	73	73	NA	165	160	NA	92	87	NA	61	61	82	71	NA	71	178	NA	171	168	NA	168	117	NA	89	97	NA	97	NA	NA	NA	NA
B19H	73	73	80	160	160	153	87	87	73	79	61	66	NA	71	71	165	164	166	168	165	165	86	103	100	NA	94	94	185	182	23	17
B19I	87	87	87	167	151	153	80	64	66	66	72	72	NA	80	80	169	169	169	168	163	163	103	97	97	NA	83	83	188	184	21	21
B19J	97	80	125	167	156	NA	70	76	NA	110	99	NA	101	NA	NA	NA	171	NA	NA	165	NA	NA	72	NA	NA	NA	NA	198	182	30	17
B19K	83	73	80	NA	174	172	NA	101	92	72	72	66	NA	71	71	180	176	183	175	172	175	108	104	117	NA	101	104	199	193	27	21
B1B	83	76	83	167	165	172	84	89	89	66	61	72	73	NA	71	178	173	171	172	175	175	112	112	99	99	NA	104	190	189	18	14
B1C	90	87	87	167	172	172	77	85	85	86	86	72	77	84	87	171	173	173	170	170	170	85	87	101	93	86	83	184	182	12	12
B1E	97	94	90	NA	172	172	NA	78	82	103	72	86	73	94	77	171	176	169	170	175	175	68	104	83	97	81	98	188	193	15	18
B1F	80	83	94	165	165	165	85	82	71	72	79	79	84	77	NA	169	171	164	163	165	163	97	92	85	79	88	NA	190	184	21	19
B1G	87	80	87	165	165	165	78	85	78	66	61	61	71	73	71	166	169	169	168	165	168	100	108	108	97	92	97	182	184	17	19

B1H		73	73	80	142	142	147	69	69	67	61	61	61	67	NA	NA	163	164	166	161	156	161	102	103	105	94	NA	NA	185	170	25	14
B1J		83	73	73	167	160	160	84	87	87	61	61	72	NA	71	71	171	169	169	163	165	170	110	108	97	NA	94	99	NA	179	NA	14
B1K		87	73	73	167	156	151	80	83	78	79	61	66	NA	73	67	183	176	173	177	170	165	104	115	107	NA	97	98	182	184	9	14
B20A		87	83	87	165	165	160	78	82	73	66	61	72	77	87	77	169	169	169	170	163	168	103	108	97	93	76	91	188	184	22	21
B20B		94	80	104	160	165	167	66	85	63	NA	NA	79	NA	NA	80	NA	NA	165	NA	NA	161	NA	NA	86	NA	NA	81	NA	NA	NA	NA
B20C		83	76	80	156	174	165	73	98	85	66	72	79	77	84	94	171	176	173	175	182	168	105	104	94	98	98	74	199	NA	26	NA
B20D		83	83	83	165	167	172	82	84	89	72	66	66	71	73	77	169	171	171	172	168	168	97	105	105	101	95	91	195	184	28	16
B20E		87	83	NA	172	165	NA	85	82	NA	66	72	NA	71	77	NA	171	171	NA	175	168	NA	105	99	NA	104	91	NA	NA	193	NA	25
B20F		90	76	80	156	153	147	66	77	67	66	61	61	77	73	NA	165	159	165	175	163	158	99	98	104	98	90	NA	184	177	25	14
B20G		87	80	80	156	151	165	69	71	85	61	61	86	73	73	77	171	164	169	163	165	NA	110	103	83	90	92	NA	185	179	23	14
B20H		90	90	125	160	160	NA	70	70	NA	NA	86	86	105	NA	77	166	171	169	NA	168	168	NA	85	83	NA	NA	91	188	193	19	25
B20I		83	80	80	156	151	160	73	71	80	72	72	82	NA	73	NA	166	163	NA	168	156	NA	94	91	NA	NA	83	NA	182	175	21	19
B20J		87	83	87	160	156	153	73	73	66	66	72	66	NA	73	80	162	163	163	154	156	154	96	91	97	NA	83	74	186	172	27	16
B20K		90	83	87	160	165	165	70	82	78	79	66	72	NA	73	77	169	164	164	161	163	165	90	98	92	NA	90	88	184	184	24	21
B21A		80	NA	76	147	NA	165	67	NA	89	79	NA	66	71	NA	71	171	NA	166	158	NA	168	92	NA	100	87	NA	97	NA	NA	NA	NA
B21B		73	73	73	165	156	160	92	83	87	66	54	61	67	71	67	166	169	166	163	163	161	100	115	105	96	92	94	184	179	18	16
B21C		83	94	97	167	NA	172	84	NA	75	66	NA	66	73	NA	80	171	NA	171	168	NA	168	105	NA	105	95	NA	88	NA	NA	NA	NA
B21E		76	76	83	151	160	156	75	84	73	72	61	86	73	73	67	163	165	183	163	163	158	91	104	97	90	90	91	185	182	23	19
B21G		76	76	73	156	160	160	80	84	87	61	61	79	71	73	71	165	165	169	175	165	161	104	104	90	104	92	90	186	179	22	14
B21H		83	87	83	156	160	153	73	73	70	66	79	72	NA	77	77	165	166	169	161	161	161	99	87	97	NA	84	84	186	177	21	16
B21I		83	83	90	156	156	156	73	73	66	79	61	72	NA	80	84	166	163	163	161	158	158	87	102	91	NA	78	74	188	182	27	24
B21J		87	87	83	160	160	156	73	73	73	66	61	66	NA	71	77	163	160	165	161	161	161	97	99	99	NA	90	84	188	177	31	16
B21K		83	80	83	156	156	160	73	76	77	72	72	66	NA	71	84	162	165	169	163	165	161	90	93	103	NA	94	77	188	179	26	14
B22A		97	94	83	167	153	160	70	59	77	79	82	72	84	84	84	164	164	164	NA	163	161	85	82	92	NA	79	77	188	179	28	16
B22B		90	83	87	167	165	165	77	82	78	61	61	66	80	77	80	163	169	165	161	161	158	102	108	99	81	84	78	188	182	22	21
B22C		97	90	87	172	172	174	75	82	87	61	72	66	65	77	77	180	171	178	177	175	175	119	99	112	112	98	98	192	199	24	24
B22E		87	83	87	165	165	165	78	82	78	66	66	72	73	73	77	165	165	169	168	163	168	99	99	97	95	90	91	184	182	22	19
B22F		87	90	87	NA	167	167	NA	77	80	NA	66	82	NA	77	77	NA	169	169	NA	168	168	NA	103	87	NA	91	91	190	193	24	25
B22G		76	73	NA	172	167	NA	96	94	NA	61	61	NA	NA	71	NA	173	173	NA	172	168	NA	112	112	NA	NA	97	NA	182	182	10	14
B22H		73	83	80	172	165	167	99	82	87	61	61	61	NA	71	77	169	171	171	168	168	163	108	110	110	NA	97	86	197	182	28	14
B22I		80	76	80	165	153	160	85	77	80	61	61	72	NA	77	NA	169	162	NA	168	163	NA	108	101	NA	NA	86	NA	185	184	27	21
B22J		87	83	83	142	156	142	55	73	59	66	61	61	77	NA	NA	162	164	162	161	154	156	96	103	101	84	NA	NA	179	172	17	18
B22K		90	NA	83	NA	NA	NA	NA	NA	NA	NA	82	NA	NA	NA	71	NA	165	NA	NA	158	NA	NA	83	NA	NA	NA	NA	182	177	19	19
B23A		97	83	80	156	138	138	59	55	58	99	79	NA	98	NA	NA	160	169	NA	NA	154	NA	61	90	NA	NA	NA	NA	186	172	20	18
B23B		73	76	73	160	165	167	87	89	94	66	61	61	65	84	67	166	169	169	165	165	168	100	108	108	100	81	101	190	186	24	21
B23C		76	76	73	151	151	NA	75	75	NA	61	66	61	65	67	NA	169	164	166	165	168	163	108	98	105	100	101	NA	184	184	24	16
B23D		87	87	80	174	174	165	87	87	85	NA	79	72	NA	NA	73	NA	183	169	NA	177	170	NA	104	97	NA	NA	97	199	196	18	19
B23E		83	73	73	165	165	167	82	92	94	66	61	66	73	71	73	171	171	176	172	170	175	105	110	110	99	99	102	190	186	22	16
B23F		97	83	94	174	NA	NA	77	NA	NA	79	NA	NA	80	NA	NA	162	NA	NA	156	NA	NA	83	NA	NA	76	NA	NA	NA	NA	NA	NA
B23G		83	73	73	165	167	165	82	94	92	66	61	79	NA	65	71	166	166	164	168	NA	163	100	105	85	NA	NA	92	190	184	25	19
B23H		73	80	94	147	156	153	74	76	59	66	61	72	73	73	73	165	166	176	170	163	168	99	105	104	97	90	95	188	177	24	14
B23I		73	76	NA	165	156	NA	92	80	NA	61	66	NA	NA	71	NA	173	171	NA	175	168	NA	112	105	NA	NA	97	NA	NA	189	NA	21
B23K		83	80	83	160	153	160	77	73	77	79	61	61	73	71	71	165	164	166	163	163	161	86	103	105	90	92	90	185	179	24	16
B24A		73	80	76	156	165	160	83	85	84	61	66	61	65	67	65	164	164	162	161	161	158	103	98	101	96	94	93	188	179	26	18
B24B		76	76	80	156	156	153	80	80	73	66	66	61	65	77	80	160	160	162	161	161	161	94	94	101	96	84	81	185	179	26	18
B24C		80	83	76	147	153	NA	67	70	NA	66	79	NA	71	NA	NA	165	NA	NA	158	NA	NA	99	NA	NA	87	NA	NA	NA	NA	NA	NA
B24D		83	76	80	160	156	156	77	80	76	66	61	79	80	80	80	164	164	176	168	161	163	98	103	97	88	81	83	184	177	23	16
B24E		73	76	73	138	147	142	65	71	69	61	72	61	71	NA	NA	169	171	173	168	168	168	108	99	112	97	NA	NA	184	184	15	16
B24F		80	83	80	147	151	153	67	68	73	66	79	82	71	71	80	158	162	164	156	158	163	92	83	82	85	87	83	185	168	25	10
B24H		73	76	80	165	165	165	92	89	85	61	61	61	NA	71	65	166	169	169	163	163	172	105	108	108	NA	92	107	188	179	22	16
B24I		83	76	80	167	167	165	84	91	85	72	61	66	NA	73	80	169	166	166	161	163	165	97	105	100	NA	90	85	186	179	21	16
B24J		80	76	73	165	160	160	85	84	87	66	66	66	NA	71	67	166	165	169	163	165	168	100	99	103	NA	94	101	190	193	28	28
B24K		83	87	73	142	153	142	59	66	69	72	72	66	77	NA	77	171	176	169	168	158	161	99	104	103	91	NA	84	188	177	14	19
B25A		80	76	83	160	165	156	80	89	73	66	61	66	73	71	71	166	164	164	170	163	163	100	103	98	97	92	92	188	182	25	19
B25B		97	104	NA	174	NA	NA	77	NA	NA	110	NA	NA	98	NA	NA	176	NA	NA	168	NA	NA	66	NA	NA	70	NA	NA	NA	NA	NA	NA
B25C		90	83	NA	156	174	NA	66	91	NA	79	86	NA	73	98	NA	171	173	NA	161	161	NA	92	87	NA	88	63	NA	188	182	16	21
B25D		80	83	76	160	153	151	80	70	75	61	66	79	67	77	67	169	166	169	172	165	163	108	100	90	105	88	96	182	179	17	14
B25E		73	73	73	147	151	151	74	78	78	61	54	61	67	71	65	163	160	164	158	158	158	102	106	103	91	87	93	185	175	26	17
B25F		83	83	NA	147	147	NA	64	64	NA	61	66	NA	73	NA	NA	169	169	NA	158	163	NA	108	103	NA	85	NA	NA	190	182	25	19
B25G		76	76	73	165	172	165	89	96	92	61	61	61	NA	73	77	171	169	169	170	168	165	110	108	108	NA	95	88	190	184	24	16
B25H		76	76	80	160	160	160	84	84	80	61	61	61	NA	73	71	169	169	169	168	168	168	108	108	108	NA	95	97	188	182	22	14
B25I		83	76	80	165	165	167	82	89	87	79	61	66	NA	73	67	173	171	169	170	170	170	94	110	103	NA	97	103	199	196	31	26
B25J		76	80	73	156	160	156	80	80	83	79	82	61	NA	71	67	163	164	169	168	163	168	84	82	108	NA	92	101	185	179	23	16
B25K		83	87	83	142	151	142	59	64	59	79	66	79	80	NA	84	169	164	166	161	161	156	90	98	87	81	NA	72	185	175	22	14
B26A		83	83	80	172	172	172	89	89	92	61	66	61	77	77	67	173	171	176	175	170	170	112	105	115	98	93	103	190	184	20	14
B26B		87	NA	NA	NA	NA	NA	NA	NA	NA	86	NA	NA	87	NA	NA	165	NA	NA	165	NA	NA	79	NA	NA	78	NA	NA	NA	NA	NA	NA
B26C		80	76	73	165	167	165	85	91	92	61	61	66	71	67	73	166	169	169	165	168	168	105	108	103	94	101	95	190	189	24	21
B26D		90	80	87	142	142	153	52	62	66	61	66	106	73	NA	NA	162	165	NA	161	161	NA	101	99	NA	88	NA	NA	185	179	24	18
B26E		80	73	73	147	153	142	67	80	69	61	61	61	71	71	NA	176	NA	176	172	170	168	115	NA	115	101	99	NA	192	184	NA	14
B26F	NA	NA	NA	NA	NA	NA	NA	NA	NA	NA	NA	NA	NA	NA	NA	NA	NA	NA	NA	NA	NA	NA	NA	NA	NA	NA	NA	NA	NA	NA	NA	
B26G		80	80	80	160	156	151	80	76	71	66	61	72	NA	65	71	164	165	163	161	156	161	98	104	91	NA	91	90	185	172	22	16
B26H		73	80	76	160	160	160	87	80	84	61	61	61	NA	71	71	165	164	164	161	161	161	104	103	103	NA	90	90	190	179	28	18
B26I		87	83	94	165	167	172	78	84	78	NA	72	82	NA	NA	NA	NA	NA	171	NA	NA	NA	NA	NA	89	NA	NA	NA	NA	NA	NA	NA
B26J		90	87	94	172	165	172	82	78	78	79	86	72	NA	84	84	169	165	160	163	163	163	90	79	88	NA	79	79	186	182	22	19
B26K		87	83	80	NA	165	165	NA	82	85	82	79	86	NA	NA	84	176	176	NA	NA	NA	NA	94	97	NA	NA	NA	NA	184	NA	11	NA
B27A		73	76	76	153	160	153	80	84	77	61	61	61	65	71	71	166	169	169	161	161	168	105	108	108	96	90	97	184	177	18	16
B27B		73	73	80	147	142	142	74	69	62	72	61	72	65	NA	NA	165	162	158	156	158	156	93	101	86	91	NA	NA	185	175	27	17
B27C		80	NA	73	NA	NA	NA	NA	NA	NA	NA	NA	NA	NA	NA	NA	NA	NA	NA	NA	NA	NA	NA	NA	NA	NA	NA	NA	NA	NA	NA	NA
B27D		90	76	80	156	142	142	66	66	62	79	61	82	73	NA	NA	169	164	163	156	154	161	90	103	81	83	NA	NA	185	168	25	14
B27E		83	80	80	156	160	160	73	80	80	72	61	61	71	77	NA	165	160	164	161	161	163	93	99	103	90	84	NA	182	184	25	23
B27G		87	76	83	172	172	172	85	96	89	66	72	72	NA	65	71	173	171	169	168	168	170	107	99	97	NA	103	99	190	193	22	25
B27H		87	80	80	151	151	151	64	71	71	79	79	82	NA	71	71	163	160	186	156	156	154	84	81	104	NA	85	83	182	172	23	16
B27I		73	76	73	147	153	142	74	77	69	61	61	61	65	65	NA	162	162	166	163	156	161	101	101	105	98	91	NA	185	170	24	14
B27J		87	83	80	165	160	160	78	77	80	79	66	66	105	NA	80	NA	171	171	NA	163	168	NA	105	105	NA	NA	88	188	179	19	16
B27K		83	76	76	147	138	138	64	62	62	79	61	72	71	NA	NA	160	159	159	161	158	158	81	98	87	90	NA	NA	185	172	27	14
B28A		83	87	83	165	165	165	82	78	82	66	72	66	71	71	77	169	165	165	170	165	170	103	93	99	99	94	93	190	184	27	19
B28B		97	94	NA	NA	172	NA	NA	78	NA	NA	106	NA	NA	94	NA	NA	190	173	NA	NA	NA	NA	84	NA	NA	NA	NA	NA	NA	NA	NA
B28D		90	94	94	NA	174	NA	NA	80	NA	NA	79	92	98	98	NA	178	183	183	177	179	179	NA	104	91	79	81	NA	197	196	16	17
B28E		83	80	83	153	151	153	70	71	70	72	79	66	73	77	77	160	163	160	156	158	161	88	84	94	83	81	84	185	177	26	19
B28F		90	83	101	174	167	NA	84	84	NA	61	61	66	80	73	NA	171	169	NA	163	165	NA	110	108	NA	83	92	NA	182	189	17	24
B28G		80	80	NA	165	165	NA	85	85	NA	66	66	NA	NA	80	NA	166	166	NA	163	163	NA	100	100	NA	NA	83	NA	186	182	21	19
B28H		80	94	83	165	165	165	85	71	82	72	61	61	NA	73	71	165	164	166	163	165	163	93	103	105	NA	92	92	185	184	22	19
B28I		80	76	80	153	153	153	73	77	73	66	61	66	101	73	NA	163	190	186	161	161	170	97	129	120	60	88	NA	NA	177	NA	16
B28K		87	90	90	165	165	165	78	75	75	79	79	79	NA	80	77	169	166	169	168	170	170	90	87	90	NA	90	93	188	193	23	23
B29A		73	80	80	165	172	165	92	92	85	61	61	72	71	77	71	169	178	169	172	175	161	108	117	97	101	98	90	197	196	21	21
B29B		80	83	80	156	156	165	76	73	85	79	61	72	77	71	73	165	164	169	168	165	165	86	103	97	91	94	92	185	179	22	14
B29C		83	83	80	151	165	165	68	82	85	72	61	79	73	67	77	159	164	164	156	161	161	87	103	85	83	94	84	188	177	28	16
B29F		83	83	83	156	160	165	73	77	82	72	79	72	71	84	84	171	166	169	170	168	168	99	87	97	99	84	84	188	189	23	21
B29G		90	83	94	165	167	165	75	84	71	79	86	86	NA	73	80	160	166	165	161	165	163	81	80	79	NA	92	83	186	184	21	19
B29H		83	83	80	165	153	165	82	70	85	61	54	66	NA	67	73	166	163	171	163	158	168	105	109	105	NA	91	95	184	172	25	14
B29J		87	80	80	147	151	142	60	71	62	79	72	79	71	NA	77	166	171	169	163	NA	161	87	99	90	92	NA	84	184	172	16	14
B29K		83	83	97	151	165	165	68	82	68	66	61	72	NA	77	77	162	165	164	168	163	161	96	104	92	NA	86	84	186	179	23	16
B2A		80	76	80	167	151	142	87	75	62	86	61	66	73	71	NA	180	165	173	165	161	156	94	104	107	92	90	NA	182	177	19	16
B2B		80	80	83	156	NA	153	76	NA	70	72	66	61	65	84	71	169	169	164	161	163	163	97	103	103	96	79	92	188	175	21	12

B2C	87	83	NA	160	160	NA	73	77	NA	61	61	NA	65	73	NA	163	164	NA	168	163	NA	102	103	NA	103	90	NA	188	182	26	19
B2D	90	NA	NA	165	NA	NA	75	NA	NA	79	NA	NA	73	NA	NA	169	NA	NA	161	NA	NA	90	NA	NA	88	NA	NA	NA	NA	NA	NA
B2E	87	80	80	167	147	174	80	67	94	89	72	72	77	NA	80	NA	165	176	NA	161	161	NA	93	104	NA	NA	81	188	177	24	16
B2F	83	83	NA	174	174	NA	91	91	NA	103	NA	NA	73	NA	NA	163	NA	NA	161	NA	NA	60	NA	NA	88	NA	NA	NA	NA	NA	NA
B2G	87	83	83	172	172	165	85	89	82	72	72	72	77	77	77	171	176	173	172	168	172	99	104	101	95	91	95	197	182	24	14
B2I	87	83	83	172	165	172	85	82	89	79	61	72	98	73	73	171	169	173	170	168	170	92	108	101	72	95	97	188	196	21	28
B2J	83	87	83	167	167	165	84	80	82	79	72	61	NA	71	80	166	165	169	168	168	175	87	93	108	NA	97	95	190	189	26	21
B2K	87	87	87	156	151	NA	69	64	NA	79	66	79	NA	87	84	165	160	NA	161	163	161	86	94	NA	NA	76	77	184	179	25	16
B30B	73	83	80	165	172	174	92	89	94	61	61	NA	65	80	NA	171	171	NA	168	NA	NA	110	110	NA	103	NA	NA	190	NA	22	NA
B30C	94	NA	NA	165	NA	NA	71	NA	NA	86	106	NA	91	84	NA	166	164	NA	161	156	NA	80	58	NA	70	72	NA	188	175	25	19
B30D	80	80	80	151	151	151	71	71	71	66	61	79	73	71	73	162	158	165	154	154	156	96	97	86	81	83	83	185	170	29	16
B30E	76	80	80	142	138	156	66	58	76	66	66	79	80	NA	84	176	169	171	163	163	168	110	103	92	83	NA	84	188	179	23	16
B30F	80	80	80	165	156	165	85	76	85	66	61	61	71	71	65	169	169	169	NA	172	161	103	108	108	NA	101	96	190	193	24	21
B30G	76	76	94	165	165	165	89	89	71	NA	61	66	NA	71	73	NA	180	164	NA	170	179	NA	119	98	NA	99	106	199	189	22	19
B30H	83	83	87	156	151	151	73	68	64	79	61	61	NA	73	71	160	162	160	156	156	158	81	101	99	NA	83	87	185	172	27	16
B30I	87	83	80	165	160	165	78	77	85	66	61	66	NA	71	73	171	169	165	168	165	161	105	108	99	NA	94	88	188	182	23	17
B30J	80	76	80	142	142	151	62	66	71	66	61	61	73	NA	71	173	173	171	163	163	161	107	112	110	90	NA	90	188	179	18	16
B30K	80	76	83	147	151	138	67	75	55	61	61	66	73	NA	67	178	171	176	161	156	154	117	110	110	88	NA	87	190	168	21	12
B31A	94	94	87	172	160	153	78	66	66	NA	86	79	NA	84	80	NA	162	173	NA	161	158	NA	76	94	NA	77	78	182	177	22	16
B31B	80	76	83	156	174	160	76	98	77	66	61	61	65	71	71	173	171	173	170	168	170	107	110	112	105	97	99	190	179	20	11
B31C	87	87	NA	174	174	NA	87	87	NA	NA	66	NA	NA	80	NA	NA	165	NA	NA	168	NA	NA	99	NA	NA	88	NA	188	184	24	16
B31D	87	NA	NA	172	NA	NA	85	NA	NA	72	NA	NA	77	NA	NA	176	NA	NA	172	NA	NA	104	NA	NA	95	NA	NA	NA	NA	NA	NA
B31E	87	83	83	151	147	153	64	64	70	79	61	72	71	NA	73	158	158	159	156	156	161	79	97	87	85	NA	88	185	175	29	19
B31F	NA	NA	NA	NA	NA	NA	NA	NA	NA	NA	NA	NA	NA	NA	NA	NA	NA	NA	NA	NA	NA	NA	NA	NA	NA	NA	NA	NA	NA	NA	NA
B31G	80	80	76	NA	142	151	NA	62	75	66	61	61	71	NA	71	163	160	164	163	158	165	97	99	103	92	NA	94	185	177	27	19
B31H	83	87	90	167	165	167	84	78	77	72	79	79	NA	87	80	163	165	169	168	172	170	91	86	90	NA	85	90	NA	193	NA	21
B31I	87	73	87	156	165	153	69	92	66	66	72	79	NA	77	77	165	163	165	165	161	158	99	91	86	NA	84	81	185	177	24	16
B31J	83	80	80	160	160	160	77	80	80	66	79	82	NA	80	91	166	160	163	161	161	161	100	81	81	NA	81	70	184	179	25	18
B31K	76	83	76	153	160	160	77	77	84	66	66	72	NA	73	80	166	169	169	165	163	163	100	103	97	NA	90	83	190	184	25	21
B32A	97	83	83	NA	142	142	NA	59	59	NA	61	86	80	NA	NA	NA	166	169	NA	163	161	NA	105	83	NA	NA	NA	189	182	24	19
B32B	83	80	90	167	165	172	84	85	82	66	61	66	71	71	73	169	171	173	172	168	172	103	110	107	101	97	99	190	186	22	18
B32C	83	90	83	153	NA	165	70	NA	82	66	NA	79	73	NA	80	171	NA	176	163	NA	161	105	NA	97	90	NA	81	NA	NA	NA	NA
B32D	83	87	80	172	167	165	89	80	85	66	61	66	77	71	77	169	171	169	163	163	163	103	110	103	86	92	86	190	177	21	14
B32E	73	73	80	147	153	138	74	80	58	79	66	72	77	71	NA	165	162	169	154	154	158	86	96	97	77	83	NA	182	168	22	14
B32F	76	76	NA	160	160	NA	84	84	NA	NA	92	NA	NA	NA	NA	NA	NA	NA	NA	NA	NA	NA	NA	NA	NA	NA	NA	NA	NA	NA	NA
B32G	87	83	90	153	142	156	66	59	66	79	66	79	84	73	87	166	162	176	161	161	168	87	96	97	77	88	81	179	177	21	16
B32H	83	76	80	165	165	156	82	89	76	66	61	61	NA	65	73	165	164	166	163	163	161	99	103	105	NA	98	88	191	182	29	19
B32I	73	80	73	153	156	160	80	76	87	61	61	72	NA	71	71	166	162	160	168	163	163	105	101	88	NA	92	92	188	184	27	21
B32J	90	80	73	165	160	160	75	80	87	61	66	61	NA	65	71	166	165	169	168	163	165	105	99	108	NA	98	94	182	182	20	19
B32K	83	76	83	165	165	165	82	89	82	72	61	82	87	87	84	169	173	171	172	168	168	97	112	89	85	81	84	190	186	18	18
B33B	87	83	87	165	165	167	78	82	80	66	72	79	71	71	71	173	171	169	172	163	163	107	99	90	101	92	92	188	184	20	21
B33C	80	73	73	165	172	165	85	99	92	61	61	61	65	67	65	171	176	173	168	170	170	110	115	112	103	103	105	190	196	18	26
B33D	87	90	94	165	167	165	78	77	71	66	72	82	71	84	77	164	166	166	168	168	165	98	94	84	97	84	88	190	189	25	21
B33E	87	76	80	165	156	160	78	80	80	72	66	66	73	73	77	165	169	169	163	158	161	93	103	103	90	85	84	188	172	21	14
B33F	90	87	94	165	160	165	75	73	71	66	86	86	84	84	NA	163	165	NA	NA	161	NA	97	79	NA	NA	77	NA	188	177	25	16
B33G	80	83	NA	156	153	NA	76	70	NA	66	61	NA	NA	71	NA	163	160	NA	165	158	NA	97	99	NA	NA	87	NA	185	177	26	19
B33H	83	83	80	165	165	160	82	82	80	61	72	61	NA	71	71	163	165	164	168	163	165	102	93	103	NA	92	94	188	179	25	16
B33I	73	80	80	147	142	142	74	62	62	61	61	66	67	67	NA	158	158	158	161	154	154	97	97	92	94	87	NA	186	172	30	18
B33J	108	87	87	174	165	NA	66	78	NA	NA	89	NA	NA	NA	84	NA	183	NA	NA	163	NA	NA	94	NA	NA	NA	NA	NA	182	NA	19
B33K	83	NA	NA	165	NA	NA	82	NA	NA	82	NA	NA	NA	80	NA	178	NA	NA	NA	NA	NA	96	NA	NA	NA	NA	NA	NA	NA	NA	NA
B34A	73	NA	NA	138	NA	NA	65	NA	NA	54	NA	NA	65	NA	NA	165	NA	NA	165	NA	NA	111	NA	NA	100	NA	NA	NA	NA	NA	NA
B34B	80	83	NA	160	153	NA	80	70	NA	66	61	NA	65	71	NA	166	169	NA	165	163	NA	100	108	NA	100	92	NA	184	182	18	19
B34C	90	87	94	165	167	165	75	80	71	79	61	82	73	65	77	166	169	176	163	168	170	87	108	94	90	103	93	182	184	16	16
B34D	76	83	80	156	151	156	80	68	76	61	61	61	73	73	71	162	NA	163	163	158	161	101	NA	102	90	85	90	185	175	24	17
B34E	80	94	80	147	142	153	67	48	73	66	61	82	71	NA	84	160	162	169	163	158	158	94	101	87	92	NA	74	185	175	26	17
B34F	87	76	80	165	160	165	78	84	85	82	66	82	94	84	80	166	NA	166	177	165	165	84	NA	84	83	81	85	182	179	NA	14
B34H	87	80	73	165	153	172	78	73	99	79	66	66	77	71	84	169	160	NA	170	163	NA	90	94	NA	93	92	NA	190	179	31	16
B34I	108	83	83	156	165	167	48	82	84	NA	79	72	101	71	87	NA	180	171	NA	NA	170	NA	101	99	NA	NA	83	190	193	13	25
B34J	94	83	76	160	156	147	66	73	71	79	66	72	80	71	80	160	162	165	161	170	165	81	96	93	81	99	85	182	189	21	19
B3A	73	73	80	142	151	151	69	78	71	79	61	72	67	67	71	173	169	173	158	156	161	94	108	101	91	89	90	188	175	21	19
B3B	73	90	NA	156	165	NA	83	75	NA	79	79	72	84	77	84	166	163	162	158	156	158	87	84	90	74	79	74	188	172	27	16
B3C	NA	NA	NA	NA	NA	NA	NA	NA	NA	NA	NA	61	NA	NA	71	NA	NA	169	NA	NA	165	NA	NA	108	NA	NA	94	NA	NA	NA	NA
B3D	83	80	87	156	153	151	73	73	64	86	61	66	77	71	77	171	163	171	163	158	163	85	102	105	86	87	86	182	177	21	19
B3E	87	80	83	165	165	165	78	85	82	86	66	66	73	71	73	169	165	165	163	163	168	83	99	99	90	92	95	188	184	25	21
B3F	73	80	76	174	172	172	101	92	96	66	61	61	71	71	71	176	173	178	172	172	177	110	112	117	101	101	106	190	193	18	21
B3G	87	83	80	165	165	167	78	82	87	61	61	66	77	73	73	169	169	171	170	168	170	108	108	105	93	95	97	184	182	18	14
B3H	87	87	87	167	172	172	80	85	85	79	66	66	NA	84	77	171	169	171	168	170	168	92	103	105	NA	86	91	190	189	23	19
B3I	83	80	80	160	160	160	77	80	80	66	61	86	NA	73	77	163	164	164	161	161	161	97	103	78	NA	88	84	NA	177	NA	16
B3K	87	NA	NA	172	NA	NA	85	NA	NA	82	NA	NA	NA	NA	NA	199	NA	NA	NA	NA	NA	117	NA	NA	NA	NA	NA	NA	NA	NA	NA
B4B	80	87	101	165	165	NA	85	78	NA	79	NA	72	73	NA	73	173	NA	NA	165	NA	170	94	NA	NA	92	NA	97	NA	NA	NA	NA
B4C	83	76	80	138	142	138	55	66	58	79	72	66	71	NA	NA	171	173	171	154	154	158	92	101	105	83	NA	NA	188	168	16	14
B4D	90	83	90	165	147	151	75	64	61	NA	61	NA	73	NA	NA	NA	166	NA	NA	168	NA	NA	105	NA	NA	NA	NA	182	NA	17	NA
B4E	80	73	73	174	174	167	94	101	94	61	61	61	71	71	65	176	176	176	172	172	172	115	115	115	101	101	107	197	193	23	21
B4F	87	83	NA	174	151	NA	87	68	NA	66	NA	NA	NA	NA	NA	NA	NA	NA	NA	NA	NA	NA	NA	NA	NA	NA	NA	NA	NA	NA	NA
B4G	90	80	73	160	151	151	70	71	78	72	61	61	71	71	71	169	165	166	156	158	NA	97	104	105	85	87	NA	185	172	24	14
B4H	87	87	87	NA	172	160	NA	85	73	82	66	72	NA	91	71	173	178	176	170	177	175	91	112	104	NA	86	104	188	193	11	16
B4J	87	83	NA	NA	172	NA	NA	89	NA	NA	NA	NA	NA	NA	NA	NA	NA	NA	NA	NA	NA	NA	NA	NA	NA	NA	NA	NA	NA	NA	NA
B4K	80	76	80	165	160	167	85	84	87	79	61	66	NA	NA	73	169	162	169	NA	163	161	90	101	103	NA	NA	88	185	175	25	12
B5A	83	94	80	167	167	165	84	73	85	79	79	82	77	80	80	171	178	171	172	168	168	92	99	89	95	88	88	185	184	9	16
B5B	80	80	NA	165	160	NA	85	80	NA	79	86	NA	71	73	NA	160	162	NA	161	158	NA	81	76	NA	90	85	NA	185	175	27	17
B5C	83	80	101	151	153	NA	68	73	NA	61	79	NA	71	73	NA	166	171	NA	158	156	NA	105	92	NA	87	83	NA	190	177	20	21
B5D	80	76	80	147	138	NA	67	62	NA	61	NA	NA	71	NA	NA	169	NA	169	154	NA	156	108	NA	NA	83	NA	NA	NA	NA	NA	NA
B5E	73	73	73	151	142	142	78	69	69	61	61	79	67	NA	NA	169	166	169	165	161	170	108	105	90	98	NA	NA	188	177	24	16
B5F	80	83	83	165	160	156	85	77	73	66	66	79	71	77	77	162	158	162	156	154	163	96	92	83	85	77	86	184	175	28	21
B5H	87	83	87	151	142	142	64	59	55	86	61	72	80	87	NA	164	159	160	172	161	154	78	98	88	92	74	NA	182	177	25	16
B5I	80	80	83	165	156	160	85	76	77	79	61	72	NA	73	77	163	160	162	168	161	172	84	99	90	NA	88	95	185	177	26	16
B5J	97	73	73	172	165	165	75	92	92	NA	66	72	71	NA	80	NA	171	169	NA	163	170	NA	105	97	NA	NA	90	188	177	20	14
B5K	87	83	80	172	165	165	85	82	85	79	61	72	NA	NA	77	183	171	169	NA	163	170	104	110	97	NA	NA	93	188	182	21	19
B6A	73	76	80	156	151	151	83	75	71	61	61	61	71	73	71	169	169	162	161	161	161	108	108	101	90	88	90	190	177	24	16
B6B	80	73	80	153	142	151	73	69	71	61	61	66	71	NA	71	166	162	166	163	161	163	105	101	100	92	NA	92	184	177	24	16
B6F	80	83	80	167	165	165	87	82	85	79	66	79	77	77	80	173	171	171	170	165	168	94	105	92	93	88	88	199	182	31	17
B6G	87	NA	NA	167	NA	NA	80	NA	NA	79	NA	NA	NA	NA	NA	165	NA	NA	NA	NA	NA	86	NA	NA	NA	NA	NA	NA	NA	NA	NA
B6H	80	76	76	160	165	151	80	89	75	61	61	61	NA	71	71	169	169	169	165	168	165	108	108	108	NA	97	94	192	184	26	16
B6J	83	83	NA	160	153	NA	77	70	NA	79	72	NA	NA	73	NA	163	162	NA	161	158	NA	84	90	NA	NA	85	NA	185	172	27	14
B6K	80	80	83	165	160	160	85	80	77	NA	61	66	NA	NA	73	NA	164	160	NA	161	163	NA	103	94	NA	NA	90	185	177	23	16
B7I	83	83	80	165	165	167	82	82	87	72	72	66	NA	73	77	166	169	169	168	168	168	94	97	103	NA	95	91	NA	189	NA	21
B7C	83	83	80	151	172	156	68	89	76	66	61	61	77	80	77	176	178	176	163	168	168	110	117	115	86	88	91	193	179	16	11
B7D	80	NA	83	165	NA	160	85	NA	77	66	NA	72	71	NA	73	173	NA	173	168	NA	170	107	NA	101	97	NA	97	NA	NA	NA	NA
B7E	76	76	73	165	147	156	89	71	83	66	61	61	71	NA	71	169	171	173	168	163	168	103	110	112	97	NA	97	198	179	30	16
B7F	83	83	80	172	167	165	89	84	85	66	72	72	NA	94	84	169	169	169	NA	170	163	103	97	97	NA	76	79	199	186	33	16
B7G	87	83	83	151	147	147	64	64	64	72	66	72	73	NA	NA	160	159	162	156	154	154	88	93	90	83	NA	NA	182	168	23	14
B7H	97	83	NA	NA	151	NA	NA	68	NA	NA	66	NA	98	NA	NA	NA	169	169	NA	NA	161	NA	103	NA	NA	NA	NA	190	NA	23	NA
B7J	83	76	76	160	160	156	77	84	80	72	66	72	NA	73	80	166	164	163	158	165	163	94	98	91	NA	92	83	182	179	20	14
B8A	83	83	76	156	151	172	73	68	96	79	61	79	77	80	77	171	173	173	170	163	158	92	112	94	93	83	81	186	177	14	14

B8B	83	80	83	165	160	165	82	80	82	66	61	72	73	71	73	169	166	169	168	161	165	103	105	97	95	90	92	192	182	28	21
B8C	80	76	80	165	160	165	85	84	85	61	61	61	77	67	73	166	166	169	163	163	163	105	105	108	86	96	90	185	175	20	12
B8E	90	76	87	160	156	160	70	80	73	66	66	72	77	84	71	164	164	164	161	161	161	98	98	92	84	77	90	186	177	24	16
B8F	73	80	80	172	167	167	99	87	87	66	61	66	73	71	71	173	169	173	170	170	170	107	108	107	97	99	99	190	193	24	23
B8G	80	80	73	160	160	151	80	80	78	72	61	61	73	73	71	169	166	169	168	163	168	97	105	108	95	90	97	185	179	21	16
B8H	83	76	80	172	172	172	89	96	92	72	61	72	NA	73	77	173	169	171	175	172	168	101	108	99	NA	99	91	190	196	23	24
B8I	83	87	80	147	153	147	64	66	67	66	72	79	77	71	NA	166	169	169	163	161	161	100	97	90	86	90	NA	NA	182	NA	21
B8J	80	83	83	160	156	156	80	73	73	79	66	72	NA	73	80	169	166	164	NA	165	163	90	100	92	NA	92	83	189	179	24	14
B8K	80	76	83	153	147	160	73	71	77	96	66	72	77	NA	77	NA	169	169	NA	165	163	NA	103	97	NA	NA	86	188	182	21	17
B9A	83	83	76	165	165	167	82	82	91	66	86	79	80	84	NA	165	165	164	170	168	170	99	79	85	90	84	NA	182	184	20	16
B9B	83	83	83	160	165	165	77	82	82	66	61	79	77	73	84	166	163	166	170	165	168	100	102	87	93	92	84	185	179	24	14
B9C	80	76	87	167	165	167	87	89	80	61	61	79	77	71	77	173	169	171	170	165	168	112	108	92	93	94	91	188	193	22	28
B9D	83	80	80	172	174	172	89	94	92	66	61	61	77	71	80	178	176	178	172	172	170	112	115	117	95	101	90	186	189	13	17
B9E	87	83	94	156	160	156	69	77	62	66	86	79	80	73	84	169	169	166	165	158	168	103	83	87	85	85	84	186	182	20	24
B9F	76	80	80	156	160	165	80	80	85	72	86	82	80	71	73	169	165	166	163	158	161	97	79	84	83	87	88	188	177	25	19
B9G	97	90	90	160	153	153	63	63	63	99	72	86	98	98	91	166	158	162	161	158	156	67	86	76	63	60	65	NA	179	NA	21
B9H	76	76	80	156	151	156	80	75	76	61	61	82	NA	67	67	165	162	169	161	165	161	104	101	87	NA	98	94	182	179	21	14
B9I	87	83	76	167	167	165	80	84	89	66	61	61	NA	73	71	169	169	166	168	168	168	103	108	105	NA	95	97	184	186	18	18
B9J	104	90	90	165	165	172	61	75	82	79	66	82	84	84	91	169	166	169	168	161	161	90	100	87	84	77	70	184	177	19	16
B9K	83	73	83	165	165	167	82	92	84	61	61	72	71	NA	71	164	165	166	168	163	168	103	104	94	97	NA	97	185	177	22	14

Phenotype annotation: ordinal emergence date (Em), ordinal heading date (Hd), days to heading (Hd-Em), ordinal anthesis date (An) and days to anthesis (An-Hd).

Qi P, Pendergast TH, Johnson A, Bahri BA, Choi S, Missaoui A, Devos KM. 2021. Quantitative trait locus mapping combined with variant and transcriptome analyses identifies a cluster of gene candidates underlying the variation in leaf wax between upland and lowland switchgrass ecotypes. Theoretical and Applied Genetics 134, 1957–1975.

Supplementary Table S3. Average trait values for plants homozygous for the AP13 allele (delayed-flowering), homozygous for the VS16 allele (earlier-flowering) and heterozygous at the flowering time QTL on Chr04K.

Traits	Tag at the highest LR	AP13	VS16	Heterozygous	AP13-VS16
Hd_2019_Rep3	Tag_4384	167.4	162.3	164.3	5.1
Hd_2019_Rep2	Tag_4322	166.4	161.9	163.2	4.5
Hd_2019_Rep1	Tag_4246	168.2	164.8	164.4	3.4
Hd_2017_Rep3	Tag_4385	163.1	155.0	159.0	8.2
Hd_2017_Rep2	Tag_4385	163.5	155.5	159.0	8.0
Hd_2017_Rep1	Tag_4384	164.8	156.5	159.4	8.2
Hd_Em_2019_Rep3	Tag_4372	94.3	85.9	89.7	8.5
Hd_Em_2019_Rep2	Tag_4435	93.4	86.5	88.8	6.8
Hd_Em_2019_Rep1	Tag_4353	94.9	88.3	90.4	6.7
Hd_Em_2017_Rep3	Tag_4384	81.3	74.4	77.2	6.9
Hd_Em_2017_Rep2	Tag_4385	82.9	75.1	78.4	7.8
Hd_Em_2017_Rep1	Tag_4385	82.5	73.9	76.7	8.6
An_Hd_2019_Rep2	Tag_4569	18.1	15.9	17.9	2.1
An_2019_Rep2	Tag_4322	184.9	178.3	180.4	6.6
An_2018_Rep2	Tag_4387	189.1	186.3	186.7	2.8

Phenotype annotation: ordinal emergence date (Em), ordinal heading date (Hd), days to heading (Hd-Em), ordinal anthesis date (An) and days to anthesis (An-Hd).

Supplementary Table S4. Annotated genes (Lovell *et al.* , 2021) underlying the flowering time QTL on chromosome 4K

Locus	Gene ID	Chr	Start (bp)	End (bp)	Functional_annotation_inswitchgrassgenome_v5.1	UniprotID	Protein names	Gene names	Organism	Function [CC]	Gene ontology IDs	Gene ontology (biological process)
Pavir.4KG397500	Pavir.4KG397500.v5.1	Chr04K	12390966	12392640	(1 of 31) PF04674 - Phosphate-induced protein 1 conserved region (Phi_1)	PHI1_TOBAC	Protein PHOSPHATE-INDUCED 1	PHI-1	Nicotiana tabacum (Common tobacco)	FUNCTION: May be involved in the regulation of cell division. {ECO:0000269|PubMed:10189698}.	GO:0005615; GO:0048046	
Pavir.4KG397600	Pavir.4KG397600.v5.1	Chr04K	12398242	12399629	(1 of 31) PF04674 - Phosphate-induced protein 1 conserved region (Phi_1)	EXOL2_ARATH	Protein EXORDIUM-like 2	EXL2 At5g64260 MSJ1.10	Arabidopsis thaliana (Mouse-ear cress)	FUNCTION: May play a role in a brassinosteroid-dependent regulation of growth and development. {ECO:0000250}.	GO:0005615; GO:0005618; GO:0005794; GO:0005829; GO:0009505; GO:0009506; GO:0009536; GO:0048046; GO:0071456	cellular response to hypoxia [GO:0071456]
Pavir.4KG397700	Pavir.4KG397700.v5.1	Chr04K	12409523	12413195	(1 of 2) K17605 - serine/threonine-protein phosphatase 2A activator (PPP2R4, PTPA)	PTPA_RABIT	Serine/threonine-protein phosphatase 2A activator (EC 5.2.1.8) (PP2A, subunit B', PR53 isoform) (Phosphotyrosyl phosphatase activator) (PTPA) (Serine/threonine-protein phosphatase 2A regulatory subunit 4) (Serine/threonine-protein phosphatase 2A regulatory subunit B')	PTPA PPP2R4	Oryctolagus cuniculus (Rabbit)	FUNCTION: PPIases accelerate the folding of proteins. It catalyzes the cis-trans isomerization of proline imidic peptide bonds in oligopeptides. Acts as a regulatory subunit for serine/threonine-protein phosphatase 2A (PP2A) modulating its activity or substrate specificity, probably by inducing a conformational change in the catalytic subunit, a proposed direct target of the PPIase. Can reactivate inactive phosphatase PP2A-phosphatase methylesterase complexes (PP2A(i)) in presence of ATP and Mg(2+). Reversibly stimulates the variable phosphotyrosyl phosphatase activity of PP2A core heterodimer PP2A(D) in presence of ATP and Mg(2+) (in vitro). The phosphotyrosyl phosphatase activity is dependent of an ATPase activity of the PP2A(D):PPP2R4 complex. Is involved in apoptosis; the function appears to be independent from PP2A (By similarity). {ECO:0000250, ECO:0000269|PubMed:14748741, ECO:0000269|PubMed:16380387}.	GO:0000159; GO:0003755; GO:0005524; GO:0005634; GO:0005737; GO:0008160; GO:0019888; GO:0032515; GO:0032516; GO:0035307; GO:0035308; GO:0042803; GO:0043666; GO:0051721	negative regulation of phosphoprotein phosphatase activity [GO:0032515]; negative regulation of protein dephosphorylation [GO:0035308]; positive regulation of phosphoprotein phosphatase activity [GO:0032516]; positive regulation of protein dephosphorylation [GO:0035307]; regulation of phosphoprotein phosphatase activity [GO:0043666]
Pavir.4KG397800	Pavir.4KG397800.v5.1	Chr04K	12413404	12417410	(1 of 332) PF01535//PF13041 - PPR repeat (PPR) // PPR repeat family (PPR_2)	PP244_ARATH	Pentatricopeptide repeat-containing protein At3g20730	PCMP-E94 At3g20730 MOE17.3	Arabidopsis thaliana (Mouse-ear cress)			
Pavir.4KG397900	Pavir.4KG397900.v5.1	Chr04K	12417972	12418446	(1 of 99) PF00025 - ADP-ribosylation factor family (Arf)	#N/A	#N/A	#N/A	#N/A	#N/A	#N/A	#N/A
Pavir.4KG398000	Pavir.4KG398000.v5.1	Chr04K	12424018	12425462	(1 of 3) PTHR11711//PTHR11711:SF183 - ADP RIBOSYLATION FACTOR-RELATED // SUBFAMILY NOT NAMED	ARF_DUGJA	ADP-ribosylation factor		Dugesia japonica (Planarian)	FUNCTION: GTP-binding protein involved in protein trafficking; may modulate vesicle budding and uncoating within the Golgi apparatus. {ECO:0000250}.	GO:0005525; GO:0005794; GO:0015031; GO:0016192	protein transport [GO:0015031]; vesicle-mediated transport [GO:0016192]
Pavir.4KG398100	Pavir.4KG398100.v5.1	Chr04K	12425929	12430250	(1 of 7) K14396 - polyadenylate-binding protein 2 (PABPN1, PABP2)	PABN2_ARATH	Polyadenylate-binding protein 2 (AtPabN2) (AtPabN3) (Poly(A)-binding protein 2) (Nuclear poly(A)-binding protein 2) (Poly(A)-binding protein II) (PABII)	PABN2 At5g65260 MQN23.20	Arabidopsis thaliana (Mouse-ear cress)	FUNCTION: Involved in the 3'-end formation of mRNA precursors (pre-mRNA) by the addition of a poly(A) tail of 200-250 nt to the upstream cleavage product. Stimulates poly(A) polymerase (PAPOLA) conferring processivity on the poly(A) tail elongation reaction and controls also the poly(A) tail length. Increases the affinity of poly(A) polymerase for RNA. Binds to poly(A) and to poly(G) with high affinity. May protect the poly(A) tail from degradation. {ECO:0000250|UniProtKB:Q7KNF2}.	GO:0003729; GO:0005737; GO:0016607	
Pavir.4KG398105	Pavir.4KG398105.v5.1	Chr04K	12445939	12447015		#N/A	#N/A	#N/A	#N/A	#N/A	#N/A	#N/A
Pavir.4KG218000	Pavir.4KG218000.v5.1	Chr04K	12493734	12495149	(1 of 8) PTHR11527//PTHR11527:SF166 - SMALL HEAT-SHOCK PROTEIN HSP20 FAMILY // SUBFAMILY NOT NAMED	HS26M_ORYSJ	26.2 kDa heat shock protein, mitochondrial (OsHsp26.2)	HSP26.2 Os06g0219500 LOC_Os06g11610 P0436F11.23	Oryza sativa subsp. japonica (Rice)		GO:0005739; GO:0009408	response to heat [GO:0009408]
Pavir.4KG218200	Pavir.4KG218200.v5.1	Chr04K	12510852	12511392		#N/A	#N/A	#N/A	#N/A	#N/A	#N/A	#N/A
Pavir.4KG218205	Pavir.4KG218205.v5.1	Chr04K	12525763	12527949		#N/A	#N/A	#N/A	#N/A	#N/A	#N/A	#N/A
Pavir.4KG267000	Pavir.4KG267000.v5.1	Chr04K	12538268	12539928	(1 of 2) PTHR22847//PTHR22847:SF430 - WD40 REPEAT PROTEIN // MZB10.11 PROTEIN-RELATED	#N/A	#N/A	#N/A	#N/A	#N/A	#N/A	#N/A
Pavir.4KG218400	Pavir.4KG218400.v5.1	Chr04K	12556773	12557914	(1 of 8) PTHR11527//PTHR11527:SF166 - SMALL HEAT-SHOCK PROTEIN HSP20 FAMILY // SUBFAMILY NOT NAMED	HS26M_ORYSJ	26.2 kDa heat shock protein, mitochondrial (OsHsp26.2)	HSP26.2 Os06g0219500 LOC_Os06g11610 P0436F11.23	Oryza sativa subsp. japonica (Rice)		GO:0005739; GO:0009408	response to heat [GO:0009408]
Pavir.4KG218500	Pavir.4KG218500.v5.1	Chr04K	12558662	12562908	(1 of 67) PF10250 - GDP-fucose protein O-fucosyltransferase (O-FucT)	OFUT7_ARATH	O-fucosyltransferase 7 (O-FucT-7) (EC 2.4.1.-) (O-fucosyltransferase family protein)	OFUT7 At1g22460 F12K8.19	Arabidopsis thaliana (Mouse-ear cress)		GO:0005737; GO:0006004; GO:0016021; GO:0016757	fucose metabolic process [GO:0006004]
Pavir.4KG218600	Pavir.4KG218600.v5.1	Chr04K	12587023	12589847	(1 of 4) PF06941 - 5' nucleotidase, deoxy (Pyrimidine), cytosolic type C protein (NT5C) (NT5C)	#N/A	#N/A	#N/A	#N/A	#N/A	#N/A	#N/A
Pavir.4KG218700	Pavir.4KG218700.v5.1	Chr04K	12590268	12594379	(1 of 88) PF01535//PF13041//PF14432 - PPR repeat (PPR) // PPR repeat family (PPR_2) // DYW family of nucleic acid deaminases (DYW_deaminase)	PP251_ARATH	Putative pentatricopeptide repeat-containing protein At3g23330	PCMP-H32 At3g23330 MLM24.6	Arabidopsis thaliana (Mouse-ear cress)		GO:0003723; GO:0008270; GO:0009451; GO:0043231	RNA modification [GO:0009451]

Pavir.4KG218800	Pavir.4KG218800.v5.1	Chr04K	12596612	12603229	(1 of 6) KOG2296 - Integral membrane protein	LMD2A_DICDI	LMBR1 domain-containing protein 2 homolog A	DDB_G0284019	Dictyostelium discoideum (Slime mold)		GO:0016020; GO:0016021	
Pavir.4KG212000	Pavir.4KG212000.v5.1	Chr04K	12642774	12645678	(1 of 49) PF03080 - Domain of unknown function (DUF239) (DUF239)	#N/A	#N/A	#N/A	#N/A	#N/A	#N/A	#N/A
Pavir.4KG212005	Pavir.4KG212005.v5.1	Chr04K	12665332	12665775		#N/A	#N/A	#N/A	#N/A	#N/A	#N/A	#N/A
Pavir.4KG212010	Pavir.4KG212010.v5.1	Chr04K	12674873	12676971	(1 of 73) PF01535//PF12854//PF13041 - PPR repeat (PPR) // PPR repeat (PPR_1) // PPR repeat family (PPR_2)	#N/A	#N/A	#N/A	#N/A	#N/A	#N/A	#N/A
Pavir.4KG212015	Pavir.4KG212015.v5.1	Chr04K	12679611	12681007	(1 of 2827) 2.7.11.1 - Non-specific serine/threonine protein kinase / Threonine-specific protein kinase	CERK1_ARATH	Chitin elicitor receptor kinase 1 (AtCERK1) (EC 2.7.11.1) (LysM domain receptor-like kinase 1) (LysM RLK1) (LysM-containing receptor-like kinase 1)	CERK1 LYK1 RLK1 At3g21630 MIL23.20	Arabidopsis thaliana (Mouse-ear cress)	FUNCTION: Lysin motif (LysM) receptor kinase that functions as a cell surface receptor in chitin elicitor (chitooligosaccharides) signaling leading to innate immunity toward both biotic and abiotic stresses (e.g. tolerance to salinity, heavy-metal stresses, and Botrytis cinerea infection). Recognizes microbe-derived N-acetylglucosamine (NAG)-containing ligands. Involved in the resistance to pathogenic fungi Alternaria brassicicola and Erysiphe cichoracearum, probably by sensing microbe-associated molecular patterns (MAMP) and pathogen-associated molecular patterns (PAMP). Plays an essential role in detecting peptidoglycans (e.g. PGNs) and restricting bacterial growth. Target of the bacterial type III effector E3-ligase protein hopAB2/avrPtoB of Pseudomonas syringae pv. tomato DC3000 that mediates ubiquitination and subsequent proteolysis, thus blocking all defense responses by suppressing PAMP-triggered immunity (PTI). Mediates chitin-induced phosphorylation of PBL27 (PubMed:24750441). {ECO:0000269|PubMed:18042724, ECO:0000269|PubMed:18263776, ECO:0000269|PubMed:19249211, ECO:0000269|PubMed:19816132, ECO:0000269|PubMed:20610395, ECO:0000269|PubMed:22106285, ECO:0000269|PubMed:22461667, ECO:0000269|PubMed:22891159, ECO:0000269|PubMed:24750441}.	GO:0002752; GO:0004672; GO:0004674; GO:0005524; GO:0005886; GO:0006468; GO:0008061; GO:0009817; GO:0010200; GO:0016021; GO:0016301; GO:0019199; GO:0032491; GO:0032499; GO:0035556; GO:0042742; GO:0042802; GO:0042803; GO:0043621; GO:0045087; GO:0046777; GO:0071219; GO:0071323; GO:2001080	cell surface pattern recognition receptor signaling pathway [GO:0002752]; cellular response to chitin [GO:0071323]; cellular response to molecule of bacterial origin [GO:0071219]; defense response to bacterium [GO:0042742]; defense response to fungus, incompatible interaction [GO:0009817]; detection of molecule of fungal origin [GO:0032491]; detection of peptidoglycan [GO:0032499]; innate immune response [GO:0045087]; intracellular signal transduction [GO:0035556]; protein autophosphorylation [GO:0046777]; protein phosphorylation [GO:0006468]; response to chitin [GO:0010200]
Pavir.4KG212020	Pavir.4KG212020.v5.1	Chr04K	12682506	12684688		#N/A	#N/A	#N/A	#N/A	#N/A	#N/A	#N/A
Pavir.4KG229200	Pavir.4KG229200.v5.1	Chr04K	12686034	12687637	(1 of 12) PTHR22893//PTHR22893:SF44 - NADH OXIDOREDUCTASE-RELATED // SUBFAMILY NOT NAMED	OPR1_ORYSJ	12-oxophytodienoate reductase 1 (EC 1.3.1.42) (12-oxophytodienoate-10,11-reductase 1) (OPDA-reductase 1) (OsOPR1)	OPR1 OPDA OPDA2 OPDAR1 OPR11 OPR2 RRJ4 Os06g0216300 LOC_Os06g11290 OsJ_20595 OSJNBb0024N18.13 P0537F07.35	Oryza sativa subsp. japonica (Rice)	FUNCTION: Probably involved in the biosynthesis or metabolism of oxylipin signaling molecules. In vitro, reduces cis(-)-12-oxophytodienoic acid (cis(-)-OPDA) and to cis(-)-OPC-8:0. {ECO:0000269|PubMed:12569412, ECO:0000269|PubMed:17938955}.	GO:0006952; GO:0009695; GO:0010181; GO:0016491; GO:0016629; GO:0031408	defense response [GO:0006952]; jasmonic acid biosynthetic process [GO:0009695]; oxylipin biosynthetic process [GO:0031408]
Pavir.4KG229205	Pavir.4KG229205.v5.1	Chr04K	12692511	12694245	(1 of 1) PTHR22893//PTHR22893:SF63 - NADH OXIDOREDUCTASE-RELATED // SUBFAMILY NOT NAMED	OPR5_ORYSJ	Putative 12-oxophytodienoate reductase 5 (EC 1.3.1.-) (OPDA-reductase 5) (OsOPR5)	OPR5 OPR-1 OPR6 Os06g0215600 LOC_Os06g11210 OSJNBb0024N18.5 P0537F07.27	Oryza sativa subsp. japonica (Rice)	FUNCTION: Putative oxophytodienoate reductase that may be involved in the biosynthesis or metabolism of oxylipin signaling molecules. {ECO:0000250}.	GO:0009695; GO:0010181; GO:0016491; GO:0031408	jasmonic acid biosynthetic process [GO:0009695]; oxylipin biosynthetic process [GO:0031408]
Pavir.4KG229210	Pavir.4KG229210.v5.1	Chr04K	12710343	12711787	(1 of 12) PTHR22893//PTHR22893:SF44 - NADH OXIDOREDUCTASE-RELATED // SUBFAMILY NOT NAMED	OPR1_ORYSJ	12-oxophytodienoate reductase 1 (EC 1.3.1.42) (12-oxophytodienoate-10,11-reductase 1) (OPDA-reductase 1) (OsOPR1)	OPR1 OPDA OPDA2 OPDAR1 OPR11 OPR2 RRJ4 Os06g0216300 LOC_Os06g11290 OsJ_20595 OSJNBb0024N18.13 P0537F07.35	Oryza sativa subsp. japonica (Rice)	FUNCTION: Probably involved in the biosynthesis or metabolism of oxylipin signaling molecules. In vitro, reduces cis(-)-12-oxophytodienoic acid (cis(-)-OPDA) and to cis(-)-OPC-8:0. {ECO:0000269|PubMed:12569412, ECO:0000269|PubMed:17938955}.	GO:0006952; GO:0009695; GO:0010181; GO:0016491; GO:0016629; GO:0031408	defense response [GO:0006952]; jasmonic acid biosynthetic process [GO:0009695]; oxylipin biosynthetic process [GO:0031408]
Pavir.4KG229215	Pavir.4KG229215.v5.1	Chr04K	12722838	12724609	(1 of 12) PTHR22893//PTHR22893:SF44 - NADH OXIDOREDUCTASE-RELATED // SUBFAMILY NOT NAMED	OPR1_ORYSJ	12-oxophytodienoate reductase 1 (EC 1.3.1.42) (12-oxophytodienoate-10,11-reductase 1) (OPDA-reductase 1) (OsOPR1)	OPR1 OPDA OPDA2 OPDAR1 OPR11 OPR2 RRJ4 Os06g0216300 LOC_Os06g11290 OsJ_20595 OSJNBb0024N18.13 P0537F07.35	Oryza sativa subsp. japonica (Rice)	FUNCTION: Probably involved in the biosynthesis or metabolism of oxylipin signaling molecules. In vitro, reduces cis(-)-12-oxophytodienoic acid (cis(-)-OPDA) and to cis(-)-OPC-8:0. {ECO:0000269|PubMed:12569412, ECO:0000269|PubMed:17938955}.	GO:0006952; GO:0009695; GO:0010181; GO:0016491; GO:0016629; GO:0031408	defense response [GO:0006952]; jasmonic acid biosynthetic process [GO:0009695]; oxylipin biosynthetic process [GO:0031408]

Pavir.4KG229220	Pavir.4KG229220.v5.1	Chr04K	12729093	12730954	(1 of 12) PTHR22893//PTHR22893:SF44 - NADH OXIDOREDUCTASE-RELATED // SUBFAMILY NOT NAMED	OPR1_ORYSJ	12-oxophytodienoate reductase 1 (EC 1.3.1.42) (12-oxophytodienoate-10,11-reductase 1) (OPDA-reductase 1) (OsOPR1)	OPR1 OPDA OPDA2 OPDAR1 OPR11 OPR2 RRJ4 Os06g0216300 LOC_Os06g11290 OsJ_20595 OSJNBb0024N18.13 P0537F07.35	Oryza sativa subsp. japonica (Rice)	FUNCTION: Probably involved in the biosynthesis or metabolism of oxylipin signaling molecules. In vitro, reduces cis(-)-12-oxophytodienoic acid (cis(-)-OPDA) and to cis(-)-OPC-8:0. {ECO:0000269|PubMed:12569412, ECO:0000269|PubMed:17938955}.	GO:0006952; GO:0009695; GO:0010181; GO:0016491; GO:0016629; GO:0031408	defense response [GO:0006952]; jasmonic acid biosynthetic process [GO:0009695]; oxylipin biosynthetic process [GO:0031408]
Pavir.4KG229100	Pavir.4KG229100.v5.1	Chr04K	12748536	12756141	(1 of 9) 3.4.22.40 - Bleomycin hydrolase / Aminopeptidase C (Lactococcus lactis)	DPF6_CAEEL	Dipeptidyl peptidase family member 6 (EC 3.4.14.-)	dpf-6 F44B9.1	Caenorhabditis elegans	FUNCTION: Removes N-terminal dipeptides sequentially from polypeptides (By similarity). Essential for control of distal tip cell migration. {ECO:0000250}.	GO:0004177; GO:0005886; GO:0008236; GO:0016021	
Pavir.4KG229000	Pavir.4KG229000.v5.1	Chr04K	12768146	12774833	(1 of 9) 3.4.22.40 - Bleomycin hydrolase / Aminopeptidase C (Lactococcus lactis)	DAPB3_PSEMX	Dipeptidyl aminopeptidase BIII (DAP BIII) (EC 3.4.14.-)	dapb3	Pseudoxanthomonas mexicana	FUNCTION: Exopeptidase that catalyzes the removal of dipeptide units (NH2-P2-P1- or -P1'-P2'-COOH) from the free amino or carboxy termini. Prefers substrates composed of bulky, hydrophobic amino acids at P1 and P1' positions. Has endopeptidase activity on N-terminally blocked peptide derivatives which contain aromatic amino acid residue at the P1 position. Exopeptidase activity is much higher than its endopeptidase activity. {ECO:0000269|PubMed:8892831}.	GO:0004175; GO:0008236; GO:0008239; GO:0051603	proteolysis involved in cellular protein catabolic process [GO:0051603]
Pavir.4KG229005	Pavir.4KG229005.v5.1	Chr04K	12775397	12779213	(1 of 1) PTHR13173:SF10 - WW DOMAIN-BINDING PROTEIN 4	ZOP1_ARATH	Zinc finger protein ZOP1 (Zinc-finger and OCRE domain-containing protein 1)	ZOP1 At1g49590 F14J22.17	Arabidopsis thaliana (Mouse-ear cress)	FUNCTION: Nucleic acid-binding protein that promotes Pol IV-dependent small interfering RNA (siRNA) accumulation, DNA methylation and transcriptional silencing. May possess both RNA-directed DNA methylation (RdDM)-dependent and -independent roles in transcriptional silencing. Acts as a pre-mRNA splicing factor that associates with several typical components of the splicing machinery as well as with Pol II. {ECO:0000269|PubMed:23524848}.	GO:0000398; GO:0003690; GO:0003723; GO:0003725; GO:0008270; GO:0008380; GO:0009845; GO:0015030; GO:0071011; GO:0080188	gene silencing by RNA-directed DNA methylation [GO:0080188]; mRNA splicing, via spliceosome [GO:0000398]; RNA splicing [GO:0008380]; seed germination [GO:0009845]
Pavir.4KG228000	Pavir.4KG228000.v5.1	Chr04K	12785078	12786700	(1 of 24) PF07816 - Protein of unknown function (DUF1645) (DUF1645)	#N/A	#N/A	#N/A	#N/A	#N/A	#N/A	#N/A
Pavir.4KG228005	Pavir.4KG228005.v5.1	Chr04K	12786980	12787593		#N/A	#N/A	#N/A	#N/A	#N/A	#N/A	#N/A
Pavir.4KG227900	Pavir.4KG227900.v5.1	Chr04K	12791676	12798301	(1 of 2) 1.6.5.9//3.1.1.1 - NADH:ubiquinone reductase (non-electrogenic) / Ubiquinone reductase // Carboxylesterase / Procaine esterase	NDC1_ARATH	Alternative NAD(P)H-ubiquinone oxidoreductase C1, chloroplastic/mitochondrial (EC 1.6.5.9) (Alternative NADH dehydrogenase NDC1) (Demethylphylloquinone reductase NDC1) (EC 1.6.5.12) (NADH:ubiquinone reductase (non-electrogenic) NDC1)	NDC1 At5g08740 T2K12.12 T2K12_90	Arabidopsis thaliana (Mouse-ear cress)	FUNCTION: Bifunctional oxidoreductase ables to act both on prenyl naphthoquinones and on prenyl benzoquinones (PubMed:21844348, PubMed:26023160). May serve a respiratory function (PubMed:12972666). Involved in an electron flow toward the plastoglobule plastoquinone pool (PubMed:21844348, PubMed:25018761). Required for plastochromanol-8 accumulation and for phylloquinone (vitamin K1) production (PubMed:21844348, PubMed:25018761). Probably not directly involved in cyclic or chlororespiratory electron flows under standard growth conditions, but participates in the redox metabolism of plastoquinone-9 and the tocophrol recycling-intermediate alpha-tocopherol quinone (PubMed:21844348, PubMed:25018761). Catalyzes the penultimate step in the biosynthesis of vitamin K1 (PubMed:26023160). {ECO:0000269|PubMed:12972666, ECO:0000269|PubMed:21844348, ECO:0000269|PubMed:25018761, ECO:0000269|PubMed:26023160}.	GO:0003955; GO:0005739; GO:0005743; GO:0009507; GO:0010287; GO:0016491; GO:0019646; GO:0042372; GO:0055114; GO:0071482	aerobic electron transport chain [GO:0019646]; cellular response to light stimulus [GO:0071482]; oxidation-reduction process [GO:0055114]; phylloquinone biosynthetic process [GO:0042372]
Pavir.4KG227905	Pavir.4KG227905.v5.1	Chr04K	12798279	12799630	(1 of 2) PTHR23024//PTHR23024:SF199 - MEMBER OF 'GDXG' FAMILY OF LIPOLYTIC ENZYMES // SUBFAMILY NOT NAMED	HIDM_GLYEC	2-hydroxyisoflavanone dehydratase (EC 3.1.1.1) (EC 4.2.1.105) (Carboxylesterase HIDM)	HIDM	Glycyrrhiza echinata (Licorice)	FUNCTION: Dehydratase that mediates the biosynthesis of isoflavonoids. Can better use 2,7-dihydroxy-4'-methoxyisoflavanone as substrate. Has also a slight carboxylesterase activity toward p-nitrophenyl butyrate. {ECO:0000269|PubMed:15734910}.	GO:0009717; GO:0009813; GO:0033987; GO:0046287; GO:0052689; GO:0080030	flavonoid biosynthetic process [GO:0009813]; isoflavonoid biosynthetic process [GO:0009717]; isoflavonoid metabolic process [GO:0046287]
Pavir.4KG227910	Pavir.4KG227910.v5.1	Chr04K	12805734	12806078		#N/A	#N/A	#N/A	#N/A	#N/A	#N/A	#N/A
Pavir.4KG224700	Pavir.4KG224700.v5.1	Chr04K	12809807	12811051	(1 of 6) PTHR23024//PTHR23024:SF131 - MEMBER OF 'GDXG' FAMILY OF LIPOLYTIC ENZYMES // CARBOXYLESTERASE 2-RELATED	TCEA1_TULGE	Tuliposide A-converting enzyme 1, chloroplastic (TgTCEA1) (EC 4.2.99.22)	TCEA1 TCEA5	Tulipa gesneriana (Garden tulip)	FUNCTION: Lactone-forming carboxylesterases, specifically catalyzing intramolecular transesterification, but not hydrolysis. Involved in the biosynthesis of tulipalins, defensive chemicals that show antimicrobial activities against a broad range of strains of bacteria and fungi. Substrates are 6-tuliposide A > 6-tuliposide B. {ECO:0000269|PubMed:22474185}.	GO:0006952; GO:0009507; GO:0016787; GO:0016829	defense response [GO:0006952]
Pavir.4KG224705	Pavir.4KG224705.v5.1	Chr04K	12819203	12819553		#N/A	#N/A	#N/A	#N/A	#N/A	#N/A	#N/A
Pavir.4KG224710	Pavir.4KG224710.v5.1	Chr04K	12823488	12824647		#N/A	#N/A	#N/A	#N/A	#N/A	#N/A	#N/A
Pavir.4KG227700	Pavir.4KG227700.v5.1	Chr04K	12825486	12826608	(1 of 8) PTHR11527//PTHR11527:SF166 - SMALL HEAT-SHOCK PROTEIN HSP20 FAMILY // SUBFAMILY NOT NAMED	#N/A	#N/A	#N/A	#N/A	#N/A	#N/A	#N/A
Pavir.4KG227705	Pavir.4KG227705.v5.1	Chr04K	12827208	12832607		#N/A	#N/A	#N/A	#N/A	#N/A	#N/A	#N/A
Pavir.4KG227600	Pavir.4KG227600.v5.1	Chr04K	12835560	12836373		#N/A	#N/A	#N/A	#N/A	#N/A	#N/A	#N/A
Pavir.4KG227500	Pavir.4KG227500.v5.1	Chr04K	12836706	12845001	(1 of 2) K08735 - DNA mismatch repair protein MSH2 (MSH2)	MSH2_MAIZE	DNA mismatch repair protein MSH2 (MUS1)	MUS1	Zea mays (Maize)	FUNCTION: Involved in postreplication mismatch repair. Binds specifically to DNA containing mismatched nucleotides thus providing a target for the excision repair processes characteristic of postreplication mismatch repair (By similarity). {ECO:0000250}.	GO:0000406; GO:0005524; GO:0005634; GO:0006298; GO:0006301; GO:0006310; GO:0008094; GO:0030983; GO:0032300; GO:0032301; GO:0032302; GO:0043570; GO:0045910	DNA recombination [GO:0006310]; maintenance of DNA repeat elements [GO:0043570]; mismatch repair [GO:0006298]; negative regulation of DNA recombination [GO:0045910]; postreplication repair [GO:0006301]

Pavir.4KG227400	Pavir.4KG227400.v5.1	Chr04K	12851281	12852686	(1 of 4) PTHR10265//PTHR10265:SF21 - CYCLIN-DEPENDENT KINASE INHIBITOR 1 // SUBFAMILY NOT NAMED	KRP2_ORYSJ	Cyclin-dependent kinase inhibitor 2 (KIP-related protein 2)	KRP2 Os06g0213700 LOC_Os06g11050 P0537F07.1 P0701E03.40	Oryza sativa subsp. japonica (Rice)		GO:0004861; GO:0005634; GO:0007050	cell cycle arrest [GO:0007050]
Pavir.4KG227300	Pavir.4KG227300.v5.1	Chr04K	12857175	12861795	(1 of 8) KOG2521 - Uncharacterized conserved protein	#N/A	#N/A	#N/A	#N/A	#N/A	#N/A	#N/A
Pavir.4KG227200	Pavir.4KG227200.v5.1	Chr04K	12867867	12868864	(1 of 4) PTHR10891//PTHR10891:SF634 - EF-HAND CALCIUM-BINDING DOMAIN CONTAINING PROTEIN // SUBFAMILY NOT NAMED	CML38_ARATH	Calcium-binding protein CML38 (Calmodulin-like protein 38)	CML38 At1g76650 F28O16.2	Arabidopsis thaliana (Mouse-ear cress)	FUNCTION: Potential calcium sensor that binds calcium in vitro. {ECO:0000269|PubMed:17579812}.	GO:0005509; GO:0005886; GO:0009611; GO:0071456	cellular response to hypoxia [GO:0071456]; response to wounding [GO:0009611]
Pavir.4KG227100	Pavir.4KG227100.v5.1	Chr04K	12869843	12873003	(1 of 4) PTHR10891//PTHR10891:SF634 - EF-HAND CALCIUM-BINDING DOMAIN CONTAINING PROTEIN // SUBFAMILY NOT NAMED	CML38_ARATH	Calcium-binding protein CML38 (Calmodulin-like protein 38)	CML38 At1g76650 F28O16.2	Arabidopsis thaliana (Mouse-ear cress)	FUNCTION: Potential calcium sensor that binds calcium in vitro. {ECO:0000269|PubMed:17579812}.	GO:0005509; GO:0005886; GO:0009611; GO:0071456	cellular response to hypoxia [GO:0071456]; response to wounding [GO:0009611]
Pavir.4KG224500	Pavir.4KG224500.v5.1	Chr04K	12878709	12891298	(1 of 2) PF11817//PF12584 - Foie gras liver health family 1 (Foie-gras_1) // Trafficking protein particle complex subunit 10, TRAPPC10 (TRAPPC10)	TR130_ORYSJ	Trafficking protein particle complex II-specific subunit 130 homolog (TRAPP II-specific subunit 130 homolog)	TRS130 Os12g0554400 LOC_Os12g36760	Oryza sativa subsp. japonica (Rice)	FUNCTION: Specific subunit of the TRAPP II complex, a highly conserved vesicle tethering complex that is required for the proper transport of proteins in post-Golgi trafficking pathways to the growing cell plate in mitotic active cells. {ECO:0000250|UniProtKB:F4K0C4}.	GO:0000919; GO:0005769; GO:0005802; GO:0005829; GO:0006891; GO:0034498; GO:1990071	cell plate assembly [GO:0000919]; early endosome to Golgi transport [GO:0034498]; intra-Golgi vesicle-mediated transport [GO:0006891]
Pavir.4KG224400	Pavir.4KG224400.v5.1	Chr04K	12895647	12899780	(1 of 2) PTHR33926//PTHR33926:SF2 - FAMILY NOT NAMED // PROTEIN TIC 22, CHLOROPLASTIC	TIC22_PEA	Protein TIC 22, chloroplastic (Translocon at the inner envelope membrane of chloroplasts 22) (PsTIC22)	TIC22 IAP25	Pisum sativum (Garden pea)	FUNCTION: Involved in protein precursor import into chloroplasts. Imported into the intermembrane space via the Toc translocon. May be involved in the import pathway used by proteins without a cleavable N-terminal pre-sequence. {ECO:0000269|PubMed:8707818, ECO:0000269|PubMed:9412463, ECO:0000269|PubMed:9817756}.	GO:0015031; GO:0016020; GO:0031972	protein transport [GO:0015031]
Pavir.4KG224300	Pavir.4KG224300.v5.1	Chr04K	12901118	12903191	(1 of 24) K13681 - xyloglucan fucosyltransferase [EC:2.4.1.-] Fuc a1-2 Gal (FUT)	FUT1_ARATH	Galactoside 2-alpha-L-fucosyltransferase (EC 2.4.1.-) (Xyloglucan alpha-(1,2)-fucosyltransferase) (AtFUT1)	FUT1 FT1 MUR2 At2g03220 T18E12.11	Arabidopsis thaliana (Mouse-ear cress)	FUNCTION: Involved in cell wall biosynthesis. Is both necessary and sufficient for the addition of the terminal fucosyl residue on xyloglucan side chains, but is not involved in the fucosylation of other cell wall components (PubMed:10373113, PubMed:11743104, PubMed:11854459, PubMed:14730072). Associates with other xyloglucan-synthesizing enzymes to form multiprotein complexes for xyloglucan synthesis in the Golgi (PubMed:25392066). {ECO:0000269|PubMed:10373113, ECO:0000269|PubMed:11743104, ECO:0000269|PubMed:11854459, ECO:0000269|PubMed:14730072, ECO:0000269|PubMed:25392066}.	GO:0005794; GO:0005797; GO:0008107; GO:0008417; GO:0009969; GO:0016021; GO:0032580; GO:0042803; GO:0071555	cell wall organization [GO:0071555]; xyloglucan biosynthetic process [GO:0009969]
Pavir.4KG224200	Pavir.4KG224200.v5.1	Chr04K	12903222	12905142	(1 of 24) K13681 - xyloglucan fucosyltransferase [EC:2.4.1.-] Fuc a1-2 Gal (FUT)	FUT1_PEA	Galactoside 2-alpha-L-fucosyltransferase (EC 2.4.1.-) (PsFT1) (Xyloglucan alpha-(1,2)-fucosyltransferase)	FT1	Pisum sativum (Garden pea)	FUNCTION: Involved in cell wall biosynthesis. Adds the terminal fucosyl residue on xyloglucan side chains. {ECO:0000269|PubMed:10747946}.	GO:0006486; GO:0008107; GO:0016021; GO:0032580; GO:0042546; GO:0071555	cell wall biogenesis [GO:0042546]; cell wall organization [GO:0071555]; protein glycosylation [GO:0006486]
Pavir.4KG224000	Pavir.4KG224000.v5.1	Chr04K	12908572	12911374	(1 of 24) K13681 - xyloglucan fucosyltransferase [EC:2.4.1.-] Fuc a1-2 Gal (FUT)	FUT1_PEA	Galactoside 2-alpha-L-fucosyltransferase (EC 2.4.1.-) (PsFT1) (Xyloglucan alpha-(1,2)-fucosyltransferase)	FT1	Pisum sativum (Garden pea)	FUNCTION: Involved in cell wall biosynthesis. Adds the terminal fucosyl residue on xyloglucan side chains. {ECO:0000269|PubMed:10747946}.	GO:0006486; GO:0008107; GO:0016021; GO:0032580; GO:0042546; GO:0071555	cell wall biogenesis [GO:0042546]; cell wall organization [GO:0071555]; protein glycosylation [GO:0006486]
Pavir.4KG224005	Pavir.4KG224005.v5.1	Chr04K	12913913	12916590	(1 of 31) 2.4.1.69 - Galactoside 2-alpha-L-fucosyltransferase / Secretor-type beta-galactoside alpha-1->2 fucosyltransferase	#N/A	#N/A	#N/A	#N/A	#N/A	#N/A	#N/A
Pavir.4KG223800	Pavir.4KG223800.v5.1	Chr04K	12935261	12937545	(1 of 13) PTHR24078:SF177 - PROTEIN DNJ-23-RELATED	RADL6_ARATH	Protein RADIALIS-like 6 (AtRL6) (Protein RAD-like 6) (Protein RADIALIS-LIKE SANT/MYB 3) (Protein RSM3)	RL6 RSM3 At1g75250 F22H5.3	Arabidopsis thaliana (Mouse-ear cress)	FUNCTION: Probable transcription factor. {ECO:0000250}.	GO:0003700; GO:0005634; GO:0006355	regulation of transcription, DNA-templated [GO:0006355]
Pavir.4KG223700	Pavir.4KG223700.v5.1	Chr04K	12946074	12948310	(1 of 2) PTHR31375//PTHR31375:SF34 - FAMILY NOT NAMED // PECTIN LYASE-LIKE SUPERFAMILY PROTEIN	#N/A	#N/A	#N/A	#N/A	#N/A	#N/A	#N/A
Pavir.4KG223100	Pavir.4KG223100.v5.1	Chr04K	12973094	12973660		#N/A	#N/A	#N/A	#N/A	#N/A	#N/A	#N/A
Pavir.4KG223105	Pavir.4KG223105.v5.1	Chr04K	13013647	13014925		#N/A	#N/A	#N/A	#N/A	#N/A	#N/A	#N/A
Pavir.4KG221000	Pavir.4KG221000.v5.1	Chr04K	13017132	13022029	(1 of 5) PTHR20961//PTHR20961:SF23 - GLYCOSYLTRANSFERASE // SUBFAMILY NOT NAMED	XAT2_ORYSJ	Alpha-1,3-arabinosyltransferase XAT2 (EC 2.4.2.-) (Xylan arabinosyltransferase 2) (OsXAT2)	XAT2 Os02g0330200 LOC_Os02g22480 OJ1116_E03.2 OsJ_06523	Oryza sativa subsp. japonica (Rice)	FUNCTION: Glycosyltransferase involved in the arabinosylation of xylan, the major hemicellulose (non-cellulosic component) of primary and secondary walls of angiosperms (PubMed:22215597). Possesses alpha-1,3-arabinosyltransferase activity, transferring an arabinofuranose residue to the xylan backbone (PubMed:22215597). {ECO:0000269|PubMed:22215597}.	GO:0000139; GO:0009664; GO:0016021; GO:0016757; GO:0052636	plant-type cell wall organization [GO:0009664]
Pavir.4KG221005	Pavir.4KG221005.v5.1	Chr04K	13037130	13037618		#N/A	#N/A	#N/A	#N/A	#N/A	#N/A	#N/A

Pavir.4KG220900	Pavir.4KG220900.v5.1	Chr04K	13041481	13042357		#N/A	#N/A	#N/A	#N/A	#N/A	#N/A	#N/A
Pavir.4KG220800	Pavir.4KG220800.v5.1	Chr04K	13059946	13060965		#N/A	#N/A	#N/A	#N/A	#N/A	#N/A	#N/A
Pavir.4KG220805	Pavir.4KG220805.v5.1	Chr04K	13104638	13105075		#N/A	#N/A	#N/A	#N/A	#N/A	#N/A	#N/A
Pavir.4KG220600	Pavir.4KG220600.v5.1	Chr04K	13108161	13114713	(1 of 2) PTHR12735//PTHR12735:SF6 - BOLA-LIKE PROTEIN-RELATED // SUBFAMILY NOT NAMED	BOLA4_ARATH	Protein BOLA4, chloroplastic/mitochondrial	BOLA4 At5g17560 K10A8.40	Arabidopsis thaliana (Mouse-ear cress)	FUNCTION: May act either alone or in interaction with glutaredoxin as a redox-regulated transcriptional regulator, or as a factor regulating Fe-S cluster biogenesis. {ECO:0000305|PubMed:24203231}.	GO:0005739; GO:0009507; GO:0009536	
Pavir.4KG220500	Pavir.4KG220500.v5.1	Chr04K	13114942	13119937	(1 of 18) PF13460 - NAD(P)H-binding (NAD_binding_10)	Y2446_ARATH	Uncharacterized protein At2g34460, chloroplastic	At2g34460 T31E10.20	Arabidopsis thaliana (Mouse-ear cress)		GO:0005783; GO:0009507; GO:0009534; GO:0009535; GO:0009941; GO:0010287	
Pavir.4KG220505	Pavir.4KG220505.v5.1	Chr04K	13143818	13149907	(1 of 15) PF16719 - SAWADEE domain (SAWADEE)	#N/A	#N/A	#N/A	#N/A	#N/A	#N/A	#N/A
Pavir.4KG220510	Pavir.4KG220510.v5.1	Chr04K	13159026	13160795	(1 of 15) PF16719 - SAWADEE domain (SAWADEE)	#N/A	#N/A	#N/A	#N/A	#N/A	#N/A	#N/A
Pavir.4KG220515	Pavir.4KG220515.v5.1	Chr04K	13172316	13178553	(1 of 15) PF16719 - SAWADEE domain (SAWADEE)	SHH2_ARATH	Protein SAWADEE HOMEODOMAIN HOMOLOG 2 (Probable DNA-binding transcription factor 2)	SHH2 At3g18380 MYF24.10	Arabidopsis thaliana (Mouse-ear cress)	FUNCTION: May play a role in the recruitment of Pol IV to genomic regions associated with K9 methylated histone H3 that are targets for RdDM.	GO:0003677; GO:0003682; GO:0005634	
Pavir.4KG233500	Pavir.4KG233500.v5.1	Chr04K	13194681	13196570	(1 of 598) PTHR19446//PTHR19446:SF355 - REVERSE TRANSCRIPTASES // SUBFAMILY NOT NAMED	#N/A	#N/A	#N/A	#N/A	#N/A	#N/A	#N/A
Pavir.4KG233505	Pavir.4KG233505.v5.1	Chr04K	13202384	13208514	(1 of 15) PF16719 - SAWADEE domain (SAWADEE)	SHH2_ARATH	Protein SAWADEE HOMEODOMAIN HOMOLOG 2 (Probable DNA-binding transcription factor 2)	SHH2 At3g18380 MYF24.10	Arabidopsis thaliana (Mouse-ear cress)	FUNCTION: May play a role in the recruitment of Pol IV to genomic regions associated with K9 methylated histone H3 that are targets for RdDM.	GO:0003677; GO:0003682; GO:0005634	
Pavir.4KG220400	Pavir.4KG220400.v5.1	Chr04K	13240833	13246958	(1 of 15) PF16719 - SAWADEE domain (SAWADEE)	SHH2_ARATH	Protein SAWADEE HOMEODOMAIN HOMOLOG 2 (Probable DNA-binding transcription factor 2)	SHH2 At3g18380 MYF24.10	Arabidopsis thaliana (Mouse-ear cress)	FUNCTION: May play a role in the recruitment of Pol IV to genomic regions associated with K9 methylated histone H3 that are targets for RdDM.	GO:0003677; GO:0003682; GO:0005634	
Pavir.4KG220405	Pavir.4KG220405.v5.1	Chr04K	13253104	13260816	(1 of 15) PF16719 - SAWADEE domain (SAWADEE)	SHH2_ARATH	Protein SAWADEE HOMEODOMAIN HOMOLOG 2 (Probable DNA-binding transcription factor 2)	SHH2 At3g18380 MYF24.10	Arabidopsis thaliana (Mouse-ear cress)	FUNCTION: May play a role in the recruitment of Pol IV to genomic regions associated with K9 methylated histone H3 that are targets for RdDM.	GO:0003677; GO:0003682; GO:0005634	
Pavir.4KG220410	Pavir.4KG220410.v5.1	Chr04K	13268160	13272774		#N/A	#N/A	#N/A	#N/A	#N/A	#N/A	#N/A
Pavir.4KG220415	Pavir.4KG220415.v5.1	Chr04K	13303478	13307516		#N/A	#N/A	#N/A	#N/A	#N/A	#N/A	#N/A
Pavir.4KG220420	Pavir.4KG220420.v5.1	Chr04K	13310297	13311143		#N/A	#N/A	#N/A	#N/A	#N/A	#N/A	#N/A
Pavir.4KG220425	Pavir.4KG220425.v5.1	Chr04K	13311274	13311642		#N/A	#N/A	#N/A	#N/A	#N/A	#N/A	#N/A
Pavir.4KG226900	Pavir.4KG226900.v5.1	Chr04K	13314180	13322589	(1 of 2827) 2.7.11.1 - Non-specific serine/threonine protein kinase / Threonine-specific protein kinase	WAK5_ARATH	Wall-associated receptor kinase 5 (EC 2.7.11.-)	WAK5 At1g21230 F16F4.9	Arabidopsis thaliana (Mouse-ear cress)	FUNCTION: Serine/threonine-protein kinase that may function as a signaling receptor of extracellular matrix component. Binding to pectin may have significance in the control of cell expansion, morphogenesis and development.	GO:0004674; GO:0005509; GO:0005524; GO:0005886; GO:0006468; GO:0007166; GO:0016021; GO:0030247	cell surface receptor signaling pathway [GO:0007166]; protein phosphorylation [GO:0006468]
Pavir.4KG226800	Pavir.4KG226800.v5.1	Chr04K	13325699	13329561	(1 of 2) PTHR27001//PTHR27001:SF36 - FAMILY NOT NAMED // PROLINE-RICH RECEPTOR-LIKE PROTEIN KINASE PERK12-RELATED	PEK12_ARATH	Proline-rich receptor-like protein kinase PERK12 (EC 2.7.11.1) (Proline-rich extensin-like receptor kinase 12) (AtPERK12) (Protein INFLORESCENCE GROWTH INHIBITOR 1)	PERK12 IGI1 At1g23540 F28C11.17 F5O8.10	Arabidopsis thaliana (Mouse-ear cress)	FUNCTION: Regulates the auxin-related MAX (More Axillary Growth) pathway during the shoot branching. {ECO:0000269|PubMed:20473553}.	GO:0004674; GO:0005524; GO:0005886; GO:0016021; GO:0032502	developmental process [GO:0032502]
Pavir.4KG226700	Pavir.4KG226700.v5.1	Chr04K	13330617	13343885	(1 of 6) K08835 - serine/threonine-protein kinase OSR1/STK39 [EC:2.7.11.1] (OXSR1, STK39)	FRAY2_DICDI	Serine/threonine-protein kinase fray2 (EC 2.7.11.1) (STE20-like kinase fray2)	fray2 DDB_G0229911	Dictyostelium discoideum (Slime mold)		GO:0004674; GO:0005524; GO:0046872	
Pavir.4KG226600	Pavir.4KG226600.v5.1	Chr04K	13346964	13353922		#N/A	#N/A	#N/A	#N/A	#N/A	#N/A	#N/A
Pavir.4KG226500	Pavir.4KG226500.v5.1	Chr04K	13371141	13374922	(1 of 2) 1.2.1.2 - Formate dehydrogenase	FDH1_ORYSJ	Formate dehydrogenase 1, mitochondrial (FDH 1) (EC 1.17.1.9) (NAD-dependent formate dehydrogenase 1)	Os06g0486800 LOC_Os06g29180 P0008F02.23 P0404G03.8	Oryza sativa subsp. japonica (Rice)	FUNCTION: Catalyzes the NAD(+)-dependent oxidation of formate to carbon dioxide. Involved in the cell stress response. {ECO:0000255|HAMAP-Rule:MF_03210}.	GO:0005739; GO:0008863; GO:0009326; GO:0009507; GO:0016616; GO:0042183; GO:0051287	formate catabolic process [GO:0042183]
Pavir.4KG226400	Pavir.4KG226400.v5.1	Chr04K	13376053	13379858		#N/A	#N/A	#N/A	#N/A	#N/A	#N/A	#N/A

Pavir.4KG226300	Pavir.4KG226300.v5.1	Chr04K	13380022	13394855	(1 of 1) KOG0778//KOG3246 - Protease, Ulp1 family // Sentrin-specific cysteine protease (Ulp1 family)	ULP1D_ARATH	Ubiquitin-like-specific protease 1D (EC 3.4.22.-) (Protein OVERLY TOLERANT TO SALT 1)	ULP1D OTS1 At1g60220 T13D8.11	Arabidopsis thaliana (Mouse-ear cress)	FUNCTION: Protease that catalyzes two essential functions in the SUMO pathway: processing of full-length SUMOs to their mature forms and deconjugation of SUMO from targeted proteins. Cleaves precursors of SUM1 and SUM2, but not of SUM3 or SUM5. Able to release SUM1 and SUM2 from conjugates, but unable to cleave SUM3. Protease activity mainly directed at deconjugating SUM1 and SUM2 from their target proteins. Regulates salt stress responses and flowering time. Redundant with ULP1C. {ECO:0000269|PubMed:16740136, ECO:0000269|PubMed:16920872, ECO:0000269|PubMed:18849491}.	GO:0005634; GO:0009651; GO:0010228; GO:0016607; GO:0016926; GO:0070139; GO:0070140	protein desumoylation [GO:0016926]; response to salt stress [GO:0009651]; vegetative to reproductive phase transition of meristem [GO:0010228]
Pavir.4KG226305	Pavir.4KG226305.v5.1	Chr04K	13454151	13461802		#N/A	#N/A	#N/A	#N/A	#N/A	#N/A	#N/A
Pavir.4KG226310	Pavir.4KG226310.v5.1	Chr04K	13461803	13463364	(1 of 23) PTHR11697//PTHR11697:SF137 - GENERAL TRANSCRIPTION FACTOR 2-RELATED ZINC FINGER PROTEIN // SUBFAMILY NOT NAMED	ZMYM1_HUMAN	Zinc finger MYM-type protein 1	ZMYM1	Homo sapiens (Human)		GO:0005634; GO:0008270; GO:0046983	#N/A
Pavir.4KG226315	Pavir.4KG226315.v5.1	Chr04K	13463699	13464392	(1 of 23) PTHR11697//PTHR11697:SF137 - GENERAL TRANSCRIPTION FACTOR 2-RELATED ZINC FINGER PROTEIN // SUBFAMILY NOT NAMED	#N/A	#N/A	#N/A	#N/A	#N/A	#N/A	#N/A
Pavir.4KG226100	Pavir.4KG226100.v5.1	Chr04K	13489440	13492919	(1 of 6) PTHR27001//PTHR27001:SF132 - FAMILY NOT NAMED // PROLINE-RICH RECEPTOR-LIKE PROTEIN KINASE PERK8	#N/A	#N/A	#N/A	#N/A	#N/A	#N/A	#N/A
Pavir.4KG226000	Pavir.4KG226000.v5.1	Chr04K	13493947	13495011		#N/A	#N/A	#N/A	#N/A	#N/A	#N/A	#N/A
Pavir.4KG225900	Pavir.4KG225900.v5.1	Chr04K	13495061	13502370	(1 of 2) PTHR24092//PTHR24092:SF41 - FAMILY NOT NAMED // PHOSPHOLIPID-TRANSPORTING ATPASE 10-RELATED	ALA9_ARATH	Putative phospholipid-transporting ATPase 9 (AtALA9) (EC 7.6.2.1) (Aminophospholipid flippase 9)	ALA9 At1g68710 F24J5.6	Arabidopsis thaliana (Mouse-ear cress)	FUNCTION: Involved in transport of phospholipids. {ECO:0000305|PubMed:11402198}.	GO:0000287; GO:0005524; GO:0005886; GO:0016021; GO:0045332; GO:0140326; GO:1901703	phospholipid translocation [GO:0045332]; protein localization involved in auxin polar transport [GO:1901703]
Pavir.4KG225800	Pavir.4KG225800.v5.1	Chr04K	13539108	13550846	(1 of 2) K13123 - G patch domain-containing protein 1 (GPATCH1)	TGHH_ORYSJ	G patch domain-containing protein TGH homolog (Protein TOUGH homolog)	Os06g0489200 LOC_Os06g29400 OsJ_21406 P0583E12.23	Oryza sativa subsp. japonica (Rice)	FUNCTION: Functions as component of microRNA (miRNA) and small interfering RNA (siRNA) biogenesis. May assist Dicer-like (DCL) proteins to efficiently process and/or recruit the precursors of miRNAs and siRNAs. {ECO:0000250|UniProtKB:Q8GXN9}.	GO:0003723; GO:0005634; GO:0006397; GO:0010087; GO:0030422; GO:0035196; GO:0040008; GO:0070878; GO:0070883	mRNA processing [GO:0006397]; phloem or xylem histogenesis [GO:0010087]; production of miRNAs involved in gene silencing by miRNA [GO:0035196]; production of siRNA involved in RNA interference [GO:0030422]; regulation of growth [GO:0040008]
Pavir.4KG225600	Pavir.4KG225600.v5.1	Chr04K	13581672	13590190	(1 of 2) K11991 - tRNA(adenine34) deaminase [EC:3.5.4.33] (tadA)	TADA_ARATH	tRNA(adenine(34)) deaminase, chloroplastic (TADA) (EC 3.5.4.33) (tRNA adenosine deaminase arginine) (tRNA arginine adenosine deaminase)	TADA At1g68720 F14K14.18 F24J5.5	Arabidopsis thaliana (Mouse-ear cress)	FUNCTION: Deaminates adenosines to inosines in tRNA-Arg(ACG). Exclusively involved in A-to-I editing of the prokaryote-type chloroplast-tRNA and not involved in C-to-U editing. {ECO:0000269|PubMed:19460869, ECO:0000269|PubMed:19602623}.	GO:0002100; GO:0005886; GO:0008251; GO:0009507; GO:0046872; GO:0052717; GO:0052718	tRNA wobble adenosine to inosine editing [GO:0002100]
Pavir.4KG225605	Pavir.4KG225605.v5.1	Chr04K	13587994	13588848		#N/A	#N/A	#N/A	#N/A	#N/A	#N/A	#N/A
Pavir.4KG225500	Pavir.4KG225500.v5.1	Chr04K	13590448	13593932		#N/A	#N/A	#N/A	#N/A	#N/A	#N/A	#N/A
Pavir.4KG225400	Pavir.4KG225400.v5.1	Chr04K	13601961	13607248	(1 of 2) PTHR10783//PTHR10783:SF49 - XENOTROPIC AND POLYTROPIC RETROVIRUS RECEPTOR 1-RELATED // SUBFAMILY NOT NAMED	PHO13_ORYSJ	Phosphate transporter PHO1-3 (Protein PHO1-3) (OsPHO1;3)	PHO1-3 Os06g0493600 LOC_Os06g29790 B1423D04.17	Oryza sativa subsp. japonica (Rice)	FUNCTION: May transport inorganic phosphate (Pi). {ECO:0000250}.	GO:0000822; GO:0005737; GO:0005794; GO:0005886; GO:0006817; GO:0015114; GO:0016021; GO:0016036	cellular response to phosphate starvation [GO:0016036]; phosphate ion transport [GO:0006817]
Pavir.4KG225300	Pavir.4KG225300.v5.1	Chr04K	13609312	13610135		#N/A	#N/A	#N/A	#N/A	#N/A	#N/A	#N/A
Pavir.4KG225100	Pavir.4KG225100.v5.1	Chr04K	13613592	13618409	(1 of 4) PTHR12346//PTHR12346:SF3 - SIN3B-RELATED // SUBFAMILY NOT NAMED	SNL4_ARATH	Paired amphipathic helix protein Sin3-like 4	SNL4 At1g70060 F20P5.21	Arabidopsis thaliana (Mouse-ear cress)	FUNCTION: Acts as a transcriptional repressor. Plays roles in regulating gene expression and genome stability (By similarity). {ECO:0000250}.	GO:0000118; GO:0000122; GO:0000785; GO:0003714; GO:0016575	histone deacetylation [GO:0016575]; negative regulation of transcription by RNA polymerase II [GO:0000122]
Pavir.4KG225000	Pavir.4KG225000.v5.1	Chr04K	13622934	13628938	(1 of 2) PTHR27002//PTHR27002:SF31 - FAMILY NOT NAMED // G-TYPE LECTIN S-RECEPTOR-LIKE SERINE/THREONINE-PROTEIN KINASE SD2-5	SD25_ARATH	G-type lectin S-receptor-like serine/threonine-protein kinase SD2-5 (EC 2.7.11.1) (S-domain-2 (SD2) receptor kinase 5) (SD2-5)	SD25 At4g32300 F10M6.60 F8B4.10	Arabidopsis thaliana (Mouse-ear cress)		GO:0004672; GO:0004674; GO:0005516; GO:0005524; GO:0005886; GO:0016021; GO:0030246; GO:0031625; GO:0046777	protein autophosphorylation [GO:0046777]
Pavir.4KG224900	Pavir.4KG224900.v5.1	Chr04K	13650883	13656023	(1 of 5) PTHR11206//PTHR11206:SF162 - MULTIDRUG RESISTANCE PROTEIN // SUBFAMILY NOT NAMED	DTX16_ARATH	Protein DETOXIFICATION 16 (AtDTX16) (Multidrug and toxic compound extrusion protein 16) (MATE protein 16)	DTX16 At5g52450 K24M7.20	Arabidopsis thaliana (Mouse-ear cress)		GO:0005774; GO:0009536; GO:0009624; GO:0015297; GO:0016020; GO:0016021; GO:0022857; GO:0042910	response to nematode [GO:0009624]
Pavir.4KG224800	Pavir.4KG224800.v5.1	Chr04K	13656716	13657945	(1 of 3) K10256 - omega-6 fatty acid desaturase (delta-12 desaturase) (FAD2)	DES2_SORBI	Fatty acid desaturase DES2 (SbDES2) (EC 1.14.19.-)	DES2 Sb04g029900 SORBIDRAFT_04g029900	Sorghum bicolor (Sorghum) (Sorghum vulgare)	FUNCTION: Delta(12) fatty acid desaturase converting palmitoleic acid (16:1(9)) to hexadecadienoic acid (16:2(9,12)). No detectable Delta(12)linoleate desaturase activity. Involved in the biosynthesis of the aliphatic side chain of sorgoleone, a phytotoxic secondary metabolite playing a direct role in allelopathic interactions. {ECO:0000269|PubMed:17178719}.	GO:0006636; GO:0016021; GO:0016717	unsaturated fatty acid biosynthetic process [GO:0006636]
Pavir.4KG224805	Pavir.4KG224805.v5.1	Chr04K	13678595	13679809	(1 of 27) PF04759 - Protein of unknown function, DUF617 (DUF617)	#N/A	#N/A	#N/A	#N/A	#N/A	#N/A	#N/A

Pavir.4KG224810	Pavir.4KG224810.v5.1	Chr04K	13693515	13699109		#N/A	#N/A	#N/A	#N/A	#N/A	#N/A	#N/A
Pavir.4KG224815	Pavir.4KG224815.v5.1	Chr04K	13730204	13730849		#N/A	#N/A	#N/A	#N/A	#N/A	#N/A	#N/A
Pavir.4KG223000	Pavir.4KG223000.v5.1	Chr04K	13731441	13735067	(1 of 23) PF07645//PF07714//PF13947 - Calcium-binding EGF domain (EGF_CA) // Protein tyrosine kinase (Pkinase_Tyr) // Wall-associated receptor kinase galacturonan-binding (GUB_WAK_bind)	WAK2_ARATH	Wall-associated receptor kinase 2 (EC 2.7.11.-)	WAK2 At1g21270 F16F4.5	Arabidopsis thaliana (Mouse-ear cress)	FUNCTION: Serine/threonine-protein kinase that may function as a signaling receptor of extracellular matrix component. Binding to pectin may have significance in the control of cell expansion, morphogenesis and development. {ECO:0000269|PubMed:11226187}.	GO:0004674; GO:0005509; GO:0005524; GO:0005634; GO:0005886; GO:0006468; GO:0007166; GO:0009311; GO:0009751; GO:0009826; GO:0009992; GO:0016021; GO:0030247	cell surface receptor signaling pathway [GO:0007166]; cellular water homeostasis [GO:0009992]; oligosaccharide metabolic process [GO:0009311]; protein phosphorylation [GO:0006468]; response to salicylic acid [GO:0009751]; unidimensional cell growth [GO:0009826]
Pavir.4KG222900	Pavir.4KG222900.v5.1	Chr04K	13746660	13754492	(1 of 4) PTHR13808//PTHR13808:SF7 - CBP/P300-RELATED // SUBFAMILY NOT NAMED	HACL3_ORYSJ	Probable histone acetyltransferase HAC-like 3 (EC 2.3.1.48)	Os01g0246100 LOC_Os01g14370 OSJNBa0004G10.16 OSJNBa0049B20.5	Oryza sativa subsp. japonica (Rice)	FUNCTION: Acetyltransferase enzyme. Acetylates histones, giving a specific tag for transcriptional activation. {ECO:0000250|UniProtKB:Q9C5X9}.	GO:0000123; GO:0001085; GO:0003713; GO:0004402; GO:0005667; GO:0008270; GO:0016573; GO:0031490; GO:0045944	histone acetylation [GO:0016573]; positive regulation of transcription by RNA polymerase II [GO:0045944]
Pavir.4KG222800	Pavir.4KG222800.v5.1	Chr04K	13765812	13777018	(1 of 1) PTHR13586//PTHR13586:SF0//PTHR13586:SF4 - SCD6 PROTEIN-RELATED // TRAILER HITCH, ISOFORM H // SUBFAMILY NOT NAMED	DCP5_ARATH	Protein decapping 5	DCP5 At1g26110 F14G11.8 F28B23.21	Arabidopsis thaliana (Mouse-ear cress)	FUNCTION: As a component of the decapping complex, involved in the degradation of mRNAs. Promotes P-body formation. Translational repressor. {ECO:0000269|PubMed:19855049}.	GO:0000932; GO:0003729; GO:0005634; GO:0005829; GO:0006397; GO:0010606; GO:0017148; GO:0031087; GO:0033962; GO:0034063; GO:0042803; GO:1990124	deadenylation-independent decapping of nuclear-transcribed mRNA [GO:0031087]; mRNA processing [GO:0006397]; negative regulation of translation [GO:0017148]; P-body assembly [GO:0033962]; positive regulation of cytoplasmic mRNA processing body assembly [GO:0010606]; stress granule assembly [GO:0034063]
Pavir.4KG222805	Pavir.4KG222805.v5.1	Chr04K	13782495	13783459		#N/A	#N/A	#N/A	#N/A	#N/A	#N/A	#N/A
Pavir.4KG222700	Pavir.4KG222700.v5.1	Chr04K	13792379	13794019	(1 of 7) PTHR13068//PTHR13068:SF40 - CGI-12 PROTEIN-RELATED // SUBFAMILY NOT NAMED	MTEFH_ARATH	Transcription termination factor MTEF18, mitochondrial (Mitochondrial transcription termination factor 18) (Protein SUPPRESSOR OF HOT1-4 1)	MTERF18 SHOT1 At3g60400 T8B10_60	Arabidopsis thaliana (Mouse-ear cress)	FUNCTION: Transcription termination factor involved in the regulation of mitochondrial-encoded gene expression. Essential for normal plant growth and development. {ECO:0000269|PubMed:22942382}.	GO:0003690; GO:0005739; GO:0006353; GO:0006355; GO:0007005; GO:0009507; GO:0009658; GO:0032502	chloroplast organization [GO:0009658]; developmental process [GO:0032502]; DNA-templated transcription, termination [GO:0006353]; mitochondrion organization [GO:0007005]; regulation of transcription, DNA-templated [GO:0006355]
Pavir.4KG222705	Pavir.4KG222705.v5.1	Chr04K	13802012	13802353		#N/A	#N/A	#N/A	#N/A	#N/A	#N/A	#N/A
Pavir.4KG222710	Pavir.4KG222710.v5.1	Chr04K	13809691	13810999	(1 of 181) PF12776 - Myb/SANT-like DNA-binding domain (Myb_DNA-bind_3)	LIMYB_ARATH	L10-interacting MYB domain-containing protein	LIMYB At5g05800 MJJ3.22	Arabidopsis thaliana (Mouse-ear cress)	FUNCTION: Transcriptional repressor that associates with ribosomal protein promoters. {ECO:0000269|PubMed:25707794}.	GO:0005634	
Pavir.4KG222500	Pavir.4KG222500.v5.1	Chr04K	13834523	13837020	(1 of 2827) 2.7.11.1 - Non-specific serine/threonine protein kinase / Threonine-specific protein kinase	CRK10_ARATH	Cysteine-rich receptor-like protein kinase 10 (Cysteine-rich RLK10) (EC 2.7.11.-) (Receptor-like protein kinase 4)	CRK10 RLK4 At4g23180 F21P8.70	Arabidopsis thaliana (Mouse-ear cress)		GO:0004674; GO:0005524; GO:0005829; GO:0005886; GO:0006468; GO:0016021; GO:0042742	defense response to bacterium [GO:0042742]; protein phosphorylation [GO:0006468]
Pavir.4KG222505	Pavir.4KG222505.v5.1	Chr04K	13839124	13839573		#N/A	#N/A	#N/A	#N/A	#N/A	#N/A	#N/A
Pavir.4KG222510	Pavir.4KG222510.v5.1	Chr04K	13843987	13844254		#N/A	#N/A	#N/A	#N/A	#N/A	#N/A	#N/A
Pavir.4KG222515	Pavir.4KG222515.v5.1	Chr04K	13852682	13856397		UGPI7_ARATH	Uncharacterized GPI-anchored protein At4g28100	At4g28100 T13J8.210	Arabidopsis thaliana (Mouse-ear cress)		GO:0005886; GO:0031225; GO:0046658	
Pavir.4KG222100	Pavir.4KG222100.v5.1	Chr04K	13858512	13859634		#N/A	#N/A	#N/A	#N/A	#N/A	#N/A	#N/A
Pavir.4KG222105	Pavir.4KG222105.v5.1	Chr04K	13877168	13878971	(1 of 598) PTHR19446//PTHR19446:SF355 - REVERSE TRANSCRIPTASES // SUBFAMILY NOT NAMED	#N/A	#N/A	#N/A	#N/A	#N/A	#N/A	#N/A
Pavir.4KG221700	Pavir.4KG221700.v5.1	Chr04K	13881698	13884109	(1 of 2) PTHR11362//PTHR11362:SF38 - PHOSPHATIDYLETHANOLAMINE-BINDING PROTEIN // PROTEIN MOTHER OF FT AND TF 1	#N/A	#N/A	#N/A	#N/A	#N/A	#N/A	#N/A
Pavir.4KG221500	Pavir.4KG221500.v5.1	Chr04K	13930821	13931362		PROA_ORYSJ	10 kDa prolamin	Os03g0766100 LOC_Os03g55730 OSJNBa0079B15	Oryza sativa subsp. japonica (Rice)	FUNCTION: Seed storage protein; serves as a source of nitrogen, carbon and sulfur for the young developing seedling.	GO:0005773; GO:0033095; GO:0045735	
Pavir.4KG221505	Pavir.4KG221505.v5.1	Chr04K	13932995	13933520	(1 of 63) PF01559 - Zein seed storage protein (Zein)	#N/A	#N/A	#N/A	#N/A	#N/A	#N/A	#N/A
Pavir.4KG221400	Pavir.4KG221400.v5.1	Chr04K	13942327	13943984		#N/A	#N/A	#N/A	#N/A	#N/A	#N/A	#N/A
Pavir.4KG221405	Pavir.4KG221405.v5.1	Chr04K	13948013	13948312		#N/A	#N/A	#N/A	#N/A	#N/A	#N/A	#N/A
Pavir.4KG221300	Pavir.4KG221300.v5.1	Chr04K	13948808	13964915	(1 of 31) PF12854//PF13041 - PPR repeat (PPR_1) // PPR repeat family (PPR_2)	PP327_ARATH	Pentatricopeptide repeat-containing protein At4g20090 (Protein EMBRYO DEFECTIVE 1025)	EMB1025 At4g20090 F18F4.190	Arabidopsis thaliana (Mouse-ear cress)	FUNCTION: May play a role in embryogenesis. {ECO:0000269|PubMed:15647901}.	GO:0009793	embryo development ending in seed dormancy [GO:0009793]
Pavir.4KG221410	Pavir.4KG221410.v5.1	Chr04K	13950060	13950773		#N/A	#N/A	#N/A	#N/A	#N/A	#N/A	#N/A
Pavir.4KG221200	Pavir.4KG221200.v5.1	Chr04K	13968326	13970287	(1 of 4) PTHR22814//PTHR22814:SF121 - COPPER TRANSPORT PROTEIN ATOX1-RELATED // SUBFAMILY NOT NAMED	#N/A	#N/A	#N/A	#N/A	#N/A	#N/A	#N/A
Pavir.4KG221205	Pavir.4KG221205.v5.1	Chr04K	13972389	13973343		#N/A	#N/A	#N/A	#N/A	#N/A	#N/A	#N/A

Pavir.4KG219900	Pavir.4KG219900.v5.1	Chr04K	13998875	14000331	(1 of 57) 1.14.13.121 - Premnaspirodiene oxygenase / Hyoscymus muticus premnaspirodiene oxygenase	C71Z6_MAIZE	Dolabradiene monooxygenase (EC 1.14.14.159) (Cytochrome P450 71Z16) (ZmCYP71Z16)	CYP71Z16 ZEAMMB73_Zm00001d014136	Zea mays (Maize)	FUNCTION: Involved in the production of antifungal dolabralexin phytoalexins in response to biotic and abiotic stresses (PubMed:29475898). Catalyzes the epoxidation of dolabradiene at C-16, followed by hydroxylation at C-3, to yield the epoxides 15,16-epoxydolabrene (epoxydolabrene) and 3b-hydroxy-15,16-epoxydolabrene (epoxydolabranol) (PubMed:29475898). {ECO:0000269|PubMed:29475898}.	GO:0005506; GO:0006952; GO:0010333; GO:0016021; GO:0016709; GO:0020037; GO:0051502	defense response [GO:0006952]; diterpene phytoalexin biosynthetic process [GO:0051502]
Pavir.4KG219905	Pavir.4KG219905.v5.1	Chr04K	14005895	14006215	(1 of 6) PTHR31973:SF5 - AMINOTRANSFERASE-LIKE, PLANT MOBILE DOMAIN FAMILY PROTEIN-RELATED	#N/A	#N/A	#N/A	#N/A	#N/A	#N/A	#N/A
Pavir.4KG219500	Pavir.4KG219500.v5.1	Chr04K	14027861	14028860		#N/A	#N/A	#N/A	#N/A	#N/A	#N/A	#N/A
Pavir.4KG219400	Pavir.4KG219400.v5.1	Chr04K	14035569	14036524		#N/A	#N/A	#N/A	#N/A	#N/A	#N/A	#N/A
Pavir.4KG127700	Pavir.4KG127700.v5.1	Chr04K	14050861	14051545		#N/A	#N/A	#N/A	#N/A	#N/A	#N/A	#N/A
Pavir.4KG219300	Pavir.4KG219300.v5.1	Chr04K	14067005	14071211	(1 of 4) PTHR21461:SF12 - F21O3.9 PROTEIN-RELATED	GALS1_ARATH	Galactan beta-1,4-galactosyltransferase GALS1 (EC 2.4.1.-) (Beta-1,4-galactan synthase) (Galactan synthase 1)	GALS1 At2g33570	Arabidopsis thaliana (Mouse-ear cress)	FUNCTION: Involved in the biosynthesis of beta-1,4-galactan. Can transfer galactose residues from UDP-galactose to beta-1,4-galactopentaose in vitro. Forms specifically beta-1,4-galactosyl linkages and can add successive beta-1,4-galactosyl residues to the acceptor. Beta-1,4-galactans are abundant polysaccharides in plant cell walls and are found as side-chain of rhamnogalacturonan I, which is a major component of pectin. {ECO:0000269|PubMed:23243126}.	GO:0000139; GO:0003831; GO:0005794; GO:0016021; GO:0042546; GO:0045489; GO:0071555	cell wall biogenesis [GO:0042546]; cell wall organization [GO:0071555]; pectin biosynthetic process [GO:0045489]
Pavir.4KG219305	Pavir.4KG219305.v5.1	Chr04K	14080378	14080954		#N/A	#N/A	#N/A	#N/A	#N/A	#N/A	#N/A
Pavir.4KG219310	Pavir.4KG219310.v5.1	Chr04K	14096492	14098248	(1 of 20) PTHR23024//PTHR23024:SF225 - MEMBER OF 'GDXG' FAMILY OF LIPOLYTIC ENZYMES // SUBFAMILY NOT NAMED	CXE12_ARATH	Probable carboxylesterase 12 (AtCXE12) (EC 3.1.1.1)	CXE12 At3g48690 T8P19.200	Arabidopsis thaliana (Mouse-ear cress)	FUNCTION: Carboxylesterase acting on esters with varying acyl chain length. {ECO:0000250}.	GO:0005737; GO:0005829; GO:0016787; GO:0052689; GO:0080030	
Pavir.4KG219315	Pavir.4KG219315.v5.1	Chr04K	14121537	14126174	(1 of 2) KOG0404 - Thioredoxin reductase	NTRA_ORYSJ	Thioredoxin reductase NTRA (EC 1.8.1.9) (NADPH-dependent thioredoxin reductase A) (OsNTRA)	Os06g0327300 LOC_Os06g22140 OSJNBb0039F24.13	Oryza sativa subsp. japonica (Rice)	FUNCTION: Possesses thioredoxin-disulfide reductase activity. {ECO:0000250}.	GO:0004791; GO:0005737; GO:0019430	removal of superoxide radicals [GO:0019430]
Pavir.4KG120500	Pavir.4KG120500.v5.1	Chr04K	14144616	14145981	(1 of 4) PTHR31100//PTHR31100:SF5 - FAMILY NOT NAMED // AT-HOOK MOTIF NUCLEAR LOCALIZED PROTEIN 29-RELATED	AHL29_ARATH	AT-hook motif nuclear-localized protein 29 (Protein SUPPRESSOR OF PHYB-4#3)	AHL29 SOB3 At1g76500 F14G6.10	Arabidopsis thaliana (Mouse-ear cress)	FUNCTION: Transcription factor that specifically binds AT-rich DNA sequences related to the nuclear matrix attachment regions (MARs) (By similarity). Acts redundantly with AHL18, AHL22 and AHL27 in the regulation of flowering and regulation of the hypocotyl elongation (PubMed:19517252). Acts redundantly with AHL27/ESC to modulate hypocotyl growth inhibition in response to light (PubMed:18088311). {ECO:0000250|UniProtKB:Q8VYJ2, ECO:0000269|PubMed:18088311, ECO:0000269|PubMed:19517252}.	GO:0003680; GO:0003700; GO:0005634; GO:0009640; GO:0009908; GO:0010228; GO:0043621	flower development [GO:0009908]; photomorphogenesis [GO:0009640]; vegetative to reproductive phase transition of meristem [GO:0010228]
Pavir.4KG120400	Pavir.4KG120400.v5.1	Chr04K	14160085	14163066	(1 of 3) K14457 - 2-acylglycerol O-acyltransferase 2 (MOGAT2, MGAT2)	DAT2D_SOYBN	Diacylglycerol O-acyltransferase 2D (GmDGAT2D) (EC 2.3.1.20)	DGAT2D Glyma01g156000	Glycine max (Soybean) (Glycine hispida)	FUNCTION: Involved in triacylglycerol (TAG) synthesis. Catalyzes the acylation of the sn-3 hydroxy group of sn-1,2-diacylglycerol using acyl-CoA. Can use oleoyl-CoA and linoleoyl-CoA as substrates. May play a role in TAG biosynthesis in different tissues in responses to environmental and hormonal cues. {ECO:0000269|PubMed:27345221}.	GO:0004144; GO:0005789; GO:0005811; GO:0006071; GO:0006629; GO:0008374; GO:0016021; GO:0019432	glycerol metabolic process [GO:0006071]; lipid metabolic process [GO:0006629]; triglyceride biosynthetic process [GO:0019432]
Pavir.4KG120200	Pavir.4KG120200.v5.1	Chr04K	14167762	14178475	(1 of 1) PF00365//PF02330 - Phosphofructokinase (PFK) // Mitochondrial glycoprotein (MAM33)	PFPA_RICCO	Pyrophosphate--fructose 6-phosphate 1-phosphotransferase subunit alpha (PFP) (6-phosphofructokinase, pyrophosphate dependent) (PPi-PFK) (Pyrophosphate-dependent 6-phosphofructose-1-kinase)	PFP-ALPHA	Ricinus communis (Castor bean)	FUNCTION: Regulatory subunit of pyrophosphate--fructose 6-phosphate 1-phosphotransferase. {ECO:0000255|HAMAP-Rule:MF_03185}.	GO:0003872; GO:0005524; GO:0005737; GO:0046872; GO:0047334	
Pavir.4KG120100	Pavir.4KG120100.v5.1	Chr04K	14187794	14188353		#N/A	#N/A	#N/A	#N/A	#N/A	#N/A	#N/A
Pavir.4KG119800	Pavir.4KG119800.v5.1	Chr04K	14193118	14193954	(1 of 1) 1.1.1.247 - Codeinone reductase (NADPH)	#N/A	#N/A	#N/A	#N/A	#N/A	#N/A	#N/A
Pavir.4KG119805	Pavir.4KG119805.v5.1	Chr04K	14196974	14199635	(1 of 28) PF05699//PF14372 - hAT family C-terminal dimerisation region (Dimer_Tnp_hAT) // Domain of unknown function (DUF4413) (DUF4413)	RSLE1_ORYSJ	Zinc finger BED domain-containing protein RICESLEEPER 1 (Transposase-like protein RICESLEEPER 1)	Os05g0239150 LOC_Os05g14940 OJ1122_B08 OsJ_17746	Oryza sativa subsp. japonica (Rice)	FUNCTION: Transposase-like protein that is essential for plant growth and development. May regulate global gene expression by recruiting other cellular factors. {ECO:0000269|PubMed:23067104}.	GO:0003677; GO:0005634; GO:0009791; GO:0046872; GO:0046983	post-embryonic development [GO:0009791]
Pavir.4KG119810	Pavir.4KG119810.v5.1	Chr04K	14202436	14202936	(1 of 208) PF03101 - FAR1 DNA-binding domain (FAR1)	#N/A	#N/A	#N/A	#N/A	#N/A	#N/A	#N/A
Pavir.4KG119815	Pavir.4KG119815.v5.1	Chr04K	14205414	14206409		#N/A	#N/A	#N/A	#N/A	#N/A	#N/A	#N/A
Pavir.4KG119820	Pavir.4KG119820.v5.1	Chr04K	14206506	14206877		#N/A	#N/A	#N/A	#N/A	#N/A	#N/A	#N/A
Pavir.4KG119200	Pavir.4KG119200.v5.1	Chr04K	14208612	14209137		#N/A	#N/A	#N/A	#N/A	#N/A	#N/A	#N/A
Pavir.4KG119205	Pavir.4KG119205.v5.1	Chr04K	14215701	14216967		#N/A	#N/A	#N/A	#N/A	#N/A	#N/A	#N/A
Pavir.4KG119000	Pavir.4KG119000.v5.1	Chr04K	14216923	14218915	(1 of 117) PF07762 - Protein of unknown function (DUF1618) (DUF1618)	#N/A	#N/A	#N/A	#N/A	#N/A	#N/A	#N/A
Pavir.4KG159800	Pavir.4KG159800.v5.1	Chr04K	14228545	14229560		#N/A	#N/A	#N/A	#N/A	#N/A	#N/A	#N/A
Pavir.4KG118700	Pavir.4KG118700.v5.1	Chr04K	14234350	14235449		#N/A	#N/A	#N/A	#N/A	#N/A	#N/A	#N/A

Pavir.4KG118600	Pavir.4KG118600.v5.1	Chr04K	14245580	14249971	(1 of 55) PF03479 - Domain of unknown function (DUF296) (DUF296)	AHL10_ARATH	AT-hook motif nuclear-localized protein 10 (AT-hook protein 1)	AHL10 AHP1 At2g33620 F4P9.39	Arabidopsis thaliana (Mouse-ear cress)	FUNCTION: Transcription factor that specifically binds AT-rich DNA sequences related to the nuclear matrix attachment regions (MARs). {ECO:0000250|UniProtKB:Q8VYJ2}.	GO:0003680; GO:0005634; GO:0005829	
Pavir.4KG118500	Pavir.4KG118500.v5.1	Chr04K	14249876	14256807	(1 of 3) PTHR12280//PTHR12280:SF31 - PANTOTHENATE KINASE // SUBFAMILY NOT NAMED	D89S2_ARATH	Damage-control phosphatase At2g17340 (EC 3.1.3.-) (Sugar phosphates phosphatase At2g17340)	At2g17340 F5J6.10	Arabidopsis thaliana (Mouse-ear cress)	FUNCTION: Metal-dependent phosphatase with probable damage-control functions (PubMed:27322068). Shows phosphatase activity against several substrates, including sugar phosphates and p-nitrophenyl phosphate(pNPP) (PubMed:27322068). Prefers sugar phosphate substrates, including the extremely potent glycating agents ribose-5-phosphate and erythrose-4-phosphate (PubMed:27322068). {ECO:0000269|PubMed:27322068}.	GO:0004594; GO:0005634; GO:0005739; GO:0005829; GO:0015937; GO:0016787; GO:0046872	coenzyme A biosynthetic process [GO:0015937]
Pavir.4KG118400	Pavir.4KG118400.v5.1	Chr04K	14257092	14257837		#N/A	#N/A	#N/A	#N/A	#N/A	#N/A	#N/A
Pavir.4KG118300	Pavir.4KG118300.v5.1	Chr04K	14277572	14281706	(1 of 2) PTHR24064//PTHR24064:SF210 - FAMILY NOT NAMED // INORGANIC PHOSPHATE TRANSPORTER 1-8-RELATED	PT110_ORYSJ	Probable inorganic phosphate transporter 1-10 (OsPT10) (OsPht1;10) (H(+)/Pi cotransporter)	PHT1-10 PT10 Os06g0325200 LOC_Os06g21950 OsJ_020312 P0421H01.35 P0652D10.5	Oryza sativa subsp. japonica (Rice)	FUNCTION: High-affinity transporter for external inorganic phosphate. {ECO:0000250}.	GO:0006817; GO:0015293; GO:0016021	phosphate ion transport [GO:0006817]
Pavir.4KG118200	Pavir.4KG118200.v5.1	Chr04K	14286645	14287871	(1 of 119) PF13912 - C2H2-type zinc finger (zf-C2H2_6)	#N/A	#N/A	#N/A	#N/A	#N/A	#N/A	#N/A
Pavir.4KG118100	Pavir.4KG118100.v5.1	Chr04K	14302958	14303694		#N/A	#N/A	#N/A	#N/A	#N/A	#N/A	#N/A
Pavir.4KG118105	Pavir.4KG118105.v5.1	Chr04K	14356186	14370441	(1 of 7) PTHR11654//PTHR11654:SF90 - OLIGOPEPTIDE TRANSPORTER-RELATED // PROTEIN NRT1/ PTR FAMILY 7.2	PTR14_ARATH	Protein NRT1/ PTR FAMILY 7.3 (AtNPF7.3) (Nitrate transporter 1.5)	NPF7.3 NRT1.5 At1g32450 F5D14.23	Arabidopsis thaliana (Mouse-ear cress)	FUNCTION: Low-affinity proton-dependent bidirectional nitrate transporter. Involved in nitrate loading into xylem and not in nitrate uptake. Not involved in histidine or dipeptides transport. {ECO:0000269|PubMed:18780802}.	GO:0005886; GO:0010150; GO:0010167; GO:0015112; GO:0015293; GO:0015386; GO:0015706; GO:0016021; GO:0055075; GO:1902600	leaf senescence [GO:0010150]; nitrate transport [GO:0015706]; potassium ion homeostasis [GO:0055075]; proton transmembrane transport [GO:1902600]; response to nitrate [GO:0010167]
Pavir.4KG118110	Pavir.4KG118110.v5.1	Chr04K	14358557	14362640		#N/A	#N/A	#N/A	#N/A	#N/A	#N/A	#N/A
Pavir.4KG117200	Pavir.4KG117200.v5.1	Chr04K	14382479	14383730	(1 of 7) PTHR10426//PTHR10426:SF34 - STRICTOSIDINE SYNTHASE-RELATED // SUBFAMILY NOT NAMED	SSL10_ARATH	Protein STRICTOSIDINE SYNTHASE-LIKE 10 (AtSSL10) (Strictosidine synthase 8) (AtSS8)	SSL10 SS8 At3g57030 F24I3.110	Arabidopsis thaliana (Mouse-ear cress)		GO:0005773; GO:0005783; GO:0005829; GO:0005886; GO:0009058; GO:0009505; GO:0009506	biosynthetic process [GO:0009058]
Pavir.4KG117000	Pavir.4KG117000.v5.1	Chr04K	14393598	14394091	(1 of 10) PF03760 - Late embryogenesis abundant (LEA) group 1 (LEA_1)	#N/A	#N/A	#N/A	#N/A	#N/A	#N/A	#N/A
Pavir.4KG116900	Pavir.4KG116900.v5.1	Chr04K	14396314	14397378		#N/A	#N/A	#N/A	#N/A	#N/A	#N/A	#N/A
Pavir.4KG116905	Pavir.4KG116905.v5.1	Chr04K	14422803	14423633	(1 of 3) PTHR31234//PTHR31234:SF12 - FAMILY NOT NAMED // LATE EMBRYOGENESIS ABUNDANT HYDROXYPROLINE-RICH GLYCOPROTEIN	#N/A	#N/A	#N/A	#N/A	#N/A	#N/A	#N/A
Pavir.4KG147100	Pavir.4KG147100.v5.1	Chr04K	14423925	14424751		#N/A	#N/A	#N/A	#N/A	#N/A	#N/A	#N/A
Pavir.4KG147105	Pavir.4KG147105.v5.1	Chr04K	14428803	14429444	(1 of 56) PTHR23155:SF455 - COILED-COIL DOMAIN-CONTAINING PROTEIN LOBO HOMOLOG	RGA2_SOLBU	Disease resistance protein RGA2 (Blight resistance protein RPI) (RGA2-blb)	RGA2 177O13.40 CB3A14.5 RB RPI-BLB1	Solanum bulbocastanum (Wild potato)	FUNCTION: Disease resistance protein. Resistance proteins guard the plant against pathogens that contain an appropriate avirulence protein via a direct or indirect interaction with this avirulence protein. That triggers a defense system which restricts the pathogen growth. Confers a broad resistance to all known races of P.infestans.	GO:0005524; GO:0006952; GO:0043531	defense response [GO:0006952]
Pavir.4KG147000	Pavir.4KG147000.v5.1	Chr04K	14429782	14433823	(1 of 3) KOG1947//KOG4658 - Leucine rich repeat proteins, some proteins contain F-box // Apoptotic ATPase	#N/A	#N/A	#N/A	#N/A	#N/A	#N/A	#N/A
Pavir.4KG147005	Pavir.4KG147005.v5.1	Chr04K	14438399	14439112		#N/A	#N/A	#N/A	#N/A	#N/A	#N/A	#N/A
Pavir.4KG147010	Pavir.4KG147010.v5.1	Chr04K	14440488	14441429		#N/A	#N/A	#N/A	#N/A	#N/A	#N/A	#N/A
Pavir.4KG147015	Pavir.4KG147015.v5.1	Chr04K	14460481	14460792		#N/A	#N/A	#N/A	#N/A	#N/A	#N/A	#N/A
Pavir.4KG143200	Pavir.4KG143200.v5.1	Chr04K	14466202	14470882	(1 of 2) KOG2542 - Uncharacterized conserved protein (YdiU family)	#N/A	#N/A	#N/A	#N/A	#N/A	#N/A	#N/A
Pavir.4KG143300	Pavir.4KG143300.v5.1	Chr04K	14471205	14472710	(1 of 2) K08907 - light-harvesting complex I chlorophyll a/b binding protein 1 (LHCA1)	CB121_HORVU	Chlorophyll a-b binding protein 1B-21, chloroplastic (LHCI type I CAB-1B-21) (LHCI-730 chlorophyll a/b binding protein) (Light-harvesting complex I 21 kDa protein)	LHC Ib-21 LHCA1	Hordeum vulgare (Barley)	FUNCTION: The light-harvesting complex (LHC) functions as a light receptor, it captures and delivers excitation energy to photosystems with which it is closely associated. {ECO:0000250}.	GO:0009522; GO:0009523; GO:0009535; GO:0009765; GO:0016021; GO:0016168; GO:0018298; GO:0046872	photosynthesis, light harvesting [GO:0009765]; protein-chromophore linkage [GO:0018298]
Pavir.4KG143400	Pavir.4KG143400.v5.1	Chr04K	14482352	14484097		#N/A	#N/A	#N/A	#N/A	#N/A	#N/A	#N/A
Pavir.4KG143000	Pavir.4KG143000.v5.1	Chr04K	14489667	14494551	(1 of 2) PTHR10353//PTHR10353:SF57 - GLYCOSYL HYDROLASE // SUBFAMILY NOT NAMED	BGL24_ORYSJ	Beta-glucosidase 24 (Os6bglu24) (EC 3.2.1.21)	BGLU24 Os06g0320200 LOC_Os06g21570 OsJ_21154 P0592B08.35	Oryza sativa subsp. japonica (Rice)		GO:0005975; GO:0008422; GO:0102483	carbohydrate metabolic process [GO:0005975]
Pavir.4KG143600	Pavir.4KG143600.v5.1	Chr04K	14494796	14498829	(1 of 2) KOG2607 - CDK5 activator-binding protein	CK5P3_ARATH	CDK5RAP3-like protein	At5g06830 MPH15.20	Arabidopsis thaliana (Mouse-ear cress)	FUNCTION: Potential regulator of CDK5 activity. {ECO:0000250}.	GO:0000079; GO:0007346	regulation of cyclin-dependent protein serine/threonine kinase activity [GO:0000079]; regulation of mitotic cell cycle [GO:0007346]
Pavir.4KG143700	Pavir.4KG143700.v5.1	Chr04K	14500889	14502212	(1 of 2) KOG3425 - Uncharacterized conserved protein	#N/A	#N/A	#N/A	#N/A	#N/A	#N/A	#N/A

Pavir.4KG143705	Pavir.4KG143705.v5.1	Chr04K	14531073	14531759	(1 of 50) PF00280 - Potato inhibitor I family (potato_inhibit)	ICI1_SOLPE	Wound-induced proteinase inhibitor 1 (Wound-induced proteinase inhibitor I)		Solanum peruvianum (Peruvian tomato) (Lycopersicon peruvianum)		GO:0004867; GO:0005576; GO:0009611	response to wounding [GO:0009611]
Pavir.4KG144000	Pavir.4KG144000.v5.1	Chr04K	14533176	14534109	(1 of 50) PF00280 - Potato inhibitor I family (potato_inhibit)	ICI1_SOLPE	Wound-induced proteinase inhibitor 1 (Wound-induced proteinase inhibitor I)		Solanum peruvianum (Peruvian tomato) (Lycopersicon peruvianum)		GO:0004867; GO:0005576; GO:0009611	response to wounding [GO:0009611]
Pavir.4KG144100	Pavir.4KG144100.v5.1	Chr04K	14534390	14536961	(1 of 8) K02910 - large subunit ribosomal protein L31e (RP-L31e, RPL31)	RL31_PERFR	60S ribosomal protein L31	RPL31	Perilla frutescens (Beefsteak mint) (Perilla ocymoides)		GO:0003735; GO:0005840; GO:0006412	translation [GO:0006412]
Pavir.4KG144200	Pavir.4KG144200.v5.1	Chr04K	14541319	14541492		#N/A	#N/A	#N/A	#N/A	#N/A	#N/A	#N/A
Pavir.4KG144205	Pavir.4KG144205.v5.1	Chr04K	14576968	14580484	(1 of 2) PF02902//PF10536 - Ulp1 protease family, C-terminal catalytic domain (Peptidase_C48) // Plant mobile domain (PMD)	#N/A	#N/A	#N/A	#N/A	#N/A	#N/A	#N/A
Pavir.4KG144210	Pavir.4KG144210.v5.1	Chr04K	14626080	14626289		#N/A	#N/A	#N/A	#N/A	#N/A	#N/A	#N/A
Pavir.4KG144215	Pavir.4KG144215.v5.1	Chr04K	14662924	14666407		#N/A	#N/A	#N/A	#N/A	#N/A	#N/A	#N/A
Pavir.4KG114900	Pavir.4KG114900.v5.1	Chr04K	14698305	14717852	(1 of 6) 5.4.2.2//5.4.2.8 - Phosphoglucomutase (alpha-D-glucose-1,6-bisphosphate-dependent) / Phosphoglucose mutase // Phosphomannomutase / Phosphomannose mutase	ALGC_PSEAE	Phosphomannomutase/phosphoglucomutase (PMM / PGM) (EC 5.4.2.2) (EC 5.4.2.8)	algC PA5322	Pseudomonas aeruginosa (strain ATCC 15692 / DSM 22644 / CIP 104116 / JCM 14847 / LMG 12228 / 1C / PRS 101 / PAO1)	FUNCTION: Highly reversible phosphoryltransferase. The phosphomannomutase activity produces a precursor for alginate polymerization, the alginate layer causes a mucoid phenotype and provides a protective barrier against host immune defenses and antibiotics. Also involved in core lipopolysaccaride (LPS) biosynthesis due to its phosphoglucomutase activity. Essential for rhamnolipid production, an exoproduct correlated with pathogenicity (PubMed:10481091). Required for biofilm production. The reaction proceeds via 2 processive phosphoryl transferase reactions; first from enzyme-phospho-Ser-108 to the substrate (generating a bisphosphorylated substrate intermediate and a dephosphorylated enzyme), a 180 degree rotation of the intermediate (probably aided by movement of domain 4), and subsequent transfer of phosphate back to the enzyme (PubMed:11716469, PubMed:16880541, PubMed:16595672, PubMed:22242625). {ECO:0000269|PubMed:10481091, ECO:0000269|PubMed:11716469, ECO:0000269|PubMed:16595672, ECO:0000269|PubMed:16880541, ECO:0000269|PubMed:18690721, ECO:0000269|PubMed:1903398, ECO:0000269|PubMed:22242625, ECO:0000269|PubMed:23517223, ECO:0000269|PubMed:7515870, ECO:0000269|PubMed:8050998}.	GO:0000287; GO:0004614; GO:0004615; GO:0009243; GO:0009244; GO:0009298; GO:0009405; GO:0042121	alginic acid biosynthetic process [GO:0042121]; GDP-mannose biosynthetic process [GO:0009298]; lipopolysaccharide core region biosynthetic process [GO:0009244]; O antigen biosynthetic process [GO:0009243]; pathogenesis [GO:0009405]
Pavir.4KG114905	Pavir.4KG114905.v5.1	Chr04K	14726649	14730824		#N/A	#N/A	#N/A	#N/A	#N/A	#N/A	#N/A
Pavir.4KG115600	Pavir.4KG115600.v5.1	Chr04K	14777279	14783003	(1 of 3) K06125 - 4-hydroxybenzoate polyprenyltransferase (COQ2)	COQ2_ARATH	4-hydroxybenzoate polyprenyltransferase, mitochondrial (4-HB polyprenyltransferase) (4HPT) (EC 2.5.1.39) (4-hydroxybenzoate nonaprenyltransferase) (Para-hydroxybenzoate--polyprenyltransferase) (PHB:PPT) (PHB:polyprenyltransferase) (Polyprenyltransferase 1) (AtPPT1)	PPT1 At4g23660 F9D16.130	Arabidopsis thaliana (Mouse-ear cress)	FUNCTION: Catalyzes the prenylation of para-hydroxybenzoate (PHB) with an all-trans polyprenyl group. Mediates the second step in the final reaction sequence of coenzyme Q (CoQ) biosynthesis, which is the condensation of the polyisoprenoid side chain with PHB, generating the first membrane-bound Q intermediate. Required for embryo development. {ECO:0000255|HAMAP-Rule:MF_03189, ECO:0000269|PubMed:15604701}.	GO:0002083; GO:0005739; GO:0006744; GO:0008299; GO:0016765; GO:0031305; GO:0047293	isoprenoid biosynthetic process [GO:0008299]; ubiquinone biosynthetic process [GO:0006744]
Pavir.4KG115605	Pavir.4KG115605.v5.1	Chr04K	14787255	14787692		#N/A	#N/A	#N/A	#N/A	#N/A	#N/A	#N/A
Pavir.4KG115700	Pavir.4KG115700.v5.1	Chr04K	14788092	14793562	(1 of 4) K15687 - E3 ubiquitin-protein ligase makorin [EC:6.3.2.19] (MKRN)	C3H41_ORYSJ	E3 ubiquitin-protein ligase makorin (EC 2.3.2.27) (RING-type E3 ubiquitin transferase makorin) (Zinc finger CCCH domain-containing protein 41) (OsC3H41)	MKRN Os06g0318700 LOC_Os06g21390 OsJ_21146 P0468A12.33 P0592B08.7	Oryza sativa subsp. japonica (Rice)	FUNCTION: E3 ubiquitin ligase catalyzing the covalent attachment of ubiquitin moieties onto substrate proteins. {ECO:0000250}.	GO:0003677; GO:0016567; GO:0046872; GO:0061630	protein ubiquitination [GO:0016567]
Pavir.4KG115805	Pavir.4KG115805.v5.1	Chr04K	14795562	14798749	(1 of 598) PTHR19446//PTHR19446:SF355 - REVERSE TRANSCRIPTASES // SUBFAMILY NOT NAMED	#N/A	#N/A	#N/A	#N/A	#N/A	#N/A	#N/A

Pavir.4KG115800	Pavir.4KG115800.v5.1	Chr04K	14795562	14803601	(1 of 2) PTHR32060//PTHR32060:SF5 - FAMILY NOT NAMED // PEPTIDASE S41 FAMILY PROTEIN	CTPA3_ARATH	Carboxyl-terminal-processing peptidase 3, chloroplastic (EC 3.4.21.102) (D1 C-terminal processing protease 3) (Photosystem II D1 protein processing peptidase 3)	CTPA3 At3g57680 F15B8.130	Arabidopsis thaliana (Mouse-ear cress)	FUNCTION: Protease involved in the C-terminal processing of the chloroplastic D1 protein of photosystem II. This proteolytic processing is necessary to allow the light-driven assembly of the tetranuclear manganese cluster, which is responsible for photosynthetic water oxidation. {ECO:0000250|UniProtKB:O04073}.	GO:0004175; GO:0004252; GO:0009543	
Pavir.4KG115810	Pavir.4KG115810.v5.1	Chr04K	14798938	14799762	(1 of 209) 2.7.7.49 - RNA-directed DNA polymerase / Revertase	#N/A	#N/A	#N/A	#N/A	#N/A	#N/A	#N/A
Pavir.4KG115815	Pavir.4KG115815.v5.1	Chr04K	14804282	14806767		#N/A	#N/A	#N/A	#N/A	#N/A	#N/A	#N/A
Pavir.4KG228900	Pavir.4KG228900.v5.1	Chr04K	14810695	14814077	(1 of 3) K13220 - WW domain-binding protein 4 (WBP4, FBP21)	ZOP1_ARATH	Zinc finger protein ZOP1 (Zinc-finger and OCRE domain-containing protein 1)	ZOP1 At1g49590 F14J22.17	Arabidopsis thaliana (Mouse-ear cress)	FUNCTION: Nucleic acid-binding protein that promotes Pol IV-dependent small interfering RNA (siRNA) accumulation, DNA methylation and transcriptional silencing. May possess both RNA-directed DNA methylation (RdDM)-dependent and -independent roles in transcriptional silencing. Acts as a pre-mRNA splicing factor that associates with several typical components of the splicing machinery as well as with Pol II. {ECO:0000269|PubMed:23524848}.	GO:0000398; GO:0003690; GO:0003723; GO:0003725; GO:0008270; GO:0008380; GO:0009845; GO:0015030; GO:0071011; GO:0080188	gene silencing by RNA-directed DNA methylation [GO:0080188]; mRNA splicing, via spliceosome [GO:0000398]; RNA splicing [GO:0008380]; seed germination [GO:0009845]
Pavir.4KG228905	Pavir.4KG228905.v5.1	Chr04K	14815633	14821678	(1 of 9) 3.4.22.40 - Bleomycin hydrolase / Aminopeptidase C (Lactococcus lactis)	#N/A	#N/A	#N/A	#N/A	#N/A	#N/A	#N/A
Pavir.4KG114100	Pavir.4KG114100.v5.1	Chr04K	14851088	14857912	(1 of 9) 3.4.22.40 - Bleomycin hydrolase / Aminopeptidase C (Lactococcus lactis)	#N/A	#N/A	#N/A	#N/A	#N/A	#N/A	#N/A
Pavir.4KG114200	Pavir.4KG114200.v5.1	Chr04K	14869166	14872586	(1 of 2) PTHR10110//PTHR10110:SF101 - SODIUM/HYDROGEN EXCHANGER // SODIUM/HYDROGEN EXCHANGER 4	NHX4_ARATH	Sodium/hydrogen exchanger 4 (Na(+)/H(+) exchanger 4) (NHE-4)	NHX4 At5g55470 MTE17.18	Arabidopsis thaliana (Mouse-ear cress)	FUNCTION: May act in low affinity electroneutral exchange of protons for cations such as Na(+) or K(+) across membranes. May also exchange Li(+) and Cs(+) with a lower affinity.	GO:0005774; GO:0005886; GO:0009651; GO:0015081; GO:0015385; GO:0015386; GO:0016021; GO:0051453; GO:0055075; GO:0071805; GO:0098719	potassium ion homeostasis [GO:0055075]; potassium ion transmembrane transport [GO:0071805]; regulation of intracellular pH [GO:0051453]; response to salt stress [GO:0009651]; sodium ion import across plasma membrane [GO:0098719]
Pavir.4KG114300	Pavir.4KG114300.v5.1	Chr04K	14877613	14880289	(1 of 4) PTHR10980//PTHR10980:SF18 - RHO GDP-DISSOCIATION INHIBITOR // IMMUNOGLOBULIN E-SET SUPERFAMILY PROTEIN	#N/A	#N/A	#N/A	#N/A	#N/A	#N/A	#N/A
Pavir.4KG114400	Pavir.4KG114400.v5.1	Chr04K	14880525	14883731	(1 of 302) PF00651 - BTB/POZ domain (BTB)	#N/A	#N/A	#N/A	#N/A	#N/A	#N/A	#N/A
Pavir.4KG114500	Pavir.4KG114500.v5.1	Chr04K	14909905	14911360	(1 of 5) PF07172 - Glycine rich protein family (GRP)	#N/A	#N/A	#N/A	#N/A	#N/A	#N/A	#N/A
Pavir.4KG114600	Pavir.4KG114600.v5.1	Chr04K	14915889	14917106	(1 of 5) PF07172 - Glycine rich protein family (GRP)	#N/A	#N/A	#N/A	#N/A	#N/A	#N/A	#N/A
Pavir.4KG114605	Pavir.4KG114605.v5.1	Chr04K	14920205	14920726		#N/A	#N/A	#N/A	#N/A	#N/A	#N/A	#N/A
Pavir.4KG116400	Pavir.4KG116400.v5.1	Chr04K	14942926	14945159	(1 of 56) PTHR23155:SF455 - COILED-COIL DOMAIN-CONTAINING PROTEIN LOBO HOMOLOG	RPV1_VITRO	Disease resistance protein RPV1 (NAD(+) hydrolase RPV1) (EC 3.2.2.6) (Resistance to Plasmopara viticola protein) (MrRVP1)	RPV1	Vitis rotundifolia (Muscadine grape)	FUNCTION: Disease resistance (R) protein that confers resistance to multiple powdery and downy mildew by promoting cell death (PubMed:24033846, PubMed:28008335). Acts as a NAD(+) hydrolase (NADase): in response to activation, catalyzes cleavage of NAD(+) into ADP-D-ribose (ADPR) and nicotinamide; NAD(+) cleavage triggering a defense system that promotes cell death (PubMed:31439792). {ECO:0000269|PubMed:24033846, ECO:0000269|PubMed:28008335, ECO:0000269|PubMed:31439792}.	GO:0005634; GO:0005737; GO:0007165; GO:0009817; GO:0042803; GO:0043068; GO:0043531; GO:0050135; GO:0061809	defense response to fungus, incompatible interaction [GO:0009817]; positive regulation of programmed cell death [GO:0043068]; signal transduction [GO:0007165]
Pavir.4KG116405	Pavir.4KG116405.v5.1	Chr04K	14990538	14991609		#N/A	#N/A	#N/A	#N/A	#N/A	#N/A	#N/A
Pavir.4KG048500	Pavir.4KG048500.v5.1	Chr04K	15036543	15038606	(1 of 35) KOG1176 - Acyl-CoA synthetase	AAE1_ARATH	Probable acyl-activating enzyme 1, peroxisomal (EC 6.2.1.-) (AMP-binding protein 1) (AtAMPBP1)	AAE1 AMPBP1 At1g20560 F5M15.12	Arabidopsis thaliana (Mouse-ear cress)	FUNCTION: May act as an acid--thiol ligase that activates carboxylic acids by forming acyl-CoAs. {ECO:0000250}.	GO:0005777; GO:0006631; GO:0016874	fatty acid metabolic process [GO:0006631]
Pavir.4KG048505	Pavir.4KG048505.v5.1	Chr04K	15040624	15049965	(1 of 11) K19355 - mannan endo-1,4-beta-mannosidase (MAN)	MAN6_ORYSJ	Mannan endo-1,4-beta-mannosidase 6 (EC 3.2.1.78) (Beta-mannanase 6) (Endo-beta-1,4-mannanase 6) (OsMAN6)	MAN6 Os06g0311600 LOC_Os06g20620 OSJNBb0005N06.19	Oryza sativa subsp. japonica (Rice)		GO:0005576; GO:0005975; GO:0016985	carbohydrate metabolic process [GO:0005975]
Pavir.4KG048510	Pavir.4KG048510.v5.1	Chr04K	15043412	15043999	(1 of 140) PTHR23227//PTHR23227:SF41 - BUCENTAUR RELATED // SUBFAMILY NOT NAMED	CFDP2_BOVIN	Craniofacial development protein 2 (p97 bucentaur protein)	CFDP2 BCNT	Bos taurus (Bovine)		GO:0005634; GO:0005737	
Pavir.4KG048515	Pavir.4KG048515.v5.1	Chr04K	15045553	15046371	(1 of 598) PTHR19446//PTHR19446:SF355 - REVERSE TRANSCRIPTASES // SUBFAMILY NOT NAMED	#N/A	#N/A	#N/A	#N/A	#N/A	#N/A	#N/A
Pavir.4KG048900	Pavir.4KG048900.v5.1	Chr04K	15051218	15055038	(1 of 2) K05399 - lipopolysaccharide-binding protein (LBP)	Y1049_ARATH	Putative BPI/LBP family protein At1g04970	At1g04970 F13M7.4	Arabidopsis thaliana (Mouse-ear cress)		GO:0001530; GO:0005615; GO:0005773; GO:0005774; GO:0010468; GO:1903409	reactive oxygen species biosynthetic process [GO:1903409]; regulation of gene expression [GO:0010468]
Pavir.4KG048905	Pavir.4KG048905.v5.1	Chr04K	15056312	15062327	(1 of 2) PTHR12321//PTHR12321:SF44 - CPG BINDING PROTEIN // SUBFAMILY NOT NAMED	Y3228_ARATH	PHD finger protein At3g20280	At3g20280 MQC12.2	Arabidopsis thaliana (Mouse-ear cress)		GO:0046872	

Pavir.4KG048910	Pavir.4KG048910.v5.1	Chr04K	15073743	15078415	(1 of 2) K12199 - vacuolar protein sorting-associated protein VTA1 (VTA1, LIP5)	LIP5_ARATH	Protein HOMOLOG OF MAMMALIAN LYST-INTERACTING PROTEIN 5 (Protein EXTENSIN-LIKE) (EXT-LIKE)	LIP5 VTA1 At4g26750 F10M23.90	Arabidopsis thaliana (Mouse-ear cress)	FUNCTION: Involved in the endosomal multivesicular bodies (MVB) pathway. MVBs contain intraluminal vesicles (ILVs) that are generated by invagination and scission from the limiting membrane of the endosome and are delivered to lysosomes enabling degradation of membrane proteins (By similarity). Thought to be a cofactor of SKD1/VPS4, which catalyzes the disassembly of membrane-associated ESCRT-III (PubMed:17468262). Target of pathogen-responsive mitogen-activated protein kinases (MPKs) that plays a critical role in plant basal resistance to Pseudomonas syringae in a SKD1-dependent manner by promoting multivesicular bodies (MVBs) trafficking upon plant infection (PubMed:25010425). {ECO:0000250, ECO:0000269|PubMed:17468262, ECO:0000269|PubMed:25010425}.	GO:0005634; GO:0005737; GO:0005771; GO:0009814; GO:0010008; GO:0015031; GO:0016192; GO:0032511; GO:0042803; GO:0080001; GO:1900426; GO:1903335	defense response, incompatible interaction [GO:0009814]; late endosome to vacuole transport via multivesicular body sorting pathway [GO:0032511]; mucilage extrusion from seed coat [GO:0080001]; positive regulation of defense response to bacterium [GO:1900426]; protein transport [GO:0015031]; regulation of vacuolar transport [GO:1903335]; vesicle-mediated transport [GO:0016192]
Pavir.4KG048915	Pavir.4KG048915.v5.1	Chr04K	15081931	15082245		#N/A	#N/A	#N/A	#N/A	#N/A	#N/A	#N/A
Pavir.4KG116200	Pavir.4KG116200.v5.1	Chr04K	15083122	15087828	(1 of 4) PTHR19321//PTHR19321:SF11 - PROTEIN REGULATOR OF CYTOKINESIS 1 PRC1-RELATED // SUBFAMILY NOT NAMED	MA652_ARATH	65-kDa microtubule-associated protein 2 (AtMAP65-2)	MAP65-2 At4g26760 F10M23.100	Arabidopsis thaliana (Mouse-ear cress)	FUNCTION: Microtubule-associated protein that stabilize microtubules (MT). Involved in the regulation of MT organization and dynamics. Confers MT resistance to the drug propyzamide and cold conditions. {ECO:0000269|PubMed:19002591}.	GO:0000226; GO:0000911; GO:0000922; GO:0005634; GO:0005737; GO:0005819; GO:0005829; GO:0005874; GO:0008017; GO:0009524; GO:0009574; GO:0048528; GO:0051301; GO:0055028; GO:0072686; GO:1990023	cell division [GO:0051301]; cytokinesis by cell plate formation [GO:0000911]; microtubule cytoskeleton organization [GO:0000226]; post-embryonic root development [GO:0048528]
Pavir.4KG116300	Pavir.4KG116300.v5.1	Chr04K	15092463	15096611	(1 of 5) K04459 - dual specificity MAP kinase phosphatase (DUSP, MKP)	IBR5_ARATH	Protein-tyrosine-phosphatase IBR5 (EC 3.1.3.48) (Protein INDOLE-3-BUTYRIC ACID RESPONSE 5) (Protein IBA RESPONSE 5) (SKP1-interacting partner 33)	IBR5 SKIP33 At2g04550 T1O3.4	Arabidopsis thaliana (Mouse-ear cress)	FUNCTION: Required for the transduction of auxin and abscisic acid (ABA) signaling pathways. Dephosphorylates and inactivates the MAP kinase MPK12. {ECO:0000269|PubMed:14630970, ECO:0000269|PubMed:18423007, ECO:0000269|PubMed:18832358, ECO:0000269|PubMed:19000167}.	GO:0004725; GO:0005634; GO:0008138; GO:0009733; GO:0009734; GO:0009737; GO:0009738; GO:0033549; GO:0035556; GO:0043407; GO:0046620; GO:0061388	abscisic acid-activated signaling pathway [GO:0009738]; auxin-activated signaling pathway [GO:0009734]; intracellular signal transduction [GO:0035556]; negative regulation of MAP kinase activity [GO:0043407]; regulation of organ growth [GO:0046620]; regulation of rate of cell growth [GO:0061388]; response to abscisic acid [GO:0009737]; response to auxin [GO:0009733]
Pavir.4KG116305	Pavir.4KG116305.v5.1	Chr04K	15096714	15108450	(1 of 2) PTHR30560//PTHR30560:SF3 - TRIGGER FACTOR CHAPERONE AND PEPTIDYL-PROLYL CIS/TRANS ISOMERASE // TRIGGER FACTOR-LIKE PROTEIN TIG	TIG_ARATH	Trigger factor-like protein TIG, Chloroplastic (EC 5.2.1.8) (Immunophilin TIG) (Peptidyl-prolyl cis-trans isomerase TIG) (PPIase TIG) (Rotamase)	TIG At5g55220 MCO15_17	Arabidopsis thaliana (Mouse-ear cress)	FUNCTION: Involved in protein export. Acts as a chaperone by maintaining the newly synthesized protein in an open conformation. Functions as a peptidyl-prolyl cis-trans isomerase (By similarity). {ECO:0000250}.	GO:0003729; GO:0003755; GO:0009507; GO:0009536; GO:0009570; GO:0009941; GO:0015031; GO:0043022; GO:0043335; GO:0044183; GO:0051083; GO:0061077	'de novo' cotranslational protein folding [GO:0051083]; chaperone-mediated protein folding [GO:0061077]; protein transport [GO:0015031]; protein unfolding [GO:0043335]
Pavir.4KG142800	Pavir.4KG142800.v5.1	Chr04K	15116140	15120027		#N/A	#N/A	#N/A	#N/A	#N/A	#N/A	#N/A
Pavir.4KG142805	Pavir.4KG142805.v5.1	Chr04K	15122914	15124689		#N/A	#N/A	#N/A	#N/A	#N/A	#N/A	#N/A
Pavir.4KG142200	Pavir.4KG142200.v5.1	Chr04K	15125988	15127463	(1 of 7) PTHR13683//PTHR13683:SF367 - ASPARTYL PROTEASES // SUBFAMILY NOT NAMED	ASPA_ARATH	Aspartyl protease family protein At5g10770 (EC 3.4.23.-)	At5g10770 T30N20_40	Arabidopsis thaliana (Mouse-ear cress)	FUNCTION: Probably not redundant with AED1 and not involved in restriction of salicylic acid (SA) or systemic acquired resistance (SAR) signaling. {ECO:0000269|PubMed:24755512}.	GO:0004190; GO:0005886; GO:0031225	
Pavir.4KG142205	Pavir.4KG142205.v5.1	Chr04K	15182104	15183165		#N/A	#N/A	#N/A	#N/A	#N/A	#N/A	#N/A
Pavir.4KG142210	Pavir.4KG142210.v5.1	Chr04K	15183022	15191567		#N/A	#N/A	#N/A	#N/A	#N/A	#N/A	#N/A
Pavir.4KG142215	Pavir.4KG142215.v5.1	Chr04K	15213929	15215498		#N/A	#N/A	#N/A	#N/A	#N/A	#N/A	#N/A
Pavir.4KG142220	Pavir.4KG142220.v5.1	Chr04K	15306289	15312113		#N/A	#N/A	#N/A	#N/A	#N/A	#N/A	#N/A
Pavir.4KG142225	Pavir.4KG142225.v5.1	Chr04K	15336668	15337081		#N/A	#N/A	#N/A	#N/A	#N/A	#N/A	#N/A
Pavir.4KG142230	Pavir.4KG142230.v5.1	Chr04K	15347457	15348962	(1 of 7) PTHR13683//PTHR13683:SF367 - ASPARTYL PROTEASES // SUBFAMILY NOT NAMED	#N/A	#N/A	#N/A	#N/A	#N/A	#N/A	#N/A
Pavir.4KG142235	Pavir.4KG142235.v5.1	Chr04K	15355006	15356806	(1 of 35) PTHR12606//PTHR12606:SF31 - SENTRIN/SUMO-SPECIFIC PROTEASE // SUBFAMILY NOT NAMED	#N/A	#N/A	#N/A	#N/A	#N/A	#N/A	#N/A
Pavir.4KG142240	Pavir.4KG142240.v5.1	Chr04K	15356807	15361695	(1 of 35) PTHR12606//PTHR12606:SF31 - SENTRIN/SUMO-SPECIFIC PROTEASE // SUBFAMILY NOT NAMED	#N/A	#N/A	#N/A	#N/A	#N/A	#N/A	#N/A
Pavir.4KG142245	Pavir.4KG142245.v5.1	Chr04K	15394942	15399070	(1 of 115) K10523 - speckle-type POZ protein (SPOP)	BPM1_ARATH	BTB/POZ and MATH domain-containing protein 1 (Protein BTB-POZ AND MATH DOMAIN 1) (AtBPM1)	BPM1 At5g19000 T16G12.40	Arabidopsis thaliana (Mouse-ear cress)	FUNCTION: May act as a substrate-specific adapter of an E3 ubiquitin-protein ligase complex (CUL3-RBX1-BTB) which mediates the ubiquitination and subsequent proteasomal degradation of target proteins. {ECO:0000269|PubMed:15618422}.	GO:0005634; GO:0006970; GO:0016567; GO:0042631; GO:0042802; GO:0071472	cellular response to salt stress [GO:0071472]; cellular response to water deprivation [GO:0042631]; protein ubiquitination [GO:0016567]; response to osmotic stress [GO:0006970]
Pavir.4KG140900	Pavir.4KG140900.v5.1	Chr04K	15402365	15403845	(1 of 7) PTHR13683//PTHR13683:SF367 - ASPARTYL PROTEASES // SUBFAMILY NOT NAMED	ASPA_ARATH	Aspartyl protease family protein At5g10770 (EC 3.4.23.-)	At5g10770 T30N20_40	Arabidopsis thaliana (Mouse-ear cress)	FUNCTION: Probably not redundant with AED1 and not involved in restriction of salicylic acid (SA) or systemic acquired resistance (SAR) signaling. {ECO:0000269|PubMed:24755512}.	GO:0004190; GO:0005886; GO:0031225	
Pavir.4KG140800	Pavir.4KG140800.v5.1	Chr04K	15405150	15406021		#N/A	#N/A	#N/A	#N/A	#N/A	#N/A	#N/A
Pavir.4KG140805	Pavir.4KG140805.v5.1	Chr04K	15430552	15431190		#N/A	#N/A	#N/A	#N/A	#N/A	#N/A	#N/A
Pavir.4KG140810	Pavir.4KG140810.v5.1	Chr04K	15440032	15442982	(1 of 117) PF07762 - Protein of unknown function (DUF1618) (DUF1618)	#N/A	#N/A	#N/A	#N/A	#N/A	#N/A	#N/A
Pavir.4KG140100	Pavir.4KG140100.v5.1	Chr04K	15448749	15452538	(1 of 26) 3.4.21.72 - IgA-specific serine endopeptidase / Immunoglobulin A1 protease	RBP2A_PLAF7	Reticulocyte-binding protein 2 homolog a	PF13_0198	Plasmodium falciparum (isolate 3D7)	FUNCTION: Involved in reticulocyte adhesion. {ECO:0000250|UniProtKB:Q00799}.	GO:0005886; GO:0008201; GO:0016020; GO:0016021; GO:0098609	cell-cell adhesion [GO:0098609]
Pavir.4KG140105	Pavir.4KG140105.v5.1	Chr04K	15455363	15456088	(1 of 5) PF13975 - gag-polyprotein putative aspartyl protease (gag-asp_proteas)	#N/A	#N/A	#N/A	#N/A	#N/A	#N/A	#N/A
Pavir.4KG140300	Pavir.4KG140300.v5.1	Chr04K	15479337	15482106	(1 of 117) PF07762 - Protein of unknown function (DUF1618) (DUF1618)	#N/A	#N/A	#N/A	#N/A	#N/A	#N/A	#N/A

Pavir.4KG140400	Pavir.4KG140400.v5.1	Chr04K	15488219	15490409	(1 of 117) PF07762 - Protein of unknown function (DUF1618) (DUF1618)	#N/A	#N/A	#N/A	#N/A	#N/A	#N/A	#N/A
Pavir.4KG139900	Pavir.4KG139900.v5.1	Chr04K	15511065	15515737	(1 of 117) PF07762 - Protein of unknown function (DUF1618) (DUF1618)	#N/A	#N/A	#N/A	#N/A	#N/A	#N/A	#N/A
Pavir.4KG139200	Pavir.4KG139200.v5.1	Chr04K	15572290	15573572	(1 of 226) K00430 - peroxidase (E1.11.1.7)	PER1_ORYSJ	Peroxidase 1 (EC 1.11.1.7)	PRX74 Os05g0499300 LOC_Os05g41990 OJ1057_B02.6 OsJ_19081	Oryza sativa subsp. japonica (Rice)	FUNCTION: Removal of H(2)O(2), oxidation of toxic reductants, biosynthesis and degradation of lignin, suberization, auxin catabolism, response to environmental stresses such as wounding, pathogen attack and oxidative stress. These functions might be dependent on each isozyme/isoform in each plant tissue.	GO:0004601; GO:0005576; GO:0006979; GO:0009505; GO:0009506; GO:0020037; GO:0042744; GO:0046872	hydrogen peroxide catabolic process [GO:0042744]; response to oxidative stress [GO:0006979]
Pavir.4KG139205	Pavir.4KG139205.v5.1	Chr04K	15581009	15583330		#N/A	#N/A	#N/A	#N/A	#N/A	#N/A	#N/A
Pavir.4KG139210	Pavir.4KG139210.v5.1	Chr04K	15590479	15592798		#N/A	#N/A	#N/A	#N/A	#N/A	#N/A	#N/A
Pavir.4KG139215	Pavir.4KG139215.v5.1	Chr04K	15601173	15602193		#N/A	#N/A	#N/A	#N/A	#N/A	#N/A	#N/A
Pavir.4KG146200	Pavir.4KG146200.v5.1	Chr04K	15662042	15663608	(1 of 5) PTHR13683//PTHR13683:SF304 - ASPARTYL PROTEASES // SUBFAMILY NOT NAMED	#N/A	#N/A	#N/A	#N/A	#N/A	#N/A	#N/A
Pavir.4KG126800	Pavir.4KG126800.v5.1	Chr04K	15762306	15779841	(1 of 108) PF00069//PF00139 - Protein kinase domain (Pkinase) // Legume lectin domain (Lectin_legB)	SIT2_ORYSJ	L-type lectin-domain containing receptor kinase SIT2 (EC 2.7.11.1) (Protein SALT INTOLERANCE 2)	SIT2 Os04g0531400 LOC_Os04g44900 OsJ_15568 OSJNBa0081C01.16	Oryza sativa subsp. japonica (Rice)	FUNCTION: Lectin-domain containing receptor kinase involved in salt stress response (PubMed:24907341). Acts as negative regulator of salt tolerance (PubMed:24907341). {ECO:0000269|PubMed:24907341}.	GO:0004675; GO:0005524; GO:0005886; GO:0006468; GO:0016021; GO:0030246; GO:1901001	negative regulation of response to salt stress [GO:1901001]; protein phosphorylation [GO:0006468]
Pavir.4KG126805	Pavir.4KG126805.v5.1	Chr04K	15763842	15764566	(1 of 2827) 2.7.11.1 - Non-specific serine/threonine protein kinase / Threonine-specific protein kinase	#N/A	#N/A	#N/A	#N/A	#N/A	#N/A	#N/A
Pavir.4KG148200	Pavir.4KG148200.v5.1	Chr04K	15770686	15772688	(1 of 108) PF00069//PF00139 - Protein kinase domain (Pkinase) // Legume lectin domain (Lectin_legB)	SIT2_ORYSJ	L-type lectin-domain containing receptor kinase SIT2 (EC 2.7.11.1) (Protein SALT INTOLERANCE 2)	SIT2 Os04g0531400 LOC_Os04g44900 OsJ_15568 OSJNBa0081C01.16	Oryza sativa subsp. japonica (Rice)	FUNCTION: Lectin-domain containing receptor kinase involved in salt stress response (PubMed:24907341). Acts as negative regulator of salt tolerance (PubMed:24907341). {ECO:0000269|PubMed:24907341}.	GO:0004675; GO:0005524; GO:0005886; GO:0006468; GO:0016021; GO:0030246; GO:1901001	negative regulation of response to salt stress [GO:1901001]; protein phosphorylation [GO:0006468]
Pavir.4KG127300	Pavir.4KG127300.v5.1	Chr04K	15786853	15794776	(1 of 108) PF00069//PF00139 - Protein kinase domain (Pkinase) // Legume lectin domain (Lectin_legB)	SIT2_ORYSJ	L-type lectin-domain containing receptor kinase SIT2 (EC 2.7.11.1) (Protein SALT INTOLERANCE 2)	SIT2 Os04g0531400 LOC_Os04g44900 OsJ_15568 OSJNBa0081C01.16	Oryza sativa subsp. japonica (Rice)	FUNCTION: Lectin-domain containing receptor kinase involved in salt stress response (PubMed:24907341). Acts as negative regulator of salt tolerance (PubMed:24907341). {ECO:0000269|PubMed:24907341}.	GO:0004675; GO:0005524; GO:0005886; GO:0006468; GO:0016021; GO:0030246; GO:1901001	negative regulation of response to salt stress [GO:1901001]; protein phosphorylation [GO:0006468]
Pavir.4KG127200	Pavir.4KG127200.v5.1	Chr04K	15801021	15804733	(1 of 9) PTHR12606//PTHR12606:SF22 - SENTRIN/SUMO-SPECIFIC PROTEASE // SUBFAMILY NOT NAMED	#N/A	#N/A	#N/A	#N/A	#N/A	#N/A	#N/A
Pavir.4KG127205	Pavir.4KG127205.v5.1	Chr04K	15804972	15805283		#N/A	#N/A	#N/A	#N/A	#N/A	#N/A	#N/A
Pavir.4KG127210	Pavir.4KG127210.v5.1	Chr04K	15806312	15806635		#N/A	#N/A	#N/A	#N/A	#N/A	#N/A	#N/A
Pavir.4KG127100	Pavir.4KG127100.v5.1	Chr04K	15807175	15807811	(1 of 9) PTHR12606//PTHR12606:SF22 - SENTRIN/SUMO-SPECIFIC PROTEASE // SUBFAMILY NOT NAMED	#N/A	#N/A	#N/A	#N/A	#N/A	#N/A	#N/A
Pavir.4KG127105	Pavir.4KG127105.v5.1	Chr04K	15809150	15812098	(1 of 208) PF03101 - FAR1 DNA-binding domain (FAR1)	FRS5_ARATH	Protein FAR1-RELATED SEQUENCE 5	FRS5 At4g38180 F20D10.300	Arabidopsis thaliana (Mouse-ear cress)	FUNCTION: Putative transcription activator involved in regulating light control of development.	GO:0005634; GO:0006355; GO:0008270	regulation of transcription, DNA-templated [GO:0006355]
Pavir.4KG127000	Pavir.4KG127000.v5.1	Chr04K	15814353	15816390	(1 of 108) PF00069//PF00139 - Protein kinase domain (Pkinase) // Legume lectin domain (Lectin_legB)	SIT2_ORYSJ	L-type lectin-domain containing receptor kinase SIT2 (EC 2.7.11.1) (Protein SALT INTOLERANCE 2)	SIT2 Os04g0531400 LOC_Os04g44900 OsJ_15568 OSJNBa0081C01.16	Oryza sativa subsp. japonica (Rice)	FUNCTION: Lectin-domain containing receptor kinase involved in salt stress response (PubMed:24907341). Acts as negative regulator of salt tolerance (PubMed:24907341). {ECO:0000269|PubMed:24907341}.	GO:0004675; GO:0005524; GO:0005886; GO:0006468; GO:0016021; GO:0030246; GO:1901001	negative regulation of response to salt stress [GO:1901001]; protein phosphorylation [GO:0006468]
Pavir.4KG126900	Pavir.4KG126900.v5.1	Chr04K	15823605	15825847	(1 of 108) PF00069//PF00139 - Protein kinase domain (Pkinase) // Legume lectin domain (Lectin_legB)	SIT2_ORYSJ	L-type lectin-domain containing receptor kinase SIT2 (EC 2.7.11.1) (Protein SALT INTOLERANCE 2)	SIT2 Os04g0531400 LOC_Os04g44900 OsJ_15568 OSJNBa0081C01.16	Oryza sativa subsp. japonica (Rice)	FUNCTION: Lectin-domain containing receptor kinase involved in salt stress response (PubMed:24907341). Acts as negative regulator of salt tolerance (PubMed:24907341). {ECO:0000269|PubMed:24907341}.	GO:0004675; GO:0005524; GO:0005886; GO:0006468; GO:0016021; GO:0030246; GO:1901001	negative regulation of response to salt stress [GO:1901001]; protein phosphorylation [GO:0006468]
Pavir.4KG126400	Pavir.4KG126400.v5.1	Chr04K	15878976	15881083	(1 of 108) PF00069//PF00139 - Protein kinase domain (Pkinase) // Legume lectin domain (Lectin_legB)	SIT2_ORYSJ	L-type lectin-domain containing receptor kinase SIT2 (EC 2.7.11.1) (Protein SALT INTOLERANCE 2)	SIT2 Os04g0531400 LOC_Os04g44900 OsJ_15568 OSJNBa0081C01.16	Oryza sativa subsp. japonica (Rice)	FUNCTION: Lectin-domain containing receptor kinase involved in salt stress response (PubMed:24907341). Acts as negative regulator of salt tolerance (PubMed:24907341). {ECO:0000269|PubMed:24907341}.	GO:0004675; GO:0005524; GO:0005886; GO:0006468; GO:0016021; GO:0030246; GO:1901001	negative regulation of response to salt stress [GO:1901001]; protein phosphorylation [GO:0006468]

Pavir.4KG126300	Pavir.4KG126300.v5.1	Chr04K	15906774	15908810	(1 of 108) PF00069//PF00139 - Protein kinase domain (Pkinase) // Legume lectin domain (Lectin_legB)	SIT2_ORYSJ	L-type lectin-domain containing receptor kinase SIT2 (EC 2.7.11.1) (Protein SALT INTOLERANCE 2)	SIT2 Os04g0531400 LOC_Os04g44900 OsJ_15568 OSJNBa0081C01.16	Oryza sativa subsp. japonica (Rice)	FUNCTION: Lectin-domain containing receptor kinase involved in salt stress response (PubMed:24907341). Acts as negative regulator of salt tolerance (PubMed:24907341). {ECO:0000269|PubMed:24907341}.	GO:0004675; GO:0005524; GO:0005886; GO:0006468; GO:0016021; GO:0030246; GO:1901001	negative regulation of response to salt stress [GO:1901001]; protein phosphorylation [GO:0006468]
Pavir.4KG126000	Pavir.4KG126000.v5.1	Chr04K	15958636	15960489	(1 of 181) PF12776 - Myb/SANT-like DNA-binding domain (Myb_DNA-bind_3)	#N/A	#N/A	#N/A	#N/A	#N/A	#N/A	#N/A
Pavir.4KG126005	Pavir.4KG126005.v5.1	Chr04K	15959287	15959769	(1 of 598) PTHR19446//PTHR19446:SF355 - REVERSE TRANSCRIPTASES // SUBFAMILY NOT NAMED	#N/A	#N/A	#N/A	#N/A	#N/A	#N/A	#N/A
Pavir.4KG125900	Pavir.4KG125900.v5.1	Chr04K	15960650	15960871		#N/A	#N/A	#N/A	#N/A	#N/A	#N/A	#N/A
Pavir.4KG125905	Pavir.4KG125905.v5.1	Chr04K	15963355	15963820		#N/A	#N/A	#N/A	#N/A	#N/A	#N/A	#N/A
Pavir.4KG125700	Pavir.4KG125700.v5.1	Chr04K	15987074	15988345	(1 of 5) PTHR13683//PTHR13683:SF304 - ASPARTYL PROTEASES // SUBFAMILY NOT NAMED	ASPA_ARATH	Aspartyl protease family protein At5g10770 (EC 3.4.23.-)	At5g10770 T30N20_40	Arabidopsis thaliana (Mouse-ear cress)	FUNCTION: Probably not redundant with AED1 and not involved in restriction of salicylic acid (SA) or systemic acquired resistance (SAR) signaling. {ECO:0000269|PubMed:24755512}.	GO:0004190; GO:0005886; GO:0031225	
Pavir.4KG125500	Pavir.4KG125500.v5.1	Chr04K	16003357	16004625	(1 of 29) PF05078 - Protein of unknown function (DUF679) (DUF679)	DMP4_ARATH	Protein DMP4 (AtDMP4)	DMP4 At4g18425	Arabidopsis thaliana (Mouse-ear cress)	FUNCTION: Involved in membrane remodeling. {ECO:0000250|UniProtKB:Q9LVF4}.	GO:0009705; GO:0009838; GO:0010256; GO:0016021; GO:0090693	abscission [GO:0009838]; endomembrane system organization [GO:0010256]; plant organ senescence [GO:0090693]
Pavir.4KG125300	Pavir.4KG125300.v5.1	Chr04K	16030593	16039887	(1 of 26) PF03080//PF14365 - Domain of unknown function (DUF239) (DUF239) // Domain of unknown function (DUF4409) (DUF4409)	#N/A	#N/A	#N/A	#N/A	#N/A	#N/A	#N/A
Pavir.4KG125100	Pavir.4KG125100.v5.1	Chr04K	16055512	16058087		#N/A	#N/A	#N/A	#N/A	#N/A	#N/A	#N/A
Pavir.4KG157600	Pavir.4KG157600.v5.1	Chr04K	16126853	16129015		#N/A	#N/A	#N/A	#N/A	#N/A	#N/A	#N/A
Pavir.4KG157605	Pavir.4KG157605.v5.1	Chr04K	16200773	16202653	(1 of 122) PF04434 - SWIM zinc finger (SWIM)	FRS3_ARATH	Protein FAR1-RELATED SEQUENCE 3	FRS3 At2g27110 T20P8.16	Arabidopsis thaliana (Mouse-ear cress)	FUNCTION: Putative transcription activator involved in regulating light control of development.	GO:0005634; GO:0006355; GO:0008270; GO:0009506	regulation of transcription, DNA-templated [GO:0006355]
Pavir.4KG249300	Pavir.4KG249300.v5.1	Chr04K	16203646	16205264	(1 of 9) PTHR12606//PTHR12606:SF22 - SENTRIN/SUMO-SPECIFIC PROTEASE // SUBFAMILY NOT NAMED	#N/A	#N/A	#N/A	#N/A	#N/A	#N/A	#N/A
Pavir.4KG249305	Pavir.4KG249305.v5.1	Chr04K	16211691	16213614		#N/A	#N/A	#N/A	#N/A	#N/A	#N/A	#N/A
Pavir.4KG137000	Pavir.4KG137000.v5.1	Chr04K	16231044	16233131	(1 of 205) PF04578//PF13968 - Protein of unknown function, DUF594 (DUF594) // Domain of unknown function (DUF4220) (DUF4220)	#N/A	#N/A	#N/A	#N/A	#N/A	#N/A	#N/A
Pavir.4KG137005	Pavir.4KG137005.v5.1	Chr04K	16352972	16353346		#N/A	#N/A	#N/A	#N/A	#N/A	#N/A	#N/A
Pavir.4KG136800	Pavir.4KG136800.v5.1	Chr04K	16389932	16394688	(1 of 2) PTHR13683//PTHR13683:SF371 - ASPARTYL PROTEASES // SUBFAMILY NOT NAMED	ASPA_ARATH	Aspartyl protease family protein At5g10770 (EC 3.4.23.-)	At5g10770 T30N20_40	Arabidopsis thaliana (Mouse-ear cress)	FUNCTION: Probably not redundant with AED1 and not involved in restriction of salicylic acid (SA) or systemic acquired resistance (SAR) signaling. {ECO:0000269|PubMed:24755512}.	GO:0004190; GO:0005886; GO:0031225	
Pavir.4KG136805	Pavir.4KG136805.v5.1	Chr04K	16397506	16398141		#N/A	#N/A	#N/A	#N/A	#N/A	#N/A	#N/A
Pavir.4KG136600	Pavir.4KG136600.v5.1	Chr04K	16411596	16413790	(1 of 28) PF01985 - CRS1 / YhbY (CRM) domain (CRS1_YhbY)	Y3544_ARATH	Uncharacterized CRM domain-containing protein At3g25440, chloroplastic	At3g25440 MWL2.5	Arabidopsis thaliana (Mouse-ear cress)		GO:0003723; GO:0009507	
Pavir.4KG136605	Pavir.4KG136605.v5.1	Chr04K	16414559	16416898	(1 of 19) PTHR12606//PTHR12606:SF25 - SENTRIN/SUMO-SPECIFIC PROTEASE // SUBFAMILY NOT NAMED	#N/A	#N/A	#N/A	#N/A	#N/A	#N/A	#N/A
Pavir.4KG136610	Pavir.4KG136610.v5.1	Chr04K	16417968	16419134		#N/A	#N/A	#N/A	#N/A	#N/A	#N/A	#N/A
Pavir.4KG136200	Pavir.4KG136200.v5.1	Chr04K	16427230	16433292	(1 of 1) PTHR11670//PTHR11670:SF37 - ACONITASE // ACONITATE HYDRATASE 3, MITOCHONDRIAL	ACOC_CUCMA	Aconitate hydratase, cytoplasmic (Aconitase) (EC 4.2.1.3) (Citrate hydro-lyase)		Cucurbita maxima (Pumpkin) (Winter squash)	FUNCTION: Catalyzes the isomerization of citrate to isocitrate via cis-aconitate. {ECO:0000250}.	GO:0003994; GO:0005737; GO:0006097; GO:0046872; GO:0047780; GO:0051539	glyoxylate cycle [GO:0006097]
Pavir.4KG136100	Pavir.4KG136100.v5.1	Chr04K	16442492	16444723	(1 of 8) 4.2.1.3 - Aconitate hydratase / Citrate(isocitrate) hydro-lyase	ACOC_CUCMC	Aconitate hydratase (Aconitase) (EC 4.2.1.3) (Citrate hydro-lyase) (Fragment)	ACO	Cucumis melo var. conomon (Oriental pickling melon)	FUNCTION: Catalyzes the isomerization of citrate to isocitrate via cis-aconitate. {ECO:0000250}.	GO:0003994; GO:0005737; GO:0006097; GO:0046872; GO:0047780; GO:0051539	glyoxylate cycle [GO:0006097]
Pavir.4KG136000	Pavir.4KG136000.v5.1	Chr04K	16445207	16446919	(1 of 119) PF13912 - C2H2-type zinc finger (zf-C2H2_6)	#N/A	#N/A	#N/A	#N/A	#N/A	#N/A	#N/A
Pavir.4KG135900	Pavir.4KG135900.v5.1	Chr04K	16476609	16483731	(1 of 4) PTHR10641//PTHR10641:SF517 - MYB-LIKE DNA-BINDING PROTEIN MYB // GB|AAC33480.1	DMTF1_DANRE	Cyclin-D-binding Myb-like transcription factor 1	dmtf1 si:dkey-153k10.8 zgc:92448	Danio rerio (Zebrafish) (Brachydanio rerio)	FUNCTION: Transcriptional activator which activates the CDKN2A/ARF locus in response to Ras-Raf signaling, thereby promoting p53/TP53-dependent growth arrest. Binds to the consensus sequence 5'-CCCG[GT]ATGT-3'. {ECO:0000250}.	GO:0000978; GO:0005654; GO:0007049	cell cycle [GO:0007049]
Pavir.4KG135700	Pavir.4KG135700.v5.1	Chr04K	16486551	16487035		#N/A	#N/A	#N/A	#N/A	#N/A	#N/A	#N/A
Pavir.4KG135600	Pavir.4KG135600.v5.1	Chr04K	16610641	16616958	(1 of 42) PF03181 - BURP domain (BURP)	BURPB_ORYSJ	BURP domain-containing protein 11 (OsBURP11)	BURP11 Os06g0302000 LOC_Os06g19800 OJ1217_C01.19 OSJNBa0042E12.9	Oryza sativa subsp. japonica (Rice)			

Pavir.4KG135500	Pavir.4KG135500.v5.1	Chr04K	16623819	16645204	(1 of 2) K06699 - proteasome activator subunit 4 (PSME4)	PSME4_ARATH	Proteasome activator subunit 4 (Proteasome activator PA200)	PA200 At3g13330 MDC11.16	Arabidopsis thaliana (Mouse-ear cress)	FUNCTION: Associated component of the proteasome that specifically recognizes acetylated histones and promotes ATP- and ubiquitin-independent degradation of core histones during DNA damage response. Recognizes and binds acetylated histones via its bromodomain-like (BRDL) region and activates the proteasome by opening the gated channel for substrate entry. Binds to the core proteasome via its C-terminus, which occupies the same binding sites as the proteasomal ATPases, opening the closed structure of the proteasome via an active gating mechanism. involved in DNA damage response: binds to acetylated histones and promotes degradation of histones (By similarity). {ECO:0000250}.	GO:0000502; GO:0005634; GO:0005829; GO:0006281; GO:0010499; GO:0016504; GO:0070577; GO:0070628	DNA repair [GO:0006281]; proteasomal ubiquitin-independent protein catabolic process [GO:0010499]
Pavir.4KG135300	Pavir.4KG135300.v5.1	Chr04K	16648680	16652541	(1 of 3) PTHR12668//PTHR12668:SF18 - TRANSMEMBRANE PROTEIN 14, 15 // SUBFAMILY NOT NAMED	FAX1_ARATH	Protein FATTY ACID EXPORT 1, chloroplastic (At-FAX1)	FAX1 At3g57280 F28O9.130	Arabidopsis thaliana (Mouse-ear cress)	FUNCTION: Mediates the export of free fatty acid from the plastids. Potentially prefers palmitic acid (C16:0) over oleic acid (C18:1) and stearic acid (C18:0). Not involved in fatty acid activation. Required for biogenesis of the outer pollen cell wall, in particular for the assembly of exine and pollen coat and for the release of ketone wax components. {ECO:0000269|PubMed:25646734}.	GO:0005829; GO:0009507; GO:0009536; GO:0009706; GO:0009941; GO:0010208; GO:0015245; GO:0015908; GO:0016020; GO:0016021; GO:0055088; GO:0071668; GO:1902001	fatty acid transmembrane transport [GO:1902001]; fatty acid transport [GO:0015908]; lipid homeostasis [GO:0055088]; plant-type cell wall assembly [GO:0071668]; pollen wall assembly [GO:0010208]
Pavir.4KG135305	Pavir.4KG135305.v5.1	Chr04K	16660633	16661910	(1 of 39) K08818 - cell division cycle 2-like [EC:2.7.11.22] (CDC2L)	CDKF2_ORYSJ	Putative cyclin-dependent kinase F-2 (CDKF;2) (EC 2.7.11.22) (EC 2.7.11.23)	CDKF-2 Os12g0424700 LOC_Os12g23700	Oryza sativa subsp. japonica (Rice)		GO:0000307; GO:0000790; GO:0004693; GO:0005524; GO:0005634; GO:0006468; GO:0008353; GO:0032968; GO:0070816	phosphorylation of RNA polymerase II C-terminal domain [GO:0070816]; positive regulation of transcription elongation from RNA polymerase II promoter [GO:0032968]; protein phosphorylation [GO:0006468]
Pavir.4KG135310	Pavir.4KG135310.v5.1	Chr04K	16665602	16670907	(1 of 4) K10601 - E3 ubiquitin-protein ligase synoviolin (SYVN1, HRD1)	HRD1_ORYSJ	ERAD-associated E3 ubiquitin-protein ligase HRD1 (EC 2.3.2.27) (RING-type E3 ubiquitin transferase HRD1)	HRD1 Os06g0301000 LOC_Os06g19680 OJ1217_C01.4 P0501G08.42	Oryza sativa subsp. japonica (Rice)	FUNCTION: Probable component of the HRD1 ubiquitin ligase complex that mediates the rapid degradation of misfolded endoplasmic reticulum (ER) proteins, a process called ER-associated degradation (ERAD). {ECO:0000250|UniProtKB:Q9LW77}.	GO:0005789; GO:0016021; GO:0016567; GO:0016740; GO:0046872	protein ubiquitination [GO:0016567]
Pavir.4KG135315	Pavir.4KG135315.v5.1	Chr04K	16673877	16679321	(1 of 42) PF03181 - BURP domain (BURP)	BURPA_ORYSJ	BURP domain-containing protein 10 (OsBURP10)	BURP10 Os06g0281800 LOC_Os06g17000 OsJ_20979 OSJNBa0063H02.22	Oryza sativa subsp. japonica (Rice)			
Pavir.4KG164800	Pavir.4KG164800.v5.1	Chr04K	16691817	16694091		#N/A	#N/A	#N/A	#N/A	#N/A	#N/A	#N/A
Pavir.4KG135200	Pavir.4KG135200.v5.1	Chr04K	16698090	16706407	(1 of 3) KOG1916 - Nuclear protein, contains WD40 repeats	VCS_ARATH	Enhancer of mRNA-decapping protein 4 (Protein VARICOSE)	VCS EDC4 At3g13300 MDC11.13	Arabidopsis thaliana (Mouse-ear cress)	FUNCTION: As a component of the decapping complex, involved in the degradation of mRNAs. Essential for postembryonic development, especially during the formation of the shoot (SAM) and root apical meristems. Required for normal patterning of internal tissues of leaves. {ECO:0000269|PubMed:14660546, ECO:0000269|PubMed:17158604, ECO:0000269|PubMed:17513503}.	GO:0000932; GO:0003729; GO:0005634; GO:0005829; GO:0006397; GO:0006402; GO:0009791; GO:0009965; GO:0010071; GO:0010072; GO:0031087; GO:0042803; GO:0071365	cellular response to auxin stimulus [GO:0071365]; deadenylation-independent decapping of nuclear-transcribed mRNA [GO:0031087]; leaf morphogenesis [GO:0009965]; mRNA catabolic process [GO:0006402]; mRNA processing [GO:0006397]; post-embryonic development [GO:0009791]; primary shoot apical meristem specification [GO:0010072]; root meristem specification [GO:0010071]
Pavir.4KG135205	Pavir.4KG135205.v5.1	Chr04K	16709638	16720702	(1 of 2) K15425 - serine/threonine-protein phosphatase 4 regulatory subunit 2 (PPP4R2)	PP4R2_DROME	Serine/threonine-protein phosphatase 4 regulatory subunit 2 (PPP4R2-related protein)	PPP4R2r CG2890	Drosophila melanogaster (Fruit fly)	FUNCTION: Regulatory subunit of serine/threonine-protein phosphatase 4 (PP4) (By similarity). The probable PP4 complex Pp4-19C-PPP4R2r-flfl (PPP4C-PPP4R2-PPP4R3) is required to prevent caspase induced cell death (in vitro). {ECO:0000250, ECO:0000269|PubMed:18487071}.	GO:0000278; GO:0000775; GO:0005634; GO:0005737; GO:0006470; GO:0019888; GO:0030289; GO:0045879	mitotic cell cycle [GO:0000278]; negative regulation of smoothened signaling pathway [GO:0045879]; protein dephosphorylation [GO:0006470]
Pavir.4KG135210	Pavir.4KG135210.v5.1	Chr04K	16714229	16714912		#N/A	#N/A	#N/A	#N/A	#N/A	#N/A	#N/A
Pavir.4KG135215	Pavir.4KG135215.v5.1	Chr04K	16739264	16740253		#N/A	#N/A	#N/A	#N/A	#N/A	#N/A	#N/A
Pavir.4KG135100	Pavir.4KG135100.v5.1	Chr04K	16743082	16748275	(1 of 2) K17427 - large subunit ribosomal protein L46 (MRPL46)	RM46_HUMAN	39S ribosomal protein L46, mitochondrial (L46mt) (MRP-L46) (Mitochondrial large ribosomal subunit protein mL46) (P2ECSL)	MRPL46 C15orf4 LIECG2	Homo sapiens (Human)		GO:0003735; GO:0005654; GO:0005739; GO:0005743; GO:0005762; GO:0016787; GO:0030054; GO:0070125; GO:0070126	mitochondrial translational elongation [GO:0070125]; mitochondrial translational termination [GO:0070126]
Pavir.4KG135000	Pavir.4KG135000.v5.1	Chr04K	16748709	16751814	(1 of 2) PTHR31133:SF2 - EXPRESSED PROTEIN	Y3739_ARATH	Uncharacterized membrane protein At3g27390	At3g27390 K1G2.10	Arabidopsis thaliana (Mouse-ear cress)		GO:0005886; GO:0016021	
Pavir.4KG135005	Pavir.4KG135005.v5.1	Chr04K	16763745	16764533	(1 of 140) PTHR23227//PTHR23227:SF41 - BUCENTAUR RELATED // SUBFAMILY NOT NAMED	CFDP2_BOVIN	Craniofacial development protein 2 (p97 bucentaur protein)	CFDP2 BCNT	Bos taurus (Bovine)		GO:0005634; GO:0005737	
Pavir.4KG123500	Pavir.4KG123500.v5.1	Chr04K	16782759	16784560	(1 of 14) K10251 - 17beta-estradiol 17-dehydrogenase / very-long-chain 3-oxoacyl-CoA reductase (HSD17B12, KAR, IFA38)	KCR1_ARATH	Very-long-chain 3-oxoacyl-CoA reductase 1 (EC 1.1.1.330) (Beta-ketoacyl reductase 1) (AtKCR1) (Protein GLOSSY 8) (gl8At)	KCR1 At-YBR159 GL8 At1g67730 F12A21.31	Arabidopsis thaliana (Mouse-ear cress)	FUNCTION: Beta-ketoacyl-coenzyme A reductase required for the elongation of fatty acids precursors of sphingolipids, triacylglycerols, cuticular waxes and suberin. Responsible for the first reduction step in very long-chain fatty acids (VLCFAs) synthesis. Decreased expression of KCR1 (RNAi) leads to plants with fused vegetative and reproductive organs, and abnormal trichome, epidermal cell and root morphology. Cannot be complemented by KCR2. {ECO:0000269|PubMed:11792704, ECO:0000269|PubMed:19439572, ECO:0000269|PubMed:9342868}.	GO:0005739; GO:0005783; GO:0005789; GO:0009793; GO:0016021; GO:0018454; GO:0042761; GO:0045703; GO:0102339; GO:0102340; GO:0102341; GO:0102342	embryo development ending in seed dormancy [GO:0009793]; very long-chain fatty acid biosynthetic process [GO:0042761]

Pavir.4KG123600	Pavir.4KG123600.v5.1	Chr04K	16859600	16859722	(1 of 55) PF08137 - DVL family (DVL)	#N/A	#N/A	#N/A	#N/A	#N/A	#N/A	#N/A
Pavir.4KG123700	Pavir.4KG123700.v5.1	Chr04K	16872145	16873877	(1 of 42) PF03181 - BURP domain (BURP)	BURPA_ORYSJ	BURP domain-containing protein 10 (OsBURP10)	BURP10 Os06g0281800 LOC_Os06g17000 OsJ_20979 OSJNBa0063H02.22	Oryza sativa subsp. japonica (Rice)			
Pavir.4KG123900	Pavir.4KG123900.v5.1	Chr04K	16883417	16885808	(1 of 14) K10251 - 17beta-estradiol 17-dehydrogenase / very-long-chain 3-oxoacyl-CoA reductase (HSD17B12, KAR, IFA38)	KCR1_ARATH	Very-long-chain 3-oxoacyl-CoA reductase 1 (EC 1.1.1.330) (Beta-ketoacyl reductase 1) (AtKCR1) (Protein GLOSSY 8) (gl8At)	KCR1 At-YBR159 GL8 At1g67730 F12A21.31	Arabidopsis thaliana (Mouse-ear cress)	FUNCTION: Beta-ketoacyl-coenzyme A reductase required for the elongation of fatty acids precursors of sphingolipids, triacylglycerols, cuticular waxes and suberin. Responsible for the first reduction step in very long-chain fatty acids (VLCFAs) synthesis. Decreased expression of KCR1 (RNAi) leads to plants with fused vegetative and reproductive organs, and abnormal trichome, epidermal cell and root morphology. Cannot be complemented by KCR2. {ECO:0000269|PubMed:11792704, ECO:0000269|PubMed:19439572, ECO:0000269|PubMed:9342868}.	GO:0005739; GO:0005783; GO:0005789; GO:0009793; GO:0016021; GO:0018454; GO:0042761; GO:0045703; GO:0102339; GO:0102340; GO:0102341; GO:0102342	embryo development ending in seed dormancy [GO:0009793]; very long-chain fatty acid biosynthetic process [GO:0042761]
Pavir.4KG124000	Pavir.4KG124000.v5.1	Chr04K	16913940	16915301	(1 of 42) PF03181 - BURP domain (BURP)	BURPA_ORYSJ	BURP domain-containing protein 10 (OsBURP10)	BURP10 Os06g0281800 LOC_Os06g17000 OsJ_20979 OSJNBa0063H02.22	Oryza sativa subsp. japonica (Rice)			
Pavir.4KG124100	Pavir.4KG124100.v5.1	Chr04K	16932855	16935129	(1 of 14) K10251 - 17beta-estradiol 17-dehydrogenase / very-long-chain 3-oxoacyl-CoA reductase (HSD17B12, KAR, IFA38)	KCR1_ARATH	Very-long-chain 3-oxoacyl-CoA reductase 1 (EC 1.1.1.330) (Beta-ketoacyl reductase 1) (AtKCR1) (Protein GLOSSY 8) (gl8At)	KCR1 At-YBR159 GL8 At1g67730 F12A21.31	Arabidopsis thaliana (Mouse-ear cress)	FUNCTION: Beta-ketoacyl-coenzyme A reductase required for the elongation of fatty acids precursors of sphingolipids, triacylglycerols, cuticular waxes and suberin. Responsible for the first reduction step in very long-chain fatty acids (VLCFAs) synthesis. Decreased expression of KCR1 (RNAi) leads to plants with fused vegetative and reproductive organs, and abnormal trichome, epidermal cell and root morphology. Cannot be complemented by KCR2. {ECO:0000269|PubMed:11792704, ECO:0000269|PubMed:19439572, ECO:0000269|PubMed:9342868}.	GO:0005739; GO:0005783; GO:0005789; GO:0009793; GO:0016021; GO:0018454; GO:0042761; GO:0045703; GO:0102339; GO:0102340; GO:0102341; GO:0102342	embryo development ending in seed dormancy [GO:0009793]; very long-chain fatty acid biosynthetic process [GO:0042761]
Pavir.4KG124105	Pavir.4KG124105.v5.1	Chr04K	16941356	16941553		#N/A	#N/A	#N/A	#N/A	#N/A	#N/A	#N/A
Pavir.4KG059200	Pavir.4KG059200.v5.1	Chr04K	16953302	16954492	(1 of 42) PF03181 - BURP domain (BURP)	BURPA_ORYSJ	BURP domain-containing protein 10 (OsBURP10)	BURP10 Os06g0281800 LOC_Os06g17000 OsJ_20979 OSJNBa0063H02.22	Oryza sativa subsp. japonica (Rice)			
Pavir.4KG059205	Pavir.4KG059205.v5.1	Chr04K	16976762	16978297	(1 of 14) K10251 - 17beta-estradiol 17-dehydrogenase / very-long-chain 3-oxoacyl-CoA reductase (HSD17B12, KAR, IFA38)	KCR1_ARATH	Very-long-chain 3-oxoacyl-CoA reductase 1 (EC 1.1.1.330) (Beta-ketoacyl reductase 1) (AtKCR1) (Protein GLOSSY 8) (gl8At)	KCR1 At-YBR159 GL8 At1g67730 F12A21.31	Arabidopsis thaliana (Mouse-ear cress)	FUNCTION: Beta-ketoacyl-coenzyme A reductase required for the elongation of fatty acids precursors of sphingolipids, triacylglycerols, cuticular waxes and suberin. Responsible for the first reduction step in very long-chain fatty acids (VLCFAs) synthesis. Decreased expression of KCR1 (RNAi) leads to plants with fused vegetative and reproductive organs, and abnormal trichome, epidermal cell and root morphology. Cannot be complemented by KCR2. {ECO:0000269|PubMed:11792704, ECO:0000269|PubMed:19439572, ECO:0000269|PubMed:9342868}.	GO:0005739; GO:0005783; GO:0005789; GO:0009793; GO:0016021; GO:0018454; GO:0042761; GO:0045703; GO:0102339; GO:0102340; GO:0102341; GO:0102342	embryo development ending in seed dormancy [GO:0009793]; very long-chain fatty acid biosynthetic process [GO:0042761]
Pavir.4KG059210	Pavir.4KG059210.v5.1	Chr04K	17005770	17006400	(1 of 50) 2.7.7.49//2.7.7.7//3.1.26.4 - RNA-directed DNA polymerase / Revertase // DNA-directed DNA polymerase / DNA-dependent DNA polymerase // Ribonuclease H / RNase H	#N/A	#N/A	#N/A	#N/A	#N/A	#N/A	#N/A
Pavir.4KG059215	Pavir.4KG059215.v5.1	Chr04K	17022298	17023829	(1 of 42) PF03181 - BURP domain (BURP)	BURPA_ORYSJ	BURP domain-containing protein 10 (OsBURP10)	BURP10 Os06g0281800 LOC_Os06g17000 OsJ_20979 OSJNBa0063H02.22	Oryza sativa subsp. japonica (Rice)			
Pavir.4KG059220	Pavir.4KG059220.v5.1	Chr04K	17041350	17043375	(1 of 14) K10251 - 17beta-estradiol 17-dehydrogenase / very-long-chain 3-oxoacyl-CoA reductase (HSD17B12, KAR, IFA38)	KCR1_ARATH	Very-long-chain 3-oxoacyl-CoA reductase 1 (EC 1.1.1.330) (Beta-ketoacyl reductase 1) (AtKCR1) (Protein GLOSSY 8) (gl8At)	KCR1 At-YBR159 GL8 At1g67730 F12A21.31	Arabidopsis thaliana (Mouse-ear cress)	FUNCTION: Beta-ketoacyl-coenzyme A reductase required for the elongation of fatty acids precursors of sphingolipids, triacylglycerols, cuticular waxes and suberin. Responsible for the first reduction step in very long-chain fatty acids (VLCFAs) synthesis. Decreased expression of KCR1 (RNAi) leads to plants with fused vegetative and reproductive organs, and abnormal trichome, epidermal cell and root morphology. Cannot be complemented by KCR2. {ECO:0000269|PubMed:11792704, ECO:0000269|PubMed:19439572, ECO:0000269|PubMed:9342868}.	GO:0005739; GO:0005783; GO:0005789; GO:0009793; GO:0016021; GO:0018454; GO:0042761; GO:0045703; GO:0102339; GO:0102340; GO:0102341; GO:0102342	embryo development ending in seed dormancy [GO:0009793]; very long-chain fatty acid biosynthetic process [GO:0042761]

Pavir.4KG124200	Pavir.4KG124200.v5.1	Chr04K	17082954	17084314	(1 of 42) PF03181 - BURP domain (BURP)	BURPA_ORYSJ	BURP domain-containing protein 10 (OsBURP10)	BURP10 Os06g0281800 LOC_Os06g17000 OsJ_20979 OSJNBa0063H02.22	Oryza sativa subsp. japonica (Rice)			
Pavir.4KG124300	Pavir.4KG124300.v5.1	Chr04K	17091494	17093275	(1 of 14) K10251 - 17beta-estradiol 17-dehydrogenase / very-long-chain 3-oxoacyl-CoA reductase (HSD17B12, KAR, IFA38)	KCR1_ARATH	Very-long-chain 3-oxoacyl-CoA reductase 1 (EC 1.1.1.330) (Beta-ketoacyl reductase 1) (AtKCR1) (Protein GLOSSY 8) (gl8At)	KCR1 At-YBR159 GL8 At1g67730 F12A21.31	Arabidopsis thaliana (Mouse-ear cress)	FUNCTION: Beta-ketoacyl-coenzyme A reductase required for the elongation of fatty acids precursors of sphingolipids, triacylglycerols, cuticular waxes and suberin. Responsible for the first reduction step in very long-chain fatty acids (VLCFAs) synthesis. Decreased expression of KCR1 (RNAi) leads to plants with fused vegetative and reproductive organs, and abnormal trichome, epidermal cell and root morphology. Cannot be complemented by KCR2. {ECO:0000269|PubMed:11792704, ECO:0000269|PubMed:19439572, ECO:0000269|PubMed:9342868}.	GO:0005739; GO:0005783; GO:0005789; GO:0009793; GO:0016021; GO:0018454; GO:0042761; GO:0045703; GO:0102339; GO:0102340; GO:0102341; GO:0102342	embryo development ending in seed dormancy [GO:0009793]; very long-chain fatty acid biosynthetic process [GO:0042761]
Pavir.4KG124400	Pavir.4KG124400.v5.1	Chr04K	17111719	17113177	(1 of 65) PF00314 - Thaumatin family (Thaumatin)	OLP1_SOLLC	Osmotin-like protein		Solanum lycopersicum (Tomato) (Lycopersicon esculentum)		GO:0005576; GO:0005618; GO:0006952	defense response [GO:0006952]
Pavir.4KG124405	Pavir.4KG124405.v5.1	Chr04K	17133817	17134274	(1 of 10) PTHR10516//PTHR10516:SF324 - PEPTIDYL-PROLYL CIS-TRANS ISOMERASE // PEPTIDYL-PROLYL CIS-TRANS ISOMERASE FKBP62	FKB62_ARATH	Peptidyl-prolyl cis-trans isomerase FKBP62 (PPIase FKBP62) (EC 5.2.1.8) (70 kDa peptidyl-prolyl isomerase) (FK506-binding protein 62) (AtFKBP62) (Immunophilin FKBP62) (Peptidylprolyl isomerase ROF1) (Protein ROTAMASE FKBP 1) (Rotamase)	FKBP62 ROF1 At3g25230 MJL12.19	Arabidopsis thaliana (Mouse-ear cress)	FUNCTION: PPIases accelerate the folding of proteins. It catalyzes the cis-trans isomerization of proline imidic peptide bonds in oligopeptides (By similarity). Co-chaperone that positively modulates thermotolerance by interacting with HSP90.1 and increasing the HSFA2-mediated accumulation of chaperones of the small-HSPs family. {ECO:0000250, ECO:0000269|PubMed:17080288, ECO:0000269|PubMed:19366428}.	GO:0000413; GO:0003755; GO:0005516; GO:0005634; GO:0005737; GO:0061077; GO:0070370	cellular heat acclimation [GO:0070370]; chaperone-mediated protein folding [GO:0061077]; protein peptidyl-prolyl isomerization [GO:0000413]
Pavir.4KG124410	Pavir.4KG124410.v5.1	Chr04K	17135385	17142207		#N/A	#N/A	#N/A	#N/A	#N/A	#N/A	#N/A
Pavir.4KG124415	Pavir.4KG124415.v5.1	Chr04K	17137896	17138202		#N/A	#N/A	#N/A	#N/A	#N/A	#N/A	#N/A
Pavir.4KG124900	Pavir.4KG124900.v5.1	Chr04K	17146919	17149773		#N/A	#N/A	#N/A	#N/A	#N/A	#N/A	#N/A
Pavir.4KG125000	Pavir.4KG125000.v5.1	Chr04K	17157595	17158056	(1 of 119) PF13912 - C2H2-type zinc finger (zf-C2H2_6)	ZAT8_ARATH	Zinc finger protein ZAT8	ZAT8 At3g46080 F12M12.50	Arabidopsis thaliana (Mouse-ear cress)	FUNCTION: Probable transcription factor that may be involved in stress responses. {ECO:0000250}.	GO:0003700; GO:0005634; GO:0006355; GO:0010200; GO:0046872; GO:0071456	cellular response to hypoxia [GO:0071456]; regulation of transcription, DNA-templated [GO:0006355]; response to chitin [GO:0010200]
Pavir.4KG141300	Pavir.4KG141300.v5.1	Chr04K	17185795	17189037	(1 of 119) PF13912 - C2H2-type zinc finger (zf-C2H2_6)	ZAT7_ARATH	Zinc finger protein ZAT7	ZAT7 At3g46090 F12M12.60	Arabidopsis thaliana (Mouse-ear cress)	FUNCTION: Probable transcription factor involved in oxidative stress response. {ECO:0000269|PubMed:14722088}.	GO:0003700; GO:0005634; GO:0006355; GO:0006979; GO:0010200; GO:0045926; GO:0046872; GO:0071456	cellular response to hypoxia [GO:0071456]; negative regulation of growth [GO:0045926]; regulation of transcription, DNA-templated [GO:0006355]; response to chitin [GO:0010200]; response to oxidative stress [GO:0006979]
Pavir.4KG141305	Pavir.4KG141305.v5.1	Chr04K	17245305	17245679		#N/A	#N/A	#N/A	#N/A	#N/A	#N/A	#N/A
Pavir.4KG141310	Pavir.4KG141310.v5.1	Chr04K	17245958	17247075	(1 of 4) PTHR23024//PTHR23024:SF137 - MEMBER OF 'GDXG' FAMILY OF LIPOLYTIC ENZYMES // SUBFAMILY NOT NAMED	TCEA1_TULGE	Tuliposide A-converting enzyme 1, chloroplastic (TgTCEA1) (EC 4.2.99.22)	TCEA1 TCEA5	Tulipa gesneriana (Garden tulip)	FUNCTION: Lactone-forming carboxylesterases, specifically catalyzing intramolecular transesterification, but not hydrolysis. Involved in the biosynthesis of tulipalins, defensive chemicals that show antimicrobial activities against a broad range of strains of bacteria and fungi. Substrates are 6-tuliposide A > 6-tuliposide B. {ECO:0000269|PubMed:22474185}.	GO:0006952; GO:0009507; GO:0016787; GO:0016829	defense response [GO:0006952]
Pavir.4KG235600	Pavir.4KG235600.v5.1	Chr04K	17266977	17268285	(1 of 14) PTHR13683//PTHR13683:SF368 - ASPARTYL PROTEASES // SUBFAMILY NOT NAMED	CLP_ORYSJ	Chitinase CLP (OsCLP) (EC 3.2.1.14) (Chitinase-like protein)	CLP Os01g0937050 LOC_Os01g71080 P0504E02.9	Oryza sativa subsp. japonica (Rice)	FUNCTION: Chitinase that possesses antifungal activity (PubMed:23331415). Inhibits the growth of the fungal pathogen Rhizoctonia solani by degrading the fungal cell wall (PubMed:23331415). Does not possess inhibiting activity against fungal endo-1,4-beta-D-xylanases belonging to glycoside hydrolase family 10 (GH10) and family 11 (GH11) (PubMed:23331415). Involved in the regulation of plant growth by regulating the intracellular calcium ion concentration in roots (PubMed:28401568). {ECO:0000269|PubMed:23331415, ECO:0000269|PubMed:28401568}.	GO:0000272; GO:0004190; GO:0006032; GO:0008843; GO:0040008; GO:0048046; GO:0048364; GO:0048367; GO:0050832	chitin catabolic process [GO:0006032]; defense response to fungus [GO:0050832]; polysaccharide catabolic process [GO:0000272]; regulation of growth [GO:0040008]; root development [GO:0048364]; shoot system development [GO:0048367]
Pavir.4KG235400	Pavir.4KG235400.v5.1	Chr04K	17278675	17279846	(1 of 7) PTHR11177:SF214 - ACIDIC ENDOCHITINASE	XIP1_ORYSJ	Xylanase inhibitor protein 1 (XIP-I) (Class III chitinase homolog a) (RIXI protein)	RIXI Chib3H-a Os11g0701800 LOC_Os11g47580 OsJ_34838	Oryza sativa subsp. japonica (Rice)	FUNCTION: Fungal xylanase inhibitor. Possesses competitive inhibiting activity against fungal endo-1,4-beta-D-xylanases belonging to glycoside hydrolase family 11 (GH11). May function in plant defense against secreted fungal pathogen xylanases. Is similar to class III chitinases, but does not exhibit chitinase activity. {ECO:0000269|PubMed:15794761}.	GO:0004568; GO:0004857; GO:0005576; GO:0005975; GO:0050832	carbohydrate metabolic process [GO:0005975]; defense response to fungus [GO:0050832]
Pavir.4KG235405	Pavir.4KG235405.v5.1	Chr04K	17283275	17284584		#N/A	#N/A	#N/A	#N/A	#N/A	#N/A	#N/A
Pavir.4KG235410	Pavir.4KG235410.v5.1	Chr04K	17284969	17286664	(1 of 33) PTHR25952:SF191 - PROTEIN T12G3.2, ISOFORM D	#N/A	#N/A	#N/A	#N/A	#N/A	#N/A	#N/A
Pavir.4KG149000	Pavir.4KG149000.v5.1	Chr04K	17291596	17298863	(1 of 3) PTHR13301//PTHR13301:SF53 - X-BOX TRANSCRIPTION FACTOR-RELATED // CELLULOSE SYNTHASE-LIKE PROTEIN B1-RELATED	#N/A	#N/A	#N/A	#N/A	#N/A	#N/A	#N/A
Pavir.4KG149005	Pavir.4KG149005.v5.1	Chr04K	17299710	17300917		#N/A	#N/A	#N/A	#N/A	#N/A	#N/A	#N/A
Pavir.4KG149010	Pavir.4KG149010.v5.1	Chr04K	17336388	17341305		#N/A	#N/A	#N/A	#N/A	#N/A	#N/A	#N/A
Pavir.4KG149015	Pavir.4KG149015.v5.1	Chr04K	17341440	17342935		#N/A	#N/A	#N/A	#N/A	#N/A	#N/A	#N/A
Pavir.4KG149200	Pavir.4KG149200.v5.1	Chr04K	17362469	17364612	(1 of 108) PF00069//PF00139 - Protein kinase domain (Pkinase) // Legume lectin domain (Lectin_legB)	#N/A	#N/A	#N/A	#N/A	#N/A	#N/A	#N/A
Pavir.4KG209700	Pavir.4KG209700.v5.1	Chr04K	17397493	17398181		#N/A	#N/A	#N/A	#N/A	#N/A	#N/A	#N/A
Pavir.4KG209800	Pavir.4KG209800.v5.1	Chr04K	17410573	17411394		#N/A	#N/A	#N/A	#N/A	#N/A	#N/A	#N/A

Pavir.4KG209900	Pavir.4KG209900.v5.1	Chr04K	17422414	17425237	(1 of 7) K14009 - B-cell receptor-associated protein 31 (BCAP31, BAP31)	#N/A	#N/A	#N/A	#N/A	#N/A	#N/A	#N/A
Pavir.4KG134800	Pavir.4KG134800.v5.1	Chr04K	17432725	17435218	(1 of 2) PF02810 - SEC-C motif (SEC-C)	SECA_CALS8	Protein translocase subunit SecA	secA Csac_1325	Caldicellulosiruptor saccharolyticus (strain ATCC 43494 / DSM 8903 / Tp8T 6331)	FUNCTION: Part of the Sec protein translocase complex. Interacts with the SecYEG preprotein conducting channel. Has a central role in coupling the hydrolysis of ATP to the transfer of proteins into and across the cell membrane, serving as an ATP-driven molecular motor driving the stepwise translocation of polypeptide chains across the membrane. {ECO:0000255|HAMAP-Rule:MF 01382}.	GO:0005524; GO:0005737; GO:0005886; GO:0006605; GO:0017038; GO:0046872; GO:0065002	intracellular protein transmembrane transport [GO:0065002]; protein import [GO:0017038]; protein targeting [GO:0006605]
Pavir.4KG134805	Pavir.4KG134805.v5.1	Chr04K	17437636	17439832	(1 of 49) PTHR11260//PTHR11260:SF120 - GLUTATHIONE S-TRANSFERASE, GST, SUPERFAMILY, GST DOMAIN CONTAINING // SUBFAMILY NOT NAMED	GSTT3_ARATH	Glutathione S-transferase T3 (AtGSTT3) (EC 2.5.1.18) (GST class-theta member 3) (Glutathione S-transferase 10C)	GSTT3 GST10C At5g41220 K1O13.1	Arabidopsis thaliana (Mouse-ear cress)	FUNCTION: May be involved in the conjugation of reduced glutathione to a wide number of exogenous and endogenous hydrophobic electrophiles and have a detoxification role against certain herbicides. {ECO:0000250}.	GO:0004364; GO:0005634; GO:0005737; GO:0009407; GO:0009536	toxin catabolic process [GO:0009407]
Pavir.4KG134810	Pavir.4KG134810.v5.1	Chr04K	17440274	17440672		#N/A	#N/A	#N/A	#N/A	#N/A	#N/A	#N/A
Pavir.4KG134200	Pavir.4KG134200.v5.1	Chr04K	17441346	17449421	(1 of 20) PF00397 - WW domain (WW)	FNBP4_MOUSE	Formin-binding protein 4 (Formin-binding protein 30)	Fnbp4 Fbp30 Kiaa1014	Mus musculus (Mouse)		GO:0016607	
Pavir.4KG134100	Pavir.4KG134100.v5.1	Chr04K	17455359	17460959	(1 of 16) PF00643//PF06203 - B-box zinc finger (zf-B_box) // CCT motif (CCT)	COL9_ARATH	Zinc finger protein CONSTANS-LIKE 9	COL9 At3g07650 MLP3.10	Arabidopsis thaliana (Mouse-ear cress)		GO:0000976; GO:0003700; GO:0005634; GO:0006355; GO:0007623; GO:0008270; GO:0048579	circadian rhythm [GO:0007623]; negative regulation of long-day photoperiodism, flowering [GO:0048579]; regulation of transcription, DNA-templated [GO:0006355]
Pavir.4KG134000	Pavir.4KG134000.v5.1	Chr04K	17460960	17461730		#N/A	#N/A	#N/A	#N/A	#N/A	#N/A	#N/A
Pavir.4KG133900	Pavir.4KG133900.v5.1	Chr04K	17467884	17474867	(1 of 2) PTHR10774//PTHR10774:SF90 - EXTENDED SYNAPTOTAGMIN-RELATED // SUBFAMILY NOT NAMED	C2GR1_ARATH	C2 and GRAM domain-containing protein At1g03370	At1g03370 F15K9.2	Arabidopsis thaliana (Mouse-ear cress)		GO:0016021; GO:0046872	
Pavir.4KG133800	Pavir.4KG133800.v5.1	Chr04K	17491101	17494067	(1 of 2) PTHR21716:SF4 - TRANSMEMBRANE PROTEIN 245	TM245_RAT	Transmembrane protein 245	Tmem245	Rattus norvegicus (Rat)		GO:0016021	
Pavir.4KG133700	Pavir.4KG133700.v5.1	Chr04K	17508614	17513108	(1 of 4) PTHR11132:SF38 - GB|AAF04433.1	NSTU2_ARATH	Nucleotide-sugar uncharacterized transporter 2	At5g55950 MYN21.6	Arabidopsis thaliana (Mouse-ear cress)		GO:0005338; GO:0005464; GO:0005794; GO:0008643; GO:0015297; GO:0016021; GO:0022857	carbohydrate transport [GO:0008643]
Pavir.4KG133600	Pavir.4KG133600.v5.1	Chr04K	17513207	17516009	(1 of 139) PF13962 - Domain of unknown function (PGG)	#N/A	#N/A	#N/A	#N/A	#N/A	#N/A	#N/A
Pavir.4KG133500	Pavir.4KG133500.v5.1	Chr04K	17530904	17534434	(1 of 139) PF13962 - Domain of unknown function (PGG)	#N/A	#N/A	#N/A	#N/A	#N/A	#N/A	#N/A
Pavir.4KG133400	Pavir.4KG133400.v5.1	Chr04K	17535127	17535951		#N/A	#N/A	#N/A	#N/A	#N/A	#N/A	#N/A
Pavir.4KG133405	Pavir.4KG133405.v5.1	Chr04K	17596493	17598145		#N/A	#N/A	#N/A	#N/A	#N/A	#N/A	#N/A
Pavir.4KG139100	Pavir.4KG139100.v5.1	Chr04K	17614085	17617020	(1 of 42) PF03181 - BURP domain (BURP)	BURPB_ORYSJ	BURP domain-containing protein 11 (OsBURP11)	BURP11 Os06g0302000 LOC_Os06g19800 OJ1217_C01.19 OSJNBa0042E12.9	Oryza sativa subsp. japonica (Rice)			
Pavir.4KG114000	Pavir.4KG114000.v5.1	Chr04K	17631498	17632741		#N/A	#N/A	#N/A	#N/A	#N/A	#N/A	#N/A
Pavir.4KG134300	Pavir.4KG134300.v5.1	Chr04K	17641111	17642361	(1 of 29) PF05078 - Protein of unknown function (DUF679) (DUF679)	DMP5_ARATH	Protein DMP5 (AtDMP5)	DMP5 At3g02430 F16B3.6	Arabidopsis thaliana (Mouse-ear cress)	FUNCTION: Involved in membrane remodeling. {ECO:0000250|UniProtKB:Q9LVF4}.	GO:0005783; GO:0005789; GO:0010256; GO:0016021	endomembrane system organization [GO:0010256]
Pavir.4KG134305	Pavir.4KG134305.v5.1	Chr04K	17665073	17665810		#N/A	#N/A	#N/A	#N/A	#N/A	#N/A	#N/A
Pavir.4KG133100	Pavir.4KG133100.v5.1	Chr04K	17670315	17671149		#N/A	#N/A	#N/A	#N/A	#N/A	#N/A	#N/A
Pavir.4KG133000	Pavir.4KG133000.v5.1	Chr04K	17671857	17675121	(1 of 139) PF13962 - Domain of unknown function (PGG)	#N/A	#N/A	#N/A	#N/A	#N/A	#N/A	#N/A
Pavir.4KG133005	Pavir.4KG133005.v5.1	Chr04K	17691899	17692578		#N/A	#N/A	#N/A	#N/A	#N/A	#N/A	#N/A
Pavir.4KG132900	Pavir.4KG132900.v5.1	Chr04K	17693246	17697523	(1 of 139) PF13962 - Domain of unknown function (PGG)	#N/A	#N/A	#N/A	#N/A	#N/A	#N/A	#N/A
Pavir.4KG132700	Pavir.4KG132700.v5.1	Chr04K	17717731	17718984		#N/A	#N/A	#N/A	#N/A	#N/A	#N/A	#N/A
Pavir.4KG132600	Pavir.4KG132600.v5.1	Chr04K	17718789	17722841	(1 of 139) PF13962 - Domain of unknown function (PGG)	#N/A	#N/A	#N/A	#N/A	#N/A	#N/A	#N/A
Pavir.4KG132605	Pavir.4KG132605.v5.1	Chr04K	17725526	17726628		#N/A	#N/A	#N/A	#N/A	#N/A	#N/A	#N/A
Pavir.4KG132500	Pavir.4KG132500.v5.1	Chr04K	17734843	17735653		#N/A	#N/A	#N/A	#N/A	#N/A	#N/A	#N/A
Pavir.4KG132400	Pavir.4KG132400.v5.1	Chr04K	17738881	17743437	(1 of 139) PF13962 - Domain of unknown function (PGG)	#N/A	#N/A	#N/A	#N/A	#N/A	#N/A	#N/A
Pavir.4KG132300	Pavir.4KG132300.v5.1	Chr04K	17750292	17754040	(1 of 3) PTHR24351//PTHR24351:SF77 - RIBOSOMAL PROTEIN S6 KINASE // SUBFAMILY NOT NAMED	G11A_ORYSI	Protein kinase G11A (EC 2.7.11.1)	OsI_021818	Oryza sativa subsp. indica (Rice)	FUNCTION: May play a role in the regulation of metabolism and signal transduction processes.	GO:0004674; GO:0005524	

Pavir.4KG132200	Pavir.4KG132200.v5.1	Chr04K	17757059	17759044	(1 of 2) PTHR31964:SF10 - ADENINE NUCLEOTIDE ALPHA HYDROLASES-LIKE SUPERFAMILY PROTEIN	PUB35_ARATH	U-box domain-containing protein 35 (Plant U-box protein 35) [Includes: E3 ubiquitin ligase (EC 2.3.2.27) (RING-type E3 ubiquitin transferase); Serine/threonine-protein kinase (EC 2.7.11.-)]	PUB35 At4g25160 F13M23.300 F24A6.13	Arabidopsis thaliana (Mouse-ear cress)	FUNCTION: Functions as an E3 ubiquitin ligase. {ECO:0000250}.	GO:0004674; GO:0004842; GO:0005524	
Pavir.4KG132100	Pavir.4KG132100.v5.1	Chr04K	17770370	17770828	(1 of 19) 3.1.11.2 - Exodeoxyribonuclease III / Exonuclease III	#N/A	#N/A	#N/A	#N/A	#N/A	#N/A	#N/A
Pavir.4KG132000	Pavir.4KG132000.v5.1	Chr04K	17775980	17776828		#N/A	#N/A	#N/A	#N/A	#N/A	#N/A	#N/A
Pavir.4KG131900	Pavir.4KG131900.v5.1	Chr04K	17852879	17859781	(1 of 1) K12119 - cryptochrome 2 (CRY2)	#N/A	#N/A	#N/A	#N/A	#N/A	#N/A	#N/A
Pavir.4KG131905	Pavir.4KG131905.v5.1	Chr04K	17878890	17879732		#N/A	#N/A	#N/A	#N/A	#N/A	#N/A	#N/A
Pavir.4KG131700	Pavir.4KG131700.v5.1	Chr04K	17882792	17884692	(1 of 2) PTHR11926//PTHR11926:SF352 - GLUCOSYL/GLUCURONOSYL TRANSFERASES // SUBFAMILY NOT NAMED	#N/A	#N/A	#N/A	#N/A	#N/A	#N/A	#N/A
Pavir.4KG131705	Pavir.4KG131705.v5.1	Chr04K	17912034	17912765		#N/A	#N/A	#N/A	#N/A	#N/A	#N/A	#N/A
Pavir.4KG131710	Pavir.4KG131710.v5.1	Chr04K	17915088	17916840	(1 of 80) 2.7.10.1//2.7.11.1 - Receptor protein-tyrosine kinase / Receptor protein tyrosine kinase // Non-specific serine/threonine protein kinase / Threonine-specific protein kinase	SBIR1_ARATH	Leucine-rich repeat receptor-like serine/threonine/tyrosine-protein kinase SOBIR1 (EC 2.7.10.1) (EC 2.7.11.1) (Protein EVERSHED) (Protein SUPPRESSOR OF BIR1-1)	SOBIR1 EVR At2g31880 F20M17.8	Arabidopsis thaliana (Mouse-ear cress)	FUNCTION: Dual specificity kinase acting on both serine/threonine- and tyrosine-containing substrates. Acting as a counterplayer of BIR1, promotes the activation of plant defense and cell death (PubMed:19616764). Component of the RLP23-SOBIR1-BAK1 complex that mediates NLP-triggered immunity (PubMed:27251392). Functions as an inhibitor/regulator of abscission, probably by regulating membrane trafficking during abscission (PubMed:20081191). {ECO:0000269|PubMed:19616764, ECO:0000269|PubMed:20081191, ECO:0000269|PubMed:27251392}.	GO:0004674; GO:0004713; GO:0004714; GO:0005524; GO:0005777; GO:0005886; GO:0006952; GO:0010942; GO:0016021; GO:0031349; GO:0060862	defense response [GO:0006952]; negative regulation of floral organ abscission [GO:0060862]; positive regulation of cell death [GO:0010942]; positive regulation of defense response [GO:0031349]
Pavir.4KG131715	Pavir.4KG131715.v5.1	Chr04K	17919314	17920106		#N/A	#N/A	#N/A	#N/A	#N/A	#N/A	#N/A
Pavir.4KG149400	Pavir.4KG149400.v5.1	Chr04K	17926626	17928581	(1 of 80) 2.7.10.1//2.7.11.1 - Receptor protein-tyrosine kinase / Receptor protein tyrosine kinase // Non-specific serine/threonine protein kinase / Threonine-specific protein kinase	SBIR1_ARATH	Leucine-rich repeat receptor-like serine/threonine/tyrosine-protein kinase SOBIR1 (EC 2.7.10.1) (EC 2.7.11.1) (Protein EVERSHED) (Protein SUPPRESSOR OF BIR1-1)	SOBIR1 EVR At2g31880 F20M17.8	Arabidopsis thaliana (Mouse-ear cress)	FUNCTION: Dual specificity kinase acting on both serine/threonine- and tyrosine-containing substrates. Acting as a counterplayer of BIR1, promotes the activation of plant defense and cell death (PubMed:19616764). Component of the RLP23-SOBIR1-BAK1 complex that mediates NLP-triggered immunity (PubMed:27251392). Functions as an inhibitor/regulator of abscission, probably by regulating membrane trafficking during abscission (PubMed:20081191). {ECO:0000269|PubMed:19616764, ECO:0000269|PubMed:20081191, ECO:0000269|PubMed:27251392}.	GO:0004674; GO:0004713; GO:0004714; GO:0005524; GO:0005777; GO:0005886; GO:0006952; GO:0010942; GO:0016021; GO:0031349; GO:0060862	defense response [GO:0006952]; negative regulation of floral organ abscission [GO:0060862]; positive regulation of cell death [GO:0010942]; positive regulation of defense response [GO:0031349]
Pavir.4KG131600	Pavir.4KG131600.v5.1	Chr04K	17967410	17970492	(1 of 5) PTHR23155//PTHR23155:SF556 - LEUCINE-RICH REPEAT-CONTAINING PROTEIN // SUBFAMILY NOT NAMED	PIKS2_ORYSJ	Disease resistance protein Piks-2 (NBS-LRR class disease resistance protein Piks-2)	PIKS-2	Oryza sativa subsp. japonica (Rice)	FUNCTION: Disease resistance (R) protein. Resistance proteins guard the plant against pathogens that contain an appropriate avirulence protein via an indirect interaction with this avirulence protein. That triggers a defense system including the hypersensitive response, which restricts the pathogen growth. {ECO:0000250|UniProtKB:P0DO07}.	GO:0005524; GO:0006952; GO:0043531	defense response [GO:0006952]
Pavir.4KG131500	Pavir.4KG131500.v5.1	Chr04K	17988263	17995281	(1 of 4) PTHR10366//PTHR10366:SF441 - NAD DEPENDENT EPIMERASE/DEHYDRATASE // ISOFLAVONE REDUCTASE HOMOLOG P3-RELATED	PYRC5_PYRCO	Phenylcoumaran benzylic ether reductase Pyrc5 (EC 1.23.1.-) (Minor fruit allergen Pyr c 5) (allergen Pyr c 5)	PYRC5	Pyrus communis (Pear) (Pyrus domestica)	FUNCTION: Oxidoreductase involved in lignan biosynthesis (PubMed:11606193). Catalyzes the NADPH-dependent reduction of phenylcoumaran benzylic ethers (PubMed:11606193). Converts dehydrodiconiferyl alcohol (DDC) to isodihydrodehydrodiconiferyl alcohol (IDDDC) (PubMed:11606193). {ECO:0000269|PubMed:11606193}.	GO:0009807; GO:0032442	lignan biosynthetic process [GO:0009807]
Pavir.4KG129100	Pavir.4KG129100.v5.1	Chr04K	18051817	18054264	(1 of 24) PF05627 - Cleavage site for pathogenic type III effector avirulence factor Avr (AvrRpt-cleavage)	NOI4_ARATH	Protein NOI4 (Protein DiDi 18T-1d)	NOI4 At5g55850 MWJ3.3	Arabidopsis thaliana (Mouse-ear cress)		GO:0005886; GO:0009624	response to nematode [GO:0009624]
Pavir.4KG129105	Pavir.4KG129105.v5.1	Chr04K	18098583	18101556	(1 of 38) PTHR23002//PTHR23002:SF63 - ZINC FINGER CCHC DOMAIN CONTAINING PROTEIN // SUBFAMILY NOT NAMED	#N/A	#N/A	#N/A	#N/A	#N/A	#N/A	#N/A

Pavir.4KG128900	Pavir.4KG128900.v5.1	Chr04K	18118712	18134283	(1 of 2) PTHR12663:SF0 - PDS5	PDS5B_MOUSE	Sister chromatid cohesion protein PDS5 homolog B (Androgen-induced proliferation inhibitor) (Androgen-induced prostate proliferative shutoff-associated protein AS3)	Pds5b Aprin As3 Kiaa0979	Mus musculus (Mouse)	FUNCTION: Regulator of sister chromatid cohesion in mitosis which may stabilize cohesin complex association with chromatin. May couple sister chromatid cohesion during mitosis to DNA replication. Cohesion ensures that chromosome partitioning is accurate in both meiotic and mitotic cells and plays an important role in DNA repair. Plays a role in androgen-induced proliferative arrest in prostate cells (By similarity). {ECO:0000250}.	GO:0000785; GO:0005634; GO:0005654; GO:0006281; GO:0007064; GO:0008285; GO:0042127; GO:0051301	cell division [GO:0051301]; DNA repair [GO:0006281]; mitotic sister chromatid cohesion [GO:0007064]; negative regulation of cell population proliferation [GO:0008285]; regulation of cell population proliferation [GO:0042127]
Pavir.4KG128800	Pavir.4KG128800.v5.1	Chr04K	18134541	18137272	(1 of 9) 1.14.11.33 - DNA oxidative demethylase / Alkylated DNA repair protein	ALKB2_ARATH	DNA oxidative demethylase ALKBH2 (EC 1.14.11.33) (Alkylated DNA repair protein alkB homolog 2) (Alpha-ketoglutarate-dependent dioxygenase alkB homolog 2)	ALKBH2 At2g22260 T26C19.8	Arabidopsis thaliana (Mouse-ear cress)	FUNCTION: Dioxygenase that repairs alkylated DNA containing 1-methyladenine and 1-ethenoadenine by oxidative demethylation. Accepts double-stranded and single-stranded substrates, with a preference for dsDNA over ssDNA. Confers resistance to methylating agents such as methylmethanesulphonate (MMS). {ECO:0000269|PubMed:22532610}.	GO:0005634; GO:0006281; GO:0006307; GO:0008198; GO:0035511; GO:0035514; GO:0043734; GO:0051747; GO:0103053	DNA dealkylation involved in DNA repair [GO:0006307]; DNA repair [GO:0006281]; oxidative DNA demethylation [GO:0035511]
Pavir.4KG128700	Pavir.4KG128700.v5.1	Chr04K	18138618	18140018	(1 of 127) PF02298 - Plastocyanin-like domain (Cu_bind_like)	ENL1_ARATH	Early nodulin-like protein 1 (Phytocyanin-like protein)	At2g25060 F27C12.2	Arabidopsis thaliana (Mouse-ear cress)		GO:0005773; GO:0005886; GO:0009055; GO:0009506; GO:0031225; GO:0046658; GO:0099503	
Pavir.4KG128705	Pavir.4KG128705.v5.1	Chr04K	18156734	18157389	(1 of 25) PTHR19446//PTHR19446:SF370 - REVERSE TRANSCRIPTASES // SUBFAMILY NOT NAMED	#N/A	#N/A	#N/A	#N/A	#N/A	#N/A	#N/A
Pavir.4KG128600	Pavir.4KG128600.v5.1	Chr04K	18162507	18164342	(1 of 139) PF13962 - Domain of unknown function (PGG)	#N/A	#N/A	#N/A	#N/A	#N/A	#N/A	#N/A
Pavir.4KG128500	Pavir.4KG128500.v5.1	Chr04K	18168169	18169601		#N/A	#N/A	#N/A	#N/A	#N/A	#N/A	#N/A
Pavir.4KG128400	Pavir.4KG128400.v5.1	Chr04K	18174073	18174573	(1 of 6) PTHR10159//PTHR10159:SF331 - DUAL SPECIFICITY PROTEIN PHOSPHATASE // SUBFAMILY NOT NAMED	IBR5_ARATH	Protein-tyrosine-phosphatase IBR5 (EC 3.1.3.48) (Protein INDOLE-3-BUTYRIC ACID RESPONSE 5) (Protein IBA RESPONSE 5) (SKP1-interacting partner 33)	IBR5 SKIP33 At2g04550 T1O3.4	Arabidopsis thaliana (Mouse-ear cress)	FUNCTION: Required for the transduction of auxin and abscisic acid (ABA) signaling pathways. Dephosphorylates and inactivates the MAP kinase MPK12. {ECO:0000269|PubMed:14630970, ECO:0000269|PubMed:18423007, ECO:0000269|PubMed:18832358, ECO:0000269|PubMed:19000167}.	GO:0004725; GO:0005634; GO:0008138; GO:0009733; GO:0009734; GO:0009737; GO:0009738; GO:0033549; GO:0035556; GO:0043407; GO:0046620; GO:0061388	abscisic acid-activated signaling pathway [GO:0009738]; auxin-activated signaling pathway [GO:0009734]; intracellular signal transduction [GO:0035556]; negative regulation of MAP kinase activity [GO:0043407]; regulation of organ growth [GO:0046620]; regulation of rate of cell growth [GO:0061388]; response to abscisic acid [GO:0009737]; response to auxin [GO:0009733]
Pavir.4KG128405	Pavir.4KG128405.v5.1	Chr04K	18192858	18193401		#N/A	#N/A	#N/A	#N/A	#N/A	#N/A	#N/A
Pavir.4KG128000	Pavir.4KG128000.v5.1	Chr04K	18226580	18227804	(1 of 139) PF13962 - Domain of unknown function (PGG)	#N/A	#N/A	#N/A	#N/A	#N/A	#N/A	#N/A
Pavir.4KG127900	Pavir.4KG127900.v5.1	Chr04K	18230129	18236086	(1 of 205) PF04578//PF13968 - Protein of unknown function, DUF594 (DUF594) // Domain of unknown function (DUF4220) (DUF4220)	#N/A	#N/A	#N/A	#N/A	#N/A	#N/A	#N/A
Pavir.4KG127800	Pavir.4KG127800.v5.1	Chr04K	18247613	18248830	(1 of 18) PTHR23155//PTHR23155:SF605 - LEUCINE-RICH REPEAT-CONTAINING PROTEIN // SUBFAMILY NOT NAMED	#N/A	#N/A	#N/A	#N/A	#N/A	#N/A	#N/A
Pavir.4KG120800	Pavir.4KG120800.v5.1	Chr04K	18288898	18291518	(1 of 3) PTHR27007//PTHR27007:SF21 - FAMILY NOT NAMED // L-TYPE LECTIN-DOMAIN CONTAINING RECEPTOR KINASE S.7-RELATED	LRKS7_ARATH	Probable L-type lectin-domain containing receptor kinase S.7 (LecRK-S.7) (EC 2.7.11.1)	LECRKS7 At5g55830 MDF20.27	Arabidopsis thaliana (Mouse-ear cress)	FUNCTION: Involved in resistance response to the pathogenic oomycetes Phytophthora infestans and Phytophthora capsici. {ECO:0000269|PubMed:25083911}.	GO:0002229; GO:0004675; GO:0005524; GO:0005886; GO:0006952; GO:0016021; GO:0030246; GO:0042742	defense response [GO:0006952]; defense response to bacterium [GO:0042742]; defense response to oomycetes [GO:0002229]
Pavir.4KG122100	Pavir.4KG122100.v5.1	Chr04K	18300580	18302293	(1 of 9) KOG1674 - Cyclin	CCU42_ARATH	Cyclin-U4-2 (CycU4;2) (Cyclin-P4.3) (CycP4;3)	CYCU4-2 At5g07450 T2I1.160	Arabidopsis thaliana (Mouse-ear cress)		GO:0000079; GO:0007049; GO:0019901; GO:0051301	cell cycle [GO:0007049]; cell division [GO:0051301]; regulation of cyclin-dependent protein serine/threonine kinase activity [GO:0000079]
Pavir.4KG122000	Pavir.4KG122000.v5.1	Chr04K	18309126	18310066	(1 of 6) PTHR11064:SF20 - NUCLEAR TRANSCRIPTION FACTOR Y SUBUNIT B-6-RELATED	NFYB6_ARATH	Nuclear transcription factor Y subunit B-6 (AtNF-YB-6) (Protein LEAFY COTYLEDON 1-LIKE)	NFYB6 L1L At5g47670 MNJ7.26	Arabidopsis thaliana (Mouse-ear cress)	FUNCTION: Component of the NF-Y/HAP transcription factor complex. The NF-Y complex stimulates the transcription of various genes by recognizing and binding to a CCAAT motif in promoters. Plays a role in the regulation of the embryogenesis. Involved in the abscisic acid (ABA) signaling pathway. {ECO:0000269|PubMed:12509518, ECO:0000269|PubMed:17322342}.	GO:0003700; GO:0005634; GO:0006355; GO:0009738; GO:0033613; GO:0043565; GO:0045893; GO:0046982	abscisic acid-activated signaling pathway [GO:0009738]; positive regulation of transcription, DNA-templated [GO:0045893]; regulation of transcription, DNA-templated [GO:0006355]
Pavir.4KG121900	Pavir.4KG121900.v5.1	Chr04K	18348202	18349705	(1 of 11) PTHR11064//PTHR11064:SF37 - CCAAT-BINDING TRANSCRIPTION FACTOR-RELATED // SUBFAMILY NOT NAMED	NFYB1_ARATH	Nuclear transcription factor Y subunit B-1 (AtNF-YB-1) (Transcriptional activator HAP3A)	NFYB1 HAP3A At2g38880 T7F6.5	Arabidopsis thaliana (Mouse-ear cress)	FUNCTION: Component of the NF-Y/HAP transcription factor complex. The NF-Y complex stimulates the transcription of various genes by recognizing and binding to a CCAAT motif in promoters.	GO:0005634; GO:0043565; GO:0045893; GO:0046982	positive regulation of transcription, DNA-templated [GO:0045893]

Pavir.4KG121800	Pavir.4KG121800.v5.1	Chr04K	18351201	18351647	(1 of 11) PTHR11064//PTHR11064:SF37 - CCAAT-BINDING TRANSCRIPTION FACTOR-RELATED // SUBFAMILY NOT NAMED	NFYB1_ARATH	Nuclear transcription factor Y subunit B-1 (AtNF-YB-1) (Transcriptional activator HAP3A)	NFYB1 HAP3A At2g38880 T7F6.5	Arabidopsis thaliana (Mouse-ear cress)	FUNCTION: Component of the NF-Y/HAP transcription factor complex. The NF-Y complex stimulates the transcription of various genes by recognizing and binding to a CCAAT motif in promoters.	GO:0005634; GO:0043565; GO:0045893; GO:0046982	positive regulation of transcription, DNA-templated [GO:0045893]
Pavir.4KG121700	Pavir.4KG121700.v5.1	Chr04K	18361869	18362360	(1 of 11) PTHR11064//PTHR11064:SF37 - CCAAT-BINDING TRANSCRIPTION FACTOR-RELATED // SUBFAMILY NOT NAMED	NFYB1_ARATH	Nuclear transcription factor Y subunit B-1 (AtNF-YB-1) (Transcriptional activator HAP3A)	NFYB1 HAP3A At2g38880 T7F6.5	Arabidopsis thaliana (Mouse-ear cress)	FUNCTION: Component of the NF-Y/HAP transcription factor complex. The NF-Y complex stimulates the transcription of various genes by recognizing and binding to a CCAAT motif in promoters.	GO:0005634; GO:0043565; GO:0045893; GO:0046982	positive regulation of transcription, DNA-templated [GO:0045893]
Pavir.4KG121705	Pavir.4KG121705.v5.1	Chr04K	18396442	18397966	(1 of 11) PTHR11064//PTHR11064:SF37 - CCAAT-BINDING TRANSCRIPTION FACTOR-RELATED // SUBFAMILY NOT NAMED	NFYB1_ARATH	Nuclear transcription factor Y subunit B-1 (AtNF-YB-1) (Transcriptional activator HAP3A)	NFYB1 HAP3A At2g38880 T7F6.5	Arabidopsis thaliana (Mouse-ear cress)	FUNCTION: Component of the NF-Y/HAP transcription factor complex. The NF-Y complex stimulates the transcription of various genes by recognizing and binding to a CCAAT motif in promoters.	GO:0005634; GO:0043565; GO:0045893; GO:0046982	positive regulation of transcription, DNA-templated [GO:0045893]
Pavir.4KG121500	Pavir.4KG121500.v5.1	Chr04K	18400986	18401432	(1 of 11) PTHR11064//PTHR11064:SF37 - CCAAT-BINDING TRANSCRIPTION FACTOR-RELATED // SUBFAMILY NOT NAMED	NFYB8_ARATH	Nuclear transcription factor Y subunit B-8 (AtNF-YB-8)	NFYB8 At2g37060 T2N18.18	Arabidopsis thaliana (Mouse-ear cress)	FUNCTION: Component of the NF-Y/HAP transcription factor complex. The NF-Y complex stimulates the transcription of various genes by recognizing and binding to a CCAAT motif in promoters.	GO:0003700; GO:0005634; GO:0043565; GO:0045893; GO:0046982	positive regulation of transcription, DNA-templated [GO:0045893]
Pavir.4KG121400	Pavir.4KG121400.v5.1	Chr04K	18407516	18409135	(1 of 5) PTHR24298:SF1 - CYTOCHROME P450, FAMILY 79, SUBFAMILY C, POLYPEPTIDE 1-RELATED	C79A1_SORBI	Tyrosine N-monooxygenase (EC 1.14.14.36) (Cytochrome P450 79A1) (Cytochrome P450Tyr)	CYP79A1 CYP79	Sorghum bicolor (Sorghum) (Sorghum vulgare)	FUNCTION: N-hydroxylase that converts L-tyrosine to p-hydroxyphenylacetaldehyde oxime. {ECO:0000269|PubMed:7937883}.	GO:0004497; GO:0005506; GO:0005789; GO:0010132; GO:0016021; GO:0016705; GO:0020037	dhurrin biosynthetic process [GO:0010132]
Pavir.4KG121300	Pavir.4KG121300.v5.1	Chr04K	18418877	18420534	(1 of 51) PF02469 - Fasciclin domain (Fasciclin)	FLA2_ARATH	Fasciclin-like arabinogalactan protein 2	FLA2 At4g12730 T20K18.80	Arabidopsis thaliana (Mouse-ear cress)	FUNCTION: May be a cell surface adhesion protein.	GO:0005774; GO:0005829; GO:0005886; GO:0031225; GO:0046658	
Pavir.4KG121200	Pavir.4KG121200.v5.1	Chr04K	18424651	18425592		#N/A	#N/A	#N/A	#N/A	#N/A	#N/A	#N/A
Pavir.4KG121100	Pavir.4KG121100.v5.1	Chr04K	18426907	18430635	(1 of 2) PTHR35502:SF2 - MOVEMENT PROTEIN BINDING PROTEIN 2C	MBP2C_TOBAC	Protein MICROTUBULE BINDING PROTEIN 2C (NtMBP2C) (Movement protein binding protein 2C) (TMV-MP30 binding protein 2C)	MBP2C LOC107762219	Nicotiana tabacum (Common tobacco)	FUNCTION: Prevents homeodomain proteins (e.g. STM) association to plasmodesmata and, consequently, cell-to-cell transport. Binds to RNA. Alters KN1 RNA-binding capacity (PubMed:17965274). Regulates cytoskeleton (e.g. actin) organization that determinates cell shape (By similarity). Interferes with cell-to-cell transport of tobacco mosaic virus movement protein (TMV-MP) by mediating its accumulation at microtubules, thus interfering with cell-to-cell virus movement. {ECO:0000250|UniProtKB:Q9LEZ4, ECO:0000269|PubMed:12913144, ECO:0000269|PubMed:17965274}.	GO:0002230; GO:0003723; GO:0005737; GO:0006952; GO:0008017; GO:0010497; GO:0015630; GO:0046740; GO:0051224; GO:0051493	defense response [GO:0006952]; negative regulation of protein transport [GO:0051224]; plasmodesmata-mediated intercellular transport [GO:0010497]; positive regulation of defense response to virus by host [GO:0002230]; regulation of cytoskeleton organization [GO:0051493]; transport of virus in host, cell to cell [GO:0046740]
Pavir.4KG121000	Pavir.4KG121000.v5.1	Chr04K	18468543	18469981	(1 of 65) PF00314 - Thaumatin family (Thaumatin)	THLP1_ARATH	Thaumatin-like protein 1 (AtTLP1)	TLP1 At4g24180 T19F6.2 T22A6.10	Arabidopsis thaliana (Mouse-ear cress)	FUNCTION: Involved in local responses of roots to colonization by non-pathogenic plant growth-promoting rhizobacteria (PGPR) fluorescent Pseudomonas spp., but seems to not being required for the establishment of subsequent induced systemic resistance (ISR). {ECO:0000269|PubMed:15988566}.	GO:0005576; GO:0006952; GO:0009617; GO:0009723	defense response [GO:0006952]; response to bacterium [GO:0009617]; response to ethylene [GO:0009723]
Pavir.4KG127600	Pavir.4KG127600.v5.1	Chr04K	18496015	18497102	(1 of 64) PF02701 - Dof domain, zinc finger (zf-Dof)	DOF4_ORYSJ	Dof zinc finger protein 4 (OsDof4)	DOF4 Os02g0726300 LOC_Os02g49440 B1121A12.10	Oryza sativa subsp. japonica (Rice)	FUNCTION: Transcription factor that may transactivate seed storage protein genes in developing seeds. {ECO:0000250|UniProtKB:Q6K537}.	GO:0003677; GO:0005634; GO:0006355; GO:0046872	regulation of transcription, DNA-templated [GO:0006355]
Pavir.4KG127605	Pavir.4KG127605.v5.1	Chr04K	18538997	18540976		#N/A	#N/A	#N/A	#N/A	#N/A	#N/A	#N/A
Pavir.4KG127610	Pavir.4KG127610.v5.1	Chr04K	18600285	18603716		#N/A	#N/A	#N/A	#N/A	#N/A	#N/A	#N/A
Pavir.4KG127615	Pavir.4KG127615.v5.1	Chr04K	18605878	18621191	(1 of 6) 5.4.2.2//5.4.2.8 - Phosphoglucomutase (alpha-D-glucose-1,6-bisphosphate-dependent) / Phosphoglucose mutase // Phosphomannomutase / Phosphomannose mutase	ALGC_PSEAE	Phosphomannomutase/phosphoglucomutase (PMM / PGM) (EC 5.4.2.2) (EC 5.4.2.8)	algC PA5322	Pseudomonas aeruginosa (strain ATCC 15692 / DSM 22644 / CIP 104116 / JCM 14847 / LMG 12228 / 1C / PRS 101 / PAO1)	FUNCTION: Highly reversible phosphoryltransferase. The phosphomannomutase activity produces a precursor for alginate polymerization, the alginate layer causes a mucoid phenotype and provides a protective barrier against host immune defenses and antibiotics. Also involved in core lipopolysaccaride (LPS) biosynthesis due to its phosphoglucomutase activity. Essential for rhamnolipid production, an exoproduct correlated with pathogenicity (PubMed:10481091). Required for biofilm production. The reaction proceeds via 2 processive phosphoryl transferase reactions; first from enzyme-phospho-Ser-108 to the substrate (generating a bisphosphorylated substrate intermediate and a dephosphorylated enzyme), a 180 degree rotation of the intermediate (probably aided by movement of domain 4), and subsequent transfer of phosphate back to the enzyme (PubMed:11716469, PubMed:16880541, PubMed:16595672, PubMed:22242625). {ECO:0000269|PubMed:10481091, ECO:0000269|PubMed:11716469, ECO:0000269|PubMed:16595672, ECO:0000269|PubMed:16880541, ECO:0000269|PubMed:18690721, ECO:0000269|PubMed:1903398, ECO:0000269|PubMed:22242625, ECO:0000269|PubMed:23517223, ECO:0000269|PubMed:7515870, ECO:0000269|PubMed:8050998}.	GO:0000287; GO:0004614; GO:0004615; GO:0009243; GO:0009244; GO:0009298; GO:0009405; GO:0042121	alginic acid biosynthetic process [GO:0042121]; GDP-mannose biosynthetic process [GO:0009298]; lipopolysaccharide core region biosynthetic process [GO:0009244]; O antigen biosynthetic process [GO:0009243]; pathogenesis [GO:0009405]

Pavir.4KG131200	Pavir.4KG131200.v5.1	Chr04K	18642745	18647407	(1 of 67) PF10250 - GDP-fucose protein O-fucosyltransferase (O-FucT)	RRT1_ARATH	Rhamnogalacturonan I rhamnosyltransferase 1 (EC 2.4.1.351) (O-fucosyltransferase 34) (O-FucT-34) (O-fucosyltransferase family protein)	RRT1 OFUT34 At5g15740 F14F8.120	Arabidopsis thaliana (Mouse-ear cress)	FUNCTION: Glycosyltransferase involved in the formation of rhamnogalacturonan I (RG-I) oligosaccharides in the seed coat mucilage, which is a specialized cell wall with abundant RG-I (PubMed:30082766). Transfers the rhamnose residue from UDP-beta-L-rhamnose to RG-I oligosaccharides (PubMed:30082766). Prefers RG-I oligosaccharides with a degree of polymerization of 5 or larger than 5 (PubMed:30082766). Does not act on oligosaccharides with a degree of polymerization of 4 or smaller than 4 (PubMed:30082766). Does not require metal ions for its activity (PubMed:30082766). {ECO:0000269|PubMed:30082766}.	GO:0000139; GO:0005737; GO:0006004; GO:0008194; GO:0010214; GO:0010395; GO:0016021; GO:0045489	fucose metabolic process [GO:0006004]; pectin biosynthetic process [GO:0045489]; rhamnogalacturonan I metabolic process [GO:0010395]; seed coat development [GO:0010214]
Pavir.4KG131205	Pavir.4KG131205.v5.1	Chr04K	18667093	18667629		#N/A	#N/A	#N/A	#N/A	#N/A	#N/A	#N/A
Pavir.4KG131000	Pavir.4KG131000.v5.1	Chr04K	18667630	18668081		#N/A	#N/A	#N/A	#N/A	#N/A	#N/A	#N/A
Pavir.4KG130800	Pavir.4KG130800.v5.1	Chr04K	18671290	18675705		MDA2_ARATH	Mediator-associated protein 2	At5g64680 MUB3.20	Arabidopsis thaliana (Mouse-ear cress)		GO:0005730; GO:0016592	
Pavir.4KG130700	Pavir.4KG130700.v5.1	Chr04K	18683892	18685491		#N/A	#N/A	#N/A	#N/A	#N/A	#N/A	#N/A
Pavir.4KG130600	Pavir.4KG130600.v5.1	Chr04K	18704488	18705991	(1 of 7) 2.7.7.2 - FAD synthetase / FMN adenylyltransferase	FAD1_SCHPO	Probable FAD synthase (EC 2.7.7.2) (FAD pyrophosphorylase) (FMN adenylyltransferase) (Flavin adenine dinucleotide synthase)	SPCC1235.04c	Schizosaccharomyces pombe (strain 972 / ATCC 24843) (Fission yeast)	FUNCTION: Adenylates FMN to FAD. {ECO:0000250}.	GO:0003919; GO:0005524; GO:0005634; GO:0005829; GO:0006747; GO:0046444	FAD biosynthetic process [GO:0006747]; FMN metabolic process [GO:0046444]
Pavir.4KG130500	Pavir.4KG130500.v5.1	Chr04K	18707477	18709977	(1 of 5) PTHR22765//PTHR22765:SF46 - RING FINGER AND PROTEASE ASSOCIATED DOMAIN-CONTAINING // SUBFAMILY NOT NAMED	RMR1_ORYSJ	Receptor homology region, transmembrane domain- and RING domain-containing protein 1 (OsRMR1)	Os03g0167500 LOC_Os03g07130 OSJNBa0091P11.36	Oryza sativa subsp. japonica (Rice)	FUNCTION: Involved in the trafficking of vacuolar proteins. Functions probably as a sorting receptor for protein trafficking to the protein storage vacuole (PSV) by binding the C-terminal vacuolar sorting determinant (VSD) of vacuolar-sorted proteins. {ECO:0000269|PubMed:21493745}.	GO:0000139; GO:0015031; GO:0016021; GO:0031902; GO:0032586; GO:0046872	protein transport [GO:0015031]
Pavir.4KG130400	Pavir.4KG130400.v5.1	Chr04K	18722564	18726553	(1 of 4) PTHR15245//PTHR15245:SF21 - SYMPLEKIN-RELATED // SUBFAMILY NOT NAMED	P4KG7_ARATH	Phosphatidylinositol 4-kinase gamma 7 (AtPI4Kgamma7) (PI-4Kgamma7) (PI4K gamma 7) (EC 2.7.1.67) (Ubiquitin-like domain kinase gamma 7) (UbDK gamma 7)	PI4KG7 PI4KGAMMA7 UBDKGAMMA7 At2g03890 T18C20.9	Arabidopsis thaliana (Mouse-ear cress)	FUNCTION: The phosphorylation of phosphatidylinositol (PI) to PI4P is the first committed step in the generation of phosphatidylinositol 4,5-bisphosphate (PIP2), a precursor of the second messenger inositol 1,4,5-trisphosphate (InsP3) (By similarity). Undergoes autophosphorylation and phosphorylates serine/threonine residues of protein substrates. {ECO:0000250, ECO:0000269|PubMed:17880284}.	GO:0004430; GO:0005524; GO:0046777; GO:0046854	phosphatidylinositol phosphorylation [GO:0046854]; protein autophosphorylation [GO:0046777]
Pavir.4KG130300	Pavir.4KG130300.v5.1	Chr04K	18727034	18737284	(1 of 6) KOG1187//KOG2345 - Serine/threonine protein kinase // Serine/threonine protein kinase/TGF-beta stimulated factor	CRLK2_ARATH	Calcium/calmodulin-regulated receptor-like kinase 2 (AtCRLK2) (EC 2.7.11.1)	CRLK2 At5g15730 F14F8.110	Arabidopsis thaliana (Mouse-ear cress)		GO:0004674; GO:0005524; GO:0005886; GO:0006468; GO:0009536; GO:0016021	protein phosphorylation [GO:0006468]
Pavir.4KG130200	Pavir.4KG130200.v5.1	Chr04K	18741802	18745195	(1 of 1) K17586 - PHD and RING finger domain-containing protein 1 (PHRF1)	PHRF1_HUMAN	PHD and RING finger domain-containing protein 1	PHRF1 KIAA1542	Homo sapiens (Human)		GO:0006366; GO:0006397; GO:0016020; GO:0019904; GO:0046872; GO:0070063	mRNA processing [GO:0006397]; transcription by RNA polymerase II [GO:0006366]
Pavir.4KG129900	Pavir.4KG129900.v5.1	Chr04K	18797164	18805483	(1 of 3) PTHR12526:SF304 - STARCH SYNTHASE 4, CHLOROPLASTIC/AMYLOPLASTIC-RELATED	SSY4_ARATH	Probable starch synthase 4, chloroplastic/amyloplastic (AtSS4) (EC 2.4.1.21) (Soluble starch synthase IV) (SSIV)	SS4 At4g18240 T9A21.90	Arabidopsis thaliana (Mouse-ear cress)	FUNCTION: Probably involved in the priming of starch granule formation. May play a regulatory role in the control of starch accumulation in plastids. Is necessary and sufficient to establish the correct number of starch granules observed in chloroplasts. {ECO:0000269|PubMed:17217470, ECO:0000269|PubMed:19666739, ECO:0000269|PubMed:21645200}.	GO:0004373; GO:0005982; GO:0009011; GO:0009501; GO:0009507; GO:0019252; GO:0033201	starch biosynthetic process [GO:0019252]; starch metabolic process [GO:0005982]
Pavir.4KG130000	Pavir.4KG130000.v5.1	Chr04K	18799330	18800040		#N/A	#N/A	#N/A	#N/A	#N/A	#N/A	#N/A
Pavir.4KG129700	Pavir.4KG129700.v5.1	Chr04K	18808649	18817294	(1 of 2) PTHR19861//PTHR19861:SF3 - WD40 REPEAT PROTEIN SWD2 // TRANSDUCIN/WD40 DOMAIN-CONTAINING PROTEIN	APRF1_ARATH	Protein ANTHESIS POMOTING FACTOR 1	APRF1 At5g14530 T15N1.20	Arabidopsis thaliana (Mouse-ear cress)	FUNCTION: Component of a chromatin regulatory complex involved in regulating chromatin structure in the nucleus (By similarity). Promotes flowering under long days (LD) via the regulation of bolting (PubMed:27968983). {ECO:0000250|UniProtKB:Q9VLN1, ECO:0000269|PubMed:27968983}.	GO:0003682; GO:0005634; GO:0009908; GO:0016571; GO:0048188; GO:0048510; GO:0048586; GO:0080008; GO:0080182	flower development [GO:0009908]; histone H3-K4 trimethylation [GO:0080182]; histone methylation [GO:0016571]; regulation of long-day photoperiodism, flowering [GO:0048586]; regulation of timing of transition from vegetative to reproductive phase [GO:0048510]
Pavir.4KG129705	Pavir.4KG129705.v5.1	Chr04K	18812087	18813050	(1 of 598) PTHR19446//PTHR19446:SF355 - REVERSE TRANSCRIPTASES // SUBFAMILY NOT NAMED	#N/A	#N/A	#N/A	#N/A	#N/A	#N/A	#N/A
Pavir.4KG129710	Pavir.4KG129710.v5.1	Chr04K	18813129	18813526		#N/A	#N/A	#N/A	#N/A	#N/A	#N/A	#N/A

Pavir.4KG129600	Pavir.4KG129600.v5.1	Chr04K	18824337	18841148	(1 of 2) PTHR11390//PTHR11390:SF27 - PROKARYOTIC DNA TOPOISOMERASE // DNA TOPOISOMERASE	TOP1_RICFE	DNA topoisomerase 1 (EC 5.6.2.1) (DNA topoisomerase I) (Omega-protein) (Relaxing enzyme) (Swivelase) (Untwisting enzyme)	topA RF_0530	Rickettsia felis (strain ATCC VR-1525 / URRWXCal2) (Rickettsia azadi)	FUNCTION: Releases the supercoiling and torsional tension of DNA, which is introduced during the DNA replication and transcription, by transiently cleaving and rejoining one strand of the DNA duplex. Introduces a single-strand break via transesterification at a target site in duplex DNA. The scissile phosphodiester is attacked by the catalytic tyrosine of the enzyme, resulting in the formation of a DNA-(5'-phosphotyrosyl)-enzyme intermediate and the expulsion of a 3'-OH DNA strand. The free DNA strand then undergoes passage around the unbroken strand, thus removing DNA supercoils. Finally, in the religation step, the DNA 3'-OH attacks the covalent intermediate to expel the active-site tyrosine and restore the DNA phosphodiester backbone. {ECO:0000255|HAMAP-Rule:MF_00952}.	GO:0003677; GO:0003917; GO:0005694; GO:0006265; GO:0046872	DNA topological change [GO:0006265]
Pavir.4KG129400	Pavir.4KG129400.v5.1	Chr04K	18842081	18851288	(1 of 2) PTHR23308//PTHR23308:SF2 - NUCLEAR INHIBITOR OF PROTEIN PHOSPHATASE-1 // KANADAPTIN	NADAP_HUMAN	Kanadaptin (Human lung cancer oncogene 3 protein) (HLC-3) (Kidney anion exchanger adapter protein) (Solute carrier family 4 anion exchanger member 1 adapter protein)	SLC4A1AP HLC3	Homo sapiens (Human)		GO:0003729; GO:0005654; GO:0005737; GO:0005886; GO:0043231	
Pavir.4KG129300	Pavir.4KG129300.v5.1	Chr04K	18857720	18865733	(1 of 3) K01012 - biotin synthase (bioB)	#N/A	#N/A	#N/A	#N/A	#N/A	#N/A	#N/A
Pavir.4KG161500	Pavir.4KG161500.v5.1	Chr04K	18866133	18869669	(1 of 7) K14207 - solute carrier family 38 (sodium-coupled neutral amino acid transporter), member 2 (SLC38A2, SNAT2)	AVT6A_ARATH	Amino acid transporter AVT6A (AtAvt6A)	AVT6A At3g30390 T6J22.16	Arabidopsis thaliana (Mouse-ear cress)		GO:0003333; GO:0005773; GO:0005774; GO:0015171; GO:0016021	amino acid transmembrane transport [GO:0003333]
Pavir.4KG161505	Pavir.4KG161505.v5.1	Chr04K	18890671	18891177		#N/A	#N/A	#N/A	#N/A	#N/A	#N/A	#N/A
Pavir.4KG161600	Pavir.4KG161600.v5.1	Chr04K	18897446	18901778		40C1_ORYSJ	Ricin B-like lectin R40C1 (Osr40c1)	R40C1 Os03g0327600 LOC_Os03g21040 OSJNBb0014I10.3	Oryza sativa subsp. japonica (Rice)	FUNCTION: Lectin which binds carbohydrates in vitro. Interacts through its lectin domain with glycan structures containing specific motifs. {ECO:0000250|UniProtKB:Q945P1}.	GO:0030246	
Pavir.4KG161700	Pavir.4KG161700.v5.1	Chr04K	18902504	18903705		#N/A	#N/A	#N/A	#N/A	#N/A	#N/A	#N/A
Pavir.4KG161800	Pavir.4KG161800.v5.1	Chr04K	18905653	18907741	(1 of 477) PF00646 - F-box domain (F-box)	#N/A	#N/A	#N/A	#N/A	#N/A	#N/A	#N/A
Pavir.4KG161805	Pavir.4KG161805.v5.1	Chr04K	18908790	18909723		#N/A	#N/A	#N/A	#N/A	#N/A	#N/A	#N/A
Pavir.4KG162000	Pavir.4KG162000.v5.1	Chr04K	18922259	18924351	(1 of 61) PF03101//PF10551 - FAR1 DNA-binding domain (FAR1) // MULE transposase domain (MULE)	FRS5_ARATH	Protein FAR1-RELATED SEQUENCE 5	FRS5 At4g38180 F20D10.300	Arabidopsis thaliana (Mouse-ear cress)	FUNCTION: Putative transcription activator involved in regulating light control of development.	GO:0005634; GO:0006355; GO:0008270	regulation of transcription, DNA-templated [GO:0006355]
Pavir.4KG162100	Pavir.4KG162100.v5.1	Chr04K	18927834	18929831		#N/A	#N/A	#N/A	#N/A	#N/A	#N/A	#N/A
Pavir.4KG162400	Pavir.4KG162400.v5.1	Chr04K	18951489	18955298	(1 of 477) PF00646 - F-box domain (F-box)	MOF_ORYSJ	MEIOTIC F-BOX protein MOF	MOF Os04g0464966 OSJNBa0060P14.14 OSJNBb0048E02.5	Oryza sativa subsp. japonica (Rice)	FUNCTION: Probable component of a SCF (SKP1-CULLIN-F-box protein) E3 ubiquitin-protein ligase complex and may function through the ubiquitin-mediated protein degradation or signaling pathway. Required for male meiotic prophase I progression. Required for telomere bouquet formation, homologous chromosome pairing and for the formation of the synaptonemal complex (SC), which stabilizes initial chromosomal axial associations and promotes crossover formation. Involved in meiotic DNA double-strand break (DSB) end-processing and repair, and is important in the recruitment of DSB repair proteins to the DSB sites. {ECO:0000269|PubMed:27436711}.	GO:0005634; GO:0005694; GO:0006281; GO:0016567; GO:0051321	DNA repair [GO:0006281]; meiotic cell cycle [GO:0051321]; protein ubiquitination [GO:0016567]
Pavir.4KG162600	Pavir.4KG162600.v5.1	Chr04K	18981618	18987516	(1 of 2) PTHR22849//PTHR22849:SF47 - WDSAM1 PROTEIN // SUBFAMILY NOT NAMED	PUB42_ARATH	Putative U-box domain-containing protein 42 (EC 2.3.2.27) (Plant U-box protein 42) (RING-type E3 ubiquitin transferase PUB42)	PUB42 At1g68940 T6L1.12	Arabidopsis thaliana (Mouse-ear cress)	FUNCTION: Functions as an E3 ubiquitin ligase. {ECO:0000250}.	GO:0004842	
Pavir.4KG162800	Pavir.4KG162800.v5.1	Chr04K	18998114	18999102	(1 of 4) PTHR22952//PTHR22952:SF191 - CAMP-RESPONSE ELEMENT BINDING PROTEIN-RELATED // SUBFAMILY NOT NAMED	BZP43_ARATH	Basic leucine zipper 43 (AtbZIP43) (bZIP protein 43)	BZIP43 At5g38800 K15E6.1	Arabidopsis thaliana (Mouse-ear cress)	FUNCTION: Probable transcription factor involved in somatic embryogenesis. Acts as positive regulator of BHLH109. {ECO:0000269|PubMed:26973252}.	GO:0003677; GO:0003700; GO:0005634	
Pavir.4KG163000	Pavir.4KG163000.v5.1	Chr04K	19029204	19031483	(1 of 16) PF00643//PF06203 - B-box zinc finger (zf-B_box) // CCT motif (CCT)	HD1_ORYSJ	Zinc finger protein HD1 (Protein CONSTANS-like) (Protein HEADING DATE 1) (OsHd1) (Protein PHOTOPERIOD SENSITIVITY 1)	HD1 SE1 Os06g0275000 LOC_Os06g16370 P0038C05.23 P0676F10.34	Oryza sativa subsp. japonica (Rice)	FUNCTION: Probable transcription factor involved in the regulation of flower development. Required for the promotion of flowering under short day (SD) conditions and the suppression of flowering under long day (LD) conditions. Regulates positively the floral activator HEADING DATE 3a (HD3A) under SD and negatively under LD conditions. {ECO:0000269|PubMed:12700762}.	GO:0003677; GO:0003700; GO:0005634; GO:0008270; GO:0009908; GO:0009909; GO:0030154; GO:0045892; GO:0048571; GO:0048572; GO:0048576; GO:0048579	cell differentiation [GO:0030154]; flower development [GO:0009908]; long-day photoperiodism [GO:0048571]; negative regulation of long-day photoperiodism, flowering [GO:0048579]; negative regulation of transcription, DNA-templated [GO:0045892]; positive regulation of short-day photoperiodism, flowering [GO:0048576]; regulation of flower development [GO:0009909]; short-day photoperiodism [GO:0048572]
Pavir.4KG163100	Pavir.4KG163100.v5.1	Chr04K	19033831	19035675	(1 of 2) PTHR31388//PTHR31388:SF9 - FAMILY NOT NAMED // PEROXIDASE 11	PER11_ARATH	Peroxidase 11 (Atperox P11) (EC 1.11.1.7) (ATP23a/ATP23b)	PER11 P11 At1g68850 F14K14.4 T6L1.4	Arabidopsis thaliana (Mouse-ear cress)	FUNCTION: Removal of H(2)O(2), oxidation of toxic reductants, biosynthesis and degradation of lignin, suberization, auxin catabolism, response to environmental stresses such as wounding, pathogen attack and oxidative stress. These functions might be dependent on each isozyme/isoform in each plant tissue.	GO:0004601; GO:0005576; GO:0006979; GO:0020037; GO:0042744; GO:0046872; GO:0048511	hydrogen peroxide catabolic process [GO:0042744]; response to oxidative stress [GO:0006979]; rhythmic process [GO:0048511]

Pavir.4KG163105	Pavir.4KG163105.v5.1	Chr04K	19045078	19049911	(1 of 332) PF01535//PF13041 - PPR repeat (PPR) // PPR repeat family (PPR_2)	PPR73_ARATH	Pentatricopeptide repeat-containing protein At1g43980, mitochondrial	PCMP-E58 At1g43980 F9C16.15	Arabidopsis thaliana (Mouse-ear cress)		GO:0003723; GO:0005739; GO:0009451; GO:0043231	RNA modification [GO:0009451]
Pavir.4KG163300	Pavir.4KG163300.v5.1	Chr04K	19050319	19054086	(1 of 4) PTHR27001:SF217 - PROTEIN NSP-INTERACTING KINASE 1	SERL2_ORYSJ	LRR receptor kinase SERL2 (EC 2.7.11.1) (BRI1-associated receptor kinase 1 homolog 7) (OsBAK1-7) (Somatic embryogenesis receptor kinase-like 2) (OsSERL2)	SERL2 BAK1-7 Os06g0274500 LOC_Os06g16330 P0038C05.17 P0676F10.28	Oryza sativa subsp. japonica (Rice)	FUNCTION: LRR receptor kinase that may be involved in defense response. {ECO:0000250|UniProtKB:Q7XV05}.	GO:0004674; GO:0005524; GO:0005886; GO:0006468; GO:0006952; GO:0016021	defense response [GO:0006952]; protein phosphorylation [GO:0006468]
Pavir.4KG163305	Pavir.4KG163305.v5.1	Chr04K	19063091	19063909	(1 of 598) PTHR19446//PTHR19446:SF355 - REVERSE TRANSCRIPTASES // SUBFAMILY NOT NAMED	#N/A	#N/A	#N/A	#N/A	#N/A	#N/A	#N/A
Pavir.4KG163500	Pavir.4KG163500.v5.1	Chr04K	19068735	19070072		#N/A	#N/A	#N/A	#N/A	#N/A	#N/A	#N/A
Pavir.4KG163700	Pavir.4KG163700.v5.1	Chr04K	19075732	19086662	(1 of 46) PTHR23155:SF402 - DISEASE RESISTANCE PROTEIN RPP13-RELATED	PIK6_ORYSJ	Disease resistance protein PIK6-NP	PIK6-NP Os11g0689100 LOC_Os11g46210 OsJ_26447	Oryza sativa subsp. japonica (Rice)	FUNCTION: Probable disease resistance protein. Resistance proteins guard the plant against pathogens that contain an appropriate avirulence protein via an indirect interaction with this avirulence protein. That triggers a defense system including the hypersensitive response, which restricts the pathogen growth. At the opposite of cultivar Kusabue, the cultivar Nipponbare doesn't recognize the effector avirulence protein AVR-Pik from M.oryzae. {ECO:0000305|PubMed:18940787, ECO:0000305|PubMed:21118257}.	GO:0005524; GO:0006952; GO:0043531	defense response [GO:0006952]
Pavir.4KG163800	Pavir.4KG163800.v5.1	Chr04K	19095811	19097237		#N/A	#N/A	#N/A	#N/A	#N/A	#N/A	#N/A
Pavir.4KG163805	Pavir.4KG163805.v5.1	Chr04K	19176017	19177053		#N/A	#N/A	#N/A	#N/A	#N/A	#N/A	#N/A
Pavir.4KG163810	Pavir.4KG163810.v5.1	Chr04K	19183040	19185215		#N/A	#N/A	#N/A	#N/A	#N/A	#N/A	#N/A
Pavir.4KG163815	Pavir.4KG163815.v5.1	Chr04K	19185494	19187654	(1 of 61) PF03101//PF10551 - FAR1 DNA-binding domain (FAR1) // MULE transposase domain (MULE)	FRS5_ARATH	Protein FAR1-RELATED SEQUENCE 5	FRS5 At4g38180 F20D10.300	Arabidopsis thaliana (Mouse-ear cress)	FUNCTION: Putative transcription activator involved in regulating light control of development.	GO:0005634; GO:0006355; GO:0008270	regulation of transcription, DNA-templated [GO:0006355]
Pavir.4KG164300	Pavir.4KG164300.v5.1	Chr04K	19193933	19201979	(1 of 3) PTHR12668//PTHR12668:SF18 - TRANSMEMBRANE PROTEIN 14, 15 // SUBFAMILY NOT NAMED	FAX1_ARATH	Protein FATTY ACID EXPORT 1, chloroplastic (At-FAX1)	FAX1 At3g57280 F28O9.130	Arabidopsis thaliana (Mouse-ear cress)	FUNCTION: Mediates the export of free fatty acid from the plastids. Potentially prefers palmitic acid (C16:0) over oleic acid (C18:1) and stearic acid (C18:0). Not involved in fatty acid activation. Required for biogenesis of the outer pollen cell wall, in particular for the assembly of exine and pollen coat and for the release of ketone wax components. {ECO:0000269|PubMed:25646734}.	GO:0005829; GO:0009507; GO:0009536; GO:0009706; GO:0009941; GO:0010208; GO:0015245; GO:0015908; GO:0016020; GO:0016021; GO:0055088; GO:0071668; GO:1902001	fatty acid transmembrane transport [GO:1902001]; fatty acid transport [GO:0015908]; lipid homeostasis [GO:0055088]; plant-type cell wall assembly [GO:0071668]; pollen wall assembly [GO:0010208]
Pavir.4KG164500	Pavir.4KG164500.v5.1	Chr04K	19203106	19204383	(1 of 39) K08818 - cell division cycle 2-like [EC:2.7.11.22] (CDC2L)	CDKF2_ORYSJ	Putative cyclin-dependent kinase F-2 (CDKF;2) (EC 2.7.11.22) (EC 2.7.11.23)	CDKF-2 Os12g0424700 LOC_Os12g23700	Oryza sativa subsp. japonica (Rice)		GO:0000307; GO:0000790; GO:0004693; GO:0005524; GO:0005634; GO:0006468; GO:0008353; GO:0032968; GO:0070816	phosphorylation of RNA polymerase II C-terminal domain [GO:0070816]; positive regulation of transcription elongation from RNA polymerase II promoter [GO:0032968]; protein phosphorylation [GO:0006468]
Pavir.4KG164600	Pavir.4KG164600.v5.1	Chr04K	19229754	19236775	(1 of 4) K10601 - E3 ubiquitin-protein ligase synoviolin (SYVN1, HRD1)	HRD1_ORYSJ	ERAD-associated E3 ubiquitin-protein ligase HRD1 (EC 2.3.2.27) (RING-type E3 ubiquitin transferase HRD1)	HRD1 Os06g0301000 LOC_Os06g19680 OJ1217_C01.4 P0501G08.42	Oryza sativa subsp. japonica (Rice)	FUNCTION: Probable component of the HRD1 ubiquitin ligase complex that mediates the rapid degradation of misfolded endoplasmic reticulum (ER) proteins, a process called ER-associated degradation (ERAD). {ECO:0000250|UniProtKB:Q9LW77}.	GO:0005789; GO:0016021; GO:0016567; GO:0016740; GO:0046872	protein ubiquitination [GO:0016567]
Pavir.4KG164605	Pavir.4KG164605.v5.1	Chr04K	19232060	19232770		#N/A	#N/A	#N/A	#N/A	#N/A	#N/A	#N/A
Pavir.4KG164610	Pavir.4KG164610.v5.1	Chr04K	19232478	19233296	(1 of 598) PTHR19446//PTHR19446:SF355 - REVERSE TRANSCRIPTASES // SUBFAMILY NOT NAMED	#N/A	#N/A	#N/A	#N/A	#N/A	#N/A	#N/A
Pavir.4KG164615	Pavir.4KG164615.v5.1	Chr04K	19236229	19236634		#N/A	#N/A	#N/A	#N/A	#N/A	#N/A	#N/A
Pavir.4KG164700	Pavir.4KG164700.v5.1	Chr04K	19239474	19240835	(1 of 42) PF03181 - BURP domain (BURP)	BURPA_ORYSJ	BURP domain-containing protein 10 (OsBURP10)	BURP10 Os06g0281800 LOC_Os06g17000 OsJ_20979 OSJNBa0063H02.22	Oryza sativa subsp. japonica (Rice)			
Pavir.4KG164705	Pavir.4KG164705.v5.1	Chr04K	19261607	19262722	(1 of 5) PTHR10178//PTHR10178:SF309 - GAG/POL/ENV POLYPROTEIN // SUBFAMILY NOT NAMED	#N/A	#N/A	#N/A	#N/A	#N/A	#N/A	#N/A
Pavir.4KG168100	Pavir.4KG168100.v5.1	Chr04K	19555069	19556551	(1 of 33) PF00335 - Tetraspanin family (Tetraspannin)	TET8_ARATH	Tetraspanin-8	TET8 At2g23810 F27L4.1	Arabidopsis thaliana (Mouse-ear cress)	FUNCTION: May be involved in the regulation of cell differentiation. {ECO:0000250}.	GO:0005773; GO:0005794; GO:0005886; GO:0009506; GO:0016021; GO:0050829	defense response to Gram-negative bacterium [GO:0050829]
Pavir.4KG168105	Pavir.4KG168105.v5.1	Chr04K	19569437	19572689	(1 of 12) PTHR13683//PTHR13683:SF267 - ASPARTYL PROTEASES // ASPARTYL PROTEASE FAMILY PROTEIN-RELATED	NEP2_NEPGR	Aspartic proteinase nepenthesin-2 (EC 3.4.23.12) (Nepenthesin-II)	nep2	Nepenthes gracilis (Slender pitcher plant)	FUNCTION: Extracellular proteinase found in the pitcher fluid of carnivorous plants. Digest prey for nitrogen uptake.	GO:0004190; GO:0005576	
Pavir.4KG168110	Pavir.4KG168110.v5.1	Chr04K	19572777	19575688		#N/A	#N/A	#N/A	#N/A	#N/A	#N/A	#N/A
Pavir.4KG168115	Pavir.4KG168115.v5.1	Chr04K	19575852	19577227		#N/A	#N/A	#N/A	#N/A	#N/A	#N/A	#N/A
Pavir.4KG168120	Pavir.4KG168120.v5.1	Chr04K	19590593	19592266	(1 of 116) PF13839//PF14416 - GDSL/SGNH-like Acyl-Esterase family found in Pmr5 and Cas1p (PC-Esterase) // PMR5 N terminal Domain (PMR5N)	TBL19_ARATH	Protein trichome birefringence-like 19	TBL19 At5g15900 F1N13.40	Arabidopsis thaliana (Mouse-ear cress)	FUNCTION: May act as a bridging protein that binds pectin and other cell wall polysaccharides. Probably involved in maintaining esterification of pectins (By similarity). May be involved in the specific O-acetylation of cell wall polymers (By similarity). {ECO:0000250|UniProtKB:Q9FG35, ECO:0000250|UniProtKB:Q9LY46}.	GO:0005794; GO:0016021; GO:0016413	

<table>
<tr><th></th><th></th><th></th><th></th><th></th><th></th><th></th><th></th><th></th><th></th><th></th><th></th><th></th></tr>
<tr><td>Pavir.4KG168600</td><td>Pavir.4KG168600.v5.1</td><td>Chr04K</td><td>19622362</td><td>19625538</td><td>(1 of 2) PF00400//PF00646 - WD domain, G-beta repeat (WD40) // F-box domain (F-box)</td><td>FBW3_ARATH</td><td>F-box/WD-40 repeat-containing protein At5g21040</td><td>At5g21040 T10F18.70</td><td>Arabidopsis thaliana (Mouse-ear cress)</td><td></td><td>GO:0005634; GO:0005737; GO:0016036</td><td>cellular response to phosphate starvation [GO:0016036]</td></tr>
<tr><td>Pavir.4KG168700</td><td>Pavir.4KG168700.v5.1</td><td>Chr04K</td><td>19671023</td><td>19676280</td><td>(1 of 116) PF13839//PF14416 - GDSL/SGNH-like Acyl-Esterase family found in Pmr5 and Cas1p (PC-Esterase) // PMR5 N terminal Domain (PMR5N)</td><td>TBL21_ARATH</td><td>Protein trichome birefringence-like 21</td><td>TBL21 At5g15890 F1N13.30</td><td>Arabidopsis thaliana (Mouse-ear cress)</td><td>FUNCTION: May act as a bridging protein that binds pectin and other cell wall polysaccharides. Probably involved in maintaining esterification of pectins (By similarity). May be involved in the specific O-acetylation of cell wall polymers (By similarity). {ECO:0000250|UniProtKB:Q9FG35, ECO:0000250|UniProtKB:Q9LY46}.</td><td>GO:0005794; GO:0016021; GO:0016413</td><td></td></tr>
<tr><td>Pavir.4KG168900</td><td>Pavir.4KG168900.v5.1</td><td>Chr04K</td><td>19676964</td><td>19678695</td><td>(1 of 116) PF13839//PF14416 - GDSL/SGNH-like Acyl-Esterase family found in Pmr5 and Cas1p (PC-Esterase) // PMR5 N terminal Domain (PMR5N)</td><td>TBL19_ARATH</td><td>Protein trichome birefringence-like 19</td><td>TBL19 At5g15900 F1N13.40</td><td>Arabidopsis thaliana (Mouse-ear cress)</td><td>FUNCTION: May act as a bridging protein that binds pectin and other cell wall polysaccharides. Probably involved in maintaining esterification of pectins (By similarity). May be involved in the specific O-acetylation of cell wall polymers (By similarity). {ECO:0000250|UniProtKB:Q9FG35, ECO:0000250|UniProtKB:Q9LY46}.</td><td>GO:0005794; GO:0016021; GO:0016413</td><td></td></tr>
<tr><td>Pavir.4KG168905</td><td>Pavir.4KG168905.v5.1</td><td>Chr04K</td><td>19680229</td><td>19682835</td><td>(1 of 24) PF04937//PF05699 - Protein of unknown function (DUF 659) (DUF659) // hAT family C-terminal dimerisation region (Dimer_Tnp_hAT)</td><td>#N/A</td><td>#N/A</td><td>#N/A</td><td>#N/A</td><td>#N/A</td><td>#N/A</td><td>#N/A</td></tr>
<tr><td>Pavir.4KG169000</td><td>Pavir.4KG169000.v5.1</td><td>Chr04K</td><td>19686371</td><td>19687270</td><td>(1 of 8) PTHR14155//PTHR14155:SF103 - RING FINGER DOMAIN-CONTAINING // SUBFAMILY NOT NAMED</td><td>ATL52_ARATH</td><td>RING-H2 finger protein ATL52 (EC 2.3.2.27) (RING-type E3 ubiquitin transferase ATL52)</td><td>ATL52 At5g17600 K10A8_80</td><td>Arabidopsis thaliana (Mouse-ear cress)</td><td></td><td>GO:0016021; GO:0016567; GO:0016740; GO:0046872</td><td>protein ubiquitination [GO:0016567]</td></tr>
<tr><td>Pavir.4KG169300</td><td>Pavir.4KG169300.v5.1</td><td>Chr04K</td><td>19702190</td><td>19708299</td><td>(1 of 2) PTHR11926//PTHR11926:SF273 - GLUCOSYL/GLUCURONOSYL TRANSFERASES // SUBFAMILY NOT NAMED</td><td>U90A1_ARATH</td><td>UDP-glycosyltransferase 90A1 (EC 2.4.1.-)</td><td>UGT90A1 At2g16890 F12A24.7</td><td>Arabidopsis thaliana (Mouse-ear cress)</td><td></td><td>GO:0008194</td><td></td></tr>
<tr><td>Pavir.4KG169400</td><td>Pavir.4KG169400.v5.1</td><td>Chr04K</td><td>19724099</td><td>19724911</td><td></td><td>#N/A</td><td>#N/A</td><td>#N/A</td><td>#N/A</td><td>#N/A</td><td>#N/A</td><td>#N/A</td></tr>
<tr><td>Pavir.4KG147700</td><td>Pavir.4KG147700.v5.1</td><td>Chr04K</td><td>19727179</td><td>19731330</td><td></td><td>#N/A</td><td>#N/A</td><td>#N/A</td><td>#N/A</td><td>#N/A</td><td>#N/A</td><td>#N/A</td></tr>
<tr><td>Pavir.4KG147705</td><td>Pavir.4KG147705.v5.1</td><td>Chr04K</td><td>19733787</td><td>19736061</td><td>(1 of 181) PF12776 - Myb/SANT-like DNA-binding domain (Myb_DNA-bind_3)</td><td>LIMYB_ARATH</td><td>L10-interacting MYB domain-containing protein</td><td>LIMYB At5g05800 MJJ3.22</td><td>Arabidopsis thaliana (Mouse-ear cress)</td><td>FUNCTION: Transcriptional repressor that associates with ribosomal protein promoters. {ECO:0000269|PubMed:25707794}.</td><td>GO:0005634</td><td></td></tr>
<tr><td>Pavir.4KG169700</td><td>Pavir.4KG169700.v5.1</td><td>Chr04K</td><td>19744178</td><td>19752918</td><td>(1 of 4) PTHR11699:SF180 - ALDEHYDE DEHYDROGENASE FAMILY 2 MEMBER B7, MITOCHONDRIAL</td><td>#N/A</td><td>#N/A</td><td>#N/A</td><td>#N/A</td><td>#N/A</td><td>#N/A</td><td>#N/A</td></tr>
<tr><td>Pavir.4KG146100</td><td>Pavir.4KG146100.v5.1</td><td>Chr04K</td><td>19747199</td><td>19747915</td><td></td><td>#N/A</td><td>#N/A</td><td>#N/A</td><td>#N/A</td><td>#N/A</td><td>#N/A</td><td>#N/A</td></tr>
<tr><td>Pavir.4KG146105</td><td>Pavir.4KG146105.v5.1</td><td>Chr04K</td><td>19757014</td><td>19758206</td><td></td><td>#N/A</td><td>#N/A</td><td>#N/A</td><td>#N/A</td><td>#N/A</td><td>#N/A</td><td>#N/A</td></tr>
<tr><td>Pavir.4KG146110</td><td>Pavir.4KG146110.v5.1</td><td>Chr04K</td><td>19758222</td><td>19758680</td><td></td><td>#N/A</td><td>#N/A</td><td>#N/A</td><td>#N/A</td><td>#N/A</td><td>#N/A</td><td>#N/A</td></tr>
<tr><td>Pavir.4KG146115</td><td>Pavir.4KG146115.v5.1</td><td>Chr04K</td><td>19786723</td><td>19791931</td><td></td><td>#N/A</td><td>#N/A</td><td>#N/A</td><td>#N/A</td><td>#N/A</td><td>#N/A</td><td>#N/A</td></tr>
<tr><td>Pavir.4KG146120</td><td>Pavir.4KG146120.v5.1</td><td>Chr04K</td><td>19787875</td><td>19790517</td><td>(1 of 9) PTHR19446//PTHR19446:SF373 - REVERSE TRANSCRIPTASES // SUBFAMILY NOT NAMED</td><td>#N/A</td><td>#N/A</td><td>#N/A</td><td>#N/A</td><td>#N/A</td><td>#N/A</td><td>#N/A</td></tr>
<tr><td>Pavir.4KG170000</td><td>Pavir.4KG170000.v5.1</td><td>Chr04K</td><td>19792199</td><td>19797903</td><td>(1 of 5) PTHR30540//PTHR30540:SF37 - OSMOTIC STRESS POTASSIUM TRANSPORTER // SUBFAMILY NOT NAMED</td><td>HAK25_ORYSJ</td><td>Potassium transporter 25 (OsHAK25)</td><td>HAK25 Os02g0730300 LOC_Os02g49760 OSJNBa0072H09.37 P0617A09.16</td><td>Oryza sativa subsp. japonica (Rice)</td><td>FUNCTION: High-affinity potassium transporter. {ECO:0000250}.</td><td>GO:0005886; GO:0006813; GO:0015079; GO:0016020; GO:0016021</td><td>potassium ion transport [GO:0006813]</td></tr>
<tr><td>Pavir.4KG361300</td><td>Pavir.4KG361300.v5.1</td><td>Chr04K</td><td>19835761</td><td>19839931</td><td>(1 of 1) PTHR13833//PTHR13833:SF42//PTHR13833:SF52 - FAMILY NOT NAMED // NHL DOMAIN-CONTAINING PROTEIN // SUBFAMILY NOT NAMED</td><td>#N/A</td><td>#N/A</td><td>#N/A</td><td>#N/A</td><td>#N/A</td><td>#N/A</td><td>#N/A</td></tr>
<tr><td>Pavir.4KG361200</td><td>Pavir.4KG361200.v5.1</td><td>Chr04K</td><td>19840693</td><td>19843874</td><td>(1 of 116) PF00892 - EamA-like transporter family (EamA)</td><td>Y788_ARCFU</td><td>Uncharacterized transporter AF_0788</td><td>AF_0788</td><td>Archaeoglobus fulgidus (strain ATCC 49558 / VC-16 / DSM 4304 / JCM 9628 / NBRC 100126)</td><td></td><td>GO:0005886; GO:0016021</td><td></td></tr>
<tr><td>Pavir.4KG361205</td><td>Pavir.4KG361205.v5.1</td><td>Chr04K</td><td>19874749</td><td>19876841</td><td></td><td>#N/A</td><td>#N/A</td><td>#N/A</td><td>#N/A</td><td>#N/A</td><td>#N/A</td><td>#N/A</td></tr>
<tr><td>Pavir.4KG361210</td><td>Pavir.4KG361210.v5.1</td><td>Chr04K</td><td>19916465</td><td>19916884</td><td></td><td>#N/A</td><td>#N/A</td><td>#N/A</td><td>#N/A</td><td>#N/A</td><td>#N/A</td><td>#N/A</td></tr>
<tr><td>Pavir.4KG361215</td><td>Pavir.4KG361215.v5.1</td><td>Chr04K</td><td>19916792</td><td>19917805</td><td></td><td>#N/A</td><td>#N/A</td><td>#N/A</td><td>#N/A</td><td>#N/A</td><td>#N/A</td><td>#N/A</td></tr>
<tr><td>Pavir.4KG361220</td><td>Pavir.4KG361220.v5.1</td><td>Chr04K</td><td>19918537</td><td>19919820</td><td></td><td>#N/A</td><td>#N/A</td><td>#N/A</td><td>#N/A</td><td>#N/A</td><td>#N/A</td><td>#N/A</td></tr>
<tr><td>Pavir.4KG361225</td><td>Pavir.4KG361225.v5.1</td><td>Chr04K</td><td>19921357</td><td>19921673</td><td></td><td>#N/A</td><td>#N/A</td><td>#N/A</td><td>#N/A</td><td>#N/A</td><td>#N/A</td><td>#N/A</td></tr>
<tr><td>Pavir.4KG170300</td><td>Pavir.4KG170300.v5.1</td><td>Chr04K</td><td>19936745</td><td>19940927</td><td>(1 of 5) KOG4711 - Predicted membrane protein</td><td>ALMT9_ARATH</td><td>Aluminum-activated malate transporter 9 (AtALMT9)</td><td>ALMT9 At3g18440 MYF24.16</td><td>Arabidopsis thaliana (Mouse-ear cress)</td><td>FUNCTION: Vacuolar malate channel. Has a higher selectivity for malate than for fumarate. Exhibits also a weak chloride conductance. {ECO:0000269|PubMed:18005230}.</td><td>GO:0005253; GO:0009705; GO:0015743; GO:0016021</td><td>malate transport [GO:0015743]</td></tr>
<tr><td>Pavir.4KG170400</td><td>Pavir.4KG170400.v5.1</td><td>Chr04K</td><td>19941326</td><td>19945425</td><td>(1 of 4) PTHR13683//PTHR13683:SF334 - ASPARTYL PROTEASES // ASPARTYL PROTEASE</td><td>APCB1_ARATH</td><td>Aspartyl protease APCB1 (EC 3.4.23.-) (Aspartyl protease cleaving BAG 1)</td><td>APCB1 At1g49050 F27J15.15</td><td>Arabidopsis thaliana (Mouse-ear cress)</td><td>FUNCTION: Involved in proteolytic processing of BAG6 and plant basal immunity. {ECO:0000269|PubMed:26739014}.</td><td>GO:0004190; GO:0016021; GO:0050832</td><td>defense response to fungus [GO:0050832]</td></tr>
<tr><td>Pavir.4KG170405</td><td>Pavir.4KG170405.v5.1</td><td>Chr04K</td><td>19971755</td><td>19982426</td><td>(1 of 2) K17906 - autophagy-related protein 2 (ATG2)</td><td>ATG2_ARATH</td><td>Autophagy-related protein 2 (AtAPG2) (Protein PEROXISOME UNUSUAL POSITIONING 1)</td><td>ATG2 APG2 PEUP1 At3g19190 MVI11.10/MVI11.11</td><td>Arabidopsis thaliana (Mouse-ear cress)</td><td>FUNCTION: Required for autophagy that plays an essential role in plant nutrient recycling (PubMed:21645148, PubMed:19773385). Involved in the early steps of autophagosome biogenesis. Involved in the negative regulation of plant defense responses to biotrophic pathogens (PubMed:21645148). Involved in a negative feedback loop that modulates NPR1-dependent salicylic acid (SA) signaling and limits senescence and immunity-related programmed cell death (PCD) in plants (PubMed:19773385). Involved in the degradation of damaged peroxisomes (PubMed:24368788). Tethers the edge of the isolation membrane (IM) to the endoplasmic reticulum (ER) and mediates direct lipid transfer from ER to IM for IM expansion (By similarity). {ECO:0000250|UniProtKB:Q96BY7, ECO:0000269|PubMed:19773385, ECO:0000269|PubMed:21645148, ECO:0000269|PubMed:24368788}.</td><td>GO:0000045; GO:0000407; GO:0000422; GO:0005789; GO:0009816; GO:0010150; GO:0015031; GO:0019898; GO:0030242; GO:0032266; GO:0034045; GO:0034727; GO:0044805; GO:0050832; GO:0061709</td><td>autophagosome assembly [GO:0000045]; autophagy of mitochondrion [GO:0000422]; autophagy of peroxisome [GO:0030242]; defense response to bacterium, incompatible interaction [GO:0009816]; defense response to fungus [GO:0050832]; late nucleophagy [GO:0044805]; leaf senescence [GO:0010150]; piecemeal microautophagy of the nucleus [GO:0034727]; protein transport [GO:0015031]; reticulophagy [GO:0061709]</td></tr>
</table>

Pavir.4KG170410	Pavir.4KG170410.v5.1	Chr04K	19983294	19983969		#N/A	#N/A	#N/A	#N/A	#N/A	#N/A	#N/A
Pavir.4KG170415	Pavir.4KG170415.v5.1	Chr04K	19993052	19994894		#N/A	#N/A	#N/A	#N/A	#N/A	#N/A	#N/A
Pavir.4KG170420	Pavir.4KG170420.v5.1	Chr04K	19995895	20000858	(1 of 17) 1.14.13.8 - Flavin-containing monooxygenase / Ziegler's enzyme	#N/A	#N/A	#N/A	#N/A	#N/A	#N/A	#N/A
Pavir.4KG139000	Pavir.4KG139000.v5.1	Chr04K	20092657	20095087	(1 of 1) PTHR10173//PTHR10173:SF38 - METHIONINE SULFOXIDE REDUCTASE // PEPTIDE METHIONINE SULFOXIDE REDUCTASE B1, CHLOROPLASTIC	MSRB1_ORYSJ	Peptide methionine sulfoxide reductase B1, chloroplastic (OsMSRB1) (EC 1.8.4.12) (Peptide-methionine (R)-S-oxide reductase)	MSRB1 Os06g0472000 LOC_Os06g27760 OsJ_21331 P0613F06.49-1	Oryza sativa subsp. japonica (Rice)	FUNCTION: Catalyzes the reduction of methionine sulfoxide (MetSO) to methionine in proteins. Involved in abiotic stress response. Plays a protective role against oxidative stress by restoring activity to proteins that have been inactivated by methionine oxidation. MSRB family specifically reduces the MetSO R-enantiomer. {ECO:0000269|PubMed:19415325}.	GO:0005737; GO:0006979; GO:0009507; GO:0030091; GO:0033743; GO:0046872	protein repair [GO:0030091]; response to oxidative stress [GO:0006979]
Pavir.4KG138900	Pavir.4KG138900.v5.1	Chr04K	20098598	20103311	(1 of 4) PTHR31079//PTHR31079:SF9 - FAMILY NOT NAMED // NAC DOMAIN-CONTAINING PROTEIN 8	NAC8_ARATH	SUPPRESSOR OF GAMMA RESPONSE 1 (NAC domain-containing protein 8) (ANAC008) (Protein SOG1) (SUPPRESSOR OF GAMMA RADIATION 1)	SOG1 NAC008 At1g25580 F14G11.2 F2J7.1	Arabidopsis thaliana (Mouse-ear cress)	FUNCTION: Transcription factor regulating the transcriptional activation response to gamma irradiation (PubMed:19549833). Required for stem-cell death induced by UVB or by gamma irradiation (PubMed:20634150). Not required for ATM activation, but participates in pathways governed by both ATM and ATR sensor kinases (PubMed:19549833). Involved in DNA damage response (DDR) system that regulates cell cycle arrest (PubMed:24399300). Functional homolog of animal p53 (PubMed:24736489). Regulates SMR5 and SMR7 transcription (PubMed:24399300). Regulates DNA repair and cytokinin signaling separately and plays a key role in controlling lateral root formation under genotoxic stress. {ECO:0000269|PubMed:19549833, ECO:0000269|PubMed:20634150, ECO:0000269|PubMed:24399300, ECO:0000305|PubMed:24736489}.	GO:0000077; GO:0000976; GO:0003700; GO:0005634; GO:0006355; GO:0010332; GO:0040020	DNA damage checkpoint [GO:0000077]; regulation of meiotic nuclear division [GO:0040020]; regulation of transcription, DNA-templated [GO:0006355]; response to gamma radiation [GO:0010332]
Pavir.4KG138800	Pavir.4KG138800.v5.1	Chr04K	20127382	20128172		#N/A	#N/A	#N/A	#N/A	#N/A	#N/A	#N/A
Pavir.4KG138500	Pavir.4KG138500.v5.1	Chr04K	20184011	20189237	(1 of 4) PTHR22849//PTHR22849:SF27 - WDSAM1 PROTEIN // SUBFAMILY NOT NAMED	PUB6_ARATH	U-box domain-containing protein 6 (EC 2.3.2.27) (Plant U-box protein 6) (RING-type E3 ubiquitin transferase PUB6)	PUB6 At1g24330 F3I6.27	Arabidopsis thaliana (Mouse-ear cress)	FUNCTION: Functions as an E3 ubiquitin ligase. {ECO:0000250}.	GO:0004842; GO:0005634; GO:0005737; GO:0009507	
Pavir.4KG138400	Pavir.4KG138400.v5.1	Chr04K	20202749	20203550		#N/A	#N/A	#N/A	#N/A	#N/A	#N/A	#N/A
Pavir.4KG138300	Pavir.4KG138300.v5.1	Chr04K	20205655	20209350	(1 of 4) PTHR10366//PTHR10366:SF441 - NAD DEPENDENT EPIMERASE/DEHYDRATASE // ISOFLAVONE REDUCTASE HOMOLOG P3-RELATED	PYRC5_PYRCO	Phenylcoumaran benzylic ether reductase Pyrc5 (EC 1.23.1.-) (Minor fruit allergen Pyr c 5) (allergen Pyr c 5)	PYRC5	Pyrus communis (Pear) (Pyrus domestica)	FUNCTION: Oxidoreductase involved in lignan biosynthesis (PubMed:11606193). Catalyzes the NADPH-dependent reduction of phenylcoumaran benzylic ethers (PubMed:11606193). Converts dehydrodiconiferyl alcohol (DDC) to isodihydrodehydrodiconiferyl alcohol (IDDDC) (PubMed:11606193). {ECO:0000269|PubMed:11606193}.	GO:0009807; GO:0032442	lignan biosynthetic process [GO:0009807]
Pavir.4KG138305	Pavir.4KG138305.v5.1	Chr04K	20284400	20288802	(1 of 598) PTHR19446//PTHR19446:SF355 - REVERSE TRANSCRIPTASES // SUBFAMILY NOT NAMED	#N/A	#N/A	#N/A	#N/A	#N/A	#N/A	#N/A
Pavir.4KG138100	Pavir.4KG138100.v5.1	Chr04K	20288799	20294861	(1 of 2) PF06792//PF09370 - Uncharacterised protein family (UPF0261) (UPF0261) // Phosphoenolpyruvate hydrolase-like (PEP_hydrolase)	TM1R_SOLLC	ToMV resistance protein Tm-1(GCR237) (Disease resistance protein Tm-1) (Protein p80(GCR237))	Tm-1	Solanum lycopersicum (Tomato) (Lycopersicon esculentum)	FUNCTION: Inhibitor of viral RNA replication which confers resistance to some tobamoviruses including tomato mosaic virus (ToMV) (e.g. isolate L), tobacco mosaic virus (TMV), tobacco mild green mosaic virus (TMGMV) and pepper mild mottle virus (PMMoV), but not to resistance-breaking isolates of ToMV (e.g. LT1, SL-1 and ToMV1-2) and tomato brown rugose fruit virus (ToBRFV) (PubMed:17699618, PubMed:3686829, PubMed:17238011, PubMed:19423673, PubMed:23415925, PubMed:29582165, PubMed:28107419). Prevents tobamoviruses RNA replication by affecting the association of tobamoviruses replication proteins (large and small subunits) with host membrane-associated proteins (e.g. TOM1, TOM2A and ARL8), thus inhibiting the replication complex formation on the membranes and avoiding viral negative-strand RNA synthesis (PubMed:17699618, PubMed:19423673, PubMed:23658455, PubMed:23415925). Inhibits triphosphatase activity of ToMV replication proteins (PubMed:25092327). {ECO:0000269|PubMed:17238011, ECO:0000269|PubMed:17699618, ECO:0000269|PubMed:19423673, ECO:0000269|PubMed:23415925, ECO:0000269|PubMed:23658455, ECO:0000269|PubMed:25092327, ECO:0000269|PubMed:28107419, ECO:0000269|PubMed:29582165, ECO:0000269|PubMed:3686829}.	GO:0003824; GO:0005524; GO:0016032; GO:0042803; GO:0044830; GO:0051607	defense response to virus [GO:0051607]; modulation by host of viral RNA genome replication [GO:0044830]; viral process [GO:0016032]
Pavir.4KG138000	Pavir.4KG138000.v5.1	Chr04K	20324846	20329337		PHS1_MAIZE	Protein POOR HOMOLOGOUS SYNAPSIS 1	PHS1 ZEAMMB73_074178	Zea mays (Maize)	FUNCTION: Required for accurate chromosome segregation in meiosis. Required for pairing to occur between homologous chromosomes. Acts in early recombination steps and ensures pairing fidelity and proper repair of meiotic DNA double-strand-breaks (PubMed:14704428, PubMed:19918061). Regulates recombination and pairing of homologous chromosomes during meiotic prophase by controlling transport of RAD50 from cytoplasm to the nucleus. May affect pairing of the gene-rich fraction of the genome rather than preventing pairing between repetitive DNA elements (PubMed:19918061). {ECO:0000269|PubMed:14704428, ECO:0000269|PubMed:19918061}.	GO:0005737; GO:0007129	homologous chromosome pairing at meiosis [GO:0007129]
Pavir.4KG137800	Pavir.4KG137800.v5.1	Chr04K	20330725	20332346	(1 of 226) K00430 - peroxidase (E1.11.1.7)	PER5_VITVI	Peroxidase 5 (EC 1.11.1.7)	GSVIVT00037159001 LOC100264145	Vitis vinifera (Grape)	FUNCTION: Removal of H(2)O(2), oxidation of toxic reductants, biosynthesis and degradation of lignin, suberization, auxin catabolism, response to environmental stresses such as wounding, pathogen attack and oxidative stress. These functions might be dependent on each isozyme/isoform in each plant tissue. {ECO:0000305}.	GO:0004601; GO:0005576; GO:0006979; GO:0020037; GO:0042744; GO:0046872	hydrogen peroxide catabolic process [GO:0042744]; response to oxidative stress [GO:0006979]
Pavir.4KG137700	Pavir.4KG137700.v5.1	Chr04K	20333410	20334338	(1 of 10) PF01277 - Oleosin (Oleosin)	OLEO_HELAN	Oleosin (Fragment)		Helianthus annuus (Common sunflower)	FUNCTION: May have a structural role to stabilize the lipid body during desiccation of the seed by preventing coalescence of the oil. Probably interacts with both lipid and phospholipid moieties of lipid bodies. May also provide recognition signals for specific lipase anchorage in lipolysis during seedling growth.	GO:0012511; GO:0016021	

Pavir.4KG137400	Pavir.4KG137400.v5.1	Chr04K	20350169	20360637	(1 of 2) PTHR15217//PTHR15217:SF0 - WILMS' TUMOR 1-ASSOCIATING PROTEIN // PRE-MRNA-SPLICING REGULATOR WTAP	FIP37_ARATH	FKBP12-interacting protein of 37 kDa (FIP-37) (Immunophilin-interacting protein AtFIP37) (Protein WTAP homolog)	FIP37 At3g54170 F24B22.130	Arabidopsis thaliana (Mouse-ear cress)	FUNCTION: Probable regulatory subunit of the N6-methyltransferase complex, a multiprotein complex that mediates N6-methyladenosine (m6A) methylation at the 5'-[AG]GAC-3' consensus sites of some mRNAs (PubMed:15047892, PubMed:28503769). Associates with MTA, MTB, VIR and HAKAI to form the m6A writer complex which is essential for adenosine methylation at specific mRNA sequences (PubMed:28503769). N6-methyladenosine (m6A) plays a role in mRNA stability, processing, translation efficiency and editing (PubMed:15047892, PubMed:28503769). Essential protein required during endosperm development and embryogenesis. Involved in endoreduplication, especially in trichomes. May play a role in splicing events (PubMed:15047892). {ECO:0000269|PubMed:15047892, ECO:0000269|PubMed:28503769}.	GO:0000381; GO:0001510; GO:0005634; GO:0006397; GO:0008380; GO:0009506; GO:0010073; GO:0016607; GO:0043621; GO:0080009	meristem maintenance [GO:0010073]; mRNA methylation [GO:0080009]; mRNA processing [GO:0006397]; regulation of alternative mRNA splicing, via spliceosome [GO:0000381]; RNA methylation [GO:0001510]; RNA splicing [GO:0008380]
Pavir.4KG137500	Pavir.4KG137500.v5.1	Chr04K	20353832	20356373		#N/A	#N/A	#N/A	#N/A	#N/A	#N/A	#N/A
Pavir.4KG137300	Pavir.4KG137300.v5.1	Chr04K	20391637	20396872	(1 of 2) K03131 - transcription initiation factor TFIID subunit 6 (TAF6)	TAF6_ARATH	Transcription initiation factor TFIID subunit 6 (Protein EMBRYO DEFECTIVE 2781) (TATA box associated factor II 59) (TBP-associated factor 6) (AtTAF6) (Transcription initiation factor TFIID subunit D5)	TAF6 EMB2781 TAFII59 At1g04950 F13M7.6	Arabidopsis thaliana (Mouse-ear cress)	FUNCTION: TAFs are components of the transcription factor IID (TFIID) complex that is essential for mediating regulation of RNA polymerase transcription. Required for proper pollen function. May stabilize the interaction of TFIID with selected promoters. Not redundant with TAF6B. {ECO:0000269|PubMed:16039640}.	GO:0000124; GO:0003713; GO:0005669; GO:0006367; GO:0009860; GO:0016251; GO:0016573; GO:0045944; GO:0046695; GO:0046982	histone acetylation [GO:0016573]; pollen tube growth [GO:0009860]; positive regulation of transcription by RNA polymerase II [GO:0045944]; transcription initiation from RNA polymerase II promoter [GO:0006367]
Pavir.4KG122400	Pavir.4KG122400.v5.1	Chr04K	20429667	20431379	(1 of 2) PTHR10108//PTHR10108:SF882 - METHYLTRANSFERASE // METHYLTRANSFERASE-RELATED	#N/A	#N/A	#N/A	#N/A	#N/A	#N/A	#N/A
Pavir.4KG122405	Pavir.4KG122405.v5.1	Chr04K	20435434	20435789		#N/A	#N/A	#N/A	#N/A	#N/A	#N/A	#N/A
Pavir.4KG122700	Pavir.4KG122700.v5.1	Chr04K	20452742	20456773		#N/A	#N/A	#N/A	#N/A	#N/A	#N/A	#N/A
Pavir.4KG122800	Pavir.4KG122800.v5.1	Chr04K	20471813	20475075		#N/A	#N/A	#N/A	#N/A	#N/A	#N/A	#N/A
Pavir.4KG123000	Pavir.4KG123000.v5.1	Chr04K	20488370	20493080	(1 of 26) PF03080//PF14365 - Domain of unknown function (DUF239) (DUF239) // Domain of unknown function (DUF4409) (DUF4409)	#N/A	#N/A	#N/A	#N/A	#N/A	#N/A	#N/A
Pavir.4KG123005	Pavir.4KG123005.v5.1	Chr04K	20523398	20523754		#N/A	#N/A	#N/A	#N/A	#N/A	#N/A	#N/A
Pavir.4KG123200	Pavir.4KG123200.v5.1	Chr04K	20543887	20545222	(1 of 7) PF00226//PF13370 - DnaJ domain (DnaJ) // 4Fe-4S single cluster domain of Ferredoxin I (Fer4_13)	#N/A	#N/A	#N/A	#N/A	#N/A	#N/A	#N/A
Pavir.4KG123300	Pavir.4KG123300.v5.1	Chr04K	20545458	20557553	(1 of 2) 2.7.9.4 - Alpha-glucan, water dikinase / Starch-related R1 protein	GWD1_SOLTU	Alpha-glucan water dikinase, chloroplastic (EC 2.7.13.3) (EC 2.7.9.4) (Starch-related R1 protein)	R1	Solanum tuberosum (Potato)	FUNCTION: Mediates the incorporation of phosphate into starch-like alpha-glucan, mostly at the C-6 position of glucose units. Acts as an overall regulator of starch mobilization. Required for starch degradation, suggesting that the phosphate content of starch regulates its degradability. More active on alpha-1,6 branched amylopectin. {ECO:0000269|PubMed:11841813, ECO:0000269|PubMed:14525539, ECO:0000269|PubMed:9592398}.	GO:0004673; GO:0005524; GO:0005739; GO:0005983; GO:0009570; GO:0009610; GO:0009631; GO:0009941; GO:0046872; GO:0102216; GO:0102218	cold acclimation [GO:0009631]; response to symbiotic fungus [GO:0009610]; starch catabolic process [GO:0005983]
Pavir.4KG123305	Pavir.4KG123305.v5.1	Chr04K	20555566	20557579		#N/A	#N/A	#N/A	#N/A	#N/A	#N/A	#N/A
Pavir.4KG123400	Pavir.4KG123400.v5.1	Chr04K	20561557	20575658	(1 of 4) PTHR23336//PTHR23336:SF19 - ZINC FINGER CW-TYPE COILED-COIL DOMAIN PROTEIN 3. // SUBFAMILY NOT NAMED	MORC6_ARATH	Protein MICRORCHIDIA 6 (AtMORC6) (EC 3.6.-.-) (Protein CRT1-homolog 6) (CRT1-h6) (Protein DEFECTIVE IN MERISTEM SILENCING 11)	MORC6 CRH6 DMS11 At1g19100 F14D16.25	Arabidopsis thaliana (Mouse-ear cress)	FUNCTION: Involved in RNA-directed DNA methylation (RdDM) as a component of the RdDM machinery and required for gene silencing (PubMed:22560611, PubMed:23675613, PubMed:27171427). Together with SUVH2 and SUVH9, regulates the silencing of some transposable elements (TEs) (PubMed:27171427). Exhibits ATPase activity (PubMed:22560611). May also be involved in the regulation of chromatin architecture/condensation to maintain gene silencing (PubMed:22555433, PubMed:27171427). Binds DNA/RNA in a non-specific manner and exhibits endonuclease activity. Probably involved in DNA repair (By similarity). Positive regulator of defense against the oomycete Hyaloperonospora arabidopsidis (Hpa) (PubMed:27171361). {ECO:0000250|UniProtKB:Q84WV6, ECO:0000269|PubMed:22555433, ECO:0000269|PubMed:22560611, ECO:0000269|PubMed:23675613, ECO:0000269|PubMed:27171361, ECO:0000269|PubMed:27171427}.	GO:0003677; GO:0003723; GO:0004519; GO:0005524; GO:0005634; GO:0006281; GO:0006282; GO:0006325; GO:0006952; GO:0009506; GO:0009941; GO:0016887; GO:0031935; GO:0042803; GO:0080188; GO:1902290	chromatin organization [GO:0006325]; defense response [GO:0006952]; DNA repair [GO:0006281]; gene silencing by RNA-directed DNA methylation [GO:0080188]; positive regulation of defense response to oomycetes [GO:1902290]; regulation of chromatin silencing [GO:0031935]; regulation of DNA repair [GO:0006282]
Pavir.4KG108500	Pavir.4KG108500.v5.1	Chr04K	20589854	20602156	(1 of 2) K12479 - vacuolar protein sorting-associated protein 45 (VPS45)	VPS45_ARATH	Vacuolar protein sorting-associated protein 45 homolog (AtVPS45)	VPS45 At1g77140 T14N5_2	Arabidopsis thaliana (Mouse-ear cress)	FUNCTION: Involved in the protein transport to the vacuole, probably at the level of vesicle fusion at the trans-Golgi network (TGN) and not in transport from the TGN to the prevacuolar compartment. Binds syntaxins.	GO:0000139; GO:0005739; GO:0005802; GO:0006886; GO:0006904; GO:0009705; GO:0016192	intracellular protein transport [GO:0006886]; vesicle docking involved in exocytosis [GO:0006904]; vesicle-mediated transport [GO:0016192]
Pavir.4KG106500	Pavir.4KG106500.v5.1	Chr04K	20644996	20645520		#N/A	#N/A	#N/A	#N/A	#N/A	#N/A	#N/A
Pavir.4KG106400	Pavir.4KG106400.v5.1	Chr04K	20648413	20648923		#N/A	#N/A	#N/A	#N/A	#N/A	#N/A	#N/A
Pavir.4KG106405	Pavir.4KG106405.v5.1	Chr04K	20715646	20719334		#N/A	#N/A	#N/A	#N/A	#N/A	#N/A	#N/A
Pavir.4KG106200	Pavir.4KG106200.v5.1	Chr04K	20767229	20768867		#N/A	#N/A	#N/A	#N/A	#N/A	#N/A	#N/A
Pavir.4KG106100	Pavir.4KG106100.v5.1	Chr04K	20846264	20847351	(1 of 12) 1.1.1.206//1.1.1.236 - Tropinone reductase I / Tropinone reductase // Tropinone reductase II / Tropinone reductase	TRNHF_ARATH	Tropinone reductase homolog At5g06060 (EC 1.1.1.-)	At5g06060 K16F4.2	Arabidopsis thaliana (Mouse-ear cress)		GO:0005829; GO:0005886; GO:0016491	
Pavir.4KG106105	Pavir.4KG106105.v5.1	Chr04K	20866292	20866573		#N/A	#N/A	#N/A	#N/A	#N/A	#N/A	#N/A
Pavir.4KG105800	Pavir.4KG105800.v5.1	Chr04K	20950131	20952572	(1 of 2) PTHR20961//PTHR20961:SF26 - GLYCOSYLTRANSFERASE // SUBFAMILY NOT NAMED	XAT3_ORYSJ	Alpha-1,3-arabinosyltransferase XAT3 (EC 2.4.2.-) (Xylan arabinosyltransferase 3) (OsXAT3)	XAT3 Os03g0567600 LOC_Os03g37010 OsJ_11476 OSJNBa0026A15.4	Oryza sativa subsp. japonica (Rice)	FUNCTION: Glycosyltransferase involved in the arabinosylation of xylan, the major hemicellulose (non-cellulosic component) of primary and secondary walls of angiosperms (PubMed:22215597). Possesses alpha-1,3-arabinosyltransferase activity, transferring an arabinofuranose residue to the xylan backbone (PubMed:22215597). {ECO:0000269|PubMed:22215597}.	GO:0000139; GO:0009664; GO:0016021; GO:0016757; GO:0052636	plant-type cell wall organization [GO:0009664]

Pavir.4KG105700	Pavir.4KG105700.v5.1	Chr04K	20969479	20973032	(1 of 2) PTHR20961//PTHR20961:SF5 - GLYCOSYLTRANSFERASE // GENOMIC DNA, CHROMOSOME 3, P1 CLONE: MRC8	XAT3_ORYSJ	Alpha-1,3-arabinosyltransferase XAT3 (EC 2.4.2.-) (Xylan arabinosyltransferase 3) (OsXAT3)	XAT3 Os03g0567600 LOC_Os03g37010 OsJ_11476 OSJNBa0026A15.4	Oryza sativa subsp. japonica (Rice)	FUNCTION: Glycosyltransferase involved in the arabinosylation of xylan, the major hemicellulose (non-cellulosic component) of primary and secondary walls of angiosperms (PubMed:22215597). Possesses alpha-1,3-arabinosyltransferase activity, transferring an arabinofuranose residue to the xylan backbone (PubMed:22215597). {ECO:0000269|PubMed:22215597}.	GO:0000139; GO:0009664; GO:0016021; GO:0016757; GO:0052636	plant-type cell wall organization [GO:0009664]
Pavir.4KG105500	Pavir.4KG105500.v5.1	Chr04K	20977667	20981867	(1 of 2) PTHR20961//PTHR20961:SF19 - GLYCOSYLTRANSFERASE // SUBFAMILY NOT NAMED	XAX1_ORYSJ	Beta-1,2-xylosyltransferase XAX1 (EC 2.4.2.-) (Protein XYLOSYL ARABINOSYL SUBSTITUTION OF XYLAN 1)	XAX1 Os02g0329800 LOC_Os02g22380 OJ1521_G01.21 OsJ_06519	Oryza sativa subsp. japonica (Rice)	FUNCTION: Glycosyltransferase involved in the xylosylation of xylan, the major hemicellulose (non-cellulosic component) of primary and secondary walls of angiosperms (PubMed:23027943). Possesses beta-1,2-xylosyltransferase activity, transferring xylose from UDP-xylose to the xylan backbone (PubMed:23027943). {ECO:0000269|PubMed:23027943}.	GO:0000139; GO:0009664; GO:0016021; GO:0016757; GO:0035252	plant-type cell wall organization [GO:0009664]
Pavir.4KG105400	Pavir.4KG105400.v5.1	Chr04K	20984442	20986985	(1 of 3) PTHR20961//PTHR20961:SF28 - GLYCOSYLTRANSFERASE // SUBFAMILY NOT NAMED	XAT3_ORYSJ	Alpha-1,3-arabinosyltransferase XAT3 (EC 2.4.2.-) (Xylan arabinosyltransferase 3) (OsXAT3)	XAT3 Os03g0567600 LOC_Os03g37010 OsJ_11476 OSJNBa0026A15.4	Oryza sativa subsp. japonica (Rice)	FUNCTION: Glycosyltransferase involved in the arabinosylation of xylan, the major hemicellulose (non-cellulosic component) of primary and secondary walls of angiosperms (PubMed:22215597). Possesses alpha-1,3-arabinosyltransferase activity, transferring an arabinofuranose residue to the xylan backbone (PubMed:22215597). {ECO:0000269|PubMed:22215597}.	GO:0000139; GO:0009664; GO:0016021; GO:0016757; GO:0052636	plant-type cell wall organization [GO:0009664]
Pavir.4KG105405	Pavir.4KG105405.v5.1	Chr04K	20989568	20990019	(1 of 7) PF04161 - Arv1-like family (Arv1)	ARV1_ARATH	Protein ARV 1 (AtArv1p)	ARV1 At1g01020 T25K16.2	Arabidopsis thaliana (Mouse-ear cress)	FUNCTION: Mediator of sterol homeostasis involved in sterol uptake, trafficking and distribution into membranes. Regulates also the sphingolipid metabolism. {ECO:0000269|PubMed:16725371}.	GO:0005783; GO:0005789; GO:0005794; GO:0006665; GO:0016021; GO:0016125; GO:0032366; GO:0032541; GO:0097036	intracellular sterol transport [GO:0032366]; regulation of plasma membrane sterol distribution [GO:0097036]; sphingolipid metabolic process [GO:0006665]; sterol metabolic process [GO:0016125]
Pavir.4KG105200	Pavir.4KG105200.v5.1	Chr04K	20993185	20994981	(1 of 2) PTHR33355//PTHR33355:SF5 - FAMILY NOT NAMED // F12F1.23 PROTEIN	#N/A	#N/A	#N/A	#N/A	#N/A	#N/A	#N/A
Pavir.4KG105000	Pavir.4KG105000.v5.1	Chr04K	21018682	21019530		#N/A	#N/A	#N/A	#N/A	#N/A	#N/A	#N/A
Pavir.4KG104800	Pavir.4KG104800.v5.1	Chr04K	21029074	21035814		#N/A	#N/A	#N/A	#N/A	#N/A	#N/A	#N/A
Pavir.4KG104805	Pavir.4KG104805.v5.1	Chr04K	21040162	21040859	(1 of 3) PTHR18934//PTHR18934:SF103 - ATP-DEPENDENT RNA HELICASE // ATP-DEPENDENT RNA HELICASE A-LIKE PROTEIN	#N/A	#N/A	#N/A	#N/A	#N/A	#N/A	#N/A
Pavir.4KG104600	Pavir.4KG104600.v5.1	Chr04K	21056635	21105206	(1 of 2) PTHR10357:SF119 - 1,4-ALPHA-GLUCAN-BRANCHING ENZYME 3, CHLOROPLASTIC/AMYLOPLASTIC	GLGB3_ARATH	1,4-alpha-glucan-branching enzyme 3, chloroplastic/amyloplastic (AtSBE III) (EC 2.4.1.18) (Branching enzyme 1) (AtBE1) (Protein EMBRYO DEFECTIVE 2729) (Starch-branching enzyme 3)	SBE3 BE1 EMB2729 At3g20440 MQC12.20	Arabidopsis thaliana (Mouse-ear cress)	FUNCTION: Catalyzes the formation of the alpha-1,6-glucosidic linkages in starch by scission of a 1,4-alpha-linked oligosaccharide from growing alpha-1,4-glucan chains and the subsequent attachment of the oligosaccharide to the alpha-1,6 position. Essential during embryogenesis. {ECO:0000269|PubMed:17028209, ECO:0000269|PubMed:20377688}.	GO:0003844; GO:0005737; GO:0005975; GO:0005978; GO:0009501; GO:0009507; GO:0009536; GO:0009570; GO:0009791; GO:0019252; GO:0043169; GO:0102752	carbohydrate metabolic process [GO:0005975]; glycogen biosynthetic process [GO:0005978]; post-embryonic development [GO:0009791]; starch biosynthetic process [GO:0019252]
Pavir.4KG104300	Pavir.4KG104300.v5.1	Chr04K	21244921	21246626		#N/A	#N/A	#N/A	#N/A	#N/A	#N/A	#N/A
Pavir.4KG104100	Pavir.4KG104100.v5.1	Chr04K	21276770	21302026	(1 of 6) 5.4.2.2//5.4.2.8 - Phosphoglucomutase (alpha-D-glucose-1,6-bisphosphate-dependent) / Phosphoglucose mutase // Phosphomannomutase / Phosphomannose mutase	ALGC_PSEAE	Phosphomannomutase/phosphoglucomutase (PMM / PGM) (EC 5.4.2.2) (EC 5.4.2.8)	algC PA5322	Pseudomonas aeruginosa (strain ATCC 15692 / DSM 22644 / CIP 104116 / JCM 14847 / LMG 12228 / 1C / PRS 101 / PAO1)	FUNCTION: Highly reversible phosphoryltransferase. The phosphomannomutase activity produces a precursor for alginate polymerization, the alginate layer causes a mucoid phenotype and provides a protective barrier against host immune defenses and antibiotics. Also involved in core lipopolysaccaride (LPS) biosynthesis due to its phosphoglucomutase activity. Essential for rhamnolipid production, an exoproduct correlated with pathogenicity (PubMed:10481091). Required for biofilm production. The reaction proceeds via 2 processive phosphoryl transferase reactions; first from enzyme-phospho-Ser-108 to the substrate (generating a bisphosphorylated substrate intermediate and a dephosphorylated enzyme), a 180 degree rotation of the intermediate (probably aided by movement of domain 4), and subsequent transfer of phosphate back to the enzyme (PubMed:11716469, PubMed:16880541, PubMed:16595672, PubMed:22242625). {ECO:0000269|PubMed:10481091, ECO:0000269|PubMed:11716469, ECO:0000269|PubMed:16595672, ECO:0000269|PubMed:16880541, ECO:0000269|PubMed:18690721, ECO:0000269|PubMed:1903398, ECO:0000269|PubMed:22242625, ECO:0000269|PubMed:23517223, ECO:0000269|PubMed:7515870, ECO:0000269|PubMed:8050998}.	GO:0000287; GO:0004614; GO:0004615; GO:0009243; GO:0009244; GO:0009298; GO:0009405; GO:0042121	alginic acid biosynthetic process [GO:0042121]; GDP-mannose biosynthetic process [GO:0009298]; lipopolysaccharide core region biosynthetic process [GO:0009244]; O antigen biosynthetic process [GO:0009243]; pathogenesis [GO:0009405]
Pavir.4KG104105	Pavir.4KG104105.v5.1	Chr04K	21330412	21333047		CCMFC_ARATH	Cytochrome c biogenesis CcmF C-terminal-like mitochondrial protein (Cytochrome c biogenesis orf452)	CCMFC CCB452 CCB6C AtMg00180	Arabidopsis thaliana (Mouse-ear cress)	FUNCTION: Forms a complex with CCMFN1, CCMFN2 and CCMH that performs the assembly of heme with c-type apocytochromes in mitochondria. {ECO:0000269|PubMed:18644794}.	GO:0005743; GO:0016021; GO:0017004	cytochrome complex assembly [GO:0017004]
Pavir.4KG104110	Pavir.4KG104110.v5.1	Chr04K	21334478	21335005	(1 of 5) K02992 - small subunit ribosomal protein S7 (RP-S7, MRPS7, rpsG)	RT07_WHEAT	Ribosomal protein S7, mitochondrial	RPS7	Triticum aestivum (Wheat)	FUNCTION: One of the primary rRNA binding proteins, it binds directly to 18S rRNA where it nucleates assembly of the head domain of the small subunit. {ECO:0000250}.	GO:0003735; GO:0005763; GO:0006412; GO:0019843	translation [GO:0006412]

Pavir.4KG104115	Pavir.4KG104115.v5.1	Chr04K	21335835	21336826	(1 of 5) K03880 - NADH-ubiquinone oxidoreductase chain 3 (ND3)	NU3M_PANGI	NADH-ubiquinone oxidoreductase chain 3 (EC 7.1.1.2) (NADH dehydrogenase subunit 3)	ND3 NAD3	Panax ginseng (Korean ginseng)	FUNCTION: Core subunit of the mitochondrial membrane respiratory chain NADH dehydrogenase (Complex I) that is believed to belong to the minimal assembly required for catalysis. Complex I functions in the transfer of electrons from NADH to the respiratory chain. The immediate electron acceptor for the enzyme is believed to be ubiquinone (By similarity). {ECO:0000250}.	GO:0008137; GO:0016021; GO:0031966; GO:0070469	
Pavir.4KG104120	Pavir.4KG104120.v5.1	Chr04K	21336827	21337328	(1 of 1) PTHR11652:SF1 - 28S RIBOSOMAL PROTEIN S12, MITOCHONDRIAL	RT12_ORYSJ	Ribosomal protein S12, mitochondrial	RPS12	Oryza sativa subsp. japonica (Rice)	FUNCTION: Protein S12 is involved in the translation initiation step.	GO:0003735; GO:0005739; GO:0005840; GO:0006412; GO:0015935	translation [GO:0006412]
Pavir.4KG104125	Pavir.4KG104125.v5.1	Chr04K	21340444	21341186		#N/A	#N/A	#N/A	#N/A	#N/A	#N/A	#N/A
Pavir.4KG116700	Pavir.4KG116700.v5.1	Chr04K	21401461	21405477		#N/A	#N/A	#N/A	#N/A	#N/A	#N/A	#N/A
Pavir.4KG116600	Pavir.4KG116600.v5.1	Chr04K	21405984	21422540		#N/A	#N/A	#N/A	#N/A	#N/A	#N/A	#N/A
Pavir.4KG116605	Pavir.4KG116605.v5.1	Chr04K	21488835	21490119	(1 of 30) PTHR23329//PTHR23329:SF9 - TUFTELIN-INTERACTING PROTEIN 11-RELATED // SUBFAMILY NOT NAMED	TFP11_XENTR	Tuftelin-interacting protein 11 (Septin and tuftelin-interacting protein 1) (STIP-1)	tfip11 stip	Xenopus tropicalis (Western clawed frog) (Silurana tropicalis)	FUNCTION: Involved in pre-mRNA splicing, specifically in spliceosome disassembly during late-stage splicing events. {ECO:0000250}.	GO:0000390; GO:0003676; GO:0005681; GO:0071008	spliceosomal complex disassembly [GO:0000390]
Pavir.4KG116610	Pavir.4KG116610.v5.1	Chr04K	21494481	21495170		#N/A	#N/A	#N/A	#N/A	#N/A	#N/A	#N/A
Pavir.4KG116615	Pavir.4KG116615.v5.1	Chr04K	21498372	21498671		#N/A	#N/A	#N/A	#N/A	#N/A	#N/A	#N/A
Pavir.4KG103400	Pavir.4KG103400.v5.1	Chr04K	21689949	21690592		#N/A	#N/A	#N/A	#N/A	#N/A	#N/A	#N/A
Pavir.4KG257000	Pavir.4KG257000.v5.1	Chr04K	22152776	22155906	(1 of 120) PF03140 - Plant protein of unknown function (DUF247)	Y3720_ARATH	UPF0481 protein At3g47200	At3g47200 F13I12.250	Arabidopsis thaliana (Mouse-ear cress)		GO:0005886; GO:0016021	
Pavir.4KG257005	Pavir.4KG257005.v5.1	Chr04K	22233212	22236176		#N/A	#N/A	#N/A	#N/A	#N/A	#N/A	#N/A
Pavir.4KG257010	Pavir.4KG257010.v5.1	Chr04K	22361627	22362100		#N/A	#N/A	#N/A	#N/A	#N/A	#N/A	#N/A
Pavir.4KG237000	Pavir.4KG237000.v5.1	Chr04K	22430673	22434320	(1 of 2) PTHR31744:SF22 - PROTEIN CUP-SHAPED COTYLEDON 1-RELATED	NAC98_ARATH	Protein CUP-SHAPED COTYLEDON 2 (NAC domain-containing protein 98) (ANAC098) (NAC domain-containing protein CUC2)	NAC098 CUC2 At5g53950 K19P17.12	Arabidopsis thaliana (Mouse-ear cress)	FUNCTION: Transcription activator of STM and KNAT6. Involved in molecular mechanisms regulating shoot apical meristem (SAM) formation during embryogenesis and organ separation. Required for the fusion of septa of gynoecia along the length of the ovaries. Activates the shoot formation in callus in a STM-dependent manner. Controls leaf margin development and required for leaf serration. Involved in axillary meristem initiation and separation of the meristem from the main stem. Regulates the phyllotaxy throughout the plant development. Seems to act as an inhibitor of cell division. {ECO:0000269|PubMed:10079219, ECO:0000269|PubMed:10750709, ECO:0000269|PubMed:12163400, ECO:0000269|PubMed:12492830, ECO:0000269|PubMed:12610213, ECO:0000269|PubMed:15202996, ECO:0000269|PubMed:15294871, ECO:0000269|PubMed:15500463, ECO:0000269|PubMed:15723790, ECO:0000269|PubMed:16798887, ECO:0000269|PubMed:17098808, ECO:0000269|PubMed:17122068, ECO:0000269|PubMed:17251269, ECO:0000269|PubMed:17287247, ECO:0000269|PubMed:9212461}.	GO:0000976; GO:0003700; GO:0005634; GO:0010072; GO:0010223; GO:0048366; GO:0090691; GO:0090709	formation of plant organ boundary [GO:0090691]; leaf development [GO:0048366]; primary shoot apical meristem specification [GO:0010072]; regulation of timing of plant organ formation [GO:0090709]; secondary shoot formation [GO:0010223]
Pavir.4KG237005	Pavir.4KG237005.v5.1	Chr04K	22496812	22497546		#N/A	#N/A	#N/A	#N/A	#N/A	#N/A	#N/A
Pavir.4KG237010	Pavir.4KG237010.v5.1	Chr04K	22715393	22717090		#N/A	#N/A	#N/A	#N/A	#N/A	#N/A	#N/A
Pavir.4KG240600	Pavir.4KG240600.v5.1	Chr04K	22717436	22721279	(1 of 1) PF00270//PF00271//PF00575 - DEAD/DEAH box helicase (DEAD) // Helicase conserved C-terminal domain (Helicase_C) // S1 RNA binding domain (S1)	DEAH5_ARATH	Probable pre-mRNA-splicing factor ATP-dependent RNA helicase DEAH5 (EC 3.6.4.13) (DEAH RNA helicase homolog PRP22)	At3g26560 MFE16.8	Arabidopsis thaliana (Mouse-ear cress)	FUNCTION: May be involved in pre-mRNA splicing. {ECO:0000305}.	GO:0000390; GO:0003723; GO:0003724; GO:0003729; GO:0005524; GO:0005739; GO:0005829; GO:0009506; GO:0071013	spliceosomal complex disassembly [GO:0000390]
Pavir.4KG240500	Pavir.4KG240500.v5.1	Chr04K	22719797	22720714	(1 of 2) PF04408//PF07717 - Helicase associated domain (HA2) (HA2) // Oligonucleotide/oligosaccharide-binding (OB)-fold (OB_NTP_bind)	#N/A	#N/A	#N/A	#N/A	#N/A	#N/A	#N/A
Pavir.4KG240400	Pavir.4KG240400.v5.1	Chr04K	22724799	22725728		#N/A	#N/A	#N/A	#N/A	#N/A	#N/A	#N/A
Pavir.4KG240300	Pavir.4KG240300.v5.1	Chr04K	22738994	22739815		#N/A	#N/A	#N/A	#N/A	#N/A	#N/A	#N/A
Pavir.4KG240200	Pavir.4KG240200.v5.1	Chr04K	22745017	22747603	(1 of 75) PF12274 - Protein of unknown function (DUF3615) (DUF3615)	#N/A	#N/A	#N/A	#N/A	#N/A	#N/A	#N/A
Pavir.4KG240100	Pavir.4KG240100.v5.1	Chr04K	22750033	22753947	(1 of 2) PTHR21392//PTHR21392:SF4 - UNCHARACTERIZED // DTW DOMAIN-CONTAINING PROTEIN	#N/A	#N/A	#N/A	#N/A	#N/A	#N/A	#N/A
Pavir.4KG240105	Pavir.4KG240105.v5.1	Chr04K	22767809	22768138		#N/A	#N/A	#N/A	#N/A	#N/A	#N/A	#N/A
Pavir.4KG240000	Pavir.4KG240000.v5.1	Chr04K	22785347	22790770	(1 of 2) PTHR10288//PTHR10288:SF134 - KH DOMAIN CONTAINING RNA BINDING PROTEIN // SUBFAMILY NOT NAMED	RCF3_ARATH	RNA-binding KH domain-containing protein RCF3 (Protein ENHANCED STRESS RESPONSE 1) (Protein HIGH OSMOTIC STRESS GENE EXPRESSION 5) (Protein REGULATOR OF CBF GENE EXPRESSION 3) (Protein SHINY 1)	RCF3 ESR1 HOS5 SHI1 At5g53060 MNB8.12	Arabidopsis thaliana (Mouse-ear cress)	FUNCTION: Acts as negative regulator of osmotic stress-induced gene expression (PubMed:10504578). Involved in the regulation of thermotolerance responses under heat stress. Functions as an upstream regulator of heat stress transcription factor (HSF) genes. Negatively regulates HSFA1A, HSFA1B AND HSFA1D, but positively controls the expression of HSFA1E, HSFA3, HSFA9, HSFB3, and DREB2C (PubMed:23087326). Forms a complex with CPL1 that modulates co-transcriptional processes such as mRNA capping and polyadenylation, and functions to repress stress-inducible gene expression (PubMed:23874224). Regulates pre-mRNA processing under salt stress (PubMed:24146632). Involved in primary miRNA processing and pri-miRNA biogenesis (PubMed:26227967, PubMed:26512101). Binds both intronless and intron-containing pri-miRNAs (PubMed:26227967). Acts as a regulator of biotic stress response gene expression and basal JA-mediated responses involved in defense. Acts as negative regulator of resistance to the fungal pathogen Fusarium oxysporum (PubMed:25985302). {ECO:0000269|PubMed:10504578, ECO:0000269|PubMed:23087326, ECO:0000269|PubMed:23874224, ECO:0000269|PubMed:24146632, ECO:0000269|PubMed:25985302, ECO:0000269|PubMed:26227967, ECO:0000269|PubMed:26512101}.	GO:0003729; GO:0005634; GO:0005737; GO:0006397; GO:0006970; GO:0008380; GO:0009867; GO:0010286; GO:0010445; GO:0010468; GO:0016607; GO:0031053; GO:0070878; GO:1900150	heat acclimation [GO:0010286]; jasmonic acid mediated signaling pathway [GO:0009867]; mRNA processing [GO:0006397]; primary miRNA processing [GO:0031053]; regulation of defense response to fungus [GO:1900150]; regulation of gene expression [GO:0010468]; response to osmotic stress [GO:0006970]; RNA splicing [GO:0008380]

Pavir.4KG240005	Pavir.4KG240005.v5.1	Chr04K	22790818	22791036	(1 of 9) PTHR21668//PTHR21668:SF5 - EIF-1A // NUCLEIC ACID-BINDING, OB-FOLD-LIKE PROTEIN-RELATED	IF1A_WHEAT	Eukaryotic translation initiation factor 1A (eIF-1A) (Eukaryotic translation initiation factor 4C) (eIF-4C)		Triticum aestivum (Wheat)	FUNCTION: Seems to be required for maximal rate of protein biosynthesis. Enhances ribosome dissociation into subunits and stabilizes the binding of the initiator Met-tRNA(I) to 40 S ribosomal subunits.	GO:0003743	
Pavir.4KG239800	Pavir.4KG239800.v5.1	Chr04K	22799037	22804037	(1 of 3) K03105 - signal recognition particle subunit SRP19 (SRP19)	SRP19_ORYSJ	Signal recognition particle 19 kDa protein (SRP19)	SRP19 Os06g0342100 LOC_Os06g23430 P0428A03.35 P0459H02.12	Oryza sativa subsp. japonica (Rice)	FUNCTION: Signal-recognition-particle assembly, binds directly to 7S RNA and mediates binding of the 54 kDa subunit of the SRP. {ECO:0000250}.	GO:0005786; GO:0006617; GO:0008312	SRP-dependent cotranslational protein targeting to membrane, signal sequence recognition [GO:0006617]
Pavir.4KG240010	Pavir.4KG240010.v5.1	Chr04K	22799675	22800835		#N/A	#N/A	#N/A	#N/A	#N/A	#N/A	#N/A
Pavir.4KG239700	Pavir.4KG239700.v5.1	Chr04K	22817188	22819567	(1 of 104) PF00612 - IQ calmodulin-binding motif (IQ)	IQD20_ARATH	Protein IQ-DOMAIN 20 (AtIQD20)	IQD20 At3g51380 F26O13.20	Arabidopsis thaliana (Mouse-ear cress)		GO:0005516; GO:0005730; GO:0005886; GO:0051592	response to calcium ion [GO:0051592]
Pavir.4KG239705	Pavir.4KG239705.v5.1	Chr04K	22823153	22823350		#N/A	#N/A	#N/A	#N/A	#N/A	#N/A	#N/A
Pavir.4KG239600	Pavir.4KG239600.v5.1	Chr04K	22827332	22827945	(1 of 4) PTHR14467//PTHR14467:SF0 - ARV1 // PROTEIN ARV1	ARV1_ARATH	Protein ARV 1 (AtArv1p)	ARV1 At1g01020 T25K16.2	Arabidopsis thaliana (Mouse-ear cress)	FUNCTION: Mediator of sterol homeostasis involved in sterol uptake, trafficking and distribution into membranes. Regulates also the sphingolipid metabolism. {ECO:0000269|PubMed:16725371}.	GO:0005783; GO:0005789; GO:0005794; GO:0006665; GO:0016021; GO:0016125; GO:0032366; GO:0032541; GO:0097036	intracellular sterol transport [GO:0032366]; regulation of plasma membrane sterol distribution [GO:0097036]; sphingolipid metabolic process [GO:0006665]; sterol metabolic process [GO:0016125]
Pavir.4KG239605	Pavir.4KG239605.v5.1	Chr04K	22828281	22829210		#N/A	#N/A	#N/A	#N/A	#N/A	#N/A	#N/A
Pavir.4KG365100	Pavir.4KG365100.v5.1	Chr04K	22878891	22884140	(1 of 4) 5.4.99.48 - Achilleol B synthase	ACBSY_ORYSJ	Achilleol B synthase (EC 5.4.99.48)	Os11g0285000 LOC_Os11g18194	Oryza sativa subsp. japonica (Rice)	FUNCTION: Specifically mediates the conversion of oxidosqualene ((3S)-2,3-epoxy-2,3-dihydrosqualene) to achilleol B. Achilleol B is probably formed by cleavage of the 8-14 and 9-10 bonds of (3S)-2,3-epoxy-2,3-dihydrosqualene as part of the cyclization reaction, after formation of the oleanane skeleton. {ECO:0000269|PubMed:21526825}.	GO:0000250; GO:0005811; GO:0016021; GO:0016104; GO:0042300	triterpenoid biosynthetic process [GO:0016104]
Pavir.4KG365105	Pavir.4KG365105.v5.1	Chr04K	22894849	22917101	(1 of 7) PTHR11764//PTHR11764:SF14 - LANOSTEROL SYNTHASE // SUBFAMILY NOT NAMED	ACBSY_ORYSJ	Achilleol B synthase (EC 5.4.99.48)	Os11g0285000 LOC_Os11g18194	Oryza sativa subsp. japonica (Rice)	FUNCTION: Specifically mediates the conversion of oxidosqualene ((3S)-2,3-epoxy-2,3-dihydrosqualene) to achilleol B. Achilleol B is probably formed by cleavage of the 8-14 and 9-10 bonds of (3S)-2,3-epoxy-2,3-dihydrosqualene as part of the cyclization reaction, after formation of the oleanane skeleton. {ECO:0000269|PubMed:21526825}.	GO:0000250; GO:0005811; GO:0016021; GO:0016104; GO:0042300	triterpenoid biosynthetic process [GO:0016104]
Pavir.4KG365110	Pavir.4KG365110.v5.1	Chr04K	22930139	22931339		#N/A	#N/A	#N/A	#N/A	#N/A	#N/A	#N/A
Pavir.4KG166800	Pavir.4KG166800.v5.1	Chr04K	22931219	22932481		#N/A	#N/A	#N/A	#N/A	#N/A	#N/A	#N/A
Pavir.4KG166805	Pavir.4KG166805.v5.1	Chr04K	22939535	22939846	(1 of 6) 5.4.99.47 - Parkeol synthase	PAKSY_ORYSJ	Parkeol synthase (EC 5.4.99.47)	Os11g0189600 LOC_Os11g08569 OsJ_33246	Oryza sativa subsp. japonica (Rice)	FUNCTION: Specifically mediates the conversion of oxidosqualene ((3S)-2,3-epoxy-2,3-dihydrosqualene) to parkeol. {ECO:0000269|PubMed:21526825}.	GO:0000250; GO:0005811; GO:0016021; GO:0016104; GO:0042300	triterpenoid biosynthetic process [GO:0016104]
Pavir.4KG166810	Pavir.4KG166810.v5.1	Chr04K	22941212	22941760		#N/A	#N/A	#N/A	#N/A	#N/A	#N/A	#N/A
Pavir.4KG166815	Pavir.4KG166815.v5.1	Chr04K	23018461	23018940		#N/A	#N/A	#N/A	#N/A	#N/A	#N/A	#N/A
Pavir.4KG166820	Pavir.4KG166820.v5.1	Chr04K	23032830	23034565		#N/A	#N/A	#N/A	#N/A	#N/A	#N/A	#N/A
Pavir.4KG210200	Pavir.4KG210200.v5.1	Chr04K	23093734	23097252	(1 of 2) PTHR34962//PTHR34962:SF1 - FAMILY NOT NAMED // PROTEIN EMBRYO DEFECTIVE 1703	#N/A	#N/A	#N/A	#N/A	#N/A	#N/A	#N/A
Pavir.4KG210100	Pavir.4KG210100.v5.1	Chr04K	23112736	23113809	(1 of 158) PF14368 - Probable lipid transfer (LTP_2)	#N/A	#N/A	#N/A	#N/A	#N/A	#N/A	#N/A
Pavir.4KG210000	Pavir.4KG210000.v5.1	Chr04K	23117317	23118490	(1 of 4) PTHR31174//PTHR31174:SF6 - SEED MATURATION FAMILY PROTEIN // SUBFAMILY NOT NAMED	LEA31_ARATH	Late embryogenesis abundant protein 31 (LEA 31) (Protein RESPONSIVE TO ABSCISIC ACID 28) (AtRAB28)	RAB28 At3g22490 F16J14.5	Arabidopsis thaliana (Mouse-ear cress)	FUNCTION: LEA proteins are late embryonic proteins abundant in higher plant seed embryos. The function of those proteins is not known (Probable). Promotes germination rate. Enhances cation toxicity (e.g. lithium ion) and osmotic stress (e.g. NaCl and sorbitol) tolerance during germination and in seedlings (PubMed:12175017). {ECO:0000269|PubMed:12175017, ECO:0000305}.	GO:0005634; GO:0005730; GO:0005829; GO:0006873; GO:0009845; GO:0010226	cellular ion homeostasis [GO:0006873]; response to lithium ion [GO:0010226]; seed germination [GO:0009845]
Pavir.4KG210005	Pavir.4KG210005.v5.1	Chr04K	23144859	23145311		#N/A	#N/A	#N/A	#N/A	#N/A	#N/A	#N/A
Pavir.4KG209000	Pavir.4KG209000.v5.1	Chr04K	23146947	23149933	(1 of 2) PTHR10666//PTHR10666:SF163 - UBIQUITIN // SUBFAMILY NOT NAMED	P4KG4_ORYSJ	Phosphatidylinositol 4-kinase gamma 4 (PI-4Kgamma4) (PI4K gamma 4) (EC 2.7.1.67) (Ubiquitin-like domain kinase gamma 4) (OsUbDKgamma4) (UbDKgamma4)	PI4KG4 Os02g0290500 LOC_Os02g18840 OJ1086_G08.5 OJ1756_H07.61 OsJ_06315	Oryza sativa subsp. japonica (Rice)	FUNCTION: The phosphorylation of phosphatidylinositol (PI) to PI4P is the first committed step in the generation of phosphatidylinositol 4,5-bisphosphate (PIP2), a precursor of the second messenger inositol 1,4,5-trisphosphate (InsP3) (By similarity). Involved in the control of flowering under long day conditions by promoting degradation of FTIP1 (PubMed:28254780). Recruits FTIP1 for degradation by the 26S proteasome in leaves, which affects RFT1 transport to the shoot apical meristem (SAM) (PubMed:28254780). {ECO:0000250|UniProtKB:Q9BTU6, ECO:0000269|PubMed:28254780}.	GO:0004430; GO:0005524; GO:0005634; GO:0005783; GO:0009908; GO:0048579	flower development [GO:0009908]; negative regulation of long-day photoperiodism, flowering [GO:0048579]
Pavir.4KG209100	Pavir.4KG209100.v5.1	Chr04K	23150728	23152017		#N/A	#N/A	#N/A	#N/A	#N/A	#N/A	#N/A
Pavir.4KG209200	Pavir.4KG209200.v5.1	Chr04K	23155893	23158411	(1 of 6) 4.3.3.7 - 4-hydroxy-tetrahydrodipicolinate synthase / Dihydrodipicolinate synthetase	DAPA1_WHEAT	4-hydroxy-tetrahydrodipicolinate synthase 1, chloroplastic (HTPA synthase 1) (EC 4.3.3.7)		Triticum aestivum (Wheat)	FUNCTION: Catalyzes the condensation of (S)-aspartate-beta-semialdehyde [(S)-ASA] and pyruvate to 4-hydroxy-tetrahydrodipicolinate (HTPA). {ECO:0000250}.	GO:0008840; GO:0009089; GO:0009507; GO:0019877	diaminopimelate biosynthetic process [GO:0019877]; lysine biosynthetic process via diaminopimelate [GO:0009089]
Pavir.4KG209300	Pavir.4KG209300.v5.1	Chr04K	23165862	23174972	(1 of 2) K03241 - translation initiation factor eIF-2B subunit gamma (EIF2B3)	EI2BG_DICDI	Translation initiation factor eIF-2B subunit gamma (eIF-2B GDP-GTP exchange factor subunit gamma)	eif2b3 DDB_G0290693	Dictyostelium discoideum (Slime mold)	FUNCTION: Catalyzes the exchange of eukaryotic initiation factor 2-bound GDP for GTP. {ECO:0000250}.	GO:0002183; GO:0003743; GO:0005085; GO:0005851; GO:0016779; GO:0032045	cytoplasmic translational initiation [GO:0002183]

Pavir.4KG209305	Pavir.4KG209305.v5.1	Chr04K	23171149	23171505		#N/A	#N/A	#N/A	#N/A	#N/A	#N/A	#N/A
Pavir.4KG209500	Pavir.4KG209500.v5.1	Chr04K	23176657	23177316	(1 of 12) PTHR11945//PTHR11945:SF232 - MADS BOX PROTEIN // SUBFAMILY NOT NAMED	AGL62_ARATH	Agamous-like MADS-box protein AGL62	AGL62 At5g60440 MUF9.24	Arabidopsis thaliana (Mouse-ear cress)	FUNCTION: Probable transcription factor. Required for suppression of cellularization and promotion of nuclear proliferation during early endosperm development. The FERTILIZATION-INDEPENDENT SEED (FIS) polycomb complex is required for suppression of ALG62 expression at the end of the syncytial phase of endosperm development. {ECO:0000269|PubMed:18334668}.	GO:0000976; GO:0000977; GO:0000981; GO:0003700; GO:0005634; GO:0008134; GO:0009960; GO:0043565; GO:0045944; GO:0046983; GO:2000012	endosperm development [GO:0009960]; positive regulation of transcription by RNA polymerase II [GO:0045944]; regulation of auxin polar transport [GO:2000012]
Pavir.4KG209600	Pavir.4KG209600.v5.1	Chr04K	23178487	23178875	(1 of 1) PTHR34453//PTHR34453:SF1 - FAMILY NOT NAMED // DEFENSIN-LIKE (DEFL) FAMILY PROTEIN-RELATED	DEF23_ARATH	Putative defensin-like protein 23	At5g19315 F7K24	Arabidopsis thaliana (Mouse-ear cress)		GO:0005576; GO:0031640; GO:0050832	defense response to fungus [GO:0050832]; killing of cells of other organism [GO:0031640]
Pavir.4KG209605	Pavir.4KG209605.v5.1	Chr04K	23200081	23201068		#N/A	#N/A	#N/A	#N/A	#N/A	#N/A	#N/A
Pavir.4KG209610	Pavir.4KG209610.v5.1	Chr04K	23205148	23205516		#N/A	#N/A	#N/A	#N/A	#N/A	#N/A	#N/A
Pavir.4KG159900	Pavir.4KG159900.v5.1	Chr04K	23209781	23210309	(1 of 3) PTHR24072//PTHR24072:SF154 - RHO FAMILY GTPASE // SUBFAMILY NOT NAMED	RAC2_ORYSJ	Rac-like GTP-binding protein 2 (OsRac2)	RAC2 Os05g051380 0 LOC_Os05g43 820 B1155G07.15 OsJ_19185 P0022D06.2	Oryza sativa subsp. japonica (Rice)	FUNCTION: Inactive GDP-bound Rho GTPases reside in the cytosol, are found in a complex with Rho GDP-dissociation inhibitors (Rho GDIs), and are released from the GDI protein in order to translocate to membranes upon activation. {ECO:0000250}.	GO:0003924; GO:0005525; GO:0005634; GO:0005737; GO:0005856; GO:0005886; GO:0005938; GO:0007015; GO:0007163; GO:0007264; GO:0008360; GO:0019901; GO:0030036; GO:0030865; GO:0031410; GO:0031982; GO:0032956; GO:0042995; GO:0043231	actin cytoskeleton organization [GO:0030036]; actin filament organization [GO:0007015]; cortical cytoskeleton organization [GO:0030865]; establishment or maintenance of cell polarity [GO:0007163]; regulation of actin cytoskeleton organization [GO:0032956]; regulation of cell shape [GO:0008360]; small GTPase mediated signal transduction [GO:0007264]
Pavir.4KG256700	Pavir.4KG256700.v5.1	Chr04K	23211904	23212221		#N/A	#N/A	#N/A	#N/A	#N/A	#N/A	#N/A
Pavir.4KG256705	Pavir.4KG256705.v5.1	Chr04K	23215079	23216525	(1 of 3) PTHR23029//PTHR23029:SF51 - PHOSPHOGLYCERATE MUTASE // PHOSPHOGLYCERATE MUTASE	PGML4_ARATH	Phosphoglycerate mutase-like protein 4	At3g50520 T20E23.120	Arabidopsis thaliana (Mouse-ear cress)	FUNCTION: May play a role in carbohydrates metabolism. {ECO:0000250|UniProtKB:Q9MAA2}.	GO:0005737; GO:0016791; GO:0016868	
Pavir.4KG108300	Pavir.4KG108300.v5.1	Chr04K	23245134	23248625	(1 of 2) PTHR13301:SF40 - CELLULOSE SYNTHASE-LIKE PROTEIN D4	CSLD5_ORYSJ	Cellulose synthase-like protein D5 (EC 2.4.1.-) (OsCslD5)	CSLD5 Os06g033650 0 LOC_Os06g22 980 OsJ_020361 P0427E01.10	Oryza sativa subsp. japonica (Rice)	FUNCTION: Thought to be a Golgi-localized beta-glycan synthase that polymerize the backbones of noncellulosic polysaccharides (hemicelluloses) of plant cell wall.	GO:0000139; GO:0005794; GO:0005802; GO:0005886; GO:0009833; GO:0016021; GO:0016760; GO:0030244; GO:0051753; GO:0071555	cellulose biosynthetic process [GO:0030244]; cell wall organization [GO:0071555]; plant-type primary cell wall biogenesis [GO:0009833]
Pavir.4KG108305	Pavir.4KG108305.v5.1	Chr04K	23257260	23257745		#N/A	#N/A	#N/A	#N/A	#N/A	#N/A	#N/A
Pavir.4KG108310	Pavir.4KG108310.v5.1	Chr04K	23267857	23270089		#N/A	#N/A	#N/A	#N/A	#N/A	#N/A	#N/A
Pavir.4KG108315	Pavir.4KG108315.v5.1	Chr04K	23270090	23271634		#N/A	#N/A	#N/A	#N/A	#N/A	#N/A	#N/A
Pavir.4KG108200	Pavir.4KG108200.v5.1	Chr04K	23271977	23273643	(1 of 2) PTHR19139//PTHR19139:SF99 - AQUAPORIN TRANSPORTER // AQUAPORIN TIP2-1	TIP22_ORYSJ	Probable aquaporin TIP2-2 (Tonoplast intrinsic protein 2-2) (OsTIP2;2)	TIP2-2 Os06g033620 0 LOC_Os06g22 960 OsJ_020360 OSJNBa0012F 14.45-1 P0427E01.5-1	Oryza sativa subsp. japonica (Rice)	FUNCTION: Aquaporins facilitate the transport of water and small neutral solutes across cell membranes. May be involved in transport from the vacuolar compartment to the cytoplasm (By similarity). {ECO:0000250}.	GO:0005774; GO:0015250; GO:0016021	
Pavir.4KG108205	Pavir.4KG108205.v5.1	Chr04K	23279737	23280090		#N/A	#N/A	#N/A	#N/A	#N/A	#N/A	#N/A
Pavir.4KG108210	Pavir.4KG108210.v5.1	Chr04K	23289769	23292426		#N/A	#N/A	#N/A	#N/A	#N/A	#N/A	#N/A
Pavir.4KG108215	Pavir.4KG108215.v5.1	Chr04K	23292901	23299119	(1 of 5) 2.7.7.8 - Polyribonucleotide nucleotidyltransferase / Polynucleotide phosphorylase	PNP1_ORYSJ	Probable polyribonucleotide nucleotidyltransferase 1, chloroplastic (EC 2.7.7.8) (Polynucleotide phosphorylase 1) (PNPase 1)	PNP1 Os07g016800 0 LOC_Os07g07 310 OSJNBa0050F 10.4	Oryza sativa subsp. japonica (Rice)	FUNCTION: Involved in the metabolism of all major classes of plastid RNAs. Required for efficient 3'-end processing of mRNAs and 3'-end maturation of rRNA transcripts, but is not sufficient to mediate their degradation. Mediates tRNA degradation. May function as a poly(A) mRNA 3'-5' degrading phosphorylase (By similarity). {ECO:0000250}.	GO:0000175; GO:0000957; GO:0000958; GO:0000963; GO:0000965; GO:0003723; GO:0004654; GO:0005739; GO:0005829; GO:0006364; GO:0006397; GO:0006401; GO:0008033; GO:0009507; GO:0009570; GO:0010323; GO:0015995; GO:0016036; GO:0016120; GO:0016123; GO:0031425	carotene biosynthetic process [GO:0016120]; cellular response to phosphate starvation [GO:0016036]; chlorophyll biosynthetic process [GO:0015995]; chloroplast RNA processing [GO:0031425]; mitochondrial mRNA catabolic process [GO:0000958]; mitochondrial RNA 3'-end processing [GO:0000965]; mitochondrial RNA catabolic process [GO:0000957]; mitochondrial RNA processing [GO:0000963]; mRNA processing [GO:0006397]; negative regulation of isopentenyl diphosphate biosynthetic process, methylerythritol 4-phosphate pathway [GO:0010323]; RNA catabolic process [GO:0006401]; rRNA processing [GO:0006364]; tRNA processing [GO:0008033]; xanthophyll biosynthetic process [GO:0016123]
Pavir.4KG181500	Pavir.4KG181500.v5.1	Chr04K	23310437	23311727	(1 of 44) K08235 - xyloglucan:xyloglucosyl transferase (E2.4.1.207)	XTH26_ARATH	Probable xyloglucan endotransglucosylase/hydrolase protein 26 (At-XTH26) (XTH-26) (EC 2.4.1.207)	XTH26 XTR18 At4g28850 F16A16.40	Arabidopsis thaliana (Mouse-ear cress)	FUNCTION: Catalyzes xyloglucan endohydrolysis (XEH) and/or endotransglycosylation (XET). Cleaves and religates xyloglucan polymers, an essential constituent of the primary cell wall, and thereby participates in cell wall construction of growing tissues (By similarity). {ECO:0000250}.	GO:0004553; GO:0005618; GO:0010411; GO:0016762; GO:0042546; GO:0048046; GO:0071555	cell wall biogenesis [GO:0042546]; cell wall organization [GO:0071555]; xyloglucan metabolic process [GO:0010411]
Pavir.4KG181600	Pavir.4KG181600.v5.1	Chr04K	23344547	23346302	(1 of 53) PF08263//PF13855 - Leucine rich repeat N-terminal domain (LRRNT_2) // Leucine rich repeat (LRR_8)	#N/A	#N/A	#N/A	#N/A	#N/A	#N/A	#N/A
Pavir.4KG181605	Pavir.4KG181605.v5.1	Chr04K	23398778	23399510		#N/A	#N/A	#N/A	#N/A	#N/A	#N/A	#N/A
Pavir.4KG181610	Pavir.4KG181610.v5.1	Chr04K	23401190	23401963		#N/A	#N/A	#N/A	#N/A	#N/A	#N/A	#N/A
Pavir.4KG181615	Pavir.4KG181615.v5.1	Chr04K	23457703	23458647	(1 of 21) PF03732 - Retrotransposon gag protein (Retrotrans_gag)	#N/A	#N/A	#N/A	#N/A	#N/A	#N/A	#N/A
Pavir.4KG181900	Pavir.4KG181900.v5.1	Chr04K	23562532	23565531	(1 of 50) K14484 - auxin-responsive protein IAA (IAA)	IAA21_ORYSJ	Auxin-responsive protein IAA21 (Indoleacetic acid-induced protein 21)	IAA21 Os06g033550 0 LOC_Os06g22 870 OSJNBa0012F 14.28	Oryza sativa subsp. japonica (Rice)	FUNCTION: Aux/IAA proteins are short-lived transcriptional factors that function as repressors of early auxin response genes at low auxin concentrations. {ECO:0000250}.	GO:0005634; GO:0006355; GO:0009733; GO:0009734	auxin-activated signaling pathway [GO:0009734]; regulation of transcription, DNA-templated [GO:0006355]; response to auxin [GO:0009733]

Supplementary Table S5. Flowering time of *PvHd1* allele-overexpressing T_2 plants in CO-null L*er*, L*er* and CS175 (CO-null)

Plant	Arabidopsis line	Batch	Germination	Bolting date	PvHd1	DaysToFlowering
AP13_T1_1_10	AP13_T1_1	1	6/23/21	8/2/21	Present	40
AP13_T1_1_11	AP13_T1_1	1	6/23/21	7/28/21	Present	35
AP13_T1_1_12	AP13_T1_1	1	6/23/21	7/23/21	Present	30
AP13_T1_1_13	AP13_T1_1	1	6/23/21	7/24/21	Present	31
AP13_T1_1_14	AP13_T1_1	1	6/23/21	7/23/21	Present	30
AP13_T1_1_16	AP13_T1_1	1	6/23/21	8/5/21	Present	43
AP13_T1_1_17	AP13_T1_1	1	6/23/21	7/27/21	Present	34
AP13_T1_1_18	AP13_T1_1	1	6/23/21	8/2/21	Present	40
AP13_T1_1_2	AP13_T1_1	1	6/23/21	7/26/21	Present	33
AP13_T1_1_20	AP13_T1_1	1	6/23/21	7/26/21	Present	33
AP13_T1_1_21	AP13_T1_1	1	6/23/21	8/6/21	Present	44
AP13_T1_1_22	AP13_T1_1	1	6/23/21	7/26/21	Present	33
AP13_T1_1_23	AP13_T1_1	1	6/23/21	7/30/21	Present	37
AP13_T1_1_24	AP13_T1_1	1	6/23/21	7/28/21	Present	35
AP13_T1_1_3	AP13_T1_1	1	6/23/21	8/2/21	Present	40
AP13_T1_1_4	AP13_T1_1	1	6/23/21	7/27/21	Present	34
AP13_T1_1_5	AP13_T1_1	1	6/23/21	8/2/21	Present	40
AP13_T1_1_6	AP13_T1_1	1	6/23/21	7/22/21	Present	29
AP13_T1_1_7	AP13_T1_1	1	6/23/21	8/2/21	Present	40
AP13_T1_1_8	AP13_T1_1	1	6/23/21	7/31/21	Present	38
AP13_T1_1_9	AP13_T1_1	1	6/23/21	8/6/21	Present	44
AP13_T1_1_5	AP13_T1_1	2	9/17/21	10/19/21	Present	32
AP13_T1_1_8	AP13_T1_1	2	9/17/21	10/19/21	Present	32
AP13_T1_1_10	AP13_T1_1	2	9/17/21	10/19/21	Present	32
AP13_T1_1_11	AP13_T1_1	2	9/17/21	10/19/21	Present	32
AP13_T1_1_12	AP13_T1_1	2	9/17/21	10/19/21	Present	32
AP13_T1_1_6	AP13_T1_1	2	9/17/21	10/22/21	Present	35
AP13_T1_1_2	AP13_T1_1	2	9/17/21	10/23/21	Present	36
AP13_T1_1_7	AP13_T1_1	2	9/17/21	10/23/21	Present	36
AP13_T1_1_3	AP13_T1_1	2	9/17/21	10/25/21	Present	38
AP13_T1_1_4	AP13_T1_1	2	9/17/21	10/25/21	Present	38
AP13_T1_1_1	AP13_T1_1	1	6/23/21	8/5/21	Absent	43
AP13_T1_1_15	AP13_T1_1	1	6/23/21	7/30/21	Absent	37
AP13_T1_1_19	AP13_T1_1	1	6/23/21	8/2/21	Absent	40
AP13_T1_1_9	AP13_T1_1	2	9/17/21	10/19/21	Absent	32
AP13_T1_1_1	AP13_T1_1	2	9/17/21	10/26/21	Absent	39
AP13_T1_3_B	AP13_T1_3	1	6/23/21	7/18/21	Present	25
AP13_T1_3_22	AP13_T1_3	1	6/23/21	7/20/21	Present	27
AP13_T1_3_10	AP13_T1_3	1	6/23/21	7/21/21	Present	28
AP13_T1_3_13	AP13_T1_3	1	6/23/21	7/21/21	Present	28
AP13_T1_3_14	AP13_T1_3	1	6/23/21	7/21/21	Present	28
AP13_T1_3_21	AP13_T1_3	1	6/23/21	7/21/21	Present	28
AP13_T1_3_7	AP13_T1_3	1	6/23/21	7/21/21	Present	28
AP13_T1_3_11	AP13_T1_3	1	6/23/21	7/22/21	Present	29
AP13_T1_3_15	AP13_T1_3	1	6/23/21	7/22/21	Present	29
AP13_T1_3_18	AP13_T1_3	1	6/23/21	7/22/21	Present	29
AP13_T1_3_1	AP13_T1_3	1	6/23/21	7/23/21	Present	30
AP13_T1_3_2	AP13_T1_3	1	6/23/21	7/23/21	Present	30
AP13_T1_3_20	AP13_T1_3	1	6/23/21	7/23/21	Present	30
AP13_T1_3_4	AP13_T1_3	1	6/23/21	7/23/21	Present	30
AP13_T1_3_16	AP13_T1_3	1	6/23/21	7/24/21	Present	31

AP13_T1_3_23	AP13_T1_3	1	6/23/21	7/24/21	Present	31
AP13_T1_3_5	AP13_T1_3	1	6/23/21	7/24/21	Present	31
AP13_T1_3_19	AP13_T1_3	1	6/23/21	7/26/21	Present	33
AP13_T1_3_9	AP13_T1_3	1	6/23/21	7/27/21	Present	34
AP13_T1_3_12	AP13_T1_3	1	6/23/21	7/30/21	Present	37
AP13_T1_3_17	AP13_T1_3	1	6/23/21	7/30/21	Present	37
AP13_T1_3_24	AP13_T1_3	1	6/23/21	7/30/21	Present	37
AP13_T1_3_3	AP13_T1_3	1	6/23/21	8/2/21	Present	40
AP13_T1_3_8	AP13_T1_3	1	6/23/21	8/2/21	Present	40
AP13_T1_3_6	AP13_T1_3	1	6/23/21	7/30/21	Absent	37
AP13_T1_5_1	AP13_T1_5	1	6/23/21	7/28/21	Present	35
AP13_T1_5_10	AP13_T1_5	1	6/23/21	7/30/21	Present	37
AP13_T1_5_11	AP13_T1_5	1	6/23/21	7/27/21	Present	34
AP13_T1_5_12	AP13_T1_5	1	6/23/21	7/24/21	Present	31
AP13_T1_5_13	AP13_T1_5	1	6/23/21	7/28/21	Present	35
AP13_T1_5_15	AP13_T1_5	1	6/23/21	8/2/21	Present	40
AP13_T1_5_16	AP13_T1_5	1	6/23/21	8/2/21	Present	40
AP13_T1_5_17	AP13_T1_5	1	6/23/21	7/29/21	Present	36
AP13_T1_5_18	AP13_T1_5	1	6/23/21	8/5/21	Present	43
AP13_T1_5_2	AP13_T1_5	1	6/23/21	7/31/21	Present	38
AP13_T1_5_3	AP13_T1_5	1	6/23/21	7/28/21	Present	35
AP13_T1_5_6	AP13_T1_5	1	6/23/21	7/27/21	Present	34
AP13_T1_5_7	AP13_T1_5	1	6/23/21	7/27/21	Present	34
AP13_T1_5_8	AP13_T1_5	1	6/23/21	7/30/21	Present	37
AP13_T1_5_14	AP13_T1_5	1	6/23/21	7/26/21	Absent	33
AP13_T1_5_4	AP13_T1_5	1	6/23/21	7/28/21	Absent	35
AP13_T1_5_5	AP13_T1_5	1	6/23/21	7/29/21	Absent	36
AP13_T1_5_9	AP13_T1_5	1	6/23/21	8/2/21	Absent	40
AP13_T1_6_10	AP13_T1_6	1	6/23/21	7/23/21	Present	30
AP13_T1_6_13	AP13_T1_6	1	6/23/21	7/22/21	Present	29
AP13_T1_6_14	AP13_T1_6	1	6/23/21	7/22/21	Present	29
AP13_T1_6_11	AP13_T1_6	1	6/23/21	7/21/21	Present	28
AP13_T1_6_2	AP13_T1_6	1	6/23/21	7/21/21	Present	28
AP13_T1_6_4	AP13_T1_6	1	6/23/21	7/21/21	Present	28
AP13_T1_6_6	AP13_T1_6	1	6/23/21	7/21/21	Present	28
AP13_T1_6_8	AP13_T1_6	1	6/23/21	7/21/21	Present	28
AP13_T1_6_12	AP13_T1_6	1	6/23/21	7/20/21	Present	27
AP13_T1_6_7	AP13_T1_6	1	6/23/21	7/20/21	Present	27
AP13_T1_6_1	AP13_T1_6	2	9/17/21	10/11/21	Present	24
AP13_T1_6_4	AP13_T1_6	2	9/17/21	10/12/21	Present	25
AP13_T1_6_5	AP13_T1_6	2	9/17/21	10/14/21	Present	27
AP13_T1_6_6	AP13_T1_6	2	9/17/21	10/11/21	Present	24
AP13_T1_6_9	AP13_T1_6	2	9/17/21	10/13/21	Present	26
AP13_T1_6_10	AP13_T1_6	2	9/17/21	10/18/21	Present	31
AP13_T1_6_11	AP13_T1_6	2	9/17/21	10/10/21	Present	23
AP13_T1_6_12	AP13_T1_6	2	9/17/21	10/10/21	Present	23
AP13_T1_6_9	AP13_T1_6	1	6/23/21	8/4/21	Absent	42
AP13_T1_6_5	AP13_T1_6	1	6/23/21	8/2/21	Absent	40
AP13_T1_6_1	AP13_T1_6	1	6/23/21	7/30/21	Absent	37
AP13_T1_6_2	AP13_T1_6	2	9/17/21	10/23/21	Absent	36
AP13_T1_6_3	AP13_T1_6	2	9/17/21	10/23/21	Absent	36
AP13_T1_6_8	AP13_T1_6	2	9/17/21	10/19/21	Absent	32
AP13_T1_8_1	AP13_T1_8	1	6/23/21	7/31/21	Present	38

AP13_T1_8_11	AP13_T1_8	1	6/23/21	7/30/21	Present	37
AP13_T1_8_13	AP13_T1_8	1	6/23/21	7/30/21	Present	37
AP13_T1_8_14	AP13_T1_8	1	6/23/21	7/19/21	Present	26
AP13_T1_8_15	AP13_T1_8	1	6/23/21	8/2/21	Present	40
AP13_T1_8_17	AP13_T1_8	1	6/23/21	7/24/21	Present	31
AP13_T1_8_18	AP13_T1_8	1	6/23/21	8/3/21	Present	41
AP13_T1_8_19	AP13_T1_8	1	6/23/21	8/2/21	Present	40
AP13_T1_8_2	AP13_T1_8	1	6/23/21	7/21/21	Present	28
AP13_T1_8_20	AP13_T1_8	1	6/23/21	8/5/21	Present	43
AP13_T1_8_3	AP13_T1_8	1	6/23/21	7/21/21	Present	28
AP13_T1_8_4	AP13_T1_8	1	6/23/21	7/31/21	Present	38
AP13_T1_8_5	AP13_T1_8	1	6/23/21	7/31/21	Present	38
AP13_T1_8_6	AP13_T1_8	1	6/23/21	7/26/21	Present	33
AP13_T1_8_7	AP13_T1_8	1	6/23/21	7/27/21	Present	34
AP13_T1_8_8	AP13_T1_8	1	6/23/21	7/21/21	Present	28
AP13_T1_8_9	AP13_T1_8	1	6/23/21	7/27/21	Present	34
AP13_T1_8_3	AP13_T1_8	2	9/17/21	10/23/21	Present	36
AP13_T1_8_4	AP13_T1_8	2	9/17/21	10/23/21	Present	36
AP13_T1_8_5	AP13_T1_8	2	9/17/21	10/20/21	Present	33
AP13_T1_8_6	AP13_T1_8	2	9/17/21	10/18/21	Present	31
AP13_T1_8_7	AP13_T1_8	2	9/17/21	10/18/21	Present	31
AP13_T1_8_8	AP13_T1_8	2	9/17/21	10/18/21	Present	31
AP13_T1_8_10	AP13_T1_8	2	9/17/21	10/12/21	Present	25
AP13_T1_8_11	AP13_T1_8	2	9/17/21	10/19/21	Present	32
AP13_T1_8_12	AP13_T1_8	2	9/17/21	10/25/21	Present	38
AP13_T1_8_10	AP13_T1_8	1	6/23/21	8/2/21	Absent	40
AP13_T1_8_12	AP13_T1_8	1	6/23/21	7/31/21	Absent	38
AP13_T1_8_16	AP13_T1_8	1	6/23/21	7/30/21	Absent	37
AP13_T1_8_1	AP13_T1_8	2	9/17/21	10/25/21	Absent	38
AP13_T1_8_2	AP13_T1_8	2	9/17/21	10/22/21	Absent	35
CS175_1	CS175	1	6/23/21	7/29/21	Absent	36
CS175_2	CS175	1	6/23/21	7/31/21	Absent	38
CS175_3	CS175	1	6/23/21	8/6/21	Absent	44
CS175_1	CS175	2	9/22/21	10/25/21	Absent	33
CS175_2	CS175	2	9/22/21	10/26/21	Absent	34
CS175_3	CS175	2	9/22/21	10/30/21	Absent	38
CS175_4	CS175	2	9/22/21	11/1/21	Absent	40
CS175_5	CS175	2	9/22/21	11/1/21	Absent	40
Ler1	Ler	1	6/23/21	7/12/21	CONSTANS p	19
Ler2	Ler	1	6/23/21	7/12/21	CONSTANS p	19
Ler3	Ler	1	6/23/21	7/12/21	CONSTANS p	19
Ler4	Ler	1	6/23/21	7/12/21	CONSTANS p	19
Ler5	Ler	1	6/23/21	7/15/21	CONSTANS p	22
Ler6	Ler	1	6/23/21	7/15/21	CONSTANS p	22
Ler7	Ler	1	6/23/21	7/15/21	CONSTANS p	22
Ler1	Ler	2	9/20/21	10/7/21	CONSTANS p	17
Ler2	Ler	2	9/20/21	10/7/21	CONSTANS p	17
Ler3	Ler	2	9/20/21	10/8/21	CONSTANS p	18
Ler4	Ler	2	9/20/21	10/9/21	CONSTANS p	19
Ler5	Ler	2	9/20/21	10/9/21	CONSTANS p	19
Ler6	Ler	2	9/20/21	10/11/21	CONSTANS p	21
Su_T1_1_1	Su_T1_1	1	6/23/21	7/13/21	Present	20
Su_T1_1_10	Su_T1_1	1	6/23/21	7/15/21	Present	22

Su_T1_1_11	Su_T1_1	1	6/23/21	7/15/21	Present	22
Su_T1_1_12	Su_T1_1	1	6/23/21	7/16/21	Present	23
Su_T1_1_14	Su_T1_1	1	6/23/21	7/15/21	Present	22
Su_T1_1_15	Su_T1_1	1	6/23/21	7/15/21	Present	22
Su_T1_1_16	Su_T1_1	1	6/23/21	7/16/21	Present	23
Su_T1_1_17	Su_T1_1	1	6/23/21	7/13/21	Present	20
Su_T1_1_18	Su_T1_1	1	6/23/21	7/16/21	Present	23
Su_T1_1_19	Su_T1_1	1	6/23/21	7/12/21	Present	19
Su_T1_1_2	Su_T1_1	1	6/23/21	7/13/21	Present	20
Su_T1_1_20	Su_T1_1	1	6/23/21	7/15/21	Present	22
Su_T1_1_21	Su_T1_1	1	6/23/21	7/15/21	Present	22
Su_T1_1_22	Su_T1_1	1	6/23/21	7/15/21	Present	22
Su_T1_1_23	Su_T1_1	1	6/23/21	7/15/21	Present	22
Su_T1_1_24	Su_T1_1	1	6/23/21	7/13/21	Present	20
Su_T1_1_3	Su_T1_1	1	6/23/21	7/15/21	Present	22
Su_T1_1_4	Su_T1_1	1	6/23/21	7/12/21	Present	19
Su_T1_1_5	Su_T1_1	1	6/23/21	7/16/21	Present	23
Su_T1_1_7	Su_T1_1	1	6/23/21	7/15/21	Present	22
Su_T1_1_8	Su_T1_1	1	6/23/21	7/15/21	Present	22
Su_T1_1_1	Su_T1_1	2	9/17/21	10/6/21	Present	19
Su_T1_1_3	Su_T1_1	2	9/17/21	10/6/21	Present	19
Su_T1_1_4	Su_T1_1	2	9/17/21	10/4/21	Present	17
Su_T1_1_5	Su_T1_1	2	9/17/21	10/8/21	Present	21
Su_T1_1_7	Su_T1_1	2	9/17/21	10/6/21	Present	19
Su_T1_1_8	Su_T1_1	2	9/17/21	10/6/21	Present	19
Su_T1_1_10	Su_T1_1	2	9/17/21	10/4/21	Present	17
Su_T1_1_11	Su_T1_1	2	9/17/21	10/8/21	Present	21
Su_T1_13	Su_T1_1	1	6/23/21	7/31/21	Absent	38
Su_T1_6	Su_T1_1	1	6/23/21	7/29/21	Absent	36
Su_T1_9	Su_T1_1	1	6/23/21	7/30/21	Absent	37
Su_T1_1_2	Su_T1_1	2	9/17/21	10/18/21	Absent	31
Su_T1_1_6	Su_T1_1	2	9/17/21	10/19/21	Absent	32
Su_T1_1_12	Su_T1_1	2	9/17/21	10/19/21	Absent	32
Su_T1_1_13	Su_T1_1	2	9/17/21	10/22/21	Absent	35
Su_T1_3_3	Su_T1_3	2	9/17/21	10/4/21	Present	17
Su_T1_3_4	Su_T1_3	2	9/17/21	10/6/21	Present	19
Su_T1_3_5	Su_T1_3	2	9/17/21	10/8/21	Present	21
Su_T1_3_7	Su_T1_3	2	9/17/21	10/8/21	Present	21
Su_T1_3_8	Su_T1_3	2	9/17/21	10/8/21	Present	21
Su_T1_3_9	Su_T1_3	2	9/17/21	10/6/21	Present	19
Su_T1_3_12	Su_T1_3	2	9/17/21	10/6/21	Present	19
Su_T1_3_13	Su_T1_3	2	9/17/21	10/6/21	Present	19
Su_T1_3_14	Su_T1_3	2	9/17/21	10/8/21	Present	21
Su_T1_3_15	Su_T1_3	2	9/17/21	10/6/21	Present	19
Su_T1_3_16	Su_T1_3	2	9/17/21	10/4/21	Present	17
Su_T1_3_17	Su_T1_3	2	9/17/21	10/5/21	Present	18
Su_T1_3_18	Su_T1_3	2	9/17/21	10/6/21	Present	19
Su_T1_3_6	Su_T1_3	2	9/17/21	10/18/21	Absent	31
Su_T1_3_10	Su_T1_3	2	9/17/21	10/19/21	Absent	32
Su_T1_3_11	Su_T1_3	2	9/17/21	10/18/21	Absent	31

Supplementary Table S6. Post-hoc Tukey testing results (Lenth, 2022; R Core Team, 2022) showing the significance of differences in flowering time of *PvHd1* -OE transgenic lines relative to L*er* and CS175 (data shown in Figure 3.a)

Comparisons	estimate	SE	df	t.ratio	p.value
AP13_T1_1 - CS175	-2.9010101	1.32486304	166	-2.1896679	0.41727879
AP13_T1_1 - Ler	15.8926641	1.09607872	166	14.4995645	7.24E-14
AP13_T1_3 - CS175	-8.0787541	1.40533383	166	-5.7486371	1.50E-06
AP13_T1_3 - Ler	10.71492	1.17469523	166	9.12144677	1.67E-13
AP13_T1_5 - CS175	-2.9716113	1.51670587	166	-1.9592535	0.5738352
AP13_T1_5 - Ler	15.8220629	1.3058876	166	12.1159454	7.87E-14
AP13_T1_6 - CS175	-11.350529	1.40961941	166	-8.052194	5.54E-12
AP13_T1_6 - Ler	7.44314519	1.20362392	166	6.18394588	1.68E-07
AP13_T1_8 - CS175	-4.4082134	1.34823728	166	-3.2696125	0.03462874
AP13_T1_8 - Ler	14.3854608	1.12555747	166	12.7807431	7.32E-14
CS175 - Ler	18.7936742	1.48942107	166	12.6181068	7.36E-14
CS175 - Su_T1_1	17.8595109	1.33845979	166	13.3433302	7.24E-14
CS175 - Su_T1_3	17.7719783	1.50427859	166	11.8142865	8.53E-14
Ler - Su_T1_1	-0.9341632	1.10979141	166	-0.8417467	0.99537269
Ler - Su_T1_3	-1.0216959	1.33994783	166	-0.7624893	0.99767421
Su_T1_1 - Su_T1_3	-0.0875327	1.19286979	166	-0.0733799	1

Lenth R v. 2022. emmeans: estimated marginal means, aka least-squares means. https://CRAN.R-project.org/package=emmeans
R Core Team. 2022. R: a language and environment for statistical computing. https://www.R-project.org/.

Supplementary Table S7. Cq values measured in *PvHd1* -overexpressing T_2 transgenic plants in CO-null L*er* plants.

Fluor	Target	Sample	Batch	tech1 Cq	tech2 Cq	tech3 Cq	tech1 dCq	tech2 dCq	tech3 dCq	std_dCq	tech1 dCq	tech2 dCq	tech3 dCq	std_dCq
SYBR	Actin2	AP13_T1_1-	1	21.11	21.61	20.82	6.52	6.63	6.87	0.18	6.52	6.63	6.87	0.1768
SYBR	Actin2	AP13_T1_1-	1	25.07	25.45	24.71	-1.42	-1.47	-1.41	0.03	-1.42	-1.47	-1.41	0.0346
SYBR	Actin2	AP13_T1_1-	1	20.41	20.86	20.16	9.18	9.51	9.88	0.35	9.18	9.51	9.88	0.3485
SYBR	Actin2	AP13_T1_1-	1	20.80	21.23	20.52	7.01	8.05	7.58	0.52	7.01	8.05	7.58	0.5191
SYBR	Actin2	AP13_T1_1-	1	21.44	22.02	21.26	0.75	0.71	0.93	0.11	0.75	0.71	0.93	0.114
SYBR	Actin2	AP13_T1_1-	1	23.89	24.35	23.79	4.21	4.50	4.19	0.17	4.21	4.50	4.19	0.1737
SYBR	Actin2	AP13_T1_3-	1	20.90	21.24	20.69	-1.01	-0.64	-0.72	0.19	-1.01	-0.64	-0.72	0.1937
SYBR	Actin2	AP13_T1_3-	1	19.06	20.50	19.20	2.06		2.82	0.54	2.06	4.79	2.82	1.4058
SYBR	Actin2	AP13_T1_3-	1	21.20	21.64	21.06	-2.44	-2.57	-2.44	0.07	-2.44	-2.57	-2.44	0.0745
SYBR	Actin2	AP13_T1_3-	1	21.26	21.85	20.99	3.45	3.57	3.71	0.13	3.45	3.57	3.71	0.1311
SYBR	Actin2	AP13_T1_3-	1	22.96	23.46	22.55	-4.07	-4.16	-3.95	0.11	-4.07	-4.16	-3.95	0.1062
SYBR	Actin2	AP13_T1_3-	1	20.87	21.29	20.51	11.18	11.48	11.16	0.18	11.18	11.48	11.16	0.1783
SYBR	Actin2	AP13_T1_3-	1	22.51	22.88	22.18	-4.14	-4.46	-4.11	0.20	-4.14	-4.46	-4.11	0.1965
SYBR	Actin2	AP13_T1_5-	1	21.81	22.39	21.56	1.78	2.09	1.93	0.15	1.78	2.09	1.93	0.1516
SYBR	Actin2	AP13_T1_5-	1	21.08	21.47	21.00	2.38	2.52	2.17	0.18	2.38	2.52	2.17	0.1777
SYBR	Actin2	AP13_T1_5-	1	20.25	20.78	20.07	-0.39	-0.26	-0.31	0.07	-0.39	-0.26	-0.31	0.0663
SYBR	Actin2	AP13_T1_5-	1	20.39	20.91	20.12	3.07	3.10	3.49	0.24	3.07	3.10	3.49	0.2356
SYBR	Actin2	AP13_T1_8-	1	21.33	22.07	21.19	-2.97	-3.14	-2.92	0.12	-2.97	-3.14	-2.92	0.1174
SYBR	Actin2	AP13_T1_8-	1	20.37	20.82	20.20	0.78	1.78	1.26	0.50	0.78	1.78	1.26	0.5033
SYBR	Actin2	AP13_T1_8-	1	20.42	20.92	20.31	1.24	1.66	1.31	0.23	1.24	1.66	1.31	0.2266
SYBR	Actin2	AP13_T1_8-	1	22.21	22.81	22.08	-1.70	-0.06	-0.96	0.82	-1.70	-0.06	-0.96	0.8214
SYBR	Actin2	AP13_T1_8-	1	20.21	20.74	20.12	-2.10		-1.98	0.08	-2.10	3.64	-1.98	3.2777
SYBR	Actin2	AP13_T1_8-	1	22.23	22.68	22.03	-2.71	-2.39	-2.43	0.17	-2.71	-2.39	-2.43	0.1719
SYBR	Actin2	Su_T1_1-18	1	21.68	22.17	21.07		10.53	10.26	0.19	5.08	10.53	10.26	3.0725
SYBR	Actin2	Su_T1_1-4	1	19.98	22.34	20.82	-0.27		-0.04	0.16	-0.27	1.31	-0.04	0.8522
SYBR	Actin2	Su_T1_1-8	1	20.49	22.05	20.88		1.87	1.90	0.02	0.64	1.87	1.90	0.7201
SYBR	PvHd1	AP13_T1_1-	1	27.64	28.24	27.69								
SYBR	PvHd1	AP13_T1_1-	1	23.65	23.98	23.30								
SYBR	PvHd1	AP13_T1_1-	1	29.59	30.36	30.04								
SYBR	PvHd1	AP13_T1_1-	1	27.81	29.28	28.10								
SYBR	PvHd1	AP13_T1_1-	1	22.19	22.73	22.19								

SYBR	PvHd1	AP13_T1_1-	1	28.09	28.85	27.99								
SYBR	PvHd1	AP13_T1_3-	1	19.89	20.61	19.96								
SYBR	PvHd1	AP13_T1_3-	1	21.12	25.29	22.02								
SYBR	PvHd1	AP13_T1_3-	1	18.75	19.07	18.62								
SYBR	PvHd1	AP13_T1_3-	1	24.71	25.41	24.70								
SYBR	PvHd1	AP13_T1_3-	1	18.89	19.30	18.60								
SYBR	PvHd1	AP13_T1_3-	1	32.05	32.77	31.67								
SYBR	PvHd1	AP13_T1_3-	1	18.37	18.42	18.07								
SYBR	PvHd1	AP13_T1_5-	1	23.59	24.47	23.49								
SYBR	PvHd1	AP13_T1_5-	1	23.45	23.98	23.17								
SYBR	PvHd1	AP13_T1_5-	1	19.86	20.52	19.76								
SYBR	PvHd1	AP13_T1_5-	1	23.46	24.02	23.62								
SYBR	PvHd1	AP13_T1_8-	1	18.37	18.93	18.27								
SYBR	PvHd1	AP13_T1_8-	1	21.15	22.60	21.46								
SYBR	PvHd1	AP13_T1_8-	1	21.66	22.58	21.61								
SYBR	PvHd1	AP13_T1_8-	1	20.51	22.75	21.12								
SYBR	PvHd1	AP13_T1_8-	1	18.11	24.38	18.14								
SYBR	PvHd1	AP13_T1_8-	1	19.52	20.29	19.59								
SYBR	PvHd1	Su_T1_1-18	1	26.76	32.70	31.33								
SYBR	PvHd1	Su_T1_1-4	1	19.72	23.65	20.79								
SYBR	PvHd1	Su_T1_1-8	1	21.13	23.92	22.78								
SYBR	Actin2	AP13_T1_1_	2	21.60	21.57	21.75	6.27	6.02	6.21	0.13	6.27	6.02	6.21	0.13
SYBR	Actin2	AP13_T1_1_	2	20.62	20.40	20.68	8.48	8.60	8.37	0.12	8.48	8.60	8.37	0.12
SYBR	Actin2	AP13_T1_1_	2	20.45	20.55	20.48	7.33	6.90	7.21	0.22	7.33	6.90	7.21	0.22
SYBR	Actin2	AP13_T1_1_	2	21.51	21.35	21.62	9.53	9.38	9.15	0.19	9.53	9.38	9.15	0.19
SYBR	Actin2	AP13_T1_1_	2	20.10	20.02	20.12	4.14	3.90	4.08	0.13	4.14	3.90	4.08	0.13
SYBR	Actin2	AP13_T1_1_	2	20.41	20.05	20.27	NA	17.58	17.14	0.31	NA	17.58	17.14	0.31
SYBR	Actin2	AP13_T1_6_	2	21.76	21.48	21.79	-1.08	-1.11	-1.17	0.05	-1.08	-1.11	-1.17	0.05
SYBR	Actin2	AP13_T1_6_	2	19.70	19.57	19.77	1.90	1.89	1.84	0.03	1.90	1.89	1.84	0.03
SYBR	Actin2	AP13_T1_6_	2	21.61	21.40	21.61	-2.21	-2.24	-2.22	0.02	-2.21	-2.24	-2.22	0.02
SYBR	Actin2	AP13_T1_6_	2	21.51	21.19	21.36	-3.12	-2.99	-2.92	0.10	-3.12	-2.99	-2.92	0.10
SYBR	Actin2	AP13_T1_6_	2	19.97	19.85	20.04	-2.72	-2.78	-2.80	0.04	-2.72	-2.78	-2.80	0.04
SYBR	Actin2	AP13_T1_6_	2	21.41	21.11	21.39	-1.22	-1.21	-1.28	0.04	-1.22	-1.21	-1.28	0.04
SYBR	Actin2	AP13_T1_6_	2	20.44	20.29	20.51	-1.81	-1.73	-1.73	0.04	-1.81	-1.73	-1.73	0.04
SYBR	Actin2	AP13_T1_8_	2	21.50	21.34	21.53	0.16	-0.14	-0.12	0.17	0.16	-0.14	-0.12	0.17

SYBR	Actin2	AP13_T1_8_	2	20.48	20.28	20.46	-1.58	-1.45	-1.50	0.07	-1.58	-1.45	-1.50	0.07
SYBR	Actin2	AP13_T1_8_	2	20.52	20.29	20.56	-0.86	-0.90	-0.97	0.05	-0.86	-0.90	-0.97	0.05
SYBR	Actin2	AP13_T1_8_	2	20.04	19.68	20.02	0.46	0.63	0.47	0.09	0.46	0.63	0.47	0.09
SYBR	Actin2	AP13_T1_8_	2	21.18	21.02	21.18	0.77	0.73	0.77	0.02	0.77	0.73	0.77	0.02
SYBR	Actin2	AP13_T1_8_	2	21.54	21.38	21.59	-0.08	0.03	-0.08	0.06	-0.08	0.03	-0.08	0.06
SYBR	Actin2	Su_T1_1_10	2	22.99	22.35	23.11	-2.66	-3.42	-3.96	0.66	-2.66	-3.42	-3.96	0.66
SYBR	Actin2	Su_T1_1_11	2	21.32	21.26	21.39	-1.64	-1.76	-1.75	0.07	-1.64	-1.76	-1.75	0.07
SYBR	Actin2	Su_T1_1_3	2	21.78	21.54	21.86	-1.82	-1.96	-2.09	0.13	-1.82	-1.96	-2.09	0.13
SYBR	Actin2	Su_T1_1_5	2	22.63	22.51	22.68	-2.25	-2.24	-2.19	0.03	-2.25	-2.24	-2.19	0.03
SYBR	Actin2	Su_T1_1_7	2	21.92	21.51	21.98	-1.49	-1.37	-1.63	0.13	-1.49	-1.37	-1.63	0.13
SYBR	Actin2	Su_T1_3_16	2	22.74	21.94	22.29	-2.58	-1.98	-2.09	0.32	-2.58	-1.98	-2.09	0.32
SYBR	Actin2	Su_T1_3_17	2	21.74	21.56	21.82	-1.28	-1.76	-1.78	0.28	-1.28	-1.76	-1.78	0.28
SYBR	Actin2	Su_T1_3_3	2	22.34	21.29	21.59		-1.59	-1.70	0.08	-2.50	-1.59	-1.70	0.50
SYBR	Actin2	Su_T1_3_5	2	20.67	20.52	20.76	-2.12	-2.02	-2.10	0.05	-2.12	-2.02	-2.10	0.05
SYBR	Actin2	Su_T1_3_7	2	20.52	20.32	20.57	-1.50	-1.49	-1.46	0.02	-1.50	-1.49	-1.46	0.02
SYBR	Actin2	Su_T1_3_8	2	20.19	20.03	20.24	-1.18	-1.17	-1.17	0.01	-1.18	-1.17	-1.17	0.01
SYBR	PvHd1	AP13_T1_1_	2	27.87	27.59	27.96								
SYBR	PvHd1	AP13_T1_1_	2	29.10	29.00	29.05								
SYBR	PvHd1	AP13_T1_1_	2	27.78	27.46	27.69								
SYBR	PvHd1	AP13_T1_1_	2	31.04	30.72	30.77								
SYBR	PvHd1	AP13_T1_1_	2	24.24	23.91	24.19								
SYBR	PvHd1	AP13_T1_1_	2	NA	37.63	37.41								
SYBR	PvHd1	AP13_T1_6_	2	20.68	20.38	20.62								
SYBR	PvHd1	AP13_T1_6_	2	21.60	21.46	21.61								
SYBR	PvHd1	AP13_T1_6_	2	19.40	19.16	19.39								
SYBR	PvHd1	AP13_T1_6_	2	18.38	18.20	18.44								
SYBR	PvHd1	AP13_T1_6_	2	17.25	17.07	17.24								
SYBR	PvHd1	AP13_T1_6_	2	20.19	19.90	20.11								
SYBR	PvHd1	AP13_T1_6_	2	18.63	18.56	18.78								
SYBR	PvHd1	AP13_T1_8_	2	21.66	21.20	21.41								
SYBR	PvHd1	AP13_T1_8_	2	18.89	18.83	18.96								
SYBR	PvHd1	AP13_T1_8_	2	19.66	19.39	19.59								
SYBR	PvHd1	AP13_T1_8_	2	20.50	20.31	20.49								
SYBR	PvHd1	AP13_T1_8_	2	21.95	21.76	21.95								
SYBR	PvHd1	AP13_T1_8_	2	21.46	21.41	21.52								

SYBR	PvHd1	Su_T1_1_10	2	20.33	18.93	19.15
SYBR	PvHd1	Su_T1_1_11	2	19.68	19.50	19.63
SYBR	PvHd1	Su_T1_1_3	2	19.95	19.58	19.77
SYBR	PvHd1	Su_T1_1_5	2	20.38	20.27	20.50
SYBR	PvHd1	Su_T1_1_7	2	20.43	20.15	20.36
SYBR	PvHd1	Su_T1_3_16	2	20.16	19.95	20.20
SYBR	PvHd1	Su_T1_3_17	2	20.46	19.81	20.04
SYBR	PvHd1	Su_T1_3_3	2	19.85	19.70	19.89
SYBR	PvHd1	Su_T1_3_5	2	18.55	18.50	18.66
SYBR	PvHd1	Su_T1_3_7	2	19.02	18.83	19.10
SYBR	PvHd1	Su_T1_3_8	2	19.01	18.87	19.07

NA indicates no Cq measurement within the 38 cycles.
Orange-filled cells indicate outliers of dCq values across technical replicates which were excluded from averaged dCq values (CqPvHd1-CqActin2).
Column M-P show the calculations without excluding deviated dCqs.

Supplementary Table S8. Days to heading in a switchgrass diversity panel (Lovell *et al.* , 2021) and the p.35 variant present in PvHd1 which locates at position 19,031,195 on Chr04K in the AP13 genome assembly v5.1 (Lovell *et al.* , 2021).

Rep	Year	Accession	p.35 in PvHd1	Daystoheading	Subpopulation	Ecotype
1	2021	J582.C	S/S	63	Midwest	Upland
1	2021	J380.B	S/S	66	Midwest	Upland
1	2021	J368.B	S/S	68	Midwest	Upland
1	2021	J368.A	S/S	68	Midwest	Upland
1	2021	J378.C	S/S	70	Midwest	Upland
1	2021	J348.C	S/S	71	Midwest	Upland
1	2021	J037.A	S/S	73	Midwest	Upland
1	2021	J353.A	S/S	78	Midwest	Upland
1	2021	J368.C	S/S	80	Midwest	Upland
1	2021	J349.A	S/S	83	Midwest	Upland
1	2021	J386.B	S/S	91	Midwest	Upland
1	2021	J421.A	S/S	94	Midwest	Upland
2	2021	J374.A	S/S	45	Midwest	Upland
2	2021	J581.C	S/S	59	Midwest	Upland
2	2021	J582.C	S/S	59	Midwest	Upland
2	2021	J348.C	S/S	59	Midwest	Upland
2	2021	J348.B	S/S	63	Midwest	Upland
2	2021	J349.A	S/S	64	Midwest	Upland
2	2021	J037.A	S/S	66	Midwest	Upland
2	2021	J386.A	S/S	88	Midwest	Upland
2	2021	J491.B	S/S	91	Midwest	Upland
2	2021	J353.A	S/S	91	Midwest	Upland
1	2020	J353.A	S/S	60	Midwest	Upland
1	2020	J380.B	S/S	62	Midwest	Upland
1	2020	J378.C	S/S	62	Midwest	Upland
1	2020	J368.C	S/S	62	Midwest	Upland
1	2020	J348.C	S/S	63	Midwest	Upland
1	2020	J582.C	S/S	65	Midwest	Upland
1	2020	J368.A	S/S	65	Midwest	Upland
1	2020	J368.B	S/S	70	Midwest	Upland
1	2020	J037.A	S/S	70	Midwest	Upland
1	2020	J349.A	S/S	70	Midwest	Upland
1	2020	J421.A	S/S	80	Midwest	Upland
1	2020	J386.B	S/S	104	Midwest	Upland
1	2021	J306.C	G/G	98	Gulf	Coastal
1	2021	J297.A	G/G	102	Gulf	Unknown
1	2021	J307.A	G/G	108	Gulf	Coastal
1	2021	J008.B	G/G	108	Gulf	Lowland
1	2021	J296.A	G/G	109	Gulf	Unknown
1	2021	J018.B	G/G	112	Gulf	Lowland
1	2021	J250.B	G/G	112	Gulf	Lowland
1	2021	J419.A	G/G	112	Gulf	Unknown
1	2021	J223.A	G/G	113	Gulf	Lowland
1	2021	J341.A	G/G	113	Gulf	Lowland
1	2021	J016.B	G/G	113	Gulf	Lowland
1	2021	J018.C	G/G	113	Gulf	Lowland
1	2021	J008.D	G/G	113	Gulf	Lowland
1	2021	J315.A	G/G	115	Gulf	Unknown
1	2021	J016.C	G/G	116	Gulf	Lowland
1	2021	J206.A	G/G	116	Gulf	Lowland
1	2021	J013.C	G/G	116	Gulf	Lowland
1	2021	J441.A	G/G	116	Gulf	Lowland
1	2021	J610.C	G/G	117	Gulf	Lowland
1	2021	J514.A	G/G	117	Gulf	Lowland
1	2021	J610.A	G/G	117	Gulf	Lowland

Supplementary Table S9. Post-hoc Tukey testing results (Lenth, 2022; R Core Team, 2022) showing the significance of differences in days to heading recorded in 2021 (Rep1 and Rep2) for accessions belonging to three genetic subpopulations in a GWAS panel (Lovell *et al.* , 2021) established at the Iron Horse Farm in Watkinsville, GA (data shown in Figure 4.a)

Comparisons	estimate	SE	df	t.ratio	p.value
Atlantic - Gulf	-41.763353	3.01772173	194	-13.839365	1.61E-14
Atlantic - Midwest	15.8173625	4.08712397	194	3.87004715	0.00043491
Gulf - Midwest	57.5807154	3.19009134	194	18.0498642	1.61E-14

Subpopulation membership was obtained from Lovell *et al* . (2021).

Lovell JT, MacQueen AH, Mamidi S, et al. 2021. Genomic mechanisms of climate adaptation in polyploid bioenergy switchgrass. Nature 590, 438–444.
Lenth R v. 2022. emmeans: estimated marginal means, aka least-squares means. https://CRAN.R-project.org/package=emmeans
R Core Team. 2022. R: a language and environment for statistical computing. https://www.R-project.org/.

Supplementary Table S10. Post-hoc Tukey testing results (Lenth, 2022; R Core Team, 2022) showing the significance of differences in days to heading recorded in 2021 (Rep1 and Rep2) for accessions belonging to three ecotypes within genetic subpopulations in a GWAS panel (Lovell *et al.* , 2021) established at the Iron Horse Farm in Watkinsville, GA (data shown in Figure 4.b)

Comparisons	estimate	SE	df	t.ratio	p.value
Atlantic Coastal - Atlantic Lowland	2.1	13.7972259	191	0.15220451	0.99999999
Atlantic Coastal - Atlantic Upland	26.7428571	5.44674489	191	4.90987878	6.75E-05
Atlantic Coastal - Gulf Coastal	-31.030435	4.98297242	191	-6.2272941	1.06E-07
Atlantic Coastal - Gulf Lowland	-25.923622	4.32069743	191	-5.9998698	3.47E-07
Atlantic Coastal - Midwest Upland	30.8272727	5.01717304	191	6.14435111	1.64E-07
Atlantic Lowland - Atlantic Upland	24.6428571	13.6168622	191	1.80973096	0.67573487
Atlantic Lowland - Gulf Lowland	-28.023622	13.206829	191	-2.1219039	0.46161177
Atlantic Lowland - Midwest Upland	28.7272727	13.4507967	191	2.1357302	0.4523169
Atlantic Upland - Midwest Upland	4.08441558	4.49750157	191	0.90815212	0.99230607
Gulf Coastal - Atlantic Lowland	33.1304348	13.4380773	191	2.46541481	0.25570096
Gulf Coastal - Atlantic Upland	57.7732919	4.45931712	191	12.9556366	0
Gulf Coastal - Gulf Lowland	5.10681274	2.98109134	191	1.71306819	0.7378636
Gulf Coastal - Midwest Upland	61.8577075	3.92307336	191	15.7676652	0
Gulf Lowland - Atlantic Upland	52.6664792	3.70458112	191	14.2165814	0
Gulf Lowland - Midwest Upland	56.7508948	3.03791323	191	18.6808808	0

Ecotype and subpopulation membership was obtained from Lovell *et al.* (2021)

Lovell JT, MacQueen AH, Mamidi S, et al. 2021. Genomic mechanisms of climate adaptation in polyploid bioenergy switchgrass. Nature 590, 438–444.
Lenth R v. 2022. emmeans: estimated marginal means, aka least-squares means. https://CRAN.R-project.org/package=emmeans
R Core Team. 2022. R: a language and environment for statistical computing. https://www.R-project.org/.

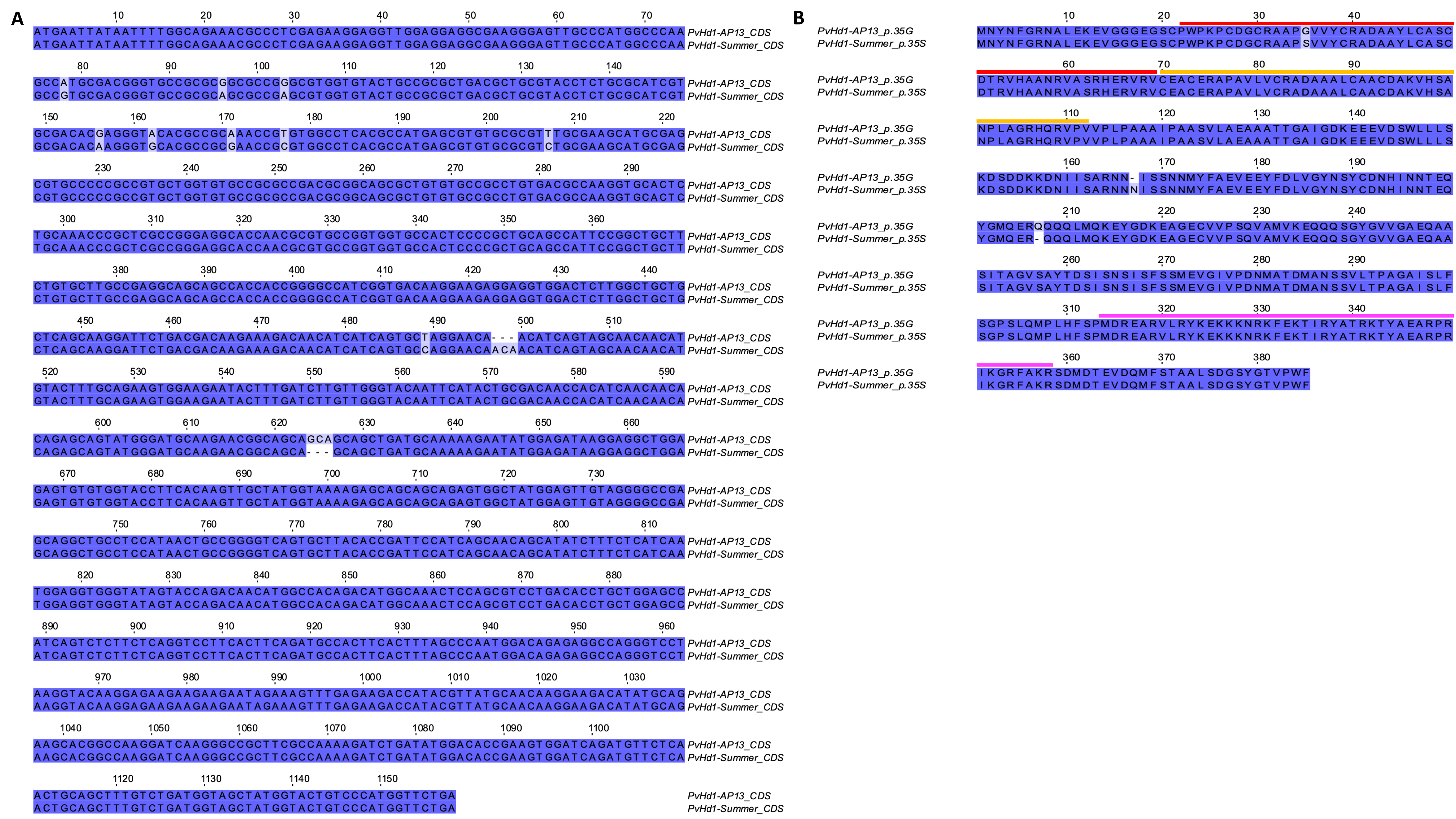
A
PvHd1-AP13_CDS
PvHd1-Summer_CDS
B
PvHd1-AP13_p.35G
PvHd1-Summer_p.35S

C

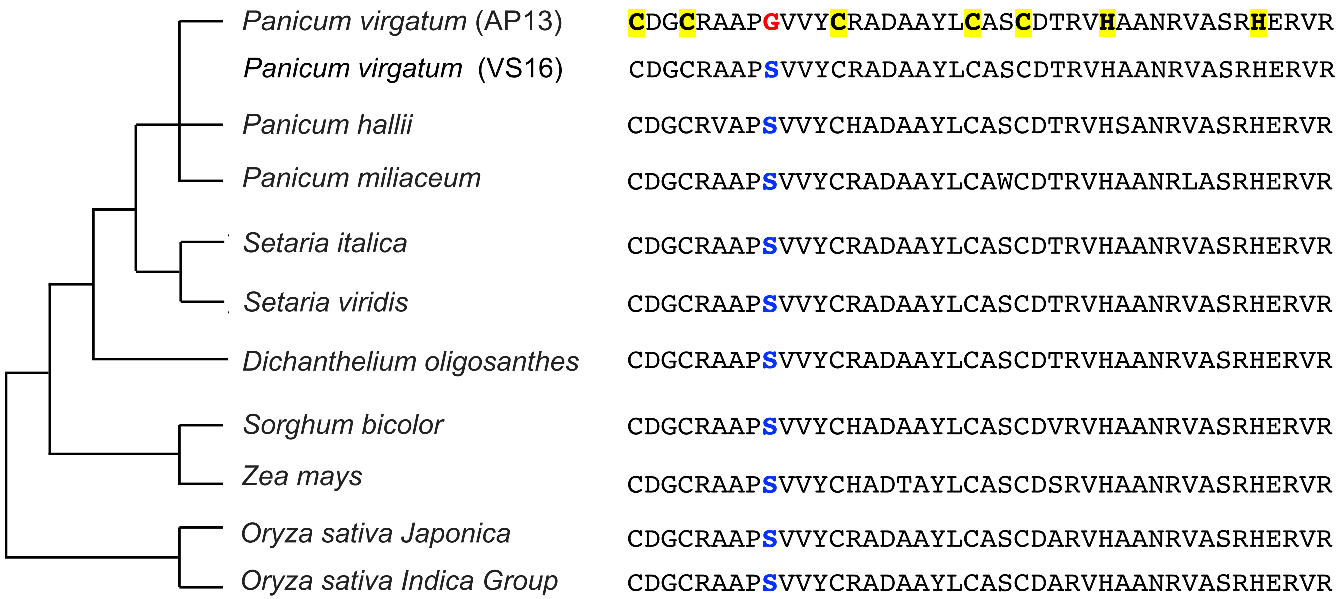

Supplementary Fig. S1. Amino acid and nucleic acid variation in PvHd1 **(A)** Jalview alignment (Waterhouse *et al.*, 2009; Troshin *et al.*, 2011, 2018) showing coding sequences of PvHd1-p.35G and PvHd1-p.35S. **(B)** Jalview alignment (Waterhouse *et al.*, 2009; Troshin *et al.*, 2011, 2018) showing the full-length protein sequences of PvHd1-p.35G (AP13 allele) and PvHd1-p.35S (Summer allele). Red, orange and magenta lines above the sequences indicate B-Box domain 1 (SMART ID: SM00336), B-Box domain 2 (SMART ID: SM00336) and CCT domain (Pfam ID: PF06203), respectively. **(C)** Aligned sequences of B-box domain 1 (p.27 to p.68) across related grass species. Phylogenetic relationships were drawn according to Rao and Dixon (2016). Yellow shaded amino acids indicate the motifs (C-X2-C-X8-C-X?-C-X2-C-X4-H-X8-H) of B-Box domain 1 identified in rice where protein-protein interactions occur (Khanna *et al.*, 2009; Huang *et al.*, 2012; Gangappa and Botto, 2014). The G in red shows the serine to glycine substitution (p.S35G) in lowland switchgrass AP13.

Gangappa SN, Botto JF. 2014. The BBX family of plant transcription factors. Trends in Plant Science 19, 460–470.

Huang J, Zhao X, Weng X, Wang L, Xie W. 2012. The Rice B-Box Zinc Finger Gene Family: Genomic Identification, Characterization, Expression Profiling and Diurnal Analysis (L-SP Tran, Ed.). PLoS ONE 7, e48242.

Khanna R, Kronmiller B, Maszle DR, Coupland G, Holm M, Mizuno T, Wu S-H. 2009. The

Rao X, Dixon RA. 2016. The Differences between NAD-ME and NADP-ME Subtypes of C4 Photosynthesis: More than Decarboxylating Enzymes. Frontiers in Plant Science 7.

Troshin P V, Procter JB, Barton GJ. 2011. Java bioinformatics analysis web services for multiple sequence alignment--JABAWS:MSA. Bioinformatics 27, 2001–2002.

Troshin P V, Procter JB, Sherstnev A, Barton DL, Madeira F, Barton GJ. 2018. JABAWS 2.2 distributed web services for Bioinformatics: protein disorder, conservation and RNA secondary structure (J Hancock, Ed.). Bioinformatics 34, 1939–1940.

Waterhouse AM, Procter JB, Martin DMA, Clamp M, Barton GJ. 2009. Jalview Version 2--a multiple sequence alignment editor and analysis workbench. Bioinformatics 25, 1189–1191.

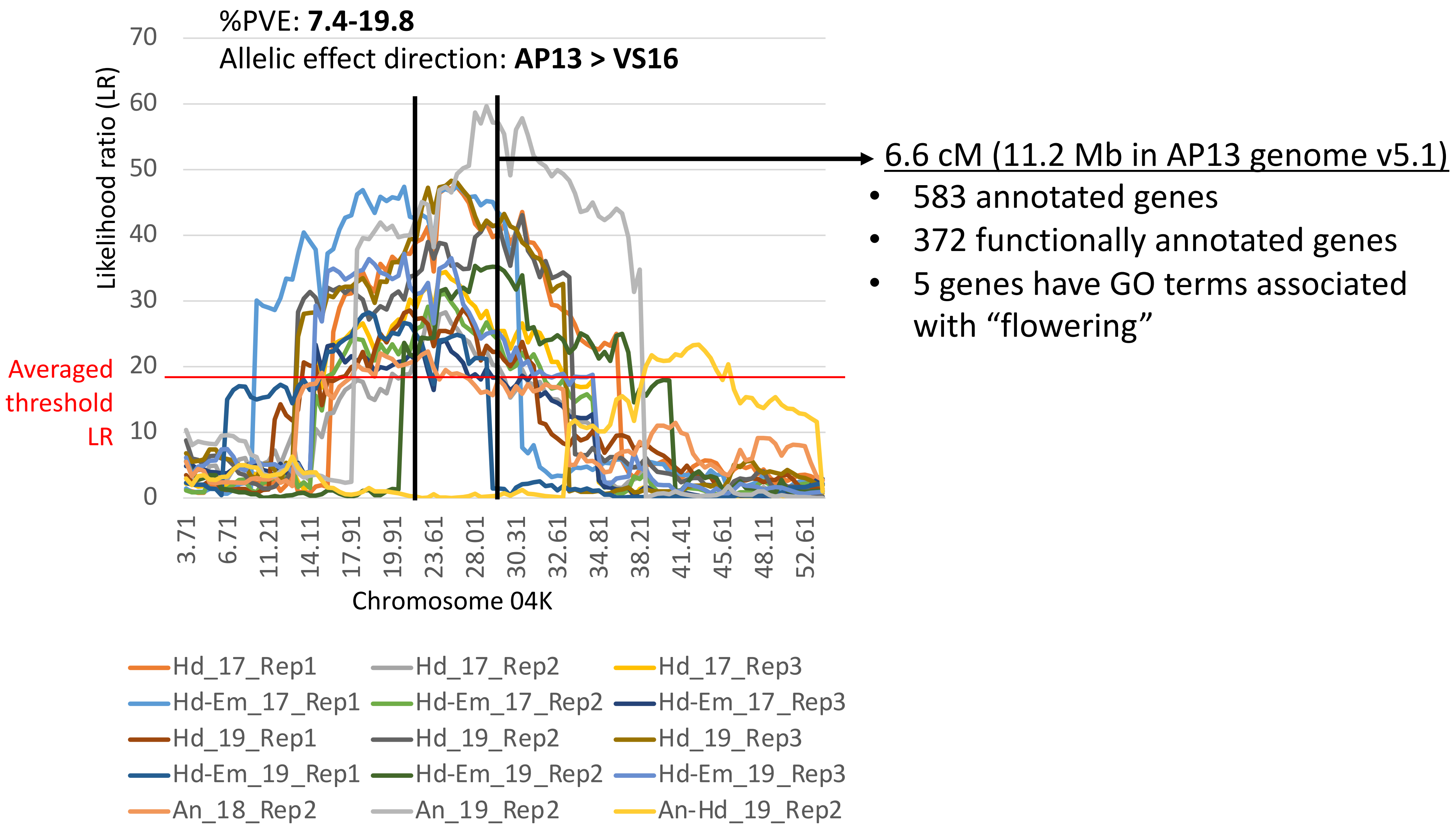

Supplementary Fig. S2. Flowering time QTL identified on Chr04K. The X-axis indicates the genetic position of the markers on Chr04K and the Y-axis indicates the likelihood ratio (LR) of association between the loci and the trait. Solid lines indicate the LR of association between markers and ordinal heading dates (Hd), days to heading (Hd-Em), ordinal anthesis dates (An) and days to anthesis (An-Hd). Hd = Heading date; Hd-Em = time interval between spring emergence and heading (days to heading). An = Anthesis date; An-Hd = time interval between heading and anthesis (days to anthesis). The red horizontal line indicates the averaged threshold LR value (range 17.7 – 19.6) across the QTL at $p < 0.05$ after 500 permutations. PVE=percent variation explained.

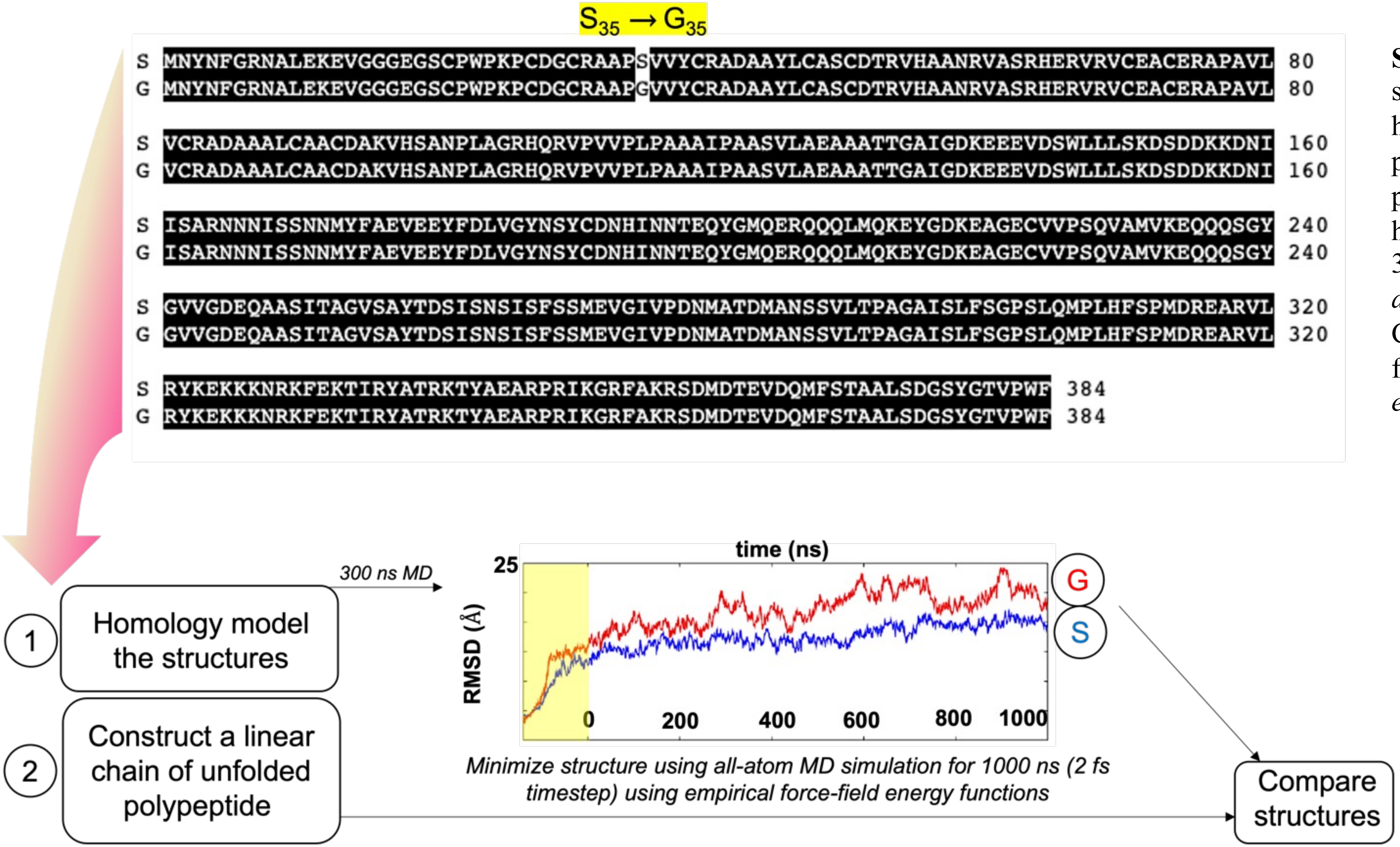

Supplementary Fig. S3. The amino acid sequence of the proteins was used to build homology models. A linear chain of unfolded polypeptide was constructed for parts of the proteins not built in the homology models. The homology models were then subjected to a 300ns MD simulation using NAMD (Phillips *et al.*, 2005; Huang and MacKerell, 2013) and CHARMM36 (Huang and MacKerell, 2013) forcefield followed by 1000ns FG-MD (Zhang *et al.*, 2011) using I-TASSER (Yang *et al.*, 2014)

Huang J, MacKerell AD. 2013. CHARMM36 all-atom additive protein force field: validation based on comparison to NMR data. Journal of computational chemistry 34, 2135–2145.
Phillips JC, Braun R, Wang W, Gumbart J, Tajkhorshid E, Villa E, Chipot C, Skeel RD, Kalé L, Schulten K. 2005. Scalable molecular dynamics with NAMD. Journal of Computational Chemistry 26, 1781–1802.
Yang J, Yan R, Roy A, Xu D, Poisson J, Zhang Y. 2014. The I-TASSER Suite: protein structure and function prediction. Nature Methods 12, 7–8.
Zhang J, Liang Y, Zhang Y. 2011. Atomic-Level Protein Structure Refinement Using Fragment-Guided Molecular Dynamics Conformation Sampling. Structure 19, 1784–1795.

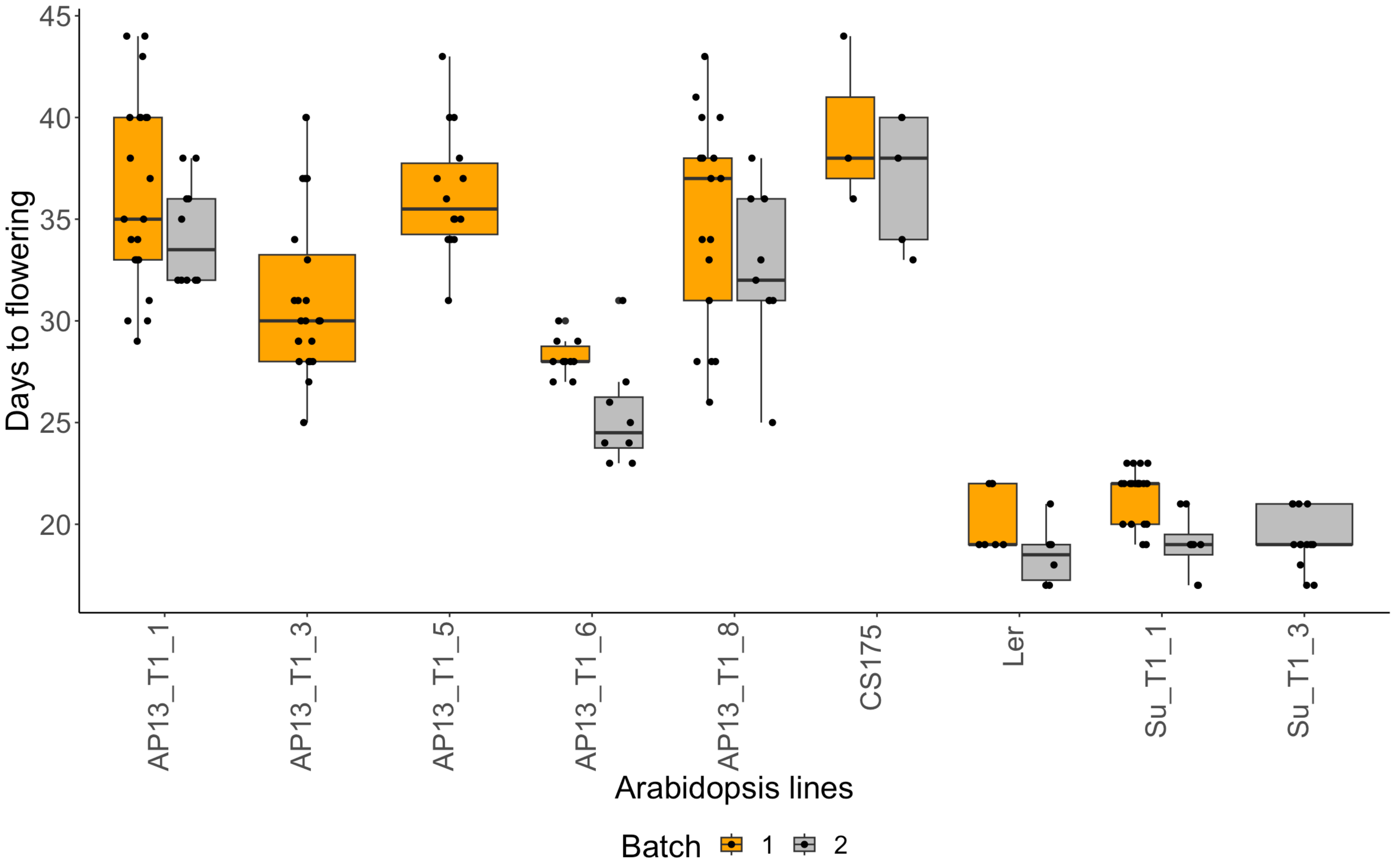

Supplementary Fig. S4. Box plot showing bolting in *Arabidopsis* wild-type (L*er*), CO-null mutant (CS175) and T_2 transgenic progeny derived from lines Su_T1_1 and Su_T1_3 (overexpressing the switchgrass *PvHd1-p.35S* allele) and from lines AP13_T1_1, AP13_T1_3, AP13_T1_5, AP13_T1_6 and AP13_T1_8 (overexpressing the *PvHd1-p.35G* allele) by harvest batch. Harvest batch 1 is indicated in orange, harvest batch 2 in grey. Significance of difference in 'days to flowering' is from the same statistics as in Fig. 3.A. Black dots in the boxplot indicate individual data points in each line and 'Batch'.

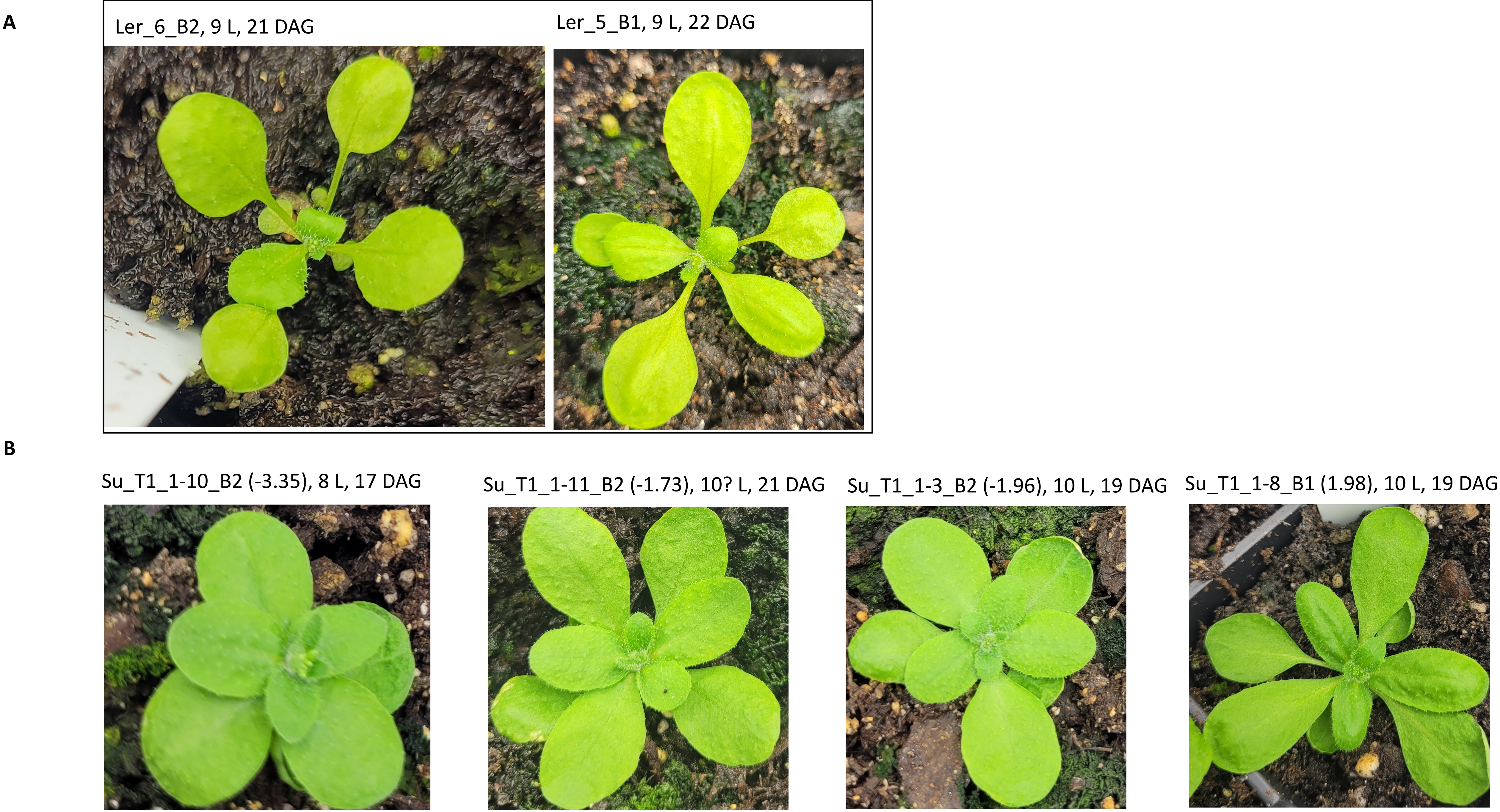
A
Ler_6_B2, 9 L, 21 DAG
Ler_5_B1, 9 L, 22 DAG
B
Su_T1_1-10_B2 (-3.35), 8 L, 17 DAG
Su_T1_1-11_B2 (-1.73), 10? L, 21 DAG
Su_T1_1-3_B2 (-1.96), 10 L, 19 DAG
Su_T1_1-8_B1 (1.98), 10 L, 19 DAG

C

Su_T1_3-16_B2 (-2.22), 10 L, 17 DAG

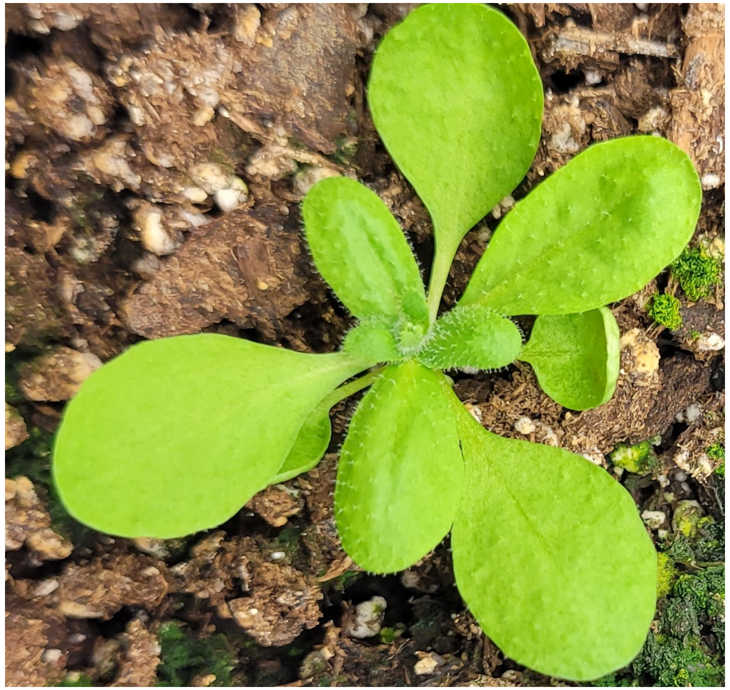

Su_T1_3-17_B2 (-1.61), 9 L, 18 DAG

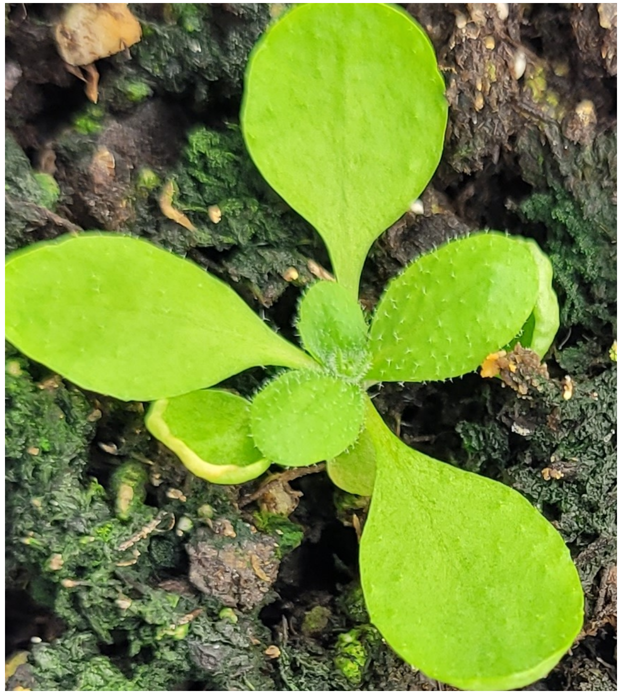

Su_T1_3-7_B2 (-1.48), 10-11? L, 21 DAG

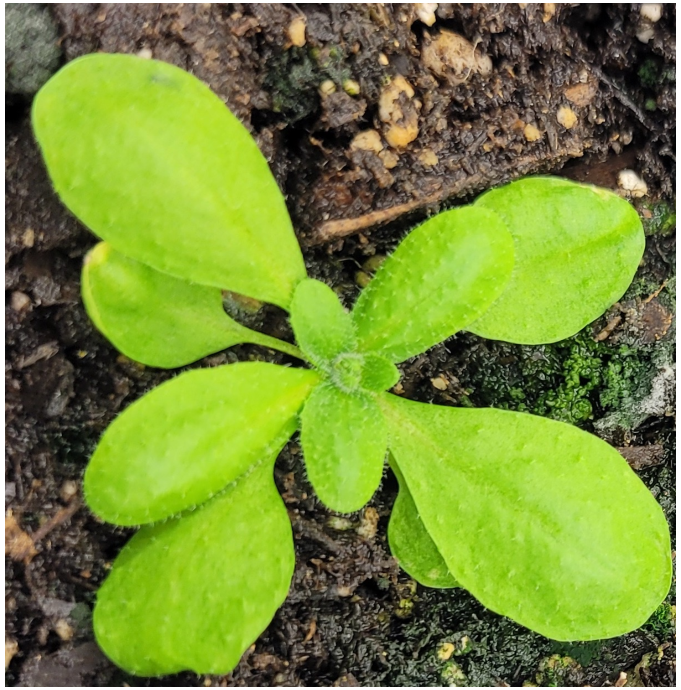

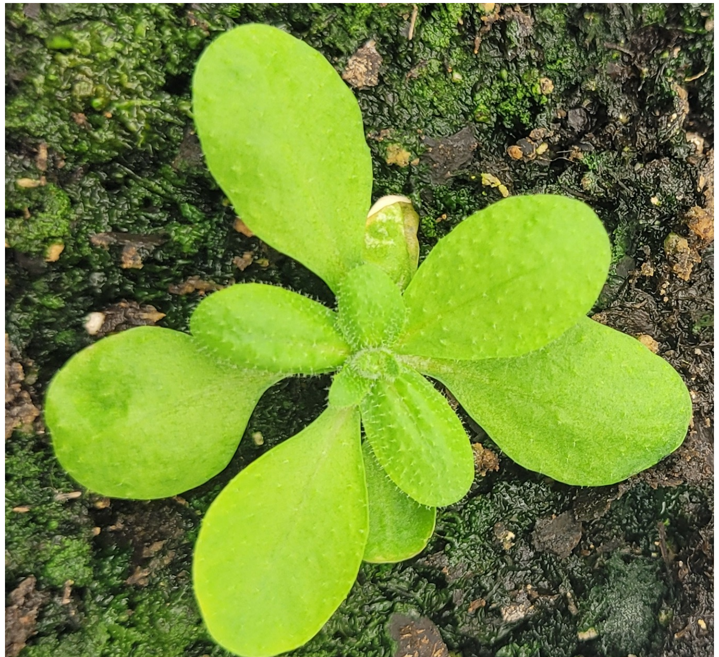

Su_T1_3-8_B2 (-1.17), 11 L, 21 DAG

D

CS175_1_B1, >13 L, 36 DAG

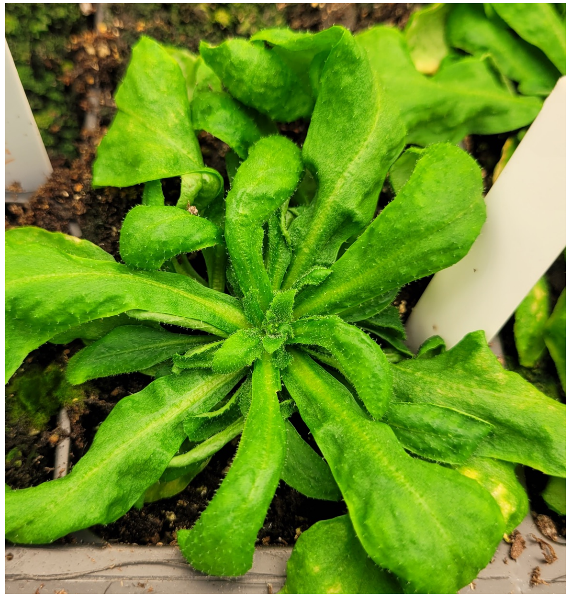

E

AP13_T1_1-14_B1 (-1.27), 13-14 L, 30 DAG

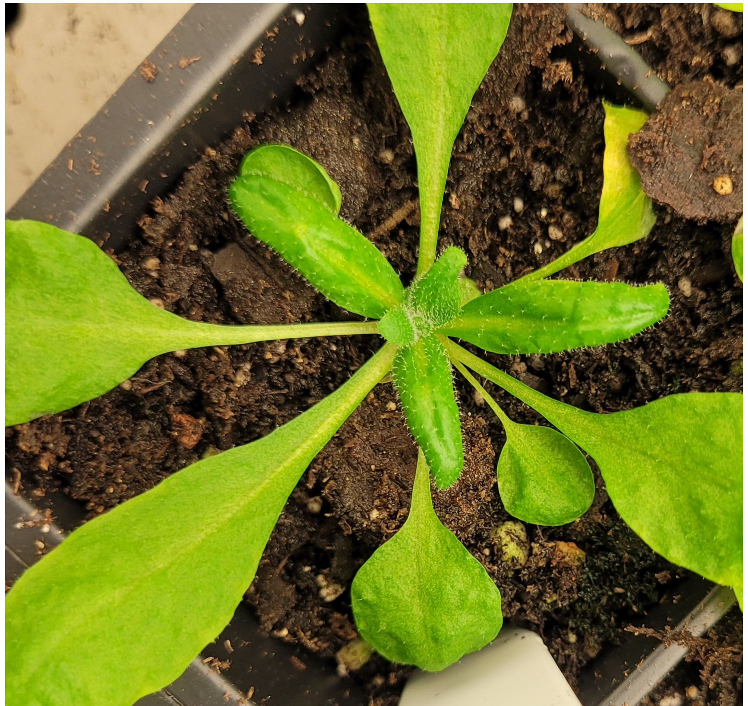

AP13_T1_1-4_B2 (8.48), 13-14 L, 38 DAG

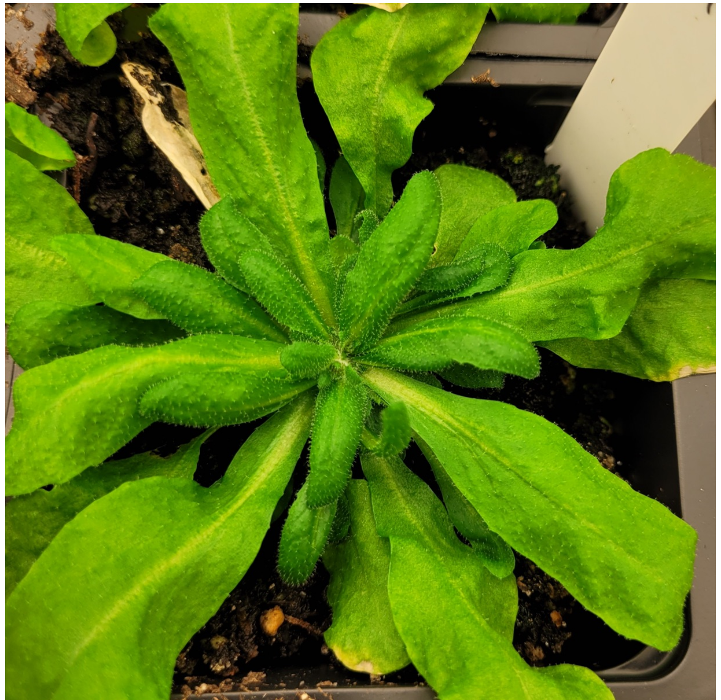

AP13_T1_1-16_B2, >13 L, 32 DAG

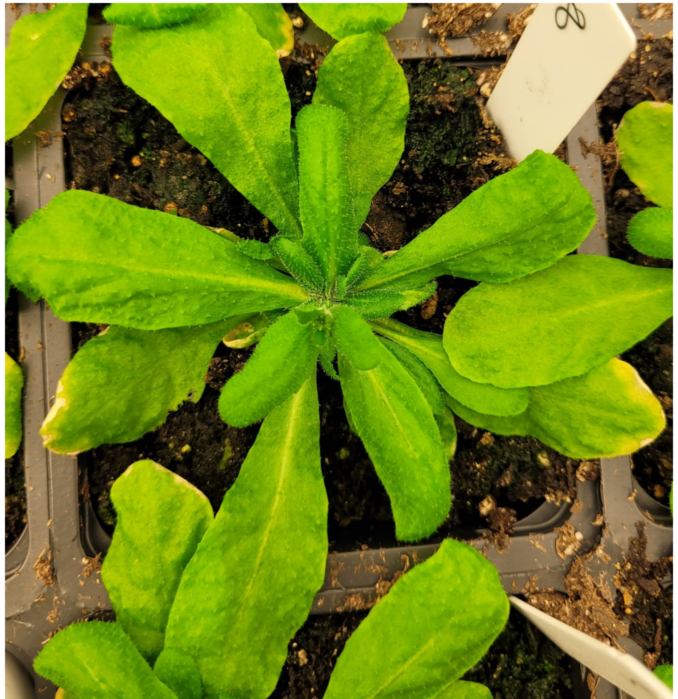

F

AP13_T1_6-6_B2 (-1.24), 13 L, 24 DAG

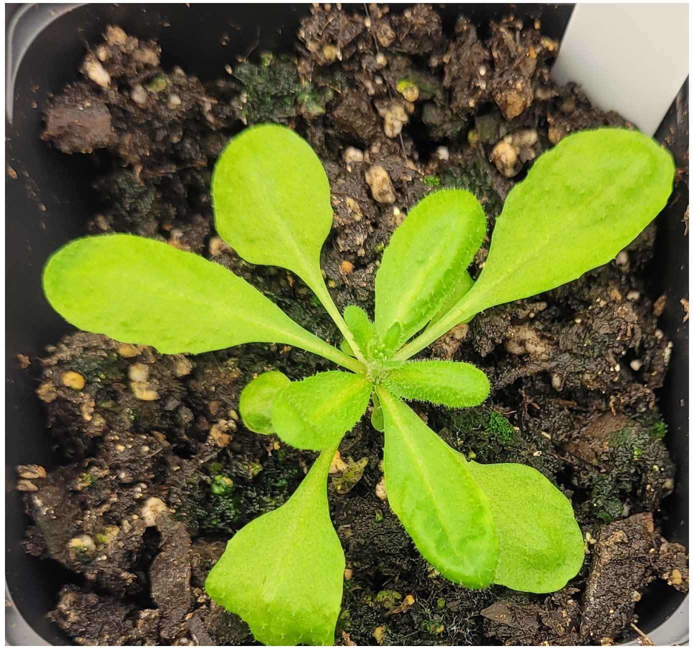

AP13_T1_6-9_B2 (-1.76), 13 L, 26 DAG

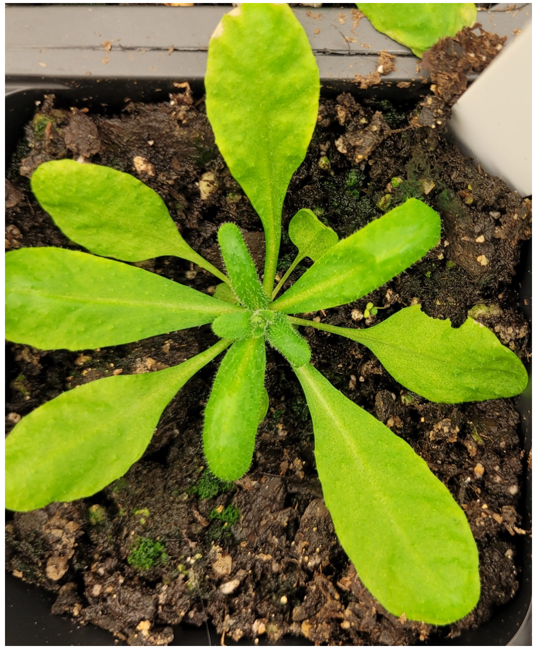

AP13_T1_6-10_B2 (1.88), 17 L, 31 DAG

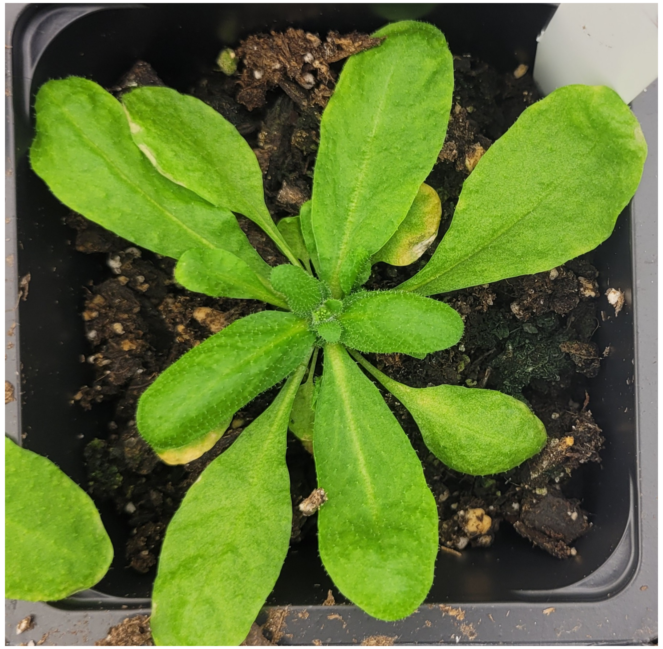

G

AP13_T1_8-10_B2 (-0.03), 13 L, 25 DAG

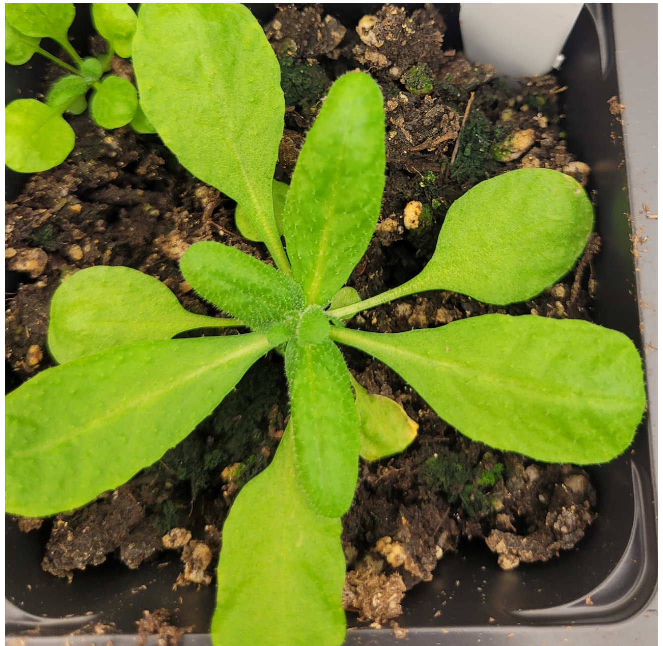

AP13_T1_8-4_B2 (0.52), >13 L, 36 DAG

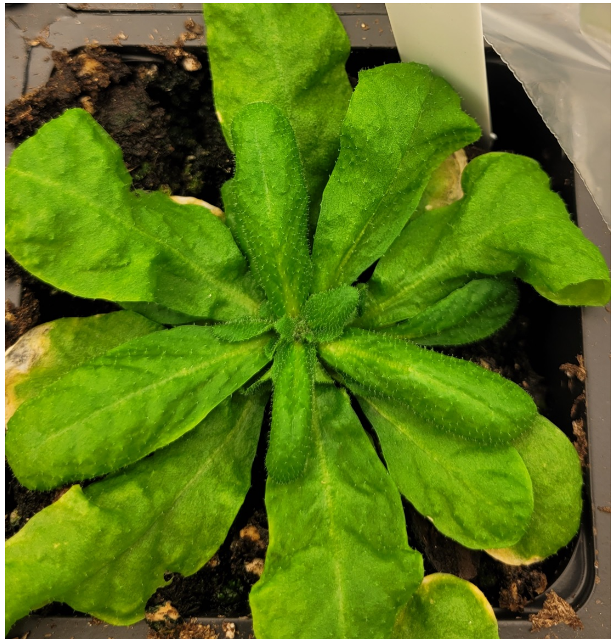

AP13_T1_8-6_B2 (0.75), 15? L, 31 DAG

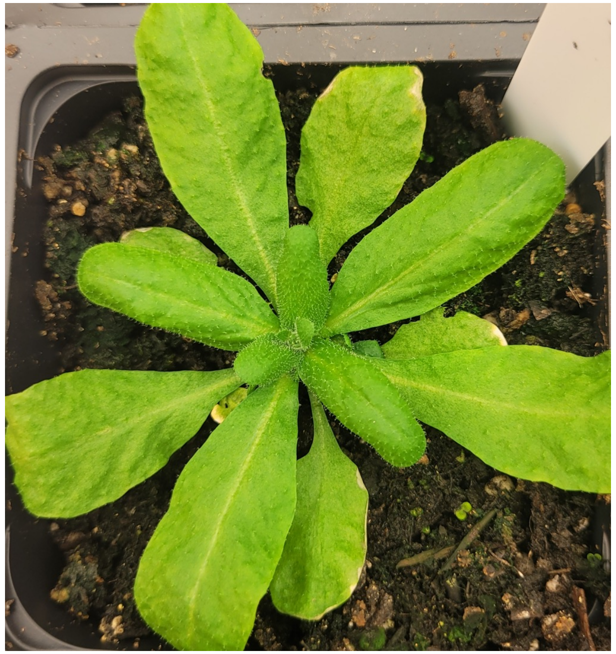

H

AP13_T1_3-10_B1, 14 L, 28 DAG

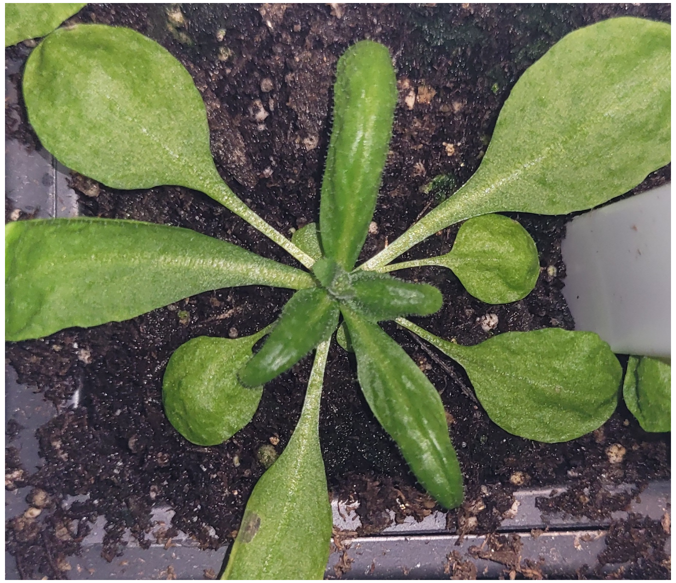

AP13_T1_3-21_B1, 13 L, 28 DAG

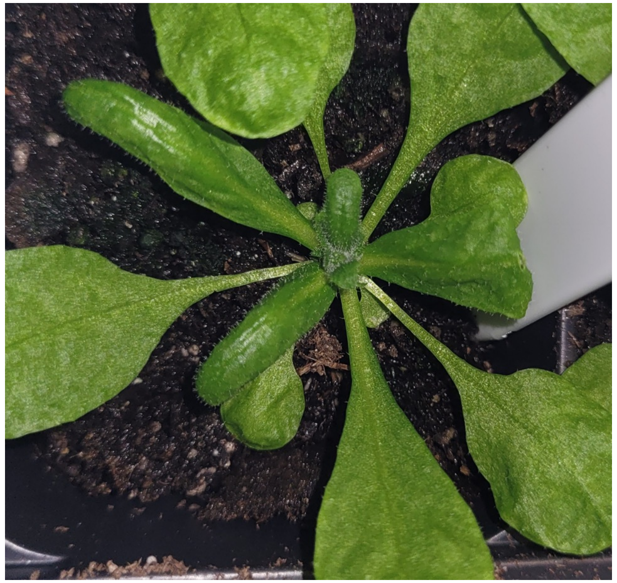

AP13_T1_3-20_B1, >13 L, 30 DAG

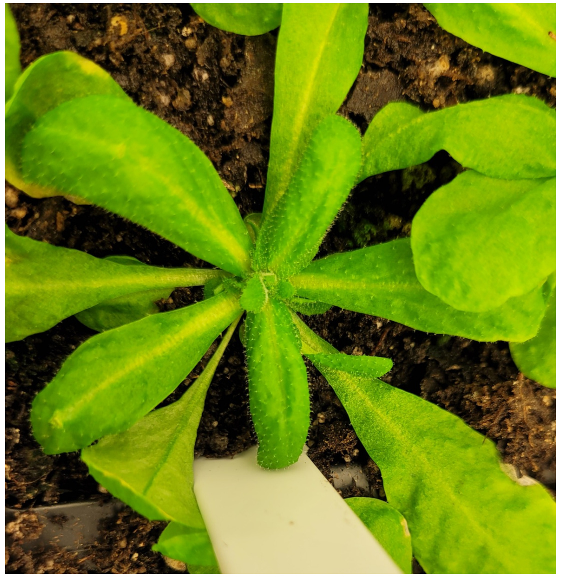

AP13_T1_3-9_B1, >13 L, 34 DAG

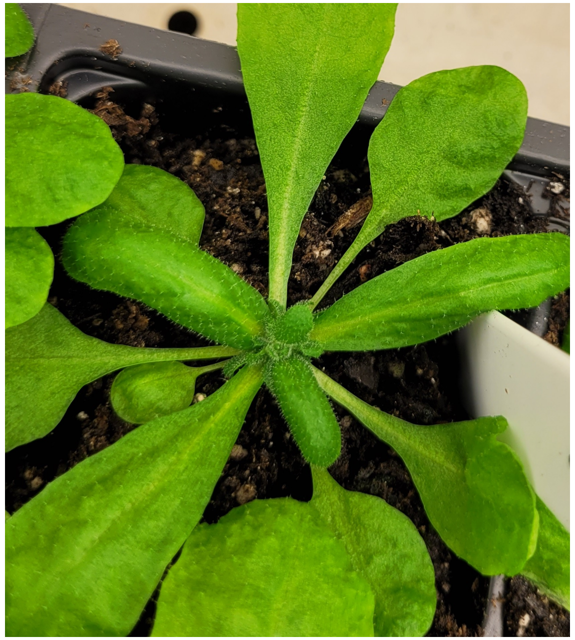

I

AP13_T1_5-12_B1, >13 L, 31 DAG

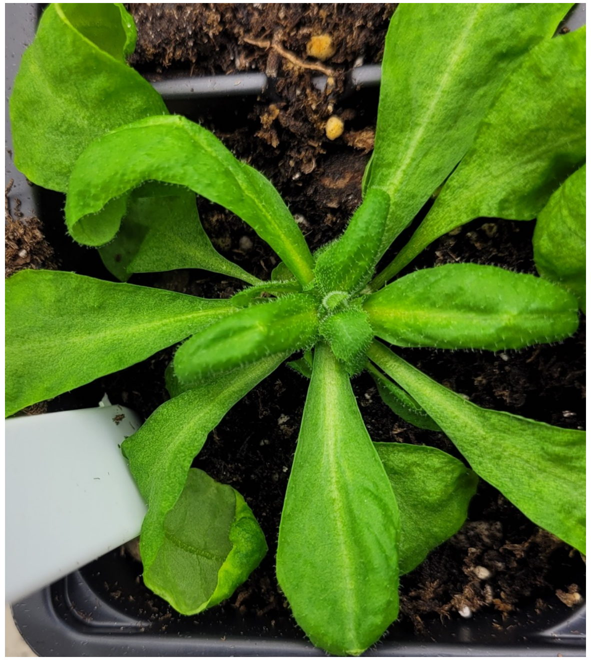

AP13_T1_5-7_B1, >13 L, 34 DAG

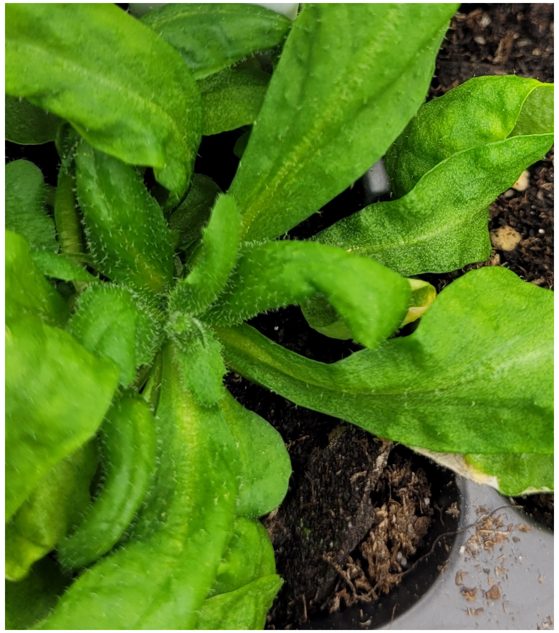

AP13_T1_5-1_B1, >13 L, 35 DAG

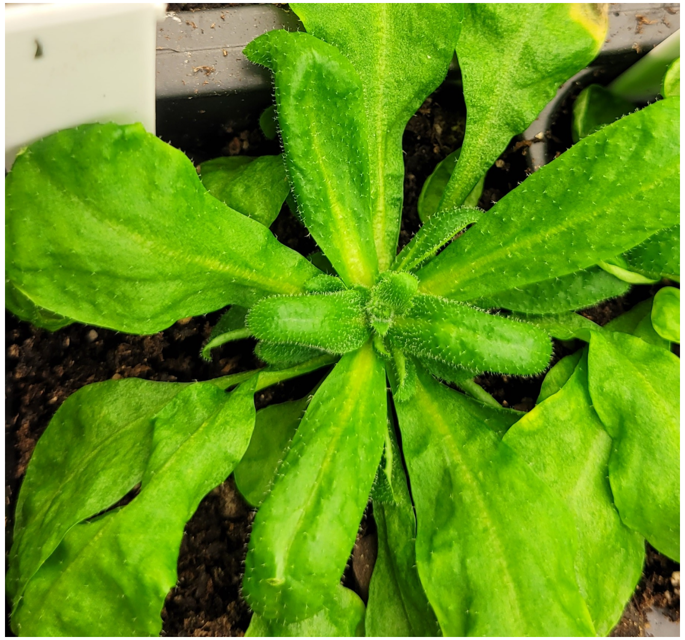

Supplementary Fig. S5. Representative phenotypes at bolting of different *Arabidopsis* lines and T_2 transformants with **(A)** L*er* plants; **(B)** Su_T1_1-derived T_2 plants ; **(C)** Su_T1_3-derived T_2 plants; **(D)** CS175 plant; **(E)** AP13_T1_1-derived T_2 plants; **(F)** AP13_T1_3-derived T_2 plants; **(G)** AP13_T1_5-derived T_2 plants; **(H)** AP13_T1_6-derived T_2 plants and **(I)** AP13_T1_8-derived T_2 plants. Where available, averaged dCq (Cq_{PvHd1}-Cq_{Actin}) values (also presented in Fig. 3.B) are given in parenthesis.
'L' = number of rosette leaves. High leaf densities are indicated as '> 13L', with 13 being the lowest leaf number observed at bolting in *p.35G*-OE lines'. 'DAG' = Days after germination

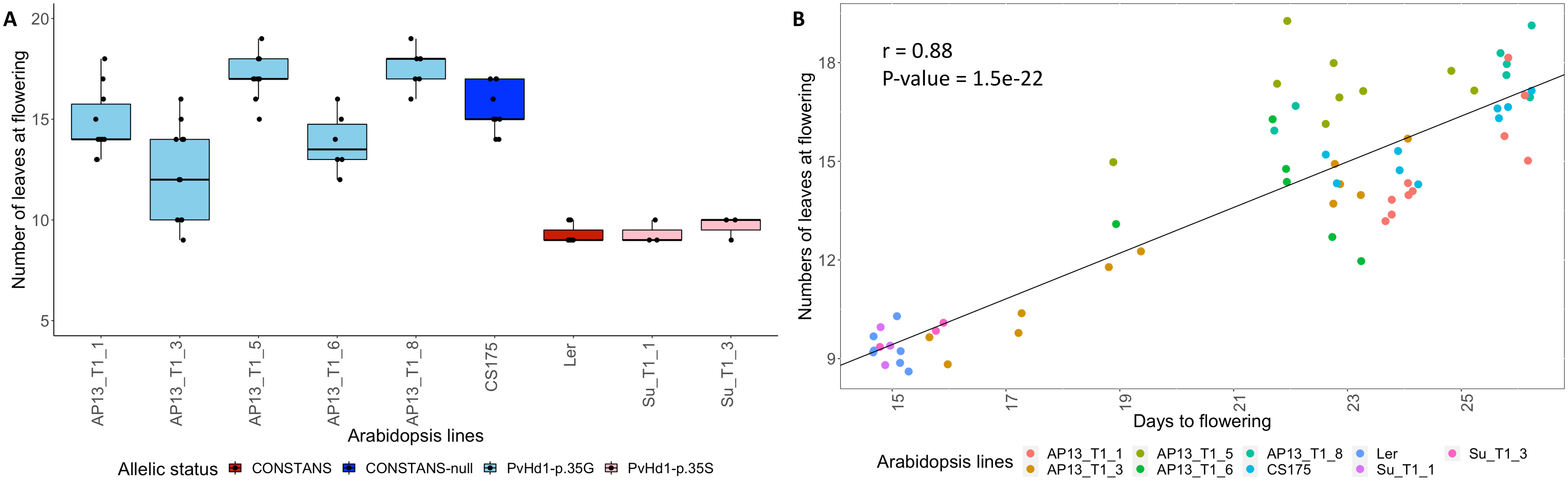

Supplementary Fig. S6. Leaf numbers at bolting in the *Arabidopsis* lines and their correlation with 'days to flowering'. **(A)** Box plot showing leaf numbers at bolting in *Arabidopsis* wild-type (L*er*), CONSTANS-null mutant (CS175) and T_2 transgenic progeny derived from lines AP13_T1_1, AP13_T1_3, AP13_T1_5, AP13_T1_6 and AP13_T1_8 (overexpressing the *PvHd1-p.35G* allele) and from lines Su_T1_1 and Su_T1_3 (overexpressing the switchgrass *PvHd1-p.35S* allele). Black dots indicate the individual data points in each line. **(B)** Scatter plot showing the positive correlation between 'Numbers of leaves at flowering' and 'Days to flowering' in the *Arabidopsis* lines analyzed in **Fig. S6.A**. The black diagonal line indicates the slope of the correlation.

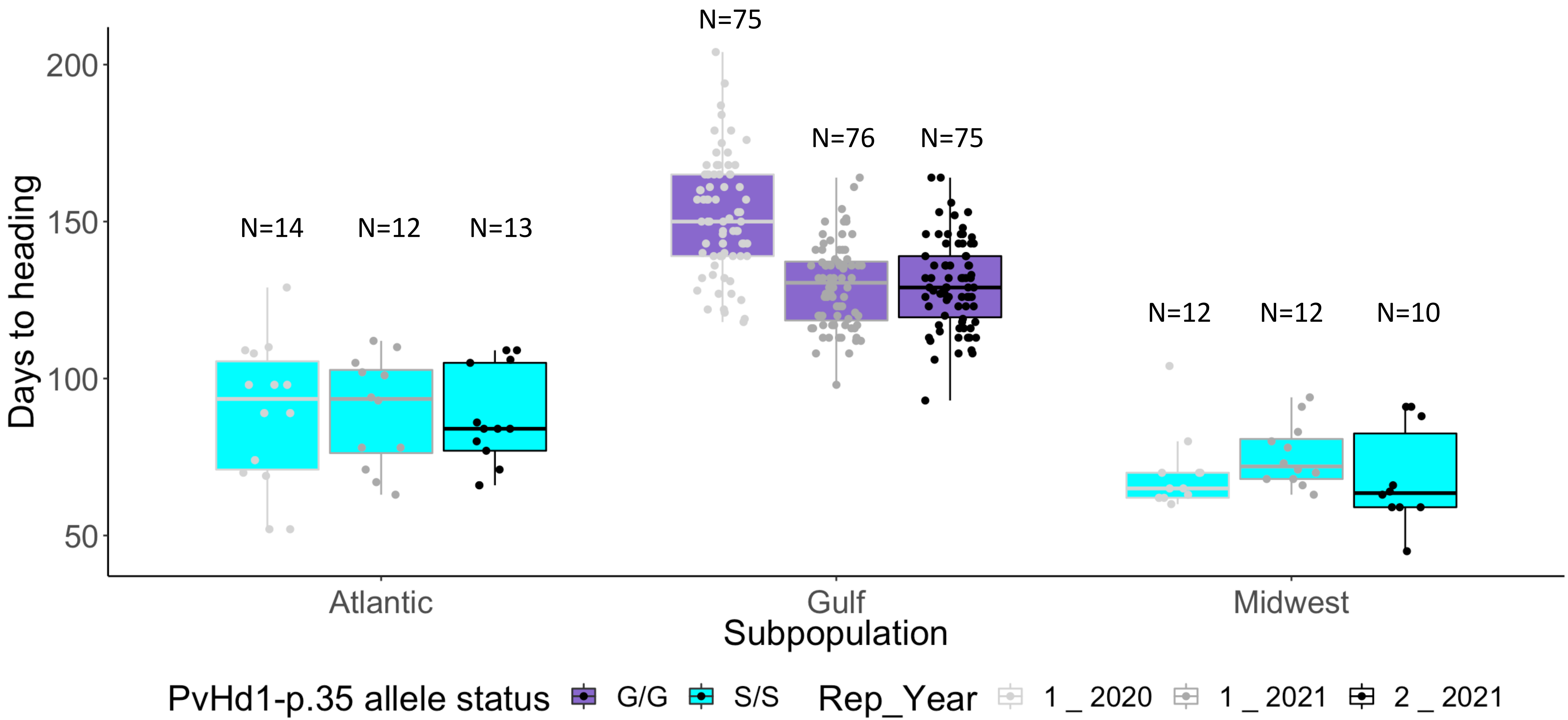

Supplementary Fig. S7. Box plot showing days to heading recorded in 2020 (Rep1) and 2021 (Rep1 and Rep2) for accessions belonging to three genetic subpopulations in a GWAS panel established at the Iron Horse Farm in Watkinsville, GA. Light grey, dark grey and black dots in the box plot show individual data points for Rep1 (2020), Rep1 (2021) and Rep2 (2021), respectively. Subpopulation membership was obtained from Lovell *et al.* (2021).

Lovell JT, MacQueen AH, Mamidi S, et al. 2021. Genomic mechanisms of climate adaptation in polyploid bioenergy switchgrass. Nature 590, 438–444.

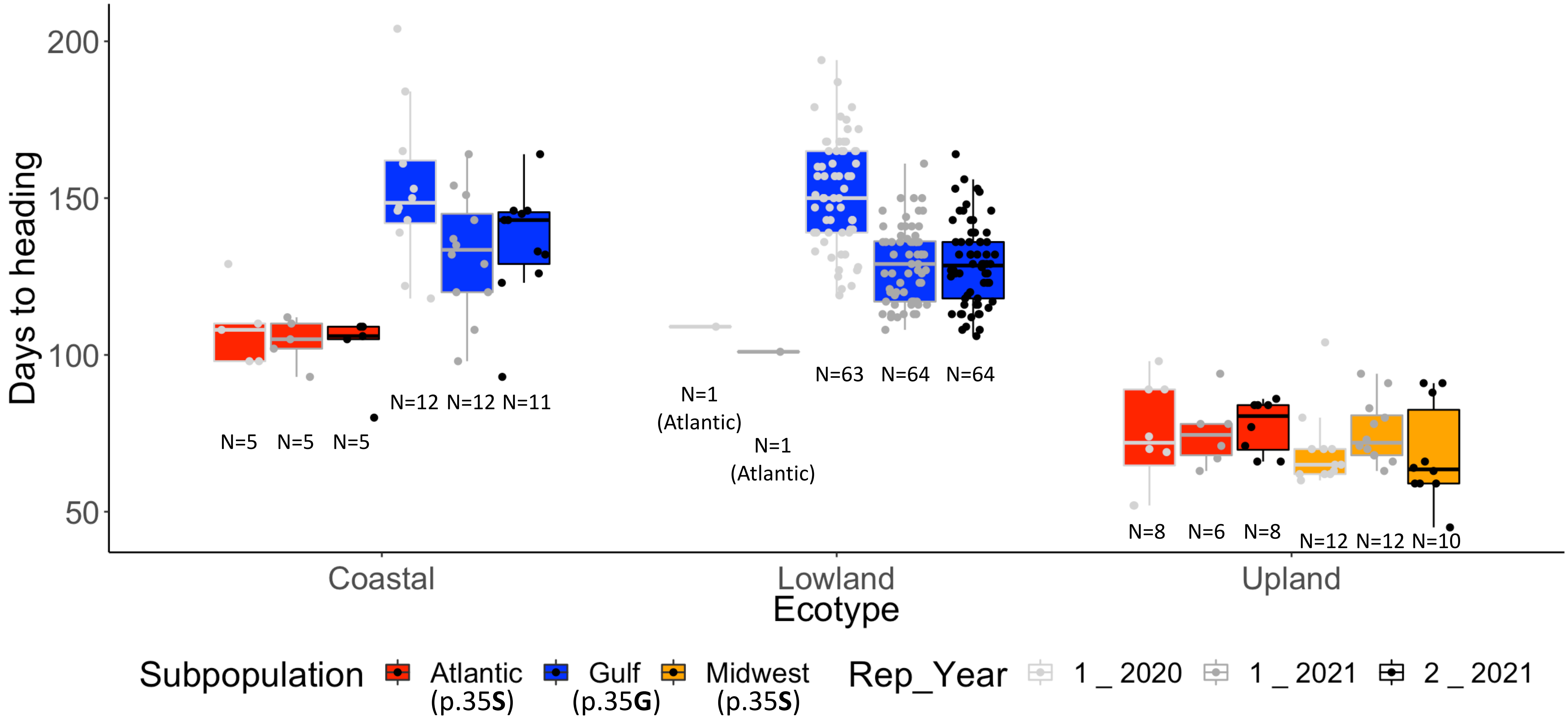

Supplementary Fig. S8. Box plot showing days to heading recorded in 2020 (Rep1) and 2021 (Rep1 and Rep2) for accessions belonging to three ecotypes within genetic subpopulations in a GWAS panel established at the Iron Horse Farm in Watkinsville, GA. Light grey, dark grey and black dots in the box plot show individual data points for Rep1 (2020), Rep1 (2021) and Rep2 (2021), respectively. Ecotype and subpopulation membership were obtained from Lovell *et al.* (2021). Allelic status of PvHd1 in each subpopulation is present in parentheses under the color codes for the subpopulations.

Lovell JT, MacQueen AH, Mamidi S, et al. 2021. Genomic mechanisms of climate adaptation in polyploid bioenergy switchgrass. Nature 590, 438–444.
