# Supplementary material for: A single amino acid change led to structural and functional differentiation of PvHd1 to control flowering in switchgrass
Source: J Exp Bot. 2023 Jul 4;74(18):5532–46. doi: 10.1093/jxb/erad255 (PMC10540729; doi:10.1093/jxb/erad255)
Supplement: erad255_suppl_Supplementary_Tables_S1_S3_S6_S9-10 [file erad255_suppl_supplementary_tables_s1_s3_s6_s9-10.docx]

| **Supplementary Table S1.** Primers used in the study | |
| --- | --- |
| Primer_name | Sequence (5'- -3') |
| 4KG163000_SacI | gcagagctcATGAATTATA |
| 4KG163000_BamHI | tgcggatccTCAGAACCAT |
| pS-OX_promoter_F | CAGTGGTCCCAAAGATGGAC |
| pS-OX_RB_R | CCGCCAATATATCCTGTCA |
| PvHd1_inside_F | TCAGTGCTTACACCGATTCC |
| PvHd1_CDS_R | TCAGAACCATGGGACAGT |
| ACN_F | ACCTTTAACTCTCCCGCTA |
| ACN_R | CAAGGTCAAGACGGAGGAT |
| PvHd1_CDS_F | ATGAATTATAATTTTGGCAG |
| PvHd1_qPCR_R | CAGAGGTACGCAGCGTCAG |
| attB1-pvHd1_F | GGGGACAAGTTTGTACAAAAAGCAGGCTCCATGAATTATAATTTTGGCAG |
| attB2-pvHd1_R | GGGGACCACTTTGTACAAGAAAGCTGGGTCTCAGAACCATGGGACAGT |
| PvHd1_stdiff_R3 | CAGAGTGCACCTTGGCGTCA |

**Supplementary Table S2**. Flowering time traits measured for three consecutive years (2017-2019) in a biparental (AP13 x VS16) F_2_ mapping population (Qi *et al.*, 2021) planted at the Iron Horse Farm in Watkinsville, GA

Provided as an excel file

| **Supplementary Table S3.** Average trait values for plants homozygous for the AP13 allele (delayed-flowering), homozygous for the VS16 allele (earlier-flowering) and heterozygous at the flowering time QTL on Chr04K. | | | | | |
| --- | --- | --- | --- | --- | --- |
| Traits | Tag at the highest LR | AP13 | VS16 | Heterozygous | AP13-VS16 |
| Hd_2019_Rep3 | Tag_4384 | 167.4 | 162.3 | 164.3 | 5.1 |
| Hd_2019_Rep2 | Tag_4322 | 166.4 | 161.9 | 163.2 | 4.5 |
| Hd_2019_Rep1 | Tag_4246 | 168.2 | 164.8 | 164.4 | 3.4 |
| Hd_2017_Rep3 | Tag_4385 | 163.1 | 155.0 | 159.0 | 8.2 |
| Hd_2017_Rep2 | Tag_4385 | 163.5 | 155.5 | 159.0 | 8.0 |
| Hd_2017_Rep1 | Tag_4384 | 164.8 | 156.5 | 159.4 | 8.2 |
| Hd_Em_2019_Rep3 | Tag_4372 | 94.3 | 85.9 | 89.7 | 8.5 |
| Hd_Em_2019_Rep2 | Tag_4435 | 93.4 | 86.5 | 88.8 | 6.8 |
| Hd_Em_2019_Rep1 | Tag_4353 | 94.9 | 88.3 | 90.4 | 6.7 |
| Hd_Em_2017_Rep3 | Tag_4384 | 81.3 | 74.4 | 77.2 | 6.9 |
| Hd_Em_2017_Rep2 | Tag_4385 | 82.9 | 75.1 | 78.4 | 7.8 |
| Hd_Em_2017_Rep1 | Tag_4385 | 82.5 | 73.9 | 76.7 | 8.6 |
| An_Hd_2019_Rep2 | Tag_4569 | 18.1 | 15.9 | 17.9 | 2.1 |
| An_2019_Rep2 | Tag_4322 | 184.9 | 178.3 | 180.4 | 6.6 |
| An_2018_Rep2 | Tag_4387 | 189.1 | 186.3 | 186.7 | 2.8 |
|  |  |  |  |  |  |
| Phenotype annotation: ordinal emergence date (Em), ordinal heading date (Hd), days to heading (Hd-Em), ordinal anthesis date (An) and days to anthesis (An-Hd). | | | | | |

**Supplementary Table S4.** Annotated genes (Lovell *et al.*, 2021) underlying the flowering time QTL on chromosome 4K

Provided as an excel file

**Supplementary Table S5**. Flowering time of *PvHd1* allele-overexpressing T_2_ plants in CO-null L*er*, L*er* and CS175 (CO-null)

Provided as an excel file

| **Supplementary Table S6.** Post-hoc Tukey testing results (Lenth, 2022; R Core Team, 2022) showing the significance of differences in flowering time of PvHd1-OE transgenic lines relative to Ler and CS175 (data shown in Figure 3.a) | | | | | | |
| --- | --- | --- | --- | --- | --- | --- |
| Comparisons | estimate | SE | df | t.ratio | p.value |  |
| AP13_T1_1 - CS175 | -2.9010101 | 1.32486304 | 166 | -2.1896679 | 0.41727879 |  |
| AP13_T1_1 - Ler | 15.8926641 | 1.09607872 | 166 | 14.4995645 | 7.24E-14 |  |
| AP13_T1_3 - CS175 | -8.0787541 | 1.40533383 | 166 | -5.7486371 | 1.50E-06 |  |
| AP13_T1_3 - Ler | 10.71492 | 1.17469523 | 166 | 9.12144677 | 1.67E-13 |  |
| AP13_T1_5 - CS175 | -2.9716113 | 1.51670587 | 166 | -1.9592535 | 0.5738352 |  |
| AP13_T1_5 - Ler | 15.8220629 | 1.3058876 | 166 | 12.1159454 | 7.87E-14 |  |
| AP13_T1_6 - CS175 | -11.350529 | 1.40961941 | 166 | -8.052194 | 5.54E-12 |  |
| AP13_T1_6 - Ler | 7.44314519 | 1.20362392 | 166 | 6.18394588 | 1.68E-07 |  |
| AP13_T1_8 - CS175 | -4.4082134 | 1.34823728 | 166 | -3.2696125 | 0.03462874 |  |
| AP13_T1_8 - Ler | 14.3854608 | 1.12555747 | 166 | 12.7807431 | 7.32E-14 |  |
| CS175 - Ler | 18.7936742 | 1.48942107 | 166 | 12.6181068 | 7.36E-14 |  |
| CS175 - Su_T1_1 | 17.8595109 | 1.33845979 | 166 | 13.3433302 | 7.24E-14 |  |
| CS175 - Su_T1_3 | 17.7719783 | 1.50427859 | 166 | 11.8142865 | 8.53E-14 |  |
| Ler - Su_T1_1 | -0.9341632 | 1.10979141 | 166 | -0.8417467 | 0.99537269 |  |
| Ler - Su_T1_3 | -1.0216959 | 1.33994783 | 166 | -0.7624893 | 0.99767421 |  |
| Su_T1_1 - Su_T1_3 | -0.0875327 | 1.19286979 | 166 | -0.0733799 | 1 |  |
|  |  |  |  |  |  |  |
| Lenth R v. 2022. emmeans: estimated marginal means, aka least-squares means. https://CRAN.R-project.org/package=emmeans | | | | | |  |
| R Core Team. 2022. R: A language and environment for statistical computing. https://www.R-project.org/. | | | | | |  |

**Supplementary Table S7**. Cq values measured in *PvHd1*-overexpressing T_2_ transgenic plants in CO-null L*er* plants.

Provided as an excel file

**Supplementary Table S8.** Days to heading in a switchgrass diversity panel (Lovell *et al.*, 2021) and the p.35 variant present in PvHd1 which locates at position 19,031,195 on Chr04K in the AP13 genome assembly v5.1 (Lovell *et al.*, 2021).

Provided as an excel file

| **Supplementary Table S9.** Post-hoc Tukey testing results (Lenth, 2022; R Core Team, 2022) showing the significance of differences in days to heading recorded in 2021 (Rep1 and Rep2) for accessions belonging to three genetic subpopulations in a GWAS panel (Lovell *et al.*, 2021) established at the Iron Horse Farm in Watkinsville, GA (data shown in Figure 4.a) | | | | | |
| --- | --- | --- | --- | --- | --- |
| Comparisons | estimate | SE | df | t.ratio | p.value |
| Atlantic - Gulf | -41.763353 | 3.01772173 | 194 | -13.839365 | 1.61E-14 |
| Atlantic - Midwest | 15.8173625 | 4.08712397 | 194 | 3.87004715 | 0.00043491 |
| Gulf - Midwest | 57.5807154 | 3.19009134 | 194 | 18.0498642 | 1.61E-14 |
|  |  |  |  |  |  |
| Subpopulation membership was obtained from Lovell *et al*. (2021). | | | | | |
| Lovell JT, MacQueen AH, Mamidi S, et al. 2021. Genomic mechanisms of climate adaptation in polyploid bioenergy switchgrass. Nature 590, 438–444. | | | | | |
| Lenth R v. 2022. emmeans: estimated marginal means, aka least-squares means. https://CRAN.R-project.org/package=emmeans | | | | | |
| R Core Team. 2022. R: A language and environment for statistical computing. https://www.R-project.org/. | | | | | |

| **Supplementary Table S10.** Post-hoc Tukey testing results (Lenth, 2022; R Core Team, 2022) showing the significance of differences in days to heading recorded in 2021 (Rep1 and Rep2) for accessions belonging to three ecotypes within genetic subpopulations in a GWAS panel (Lovell *et al.*, 2021) established at the Iron Horse Farm in Watkinsville, GA (data shown in Figure 4.b) | | | | | |
| --- | --- | --- | --- | --- | --- |
| Comparisons | estimate | SE | df | t.ratio | p.value |
| Atlantic Coastal - Atlantic Lowland | 2.1 | 13.7972259 | 191 | 0.15220451 | 0.99999999 |
| Atlantic Coastal - Atlantic Upland | 26.7428571 | 5.44674489 | 191 | 4.90987878 | 6.75E-05 |
| Atlantic Coastal - Gulf Coastal | -31.030435 | 4.98297242 | 191 | -6.2272941 | 1.06E-07 |
| Atlantic Coastal - Gulf Lowland | -25.923622 | 4.32069743 | 191 | -5.9998698 | 3.47E-07 |
| Atlantic Coastal - Midwest Upland | 30.8272727 | 5.01717304 | 191 | 6.14435111 | 1.64E-07 |
| Atlantic Lowland - Atlantic Upland | 24.6428571 | 13.6168622 | 191 | 1.80973096 | 0.67573487 |
| Atlantic Lowland - Gulf Lowland | -28.023622 | 13.206829 | 191 | -2.1219039 | 0.46161177 |
| Atlantic Lowland - Midwest Upland | 28.7272727 | 13.4507967 | 191 | 2.1357302 | 0.4523169 |
| Atlantic Upland - Midwest Upland | 4.08441558 | 4.49750157 | 191 | 0.90815212 | 0.99230607 |
| Gulf Coastal - Atlantic Lowland | 33.1304348 | 13.4380773 | 191 | 2.46541481 | 0.25570096 |
| Gulf Coastal - Atlantic Upland | 57.7732919 | 4.45931712 | 191 | 12.9556366 | 0 |
| Gulf Coastal - Gulf Lowland | 5.10681274 | 2.98109134 | 191 | 1.71306819 | 0.7378636 |
| Gulf Coastal - Midwest Upland | 61.8577075 | 3.92307336 | 191 | 15.7676652 | 0 |
| Gulf Lowland - Atlantic Upland | 52.6664792 | 3.70458112 | 191 | 14.2165814 | 0 |
| Gulf Lowland - Midwest Upland | 56.7508948 | 3.03791323 | 191 | 18.6808808 | 0 |
|  |  |  |  |  |  |
| Ecotype and subpopulation membership was obtained from Lovell *et al.* (2021) | | | | | |
| Lovell JT, MacQueen AH, Mamidi S, et al. 2021. Genomic mechanisms of climate adaptation in polyploid bioenergy switchgrass. Nature 590, 438–444. | | | | | |
| Lenth R v. 2022. emmeans: estimated marginal means, aka least-squares means. https://CRAN.R-project.org/package=emmeans | | | | | |
| R Core Team. 2022. R: A language and environment for statistical computing. https://www.R-project.org/. | | | | | |
